# Ensuring scientific reproducibility in bio-macromolecular modeling via extensive, automated benchmarks

**DOI:** 10.1038/s41467-021-27222-7

**Published:** 2021-11-29

**Authors:** Julia Koehler Leman, Sergey Lyskov, Steven M. Lewis, Jared Adolf-Bryfogle, Rebecca F. Alford, Kyle Barlow, Ziv Ben-Aharon, Daniel Farrell, Jason Fell, William A. Hansen, Ameya Harmalkar, Jeliazko Jeliazkov, Georg Kuenze, Justyna D. Krys, Ajasja Ljubetič, Amanda L. Loshbaugh, Jack Maguire, Rocco Moretti, Vikram Khipple Mulligan, Morgan L. Nance, Phuong T. Nguyen, Shane Ó Conchúir, Shourya S. Roy Burman, Rituparna Samanta, Shannon T. Smith, Frank Teets, Johanna K. S. Tiemann, Andrew Watkins, Hope Woods, Brahm J. Yachnin, Christopher D. Bahl, Chris Bailey-Kellogg, David Baker, Rhiju Das, Frank DiMaio, Sagar D. Khare, Tanja Kortemme, Jason W. Labonte, Kresten Lindorff-Larsen, Jens Meiler, William Schief, Ora Schueler-Furman, Justin B. Siegel, Amelie Stein, Vladimir Yarov-Yarovoy, Brian Kuhlman, Andrew Leaver-Fay, Dominik Gront, Jeffrey J. Gray, Richard Bonneau

**Affiliations:** 1 Center for Computational Biology, Flatiron Institute, Simons Foundation, New York, NY 10010 USA; 2 Department of Biology, New York University, New York, NY 10003 USA; 3 Department of Chemical and Biomolecular Engineering, Johns Hopkins University, Baltimore, MD 21218 USA; 4 Cyrus Biotechnology, 1201 Second Ave, Suite 900, Seattle, WA 98101 USA; 5 Department of Immunology and Microbiology, Scripps Research, La Jolla, CA 92037 USA; 6 IAVI Neutralizing Antibody Center, Scripps Research, La Jolla, CA 92037 USA; 7 Graduate Program in Bioinformatics, University of California San Francisco, San Francisco, CA 94158 USA; 8 Department of Microbiology and Molecular Genetics, Hebrew University, Hadassah Medical School, POB 12272, Jerusalem, 91120 Israel; 9 Department of Biochemistry, University of Washington, Seattle, WA 98195 USA; 10 Institute for Protein Design, University of Washington, Seattle, WA 98195 USA; 11 Genome Center, University of California, Davis, CA 95616 USA; 12 Department of Biochemistry & Molecular Medicine, University of California, Davis, CA 95616 USA; 13 Department of Chemistry, University of California, Davis, CA 95616 USA; 14 Department of Chemistry and Chemical Biology, Rutgers, The State University of New Jersey, Piscataway, NJ 08904 USA; 15 Institute for Quantitative Biomedicine, Rutgers, The State University of New Jersey, Piscataway, NJ 08904 USA; 16 Program in Molecular Biophysics, Johns Hopkins University, Baltimore, MD 21218 USA; 17 Department of Chemistry, Vanderbilt University, Nashville, TN 37235 USA; 18 Center for Structural Biology, Vanderbilt University, Nashville, TN 37235 USA; 19 Institute for Drug Discovery, Medical School, Leipzig University, 04103 Leipzig, Germany; 20 Faculty of Chemistry, Biological and Chemical Research Center, University of Warsaw, Pasteura 1, 02-093 Warsaw, Poland; 21 Department of Bioengineering and Therapeutic Sciences, University of California San Francisco, San Francisco, CA 94158 USA; 22 Biophysics Graduate Program, University of California San Francisco, San Francisco, CA 94158 USA; 23 Program in Bioinformatics and Computational Biology, University of North Carolina at Chapel Hill, Chapel Hill, NC 27599 USA; 24 Department of Physiology and Membrane Biology, School of Medicine, University of California, Davis, CA 95616 USA; 25 Chemical and Physical Biology Program, Vanderbilt University, Nashville, TN 37235 USA; 26 Department of Bioochemistry and Biophysics, University of North Carolina at Chapel Hill, Chapel Hill, NC 27516 USA; 27 Linderstrøm-Lang Centre for Protein Science, Department of Biology, University of Copenhagen, DK-2200 Copenhagen N., Denmark; 28 Department of Biochemistry, Stanford University School of Medicine, Stanford, CA 94305 USA; 29 Institute for Protein Innovation, Boston, MA 02115 USA; 30 Division of Hematology/Oncology, Boston Children’s Hospital, Boston, MA 02115 USA; 31 Department of Pediatrics, Harvard Medical School, Boston, MA 02115 USA; 32 Department of Computer Science, Dartmouth, Hanover, NH 03755 USA; 33 Department of Computer Science, New York University, New York, NY 10003 USA

**Keywords:** Protein structure predictions, Software, Proteins

## Abstract

Each year vast international resources are wasted on irreproducible research. The scientific community has been slow to adopt standard software engineering practices, despite the increases in high-dimensional data, complexities of workflows, and computational environments. Here we show how scientific software applications can be created in a reproducible manner when simple design goals for reproducibility are met. We describe the implementation of a test server framework and 40 scientific benchmarks, covering numerous applications in Rosetta bio-macromolecular modeling. High performance computing cluster integration allows these benchmarks to run continuously and automatically. Detailed protocol captures are useful for developers and users of Rosetta and other macromolecular modeling tools. The framework and design concepts presented here are valuable for developers and users of any type of scientific software and for the scientific community to create reproducible methods. Specific examples highlight the utility of this framework, and the comprehensive documentation illustrates the ease of adding new tests in a matter of hours.

## Abstract (web summary)

Computational methods are becoming an increasingly important part of biological research. Using the Rosetta framework as an example, the authors demonstrate how community-driven development of computational methods can be done in a reproducible and reliable fashion.

## Introduction

Reproducibility in science is a systemic problem. In a survey published by *Nature* in 2016, 90% of scientists responded that there is a reproducibility crisis^1^. Over 70% of the over 1500 researchers surveyed were unable to reproduce another scientist’s experiments and over half were unable to reproduce their own experiments. Another analysis published by *PLOS One* in 2015 concluded that, in the US alone, about half of preclinical research was irreproducible, amounting to a total of about $28 billion being wasted per year^2^!

Reproducibility in biochemistry lab experiments remains challenging to address, as it depends on the quality and purity of reagents, unstable environmental conditions, and the accuracy and skill with which the experiments are performed. Even small changes in input and method ultimately lead to an altered output. In contrast, computational methods should be inherently scientifically reproducible since computer chips perform computations in the same way, removing some variations that are difficult to control. However, in addition to poorly controlled computing environment variables, computational methods become increasingly complex pipelines of data handling and processing. This effect is further compounded by the explosion of input data through “big data” efforts and exacerbated by a lack of stable, maintained, tested, and well-documented software, creating a huge gap between the theoretical limit for scientific reproducibility and the current reality^3^.

These circumstances are often caused by a lack of best practices in software engineering or computer science^4,5^, errors in laboratory management during project or personnel transitions, and a lack of academic incentives for software stability, maintenance, and longevity^6^. Shifts in accuracy can occur when re-writing functionality or when several authors work on different parts of the codebase simultaneously. An increase in complexity of scientific workflows with many and overlapping options and variables can prevent scientific reproducibility, as can code implementations that lack or even prevent suitable testing^4^. The absence of testing and maintenance causes software erosion (also known as *bit rot*), leading to a loss of users and often the termination of a software project. Further, barriers are created through intellectual property agreements, competition, and refusal to share inputs, methods, and detailed protocols.

As an example, in 2011 the Open Science Collaboration in Psychology tried to replicate the results of 100 studies as part of the Reproducibility Project^7^. The collaboration consisting of 270 scientists could only reproduce 39% of study outcomes. Since then, some funding agencies and publishers have implemented data management plans or standards to improve reproducibility^8–11^, for instance, the FAIR data management principles^12^. Guidelines to enhance reproducibility^13,14^ are certainly applicable, are outlined in Table 3, and are discussed in detail in an excellent editorial^15^ describing the *Ten Year Reproducibility Challenge*^16^ that is published in its own reproducibility journal ReScience C^17^. Other efforts focus directly on improving the methods with which the researchers process their data—for instance, the Galaxy platform fosters accessibility, transparency, reproducibility, and collaboration in biomedical data analysis and sharing^13^.

Reproducibility is also impacted by *how* methods are developed. Comparing a newly developed method to established ones, or an improved method to a previous version is important to assess its accuracy and performance, monitor changes and improvements over time and evaluate the cost/benefit ratio for software products to commercial entities. However, biases in publishing positive results or improvements to known methods, in conjunction with errors in methodology or statistical analyses^18^, lead to an acute need to test methods via third parties. Often, methods are developed and tested on a specific benchmark set created for that purpose and will perform better on that dataset than methods not trained on that dataset. A rigorous comparison and assessment require the benchmark to be independently created from the method, which unfortunately is rarely the case. Compounding issues are lack of diversity in the benchmark set (towards easier prediction targets) and reported improvements smaller than the statistical variation of the predicted results. Guidelines on how to create a high-quality benchmark^19,20^ are outlined in Table 3 below.

Scientific reproducibility further requires a stable, maintainable, and well-tested codebase. Software testing is typically achieved on multiple levels^4,21^. Unit tests check for scientific correctness of small, individual code blocks, integration tests check an entire application by integrating various code blocks, and profile and performance tests ensure consistency in runtime and program simplicity. Scientific tests or benchmarks safeguard the scientific validity and accuracies of the predictions. They are typically only carried out during or after the development of a new method (static benchmarking), as they require domain expertise and rely on vast computational resources to test an application on a larger dataset. However, the accuracy and performance of a method depend on the test set, the details of the protocol (i.e., specific command lines, options, and variables), and the software version. To overcome the static benchmarking approach, blind prediction challenges such as the Critical Assessments in protein Structure Prediction^22^, PRediction of protein Interactions^23^, Functional Annotation^24^, Genome Interpretation^25^, RNA Puzzles^26^, and Continuous Automated Model EvaluatiOn^15,27^ hold double-blind competitions at regular intervals. While these efforts are valuable to drive progress in method development in the scientific community, participation often requires months of commitment and does not address the reproducibility of established methods over time.

The Rosetta macromolecular modeling suite^28,29^ has been developed for over 20 years by a global community with now hundreds of developers at over 70 institutions^4,30^. This history and growth required us to adopt many best practices in software engineering^4,29^, including the implementation of a battery of tests. A detailed description of our community, including standards and practices, has previously been provided^4^. Scientific tests are important to maintain prediction accuracies for our own community and our users (including commercial users whose licensing fees, in our case, support much of Rosetta’s infrastructure and maintenance). We further want to directly compare different protocols and implementations and monitor the effect of score function changes on the prediction results. For many years, Rosetta applications^31,32^ and score functions^33–36^ have been tested independently using the static benchmarking approach^20,37^, often with complete protocol captures^38,39^. The disadvantage of static benchmarking is that the results become outdated due to the lack of automation. Reproducibility becomes impossible due to a lack of preservation of inputs, options, environment variables, and data analyses over time.

This background highlights the challenges in rigorously and continuously testing how codebase changes affect the scientific validity of a prediction method while maintaining or improving scientific reproducibility. Running scientific benchmarks continuously (1) suffers from a lack of incentive to set up as the maintenance character of these tests collides with academic goals; (2) requires both scientific and programming/technical expertise to implement, interpret and maintain; (3) is difficult to interpret with pass/fail criteria; and (4) requires a continuous investment of considerable computational resources. Here, we address these challenges by introducing a general framework for continuously running scientific benchmarks for a large and increasing number of protocols in the Rosetta macromolecular modeling suite. We present the general setup of this framework, demonstrate how we solve each of the above challenges, and present the results of the individual benchmarks in the Supplementary Information of this paper, complete with detailed protocol captures. The results can be used as a baseline by anyone developing macromolecular modeling methods, and the code of this framework is sufficiently general to be integrated into other types of software. The design principles presented here can be used by anyone developing scientific software, independent of the size of the method. We highly encourage small software development groups to follow these guidelines, even though their technical and personnel setup might differ. Supplementary Note (1) describes several options that small groups have available to test their software with limited resources.

## Results

**Fig. 1:**
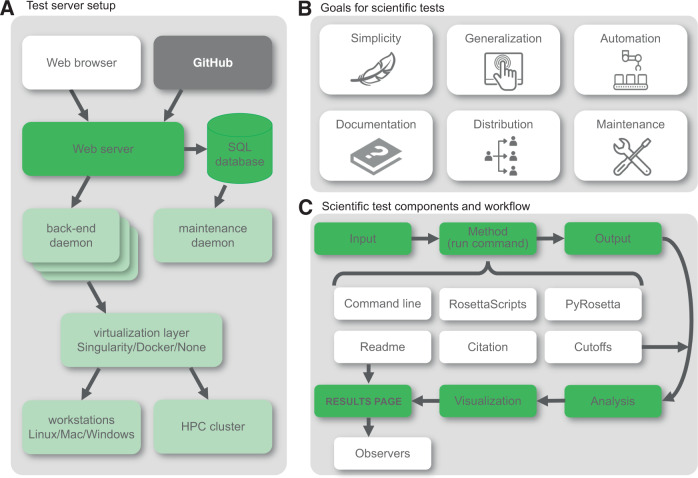
Goals and setups for the scientific tests. **A** Test server setup with the web browser as the user interface, the frontend in bright green, and the backend in light green. The code is stored in GitHub, shown in dark gray. **B** Specific goals for our scientific tests, driven by flaws in a previous iteration of these tests. Each point is described in detail in the text. **C** Basic infrastructure of the scientific test framework, motivated by simplicity. Each box represents a file, folder, or script that is either provided in the template folder or generated throughout the protocol run. The basic workflow is highlighted in green with components that facilitate documentation and maintenance shown in white. [Icons in Fig. 1B were created by Ana Teixeira, Aman, Ben Davis, Gregor Cresnar, Anna Sophie, and Joel Avery from Noun Project.] SQL structured query language, HPC cluster high-performance computing cluster.

Successful software development can be achieved by following a number of guidelines which are have previously been described in detail in ref. ^4^. Software testing is an essential part of this strategy which ties into scientific reproducibility. Over the past 15 years, the Rosetta community has created its own custom-built test server framework connected to a dedicated high-performance computing (HPC) cluster—its setup is shown in Fig. 1A and described in the Supplementary Information. The scientific testing setup is integrated into this framework.

### Insights from the previous round of scientific tests led to specific goals

The Rosetta community learned valuable lessons from the long-term maintenance (or lack thereof) of several scientific benchmark tests set up over 10 years ago (see Supplementary Note 2). Their deterioration and development life cycle motivated specific goals that we think lead to more durable scientific benchmarks (Fig. 1B): (1) simplicity of the framework to encourage maintenance and support; (2) Generalization to support all user interfaces to the Rosetta codebase (command line, RosettaScripts^40^, PyRosetta^41,42^); (3) automation to continuously run the tests on an HPC cluster with little manual intervention; (4) documentation on how to add tests and scientific details of each test to allow maintenance by anyone with a general science or Rosetta background; (5) distribution of the tests to both the Rosetta community and their users, and publicizing their existence to encourage the addition of new tests and maintenance by the community; and (6) maintenance of the tests, facilitated by each of the previous points.

### Goal 1—simplicity: simple setup facilitates broad adoption and support from our community

To encourage our community to contribute as many tests as possible, the testing framework needs to be simple and support fast and easy addition of tests. We decided on a Python framework that integrates well with our pre-existing testing HPC cluster (Supplementary Note 3). We further require these tests to be able to run on local machines (with different operating systems) as well as various HPC clusters with minimal adjustments. Debugging the scripts should be as simple as possible. With these requirements in mind, we decided on a setup as shown in Fig. 1C. We provide a template directory with all necessary files (described in detail in *Methods*). Simple modifications like naming scripts in the order in which they run—e.g., *0.compile.py* to *9.finalize.py*—greatly facilitate debugging or extension by new users.

### Goal 2—generalization: new tests support interfaces of the command line, PyRosetta, or RosettaScripts

Rosetta supports several interfaces to facilitate quick protocol development while lowering the necessary expertise required by new developers to join our community^4^. Many mainstream protocols have been developed as standalone applications to be run via the command line, while customized protocols have been developed in RosettaScripts^40^ and PyRosetta^41,42^. For our test server framework, we sought a general code design that allows input from all three interfaces while supporting different types of outputs, quality measures, and analyses, sometimes even written in different scripting languages.

### Goal 3—automation: tests require substantial compute power and are run on a dedicated test server

**Fig. 2:**
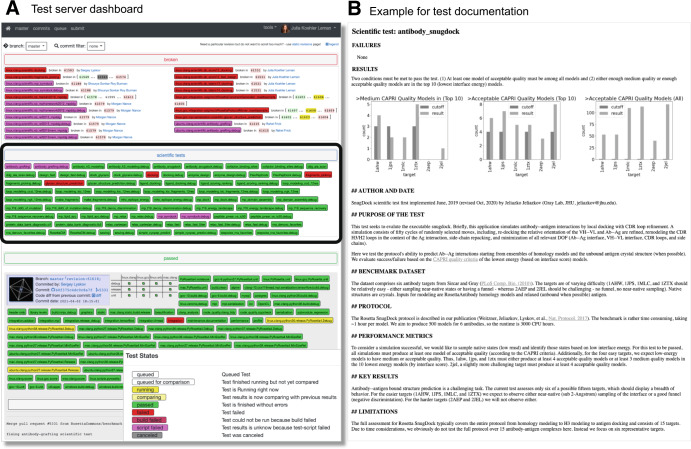
Webpages for the main dashboard and documentation of the tests. **A** Dashboard of our benchmark server testing infrastructure. Each test is colored according to its test results: red denotes breakage, magenta denotes script failure, green denotes passing of a test, yellow denotes the test is currently running, and white denotes the test has yet to be run. All broken tests are shown prominently at the top of the page. All scientific tests are shown in the blue tab below (also encircled in bold black). Tests of the latest revision merged into the main branch are shown below with information about the committer, the pull request ID, a link to the code difference, and the commit message. **B** The results page shows the results of the run, the documentation, and the description of whether the test passes or fails. Results pages are automatically generated at the end of the run for each test.

Running scientific benchmarks requires extensive CPU time; hence we chose to integrate them with our own custom-built test server framework connected to a dedicated HPC cluster (Fig. 1A and Supplementary Information). This test server framework consists of two main components: the backend holds low-level primitive code for compilation on different operating systems and HPC environments, cluster submission scripts, and web server integration code. The front end contains the test directories that are implemented by the test author. Our test server is accessible through a convenient web interface (Fig. 2A; available at https://benchmark.graylab.jhu.edu/). This framework has had a hugely positive impact on the growth and maintenance of both the Rosetta software and our community, due to its accessibility, GitHub integration, ease of use, and automation. In small software communities that lack the ability or resources to set up a dedicated test server, integration testing via external services like Github Actions^43^, Drone CI^44^, Travis CI^45^, or Jenkins^46^ is an excellent alternative. More details can be found in the Supplementary Information.

The RosettaCommons supports our benchmarking effort through expansion of our centralized test server cluster hardware and labor with an annual budget (see Supplementary Information and our previous publication^4^). Because the scientific tests are integrated into our test server framework, authors of the tests can focus on the scientific protocols (starting from a template directory set up as in Fig. 1C) instead of debugging errors in compilation, cluster submission, and computational environment. This pattern also makes these tests system-independent (the author writes the setup for a local machine and runs it on this server), i.e., portable between operating systems and computational environments. We currently limit the runtime per scientific test to typically 1000–2000 CPU hours.

Due to the required computational resources, we are unable to test every code revision in the main development branch of Rosetta; instead, we dedicate computational nodes to the scientific tests and run tests such that the nodes are continuously occupied. We found that scheduling the earliest-run test on an individual rolling basis, as compute nodes become available, is most efficient in balancing the server load while keeping nodes available for tests in feature branches. Upon discovery of a test failure and to find the specific revision (and therefore the code change) that caused the failure, our *bisect* tool schedules intermediate revisions on a low-priority basis. All test results are stored in the database and are accessible through a web interface (Fig. 2).

### Goal 4—documentation: anyone can quickly and easily add new tests

Creating well-designed scientific benchmarks requires expertise in defining the scientific objective, establishing a protocol, and creating a high-quality test dataset. The last step of incorporating the test into our framework should be as simple as possible (as per our *simplicity* requirement). Once the dataset, interface (command line, RosettaScripts, or PyRosetta), specific command line, and quality measures have been chosen, the author can simply follow the individual steps outlined on the documentation page^47^ to contribute the test; the template guides the setup (Supplementary Note 4). We found that the setup is simple enough that untrained individuals can contribute a test in a few hours based on documentation alone—hence we achieved our goal of simplicity and detail in our documentation.

One of the reasons for the deterioration of earlier scientific tests was lack of maintenance due to insufficient documentation. Our goal is to drive the creation of extensive documentation for each test such that anybody with an average scientific knowledge of biophysics and introductory knowledge of programming in Rosetta can understand and maintain the tests. To ensure comprehensive documentation and consistency between tests, we provide a readme template with specific sections and questions that need to be answered for each test (see Supplementary Information). The template discourages writing short, insufficient, free-form documentation, and instead encourages the addition of important details and significantly lowers the barrier for writing extensive documentation. The questionnaire-style readme template (see Supplementary Information) saves time to locate necessary details to repair broken tests. The extent and quality of documentation is independently approved by a pull-request reviewer before the test is merged into the main repository. The benchmarking framework is configured such that documentation needs to be written once and is then directly embedded into the results page. Thus, the documentation is accessible both in the code and on the web interface while eliminating text duplication that could lead to discrepancies and confusion.

### Goal 5—distribution: additions and usage of tests by our community requires broad distribution

Earlier scientific tests also deteriorated due to poor communication as to the existence of these tests, which resulted in a small pool of maintainers. Because our new scientific tests are integrated into our test server framework which most of our community uses and monitors, developers are immediately aware of the tests that exist and their pass/fail status. In conjunction with regular announcements to our community, this visibility should significantly broaden the number of people able and willing to sustain the scientific tests for a long time. If we nevertheless find that our new tests deteriorate, we will host a hackathon (eXtreme Rosetta Workshop^4^) to supplement or repair these tests in a concentrated effort.

### Goal 6—maintenance: test failures are handled by a defined procedure

The often overlooked, *real* work in software development is not necessarily the development of the software itself, but its maintenance. We have a system in place outlining how test failures are handled and by whom. Each test has at least one dedicated maintainer (aka ‘observer’, usually the test author) who is notified of the test breakage via email and whose responsibility it is to repair the test. Test failures can be three-fold: technical failures, stochastic failures, or scientific failures. Technical failures (such as compiler errors, script failures due to new versions of programs, etc.) typically require small adjustments and fall under the responsibility of the test author and our dedicated test engineer.

Stochastic failures are an uncommon feature in software testing and are a rare but possible occurrence in this framework. Rosetta often uses Metropolis Monte Carlo algorithms and thus has an element of randomness present in most protocols. Setting specific seeds is done for integration tests in Rosetta (which are technically regression tests, discussed in the Supplementary Information of a previous publication^4^), which are not discussed here in detail. We refrain from setting random seeds in our scientific tests because the goal is to check whether the overall statistical and scientific interpretations hold after running the same protocol twice, irrespective of the initial seed. Further, a change in the vast Rosetta codebase that adds or removes a random number generator call is expected to cause trajectory changes even with set random seeds. The scientific tests are scaled so that individual trajectories are treated statistically and the lack of response to both seed changes and minor code changes is a feature and goal of the test. Moreover, due to the reasons above, rare stochastic failures are not a concern in our case and point to a sensibly chosen cutoff value (Supplementary Note 5). Scientific tests are interpreted in a Boolean pass/fail fashion but generally have an underlying statistical interpretation and are sampling from a distribution against a chosen target value. The statistical interpretation often varies from test to test and depends on the output of the protocol, the types of quality metrics, and sample sizes; therefore, we cannot provide specific suggestions as to which statistical measures should be used in general. Details about which statistics are used in which protocol are provided in the Supplementary Information and the linked tests. The randomness of Monte Carlo will occasionally cause a stochastic test failure because those runs happen to produce poor predictions by the tested metric. This is handled by simply rerunning the test: rare “stochastic” failures are either not stochastic—i.e., the test is signaling breakage—or are a symptom that the structure or pass/fail criteria of the test are not working as intended.

A scientific failure requires more in-depth troubleshooting and falls under the responsibility of the maintainer. If the maintainer does not fix the test, we have a rank-order of responsibilities to enforce the test repair. The principal investigator of the test designates someone in their lab. If the necessary expertise does not exist in the lab at the time (usually because people have moved on in their career), repairing the test becomes the responsibility of the person who broke it. If this developer lacks the expertise, the repair becomes community responsibility, which typically falls onto one of our senior developers.

### Most major Rosetta protocols are now implemented as scientific benchmarks

Using the framework described above, our community implemented 40 scientific benchmarks spanning a broad range of applications including antibody modeling, docking, loop modeling, incorporation of NMR data, ligand docking, protein design, flexible peptide docking, membrane protein modeling, etc. (Table 1 and Supplementary Note 6). Each benchmark is unique in its selection of targets in the benchmark set, the specific protocol that is run, the quality metrics that are evaluated, and the analysis to define the pass/fail criterion. The details for all the benchmarks are provided in the comprehensive supplement to this paper. We further publish the benchmarks with results and protocol captures on our website (https://graylab.jhu.edu/download/rosetta-scientific-tests/) twice per year for our users to see, download, run, and compare their own methods against. This transparency is crucial for the representation of realistic performance and to enhance the scientific reproducibility of our tools.

**Table 1:** Scientific tests for bio-macromolecular modeling, continuously running on our testing server framework.

Test suite	Tests	Refs.	Test author	Quality measures	Targets	nstruct	Runtime in CPUh
Antibodies	antibody_grafting	^55^	Jeliazko Jeliazkov	Fraction residues within rmsd to native	48	1	3
	antibody_h3_modeling	^56^		Score vs. rmsd	6	500	3000
	antibody_snugdock	^57^		I_sc vs. I_rmsd	6	500	3000
Carbohydrates	glycan_dock, (dock_glycans)^*^	^58,59^	Jason Labonte, Morgan Nance	I_sc vs. L_rmsd	6	1000	1100
	glycan_structure_prediction	^60^	Jared Adolf-Bryfogle	Score vs. rmsd	4	500	950
Comparative modeling	RosettaCM	^61^	Jason Fell	GDT-MM	16	200	1800
Design	ddg_alanine_scan	^62^	Ajasja Ljubetič	R, MAE, fraction correctly classified	19: 381	1	3
Design	SEWING	^63^	Frank Teets	MotifScorer, InterModelMotifScorer	1	100	75
Design	enzyme_design	^64^	Rocco Moretti	Various sequence recoveries	50	1	50
Design	design_fast	^65^	Jack Maguire, Chris Bahl	Score vs. seqrec	48	100	2600
Design, interfaces	cofactor_binding_sites	^66^	Amanda Loshbaugh	rank top, position profile similarity	7	200	170
design, immune system	mhc_epitope_energy	^67^	Brahm Yachnin	Degree of de-immunization, among others	50	100	2000
docking	protein_protein_docking	^68^	Shourya SR Burman	I_sc vs. I_rmsd	10	5000	833
	ensemble docking	^69^	Ameya Hamalkar	I_sc vs I_rmsd	3	5000	3000
FlexPepDock	FlexPepDock	^70^	Ziv Ben-Aharon	reweighted I_sc vs backbone I_rmsd	2	200	70
fragments	fragment_picking	^71^	Justyna Krys, Dominik Gront	rmsd	10	400	2000
fragments	make fragments pipeline	^71^	Daniel Farrell	Coverage, precision	65	1	3000
ligand docking	ligand_docking	^50^	Shannon Smith	Delta_Isc vs. ligand_rmsd	50	200	2000
	ligand_scoring_ranking	^50^		Spearman and Pearson correlation coefficient	57: 285	1	2
loop modeling	loop_modeling_CCD	^72^	Phuong Tran, Shane Ó Conchúir	Score vs. loop_rmsd	7	500	500
	loop_modeling_KIC	^73^		Score vs. loop_rmsd	7	500	620
	loop_modeling_KIC_fragments	^74^		Score vs. loop_rmsd	7	500	760
	loop_modeling_NGK	^75^		Score vs. loop_rmsd	7	500	570
membrane protein-energy function	mp_f19_energy_landscape^#^	^37^	Rituparna Samanta, Rebecca Alford	ddG, depth and title angle	4	1	10
	mp_f19_decoy_discrimination	^37^		Score vs. rmsd, Wrms	4×100	1	2000
	mp_f19_sequence_recovery	^37^		sequence recovery, Kullback-Leibler divergence	130	1	500
	mp_f19_ddG_of_mutation	^76^		Pearson correlation coefficient	3	1	1
membrane proteins	mp_dock	^77^	Julia Koehler Leman, Rebecca Alford	I_sc vs. I_rmsd	10	1000	200
	mp_domain_assembly	^78^		Score vs. rmsd	5	5000	700
	mp_lipid_acc	^79^		Accuracy	223	1	2
	mp_relax	^77^		Score vs. rmsd	4	100	40
	mp_symdock	^77^		I_sc vs. rmsd	5	1000	140
PDB diagnostic	PDB_diagnostic	NA	Steven Lewis, William Hansen, Sergey Lyskov	Read-in error type	entire PDB	1	1000
peptide structure prediction	simple_cycpep_predict	^48^	Vikram K. Mulligan	Score vs. rmsd, PNear	1	~800,000	320
	peptide_pnear_vs_ic50	^51^		IC50 vs. folding energy	7	80,000	400
refinement	relax_cartesian	^32^	Julia Koehler Leman	Score vs. rmsd	12	100	120
	relax_fast	^80^		Score vs. rmsd	12	100	120
	relax_fast_5iter	^80^		Score vs. rmsd	12	100	120
RNA	rna_denovo_favorites	^81^	Andy Watkins	Score vs. rmsd	12	200	120
	stepwise_rna_favorites	^82^		Score vs. rmsd	12	200	240
RosettaNMR	abinitio_RosettaNMR_rdc	^83^	Georg Kuenze, Julia Koehler Leman	Score vs. rmsd	3	2000	170
	abinitio_RosettaNMR_pcs	^83^		Score vs. rmsd	3	2000	1400

The number of tests is constantly being expanded. The test suite is the overall application, the test is the specific test, implemented by the test author(s). The quality measures are evaluated to choose a pass/fail criterion. The targets are the number of different proteins (or biomolecules) tested on, nstruct is the number of models built for each target, and the runtime in CPU hours is the total runtime over all targets.

^*^The dock_glycans test has been superceded by glycan_dock.

^#^The mp_f19_energy_landscape test has been renamed to mp_f19_tilt_angle.

### Standardizing workflows highlights heterogeneity in score function implementations

Standardizing the workflows and creating this framework provides us with the possibility of running some protocols with different score functions. Rosetta has been developed over the past 25 years and the score function has been constantly improved over this timeframe. Details of this evolution and the latest standard score function REF2015 can be found in references^35,36^. The attempt to easily switch score functions for an application reveals a major challenge: many applications employ the global default score function differently, a problem exacerbated by the various user interfaces to the code (see Supplementary Note 7). The heterogeneity in implementations makes it impossible to easily test different score functions for all of the applications and reveals that it hinders both progress and unification of the score functions, possibly into a single one.

### Use case (1): test framework allows comparison of score functions for multiple protocols

**Fig. 3:**
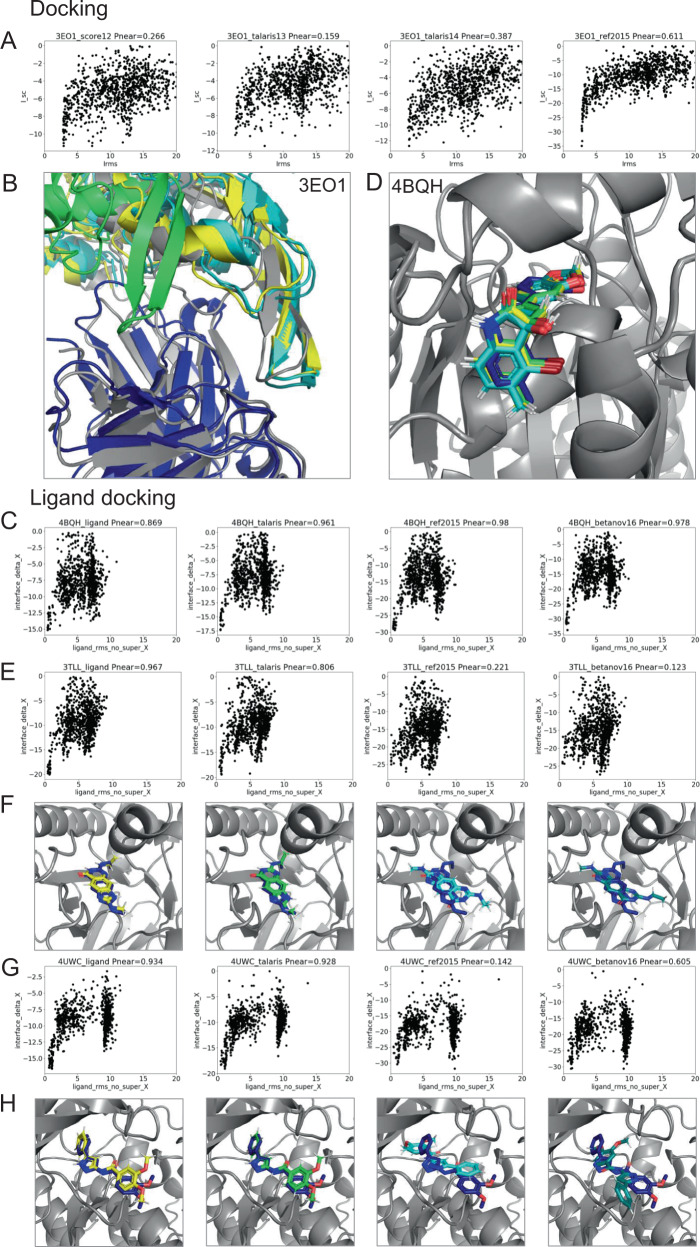
Score function comparison for specific proteins for protein–protein docking and ligand docking. Comparison of different score functions for different applications using the *P*_Near_ metric as an indication of “funnel quality”. *P*_Near_ falls between 0 (no funnel or incorrect global minimum) and 1 (the perfect funnel). The lambda parameter indicates the spread on the *x*-axis and is set to 4.0. Score functions are sorted from oldest to newest (left to right) and the models are colored in gray as the native (PDB) structure, then according to the score functions in order: yellow, green, cyan, and teal. **A**, **B** comparison for protein-protein docking on target with PDB ID 3eo1. The starting model is shown in dark blue—the docking partner of the starting model is too far away to be shown in the picture. The quality of the prediction improves over different score functions as indicated by tightening of the energy funnel. **C**, **D** comparison for ligand docking on target 4bqh. The native ligand pose is shown in dark blue. The quality of the prediction improves over different score functions as indicated by tightening of the energy funnel. **E**–**H** Ligand docking comparison on targets 3tll and 4uwc, respectively. Newer score functions lower the energy of an incorrect, alternative docking conformation, leading to a worse prediction.

**Fig. 4:**
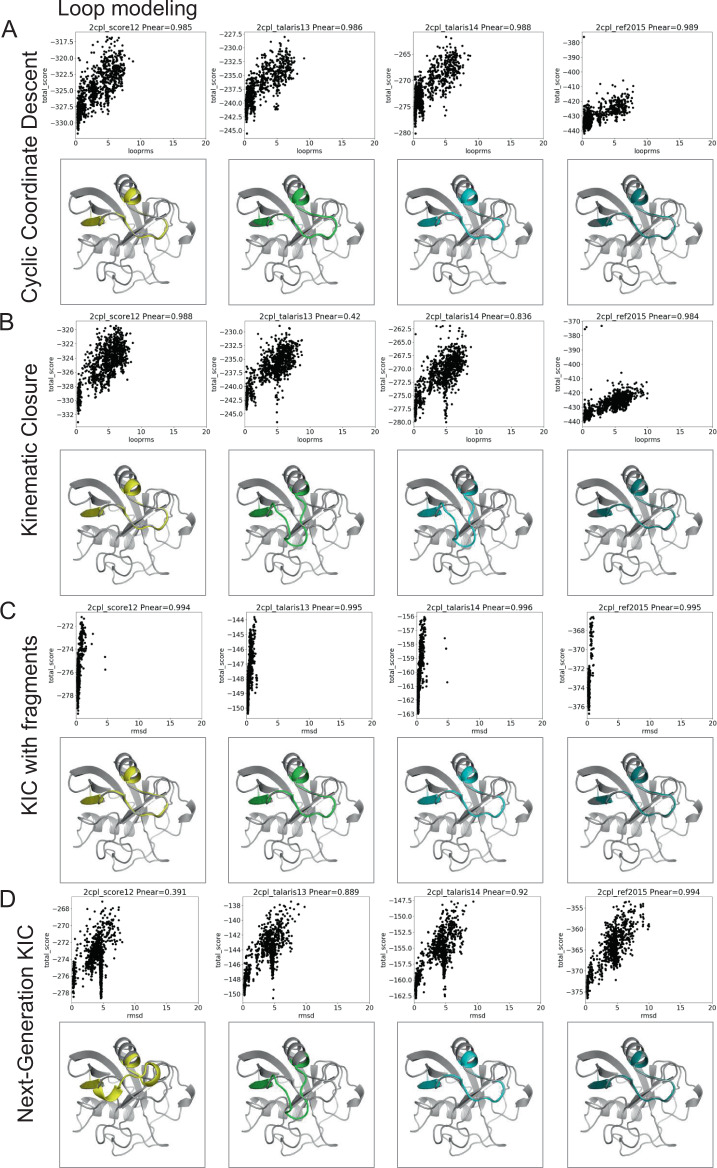
Score function comparison for one protein and different loop modeling protocols. The protocols are **A** cyclic coordinate descent—CCD, **B** kinematic closure—KIC, **C** KIC with fragments, and **D** next-generation KIC—NGK. Score functions are sorted from oldest to newest (left to right) and the models are colored in gray as the native (PDB) structure, then according to the score functions in order: yellow—score12, green—talaris13, cyan—talaris14, and teal—ref2015. This figure shows a particularly interesting example, which is not necessarily representative of other targets. Interesting for this target are the differences in the energy landscapes that are sampled and the presence of a second, incorrect conformation with low energy for some protocols and some score functions, but not others. For 3 out of 7 targets in our comparison, including this one, most conformations that KIC (kinematic closure) with fragments samples, are close to the native structure. Again, for larger benchmarks, this is likely not as often the case.

**Fig. 5:**
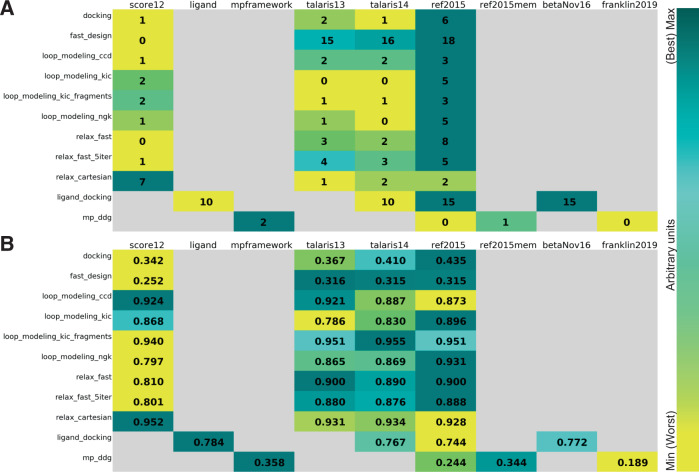
Summary of score function comparisons. Comparison of different score functions (one per column) for different applications and protocols, using the *P*_Near_ metric as an indication of “funnel quality”. *P*_Near_ falls between 0 (no funnel or incorrect global minimum) and 1 (the perfect funnel). The lambda parameter indicates the spread on the *x*-axis and is set to 4.0 in our comparison. Cells are colored according to the color bar on the right, teal is better. Unavailable data is indicated in gray. **A** The panel uses a “winner-takes-all” comparison: for each protein, the score function with the highest (i.e., best) *P*_Near_ value (see methods) gets a point. Points are then summed by column, identifying the score function with the most and highest *P*_Near_ values across proteins, the higher the better. **B** The averages of the *P*_Near_ values for each score function were used, i.e., computed over each column. Higher values are better.

Using our framework allows us to directly compare runs with different variables. For instance, we can compare different score functions for various applications: protein–protein docking, high-resolution refinement, loop modeling, design, ligand docking, and membrane protein ddG’s (Table 2 and Figs. 3–5). We test the latest four score functions: score12, talaris2013, talaris2014, and REF2015 for all but ligand docking and membrane protein ddG’s. Ligand docking has a special score function and requires adjustments—we test the ligand score function, talaris2014, REF2015, and the experimental score function betaNov2016. Membrane protein ddG’s are tested on the membrane score functions mpframework2012, REF2015_mem, franklin2019, and the non-membrane score function REF2015 as a control.

**Table 2:** Tests for which we compare different score functions (score12, talaris2013, talaris2014, ref2015, ligand, betaNov16, mpframework, ref2015mem, and franklin2019), complete with quality measures, number of targets in each benchmark, number of models created (nstruct) and runtime in CPU hours per score function.

Test suite	Tests	score12	ligand	mpframework	talaris13	talaris14	ref2015	ref2015mem	betaNov16	franklin2019	Quality measures	Targets	nstruct	Runtime in CPUh
Docking	docking	x			x	x	x				I_sc vs. I_rmsd	10	1000	150
Design	design_fast	x			x	x	x				Score vs. seqrec	48	100	2600
Loop modeling	loop_modeling_CCD	x			x	x	x				Score vs. loop_rmsd	7	500	500
	loop_modeling_KIC	x			x	x	x				Score vs. loop_rmsd	7	500	620
	loop_modeling_KIC_fragments	x			x	x	x				Score vs. loop_rmsd	7	500	760
	loop_modeling_NGK	x			x	x	x				Score vs. loop_rmsd	7	500	570
Refinement	relax_fast	x			x	x	x				Score vs. rmsd	12	100	120
	relax_fast5	x			x	x	x				Score vs. rmsd	12	100	120
	relax_cart	x			x	x	x				Score vs. rmsd	12	100	120
Ligand docking	ligand_docking		x			x	x		x		Delta_Isc vs. ligand_rmsd	50	200	2000
Membrane proteins	mp_ddg (ddG of mutation)			x			x	x		x	Pearson correlation	3	50	1800

The ligand docking and membrane ddG applications require specialized score functions.

The benchmark sets and quality metrics are described in Table 2 and in detail in the Supplementary Information. To compare the score functions, we plot each application’s quality metrics (for instance interface score vs. interface RMSD for protein-protein docking, total score vs. loop RMSD for loop modeling). We then evaluate the “funnel quality” by computing the *P*_Near_ metric, which falls between 0 and 1, with higher values indicating higher quality^48,49^. For the protein design test, we compute the average sequence similarity of the 10 lowest-scoring (best) models instead of *P*_Near_ and for the membrane ddG test, we use the Pearson correlation coefficient between experimental and predicted ddG’s. We further summarize the quality metrics per protocol and score function by a “winner-takes-it-all” comparison (Fig. 5A) and by an average metric overall target per application per score function (Fig. 5B).

A few main observations follow from this comparison: at first glance, in this comparison, REF2015 performs generally better overall, yet the best score function to use depends on the application—even different types of protocols can impact prediction accuracy. However, it should be noted that some tests have a small sample size due to the required computational resources, therefore impacting the statistical significance of these outcomes. Second, more recent score functions are not automatically better for any given application, likely because performance depends on how the score function was developed and tested. For a more detailed discussion, see the Supplementary Information. Third, results differ in some cases depending on how the data were summarized; the top-performing score functions per application from the “winner-takes-it-all” comparison are not necessarily the top performers when the average of the *P*_Near_ value is used, as can be seen in ligand docking (Fig. 5B—reference^50^ discussed this in-depth).

### Use case (2): scientific test framework facilitates bug fixes and maintenance

The scientific test framework is also useful for code maintenance, to ensure that the correction of bugs does not invalidate the scientific performance of the application. This can be achieved by comparing the scientific performance of a run before and after fixing a bug in the code. For example, in October 2019, we identified an integer division error in one of our core libraries: the fraction 2/3 was incorrectly assumed to evaluate to 0.6666…, when in fact integer division discards remainders, yielding 0. This calculation affected the computation of hydrogen bonding energies and their derivatives and correcting it resulted in a small but perceptible change in some of the hydrogen bond energies. This led to the need to balance between fixing the bug and managing the complex interdependencies or to preserve the existing scoring behavior since the rest of the score function had been calibrated with the bug present. By running the scientific tests on a development branch in which we had fixed the bug, we confirmed that although the correction results in a small change in the energies, it had no perceptible effect on the scientific accuracy of large-scale sampling runs for structure prediction, docking, design, and any other protocol tested. This allowed us to make the correction without harming Rosetta’s scientific performance. We are certain that the scientific tests will be invaluable for ensuring that future bug-fixing and refactoring efforts do not hinder the scientific performance of our software, thus illustrating a key example of scientific benchmarks informing substantive decisions developers must make as they navigate code life cycles.

### Use case (3): test framework allows detailed investigation of new score functions under development

Our framework can also be used to test how major code improvements would affect scientific performance before they are adopted as default options in the code. As an example, we can test how newly developed score functions perform: although small molecules and proteins are generally more rigid structures, intermediate-scale molecules are frequently disordered and flexible. A recent study shows that Rosetta’s estimates of rigidity (using the funnel quality metric *P*_Near_ computed to a designed binding conformation) for peptides designed to bind to and inhibit a target of therapeutic interest correlate well with IC_50_ values^51^. Since this prediction has relevance to computer-aided drug development efforts, we want to ensure that future protocol development would not impair these predictions. We created a test (called *peptide_pnear_vs_ic50*) that performs rigidity analysis on a pool of peptides that were previously characterized experimentally and computes the correlation coefficient for the *P*_Near_ values from predicted models to the experimentally measured IC_50_ values. We find that the current default score function, REF2015, produces much better predictions than the legacy talaris2013 and talaris2014 score functions (*R*^2^ = 0.53, 0.53, and 0.90 for talaris2013, talaris2014, and REF2015, respectively), indicating an improvement of the score function accuracy for this particular application^35^. However, this correlation is considerably worse with the score function Beta currently under development (*R*^2^ = 0.19). This reveals problems in the candidate’s next-generation score function that will have to be addressed before it becomes the default. Our scientific tests embedded in the test server framework provide a means of rapidly benchmarking and addressing these problems.

### Use case (4): this framework and tests encourage scientific reproducibility on several levels

How is this framework useful beyond the specific tests mentioned here? Its usefulness for Rosetta developers and users lies in the protocol captures, the specific performance of each protocol, and the knowledge that scientific performance is monitored over time. Developers of macromolecular modeling methods outside of Rosetta can use and run the exact test protocol captures to compare Rosetta’s results to their own, newly developed methods. The code for the general framework to run large-scale, continuous, automated tests is available under the standard Rosetta license and is useful for developers of any type of software. Lastly, the framework highlights how software can be developed in a scientifically reproducible manner, lessons of which are useful and necessary for the scientific community at large. While we recognize the time and work required to implement such tests and the underlying framework, the benefits far outweigh the effort spent in trying to reproduce results that were implemented in a manner that lacks necessary aspects for reproducibility, as discussed in Table 3.

**Table 3:** Guidelines for reproducible research and for the development of high-quality methods.

General guidelines for reproducibility	Guidelines for high-quality benchmarks
1. Document artifacts	1. Define scientific questions for the benchmark
2. Share input, output, and exact workflow in detail under an open license in public repositories	2. Define quality metrics that are practically relevant
3. Cite the data, software, and workflows	3. Diversify examples in the benchmark set to cover easy and difficult targets
4. Use persistent links in the publication	4. Separate benchmark set from the developed method
5. Journals should check for reproducibility	5. Pick cutting edge methods to compare your method to
6. Funding agencies should fund reproducibility research	6. Use benchmarked methods that are freely available

## Discussion

Here, we present a test server framework for continuously running scientific benchmarks on an integrated HPC cluster and detail the way this framework has had a positive and substantive effect on our large community of scientists. The framework itself is sufficiently general that it could in principle be used on many types of scientific software. We use it on Rosetta protocols that cover the three main interfaces to the codebase: the command line, RosettaScripts, and PyRosetta. New benchmarks are easily added and debugged, and the workflow for setting them up is well-documented and general: new tests can be added in a matter of hours and require minimal coding experience in Rosetta. We provide detailed documentation and consistency in the presentation of results, thereby facilitating maintenance by more than just experts in the community and ensuring the longevity of these tests. Automated and continuous runs of these tests allow us to recognize shifts in performance, as development is simultaneously carried out on several interdependent but otherwise unrelated fronts. Thus, we can build a longitudinal map of accuracy and scientific correctness in a constantly evolving codebase (for ourselves and our users), provide realistic protocol captures of how to run applications, and build tools that follow guidelines for improving reproducibility. Diversity in the choice of targets in the benchmark sets provides a realistic performance somewhat insulated from institutional and career incentives. So far, 40 benchmarks for various biomolecular systems and prediction tasks have been added to our server framework and more will be added over time. Due to the size of our software and the large number of protocols available, running these benchmarks requires a substantial amount of resources, which are funded through RosettaCommons, since such benchmarks are a priority for software sustainability. Even though our setup involves the integration of a custom software framework and web interface with typical HPC hardware, we expect our design choices to be of general interest and integrable with paid services such as Drone CI^44^, Travis CI^45^, or Jenkins^46^, which are great options for small software development communities or labs that lack the hardware or personnel resources. This framework demonstrates how challenges in scientific reproducibility can be approached and handled in a general manner, even in a large and diverse community.

Implementation of a modular testing system addressing the goals above is a crucial step in achieving the reproducibility of software codes. Yet, several challenges remain that are mostly due to a lack of incentive structure. (1) In the past several years, funding agencies and journals have introduced requirements for data sharing, storing, and ensuring reproducibility. However, even if data/detailed workflows and output are shared and available, grant or paper reviewers are likely not going to take the time to run the code because it often comes with a substantial time investment for which the reviewers do not get much in return. We argue that offering high-value incentives, such as co-authorship on the paper, mini-grants, or other compensation to the reviewer, in return for them running the code and comparing the data, could potentially make a huge difference in closing the gap in the reproducibility crisis. Alternatively, funding agencies and journals could require that another scientist, independent from the group publishing the method, is the independent code reviewer and becomes a co-author. (2) Both funding agencies and academic labs working on smaller software tools need to understand that the bulk of the work in developing a tool is not the development of the tool itself, but its maintenance, requiring years of sustained effort for it to thrive into something valuable and useful with actual impact on the scientific community. (3) Similarly, funding agencies and labs need to understand that *code is cheap but high-quality code is expensive* to create. The short-term nature of most academic research labor (undergraduate, graduate student, and postdoctoral researcher) conflicts with the long-term necessity of maintenance. Sustainable research tooling requires careful oversight and long-term management by a project leader, ensuring that maintenance responsibilities are continually reassigned as the labor pool shifts.

## Methods

The RosettaCommons community of developers has emphasized software testing for over 15 years. To support our community of hundreds of developers, our user base of tens of thousands of users, and the codebase of over 3 million lines of code^4^, we implemented a custom testing architecture to fit our needs. We use this platform (a.k.a. the “Benchmark Server”) to run all our tests including unit tests, integration tests, profile tests, style tests, score function tests, build tests, and others. Using this benchmark server to implement scientific tests is therefore a natural extension of its current use. Our custom testing software runs on a dedicated HPC cluster (which also runs the ROSIE server^52^), paid for by the RosettaCommons from government and non-profit funding, and commercial licensing fees.

### The backend of the benchmark infrastructure

Our testing infrastructure consists of a number of machines:

- Database server. Our data center stores information about revisions, test, and sub-test results as well as auxiliary data like comments to revisions or a list of branches that are currently tracked via GitHub^4,53^. We are using PostgreSQL.
- Web server. The web interface for Rosetta developers connects to the database server. When a developer asks for a particular revision or test results, the webserver gathers these data from the database server, generates the HTML page, and sends it to the developer who looks at the page in a web browser. The web server also allows developers to queue new tests through the submit page on the web interface.
- Revision daemon. This application watches the state of various branches, queues tests, and sends notifications. The daemon tracks the list of branches and periodically checks if a new revision for a particular branch was committed. When a new revision has been committed, it schedules the default test set for that branch. The daemon also watches for open pull requests on GitHub, and for each pull request, it checks for specific test labels (for instance “standard tests”). The revision daemon schedules any tests with that label for that pull request.

Because scientific tests require an enormous amount of computing power, we are currently unable to test every single revision in the Rosetta main branch. Instead, we run scientific tests on a best-effort basis. The tests run continuously, but because there are sometimes multiple updates to the main branch per day and it takes the scientific tests about a week to run, many revisions in the main branch remain untested. In case of a test failure, the revision daemon performs a binary search bisecting the untested revisions to determine the exact revision that is responsible for the breakage.

[4.-N.] Testing daemons. The testing daemons run on various platforms: Mac, Linux, and Windows. We currently have 18 of these daemons, some of which are meant for build tests (i.e., on Windows) and some of which are capable of running tests on our HPC cluster. Each daemon periodically checks the list of queued tests from the database server. If there is any test which that daemon is capable of running, it runs the test and then uploads the test results (logs, result files, and test results encoded in JSON) to our SQL database.

This backend code is specific to our hardware, HPC use patterns, and system administration environment, and maintained separately from the code that performs or tests science. This code does not include the frontend scientific testing framework (next paragraph) and is not needed to replicate any of the scientific results. The frontend implementation of the scientific testing framework including all the scientific benchmarks is fully available under the RosettaCommons license.

#### Setup of the scientific tests

We chose a simple setup as shown in Fig. 1C. Each scientific test requires a small number of files, available in a template directory. All files in this directory are well documented with comments, and the lines that require editing for specific tests are highlighted. Each scientific test directory starts from a template containing the following files:

- *input files*—are either located in this directory or in a parallel git submodule if the input files exceed 5 MB. This policy prevents our main code repository from becoming overly inflated with thousands of input files for scientific benchmarking.
- *0.compile.py*—compiles the Rosetta and/or PyRosetta executable.
- *1.submit.py*—submits the benchmark jobs either to the local machine *or* to the HPC cluster. Note that this “or” provides hardware non-specificity; the user writes and debugs locally and can run at scale on the benchmark server.
- *2.analyze.py*—analyzes the output data, depending on the scientific objective. Analysis functions that are specific to this particular test live in this script, while broadly useful analysis functions are located in a file that is part of the overall Python test server framework and that contains functions to evaluate quality measures.
- *3.plot.py*—plots the output data via *matplotlib*^54^, or other plotting software as appropriate.
- … – other numbered scripts can be added as needed; they will run consecutively as numbered.
- *9.finalize.py*—gathers the output data and classifies the test as passed or failed, creates an HTML page by combining the documentation from the readme file, the plots of the output data and the pass/fail criterion. The HTML page is the main results page that developers, maintainers, and observers examine.
- *citation*—includes all relevant citations that describe the protocol, the benchmark set, or the quality measures.
- *cutoffs*—contains the cutoffs used for distinguishing between a pass or a failure for this test.
- *observers*—email addresses of developers that either set up the test and/or maintain it. If a test fails on the test server, an email is sent to the observers to inform them of the test breakage.
- *readme.md*—is a questionnaire-style markdown file that contains all necessary documentation to understand the purpose and detailed methods of the test. Obtaining detailed documentation is essential for the maintenance and longevity of the test. The goal is that anyone with basic Rosetta expertise and training can understand, reproduce, and maintain the test. The template readme file is provided in the Supplementary Information of this paper.

Most Rosetta protocols use the Monte-Carlo sampling protocol to create protein or biomolecule conformations, which are then evaluated by a score function.

### Reporting summary

Further information on research design is available in the Nature Research Reporting Summary linked to this article.

## Supplementary information

Supplementary Information

Peer Review File

Reporting Summary

## Data Availability

All Rosetta code and the frontend implementation of the scientific testing framework including all the scientific benchmarks are fully available under the RosettaCommons license. In addition, complete protocol captures for all benchmarks with input files, command lines, output files, analyses, and result summaries are publicly available to view and download at https://graylab.jhu.edu/download/rosetta-scientific-tests/. These complete protocol captures are available in two code revisions and will be automatically expanded with new revisions added about every 6 months. Older revisions remain on the server. Details about each of the 42 datasets with accession codes etc. are provided under the link above.

## Code availability

Rosetta is licensed and distributed through https://www.rosettacommons.org. Licenses for academic, non-profit, and government laboratories are free of charge; there is a license fee for industry users. A license is required to gain access to the Github repository. Specific version numbers are given in the Supplementary Information.
