# Supplementary material for: Ensuring scientific reproducibility in bio-macromolecular modeling via extensive, automated benchmarks
Source: Nat Commun. 2021 Nov 29;12:6947. doi: 10.1038/s41467-021-27222-7 (PMC8630030; doi:10.1038/s41467-021-27222-7)
Supplement: Supplementary file 1 — Supplementary Information [file 41467_2021_27222_MOESM1_ESM.pdf]

Supplement for:

Ensuring scientific reproducibility in bio-macromolecular modeling via extensive, automated benchmarks

Koehler Leman, Julia; Lyskov, Sergey; Lewis, Steven; Adolf-Bryfogle, Jared; Alford, Rebecca F.; Barlow, Kyle; Ben-Aharon, Ziv; Farrell, Daniel; Fell, Jason; Hansen, William A.; Harmalkar, Ameya; Jeliazkov, Jeliazko; Kuenze, Georg; Krys, Justyna D.; Ljubetič, Ajasja; Loshbaugh, Amanda L.; Maguire, Jack; Moretti, Rocco; Mulligan, Vikram Khipple; Nance, Morgan L.; Nguyen, Phuong T.; Ó Conchúir, Shane; Roy Burman, Shourya S.; Samanta, Rituparna; Smith, Shannon T.; Teets, Frank; Tiemann, Johanna KS; Watkins, Andrew; Woods, Hope; Yachnin, Brahm J.; Bahl, Christopher D.; Bailey-Kellogg, Chris; Baker, David; Das, Rhiju; DiMaio, Frank; Khare, Sagar D.; Kortemme, Tanja; Labonte, Jason W.; Lindorff-Larsen, Kresten; Meiler, Jens; Schief, William; Schueler-Furman, Ora; Siegel, Justin; Stein, Amelie; Yarov-Yarovoy, Vladimir; Kuhlman, Brian; Leaver-Fay, Andrew; Gront, Dominik; Gray, Jeffrey J.; Bonneau, Richard

Supplementary Note (1): Testing tools and possible setups for small software development groups

The Rosetta community is fortunate to have its own hardware and personnel resources; such resources are often not readily available for small software development groups. Below are a few recommendations of how a (similar) testing setup can be achieved with public or paid resources. The mentioned resources should serve as inspiration or starting points and we recommend checking on pricing and setup requirements, as these can change over time.

Software is often tested via Continuous Integration (CI) tests, which require software and hardware resources. Several of the automation software packages are integrated with hardware on the backend but can also be run on locally available hardware.

Automation packages:

- Github Actions[1] is integrated with Github[2] and a hardware backend. There is detailed documentation available, and one can run tests from public repositories for free. Code in private repositories can be run on a pay-per-minute basis. Setup can be achieved by a technically interested graduate student without a computer science background.
- Drone CI[3] is easy to set up and is used by several well-known companies.
- Travis CI[4] is a more mature tool. The plan is pay-per-credit and one might be able to get free compute resources for a private Github repository under an academic pro Github plan.
- Jenkins[5] is an older tool with more complexity but might have more configuration options available. It can be run on any hardware, for instance on Amazon Web Services[6] (AWS) through the Terraform infrastructure builder.

Hardware resources:

- Many academic labs developing software already have hardware available for compiling and running their code. Several of the automation packages mentioned above can be configured to run on this hardware.
- There are many paid cloud server options like Amazon Web Services[6] (AWS), Heroku[7], and Google Cloud Services[8].
- Academic options like XSEDE[9] funded by the US National Science Foundation or university-scale compute clusters are available to many laboratories.

Supplementary Note (2): Basis for the specific goals for the scientific test server framework

The Rosetta community had implemented scientific tests over 10 years ago which deteriorated over time. The goals outlined in the main paper are a result of critically evaluating reasons for their deterioration, combined with long-term knowledge of our community and the organization of our gigantic codebase. Below we state the goals and our reasoning behind these goals.

(1) Simplicity of the framework to encourage maintenance and support – we know from experience that more complex software design leads to higher time and labor investments in maintaining it. Simpler software design leads to more efficient support. This is critically important as we have learned from the growth over the Rosetta software suite which now has over 3.1 million lines of code.

(2) Generalization to support all user interfaces to the Rosetta codebase (command line, RosettaScripts[10], PyRosetta[11,12]) – setting up a framework for each interface to the codebase would increase complexity and therefore maintenance and support. We wanted to support all interfaces with a single framework to make it easy on implementers and maintainers.

(3) Automation to continuously run the tests on an HPC cluster with little manual intervention – we noticed that in a huge codebase such as Rosetta, any type of automation helps tremendously in maintenance. Our community has very few people truly supporting and maintaining the software, so increasing automation and decreasing the requirement for manual intervention is necessary and useful. Additionally, a requirement for manually running these tests in a defined frequency increases the chances of them being neglected, this being the first step in deterioration.

(4) Documentation on how to add tests and scientific details of each test to allow maintenance by anyone with a general science or Rosetta background – the previous scientific tests were barely documented and therefore were very hard to maintain. The requirement to improve long-term support is proper and detailed documentation.

(5) Distribution of the tests to both the Rosetta community and their users and publicizing their existence to encourage addition of new tests and maintenance by the community. The previous scientific tests were poorly publicized, which, in addition to lack of documentation, made them almost impossible to maintain. The new tests are automatically distributed to users and developers of Rosetta and their presence on the test server website, that developers monitor frequently, improves knowledge of these tests and increases willingness to maintain them.

(6) Maintenance of the tests, facilitated by each of the previous points. Scientific tests are only useful if they're continuously run, monitored and maintained. All of the above points are a requirement to improve maintenance and support.

Supplementary Note (3): Notes on our test server framework

We use our test server architecture to run unit, integration, build, and performance tests. The integration between hardware and software in the test server allows anyone in our community to schedule any tests for any Rosetta development branches (their own, or other community members') that have been pushed to the version control server GitHub[2]. The test server web interface (FIG. 2A; available at https://benchmark.graylab.jhu.edu/) is integrated with GitHub and its pull-request feature allows scheduling of tests via branch name, pull-request identifier, or GitHub commit *sha1* key. Dedicated maintainers ('observers') are notified of test breakage via email and community members can view all test results on the test server web interface after GitHub login. The HPC cluster that runs these computations is partly funded through government grants and funds from the RosettaCommons[13].

Supplementary Note (4): Steps to add a scientific test to the Rosetta test server framework for continuous, automated testing

Once the dataset, interface (command line, RosettaScripts, or PyRosetta), specific command line, and quality measures have been chosen, the author can use the template and follow the steps outlined on the documentation page[14] to contribute the test.

The test should be created in a feature branch, pushed to GitHub and a pull-request should be created for review. Once the test runs without errors locally, it can be run on the test server. Having individually numbered scripts from compilation to finalizing the results page facilitates debugging. Once the test runs without errors locally and on the test server and reviewers approved, the feature branch is merged into Rosetta's main development branch. The test will then run 'continuously' from the 'oldest' (i.e., the test that was run longest ago) to the newest, based on node availability, and the results are stored in the database.

Supplementary Note (5): Documentation: Defining appropriate cutoffs for pass/fail is crucial for longevity

Scientific tests check the scientific performance of an entire protocol or workflow. Since Rosetta samples via a Monte-Carlo procedure, the randomness from the sampling will affect the results, making it more difficult to generalize a pass/fail criterion. Other difficulties are the varying scientific objectives of different tests and the question of how to set the cutoff for the test to be most useful and maintainable. For instance, if the cutoffs are such that the test almost never passes, the test will be ignored as fundamentally miswritten and becomes essentially meaningless. The goal is to find an optimal cutoff that probes scientific validity while failing only when the latter is compromised. We determine the cutoffs by defining specific quality measures, running each protocol several times, and adjusting the cutoffs such that the tests generally pass, but fail when the output shifts too far from its original position. We find that tests that define a cutoff by adding a small number of standard deviations to the mean of the results are generally useful.

Supplementary Note (6): Benchmarks sets are diverse and provide a more realistic performance

One of the problems with publishing newly developed or improved methods is the pressure to promote the method as best as possible to warrant publication and use by the scientific community. This is often at odds with presenting the method in a realistic light. The methods'

performance is often artificially inflated through cherry picking targets and removing outliers, or its improvement is marginal and not statistically relevant. Overly positive presentations pertain to the collection of targets in the benchmark dataset, the choice of quality measures and the appropriate use of statistics. To produce a realistic performance of the method, we ask our authors to collect benchmark sets that prevent biases in the presentation of results and to choose examples that are diverse in prediction difficulty and other independent dimensions or features. Explicitly stating shortcomings and areas of improvement for the benchmark set, method, or quality measures directs new developers towards areas of improvements for future method development tasks.

Supplementary Note (7): Score function implementations suffer from heterogeneity
One possible area of improvement that the scientific benchmarks revealed is the different use and implementations of score functions across applications. The heterogeneity in score function implementation makes it impossible to easily test different score functions for all applications and hinders progress in development. Some applications use and implement hardcoded score functions, some use the one provided in the command line, some use the ones provided in the RosettaScripts interface. Some applications implement different score functions for low- and high-resolution modes, sometimes employing different ones for sampling and scoring. Some applications use special score functions, specifically designed for a particular application and improved over the years, sometimes for different stages of the protocol or with and without specific constraints or features turned on or off.

Supplementary Note (8): Documentation template in markdown format

AUTHOR AND DATE
Who set up the benchmark? Please add name, email, PI, month and year

PURPOSE OF THE TEST
What does the benchmark test and why?

BENCHMARK DATASET
How many proteins are in the set?
What dataset are you using? Is it published? If yes, please add a citation.
What are the input files? How were they created?

PROTOCOL
State and briefly describe the protocol.
Is there a publication that describes the protocol?
How many CPU hours does this benchmark take approximately?

PERFORMANCE METRICS
What are the performance metrics used and why were they chosen?
How do you define a pass/fail for this test?
How were any cutoffs defined?

KEY RESULTS
What is the baseline to compare things to - experimental data or a previous Rosetta protocol?
Describe outliers in the dataset.

DEFINITIONS AND COMMENTS
State anything you think is important for someone else to replicate your results.

LIMITATIONS
What are the limitations of the benchmark? Consider dataset, quality measures, protocol etc.
How could the benchmark be improved?
What goals should be hit to make this a "good" benchmark?

Supplementary Note (9): Examples of the reports associated with the scientific tests conducted in this manuscript are included below

To maintain fidelity to the original reports in which figures are not accompanied by a legend, the figures in these reports are not referred to as Supplementary Figures. For an updated list of the scientific tests and the latest run data, please visit:

https://graylab.jhu.edu/download/rosetta-scientific-tests/

Scientific test: abinitio_RosettaNMR_pcs

FAILURES

None

RESULTS

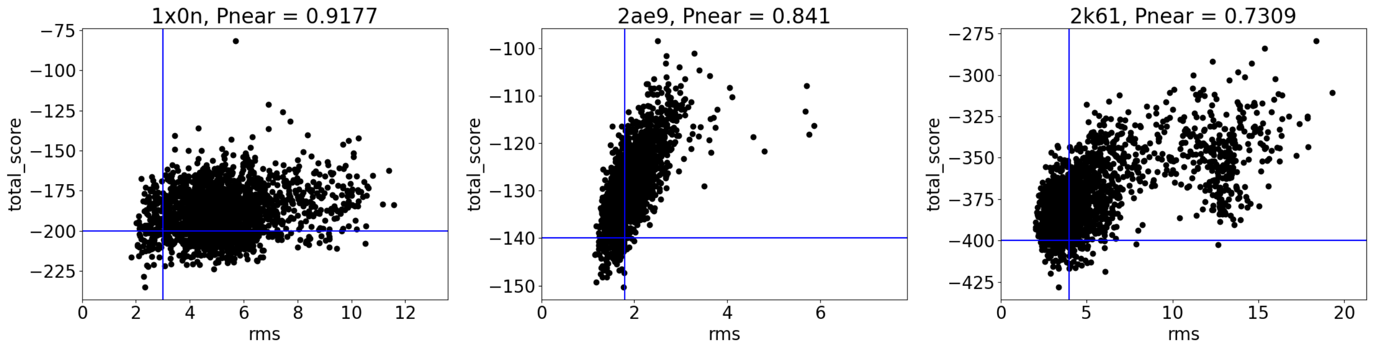

AUTHOR AND DATE

The benchmark was originally created by Georg Kuenze (georg.kuenze@gmail.com), published in 2019, former Meiler lab, now at Leipzig University. It was implemented on the test server by Julia Koehler Leman (julia.koehler.leman@gmail.com) in the Bonneau lab, in July 2021.

PURPOSE OF THE TEST

The benchmark ensures that the score-vs-rmsd distribution of ab initio models created with NMR data (RDC / PCS specifically) don't shift too much from the original distribution.

BENCHMARK DATASET

The benchmark set contains 3 proteins of various sizes:

2ae9 - alpha protein, 76 residues, 86 PCS constraints

1x0n - alpha / beta protein, 104 residues, 227 PCS constraints

2k61 - alpha / beta protein, 146 residues, 403 PCS constraints

The benchmark set is described in detail in (Kuenze, Structure, 2019). The protocol runs ab initio structure prediction with NMR constraints, so input files are essentially a fasta sequence and constraint files. All input files are located in the scientific data submodule with the folder of the same name. Input files were originally taken from the protocol capture. Input files are the following:

.fasta - containing the sequence

.pdb - as a native for RMSD comparison

fragments (3mers / 9mers) - created with chemical shift information from TALOS - these are the .tab files in the scientific data submodule

.wts_patch - these are patch files containing the weight of the the PCS score against the rest of the Rosetta scorefunction terms, see below for how they are determined

.tbp - topology broker file for ab initio structure prediction

.rdc.inp - RosettaNMR constraint files containing the mathematical details of the PCS constraint setup, for instance the spinlabel position. This file contains the .dat file names that contain the actual measured PCS.

PROTOCOL

In a nutshell, the overall protocol comprises of the following steps:

1) use chemical shift information to run TALOS for the prediction of secondary structure

2) use secondary structure prediction from TALOS files for fragment picking

3) run ab initio structure prediction WITHOUT NMR constraints to get a baseline of the score distribution

4) rescore these decoys with NMR constraint data to get score distribution WITH NMR data

5) from both score distributions of the models WITH and WITHOUT NMR data, compute the optimal weight of the NMR score term

6) run ab initio structure prediction WITH NMR constraints with the optimized weight

Note that the protocol on the test server only runs the last step (step 6) and both fragment picking and constraint weight optimization has been done beforehand.

Runtimes are about 1400 CPU hours for this test: (800s per model per target on avg) x (3 targets) x (2000 decoys)

PERFORMANCE METRICS

Output files of structure prediction are a score file and a binary silent file. We look at the score file and plot the score-vs-rmsd distribution of the created models. Passes are defined by the cutoffs for all to be true: 10% of the models below the RMSD cutoff, 10% of the scores below the score cutoff and PNear higher than the PNear cutoff, defined by the first run minus 0.1. All cutoffs were defined by the first run of the protocols and adjusted over several runs.

KEY RESULTS

We compare the results against the benchmarks described in (Kuenze, Structure, 2019). The funnel of 1x0n isn't quite as well-defined as in the paper and from the first, single run it is unclear why.

DEFINITIONS AND COMMENTS

LIMITATIONS

The run times are on the higher end, which is why we're only testing 3 proteins on the test server. Ideally, it would be nice to run all benchmarks from the paper on the test server, but this is computationally prohibitive. Target diversity, size and complexity of the full benchmark are well-chosen and optimized.

REVISION

revision: 61711

test_id: 713284

Scientific test: abinitio_RosettaNMR_rdc

FAILURES

None

RESULTS

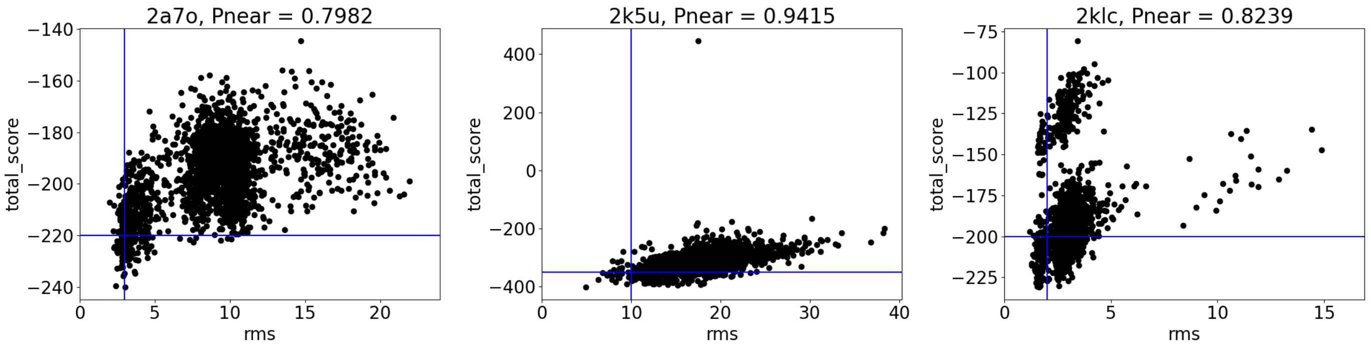

AUTHOR AND DATE

The benchmark was originally created by Georg Kuenze (georg.kuenze@gmail.com), published in 2019, former Meiler lab, now at Leipzig University. It was implemented on the test server by Julia Koehler Leman (julia.koehler.leman@gmail.com) in the Bonneau lab, in July 2021.

PURPOSE OF THE TEST

The benchmark ensures that the score-vs-rmsd distribution of ab initio models created with NMR data (RDC / PCS specifically) don't shift too much from the original distribution.

BENCHMARK DATASET

The benchmark set contains 3 proteins of various sizes:

2klc - alpha / beta protein, 101 residues, 75 RDC constraints

2a7o - alpha protein, 112 residues, 120 RDC constraints

2k5u - alpha / beta protein, 181 residues, 322 RDC constraints

The benchmark set is described in detail in (Kuenze, Structure, 2019). The protocol runs ab initio structure prediction with NMR constraints, so input files are essentially a fasta sequence and constraint files. All input files are located in the scientific data submodule with the folder of the same name. Input files were originally taken from the protocol capture. Input files are the following:

.fasta - containing the sequence

.pdb - as a native for RMSD comparison

fragments (3mers / 9mers) - created with chemical shift information from TALOS - these are the .tab files in the scientific data submodule

.wts_patch - these are patch files containing the weight of the the RDC score against the rest of the Rosetta scorefunction terms, see below for how they are determined

.tbp - topology broker file for ab initio structure prediction

.rdc.inp - RosettaNMR constraint files containing the mathematical details of the RDC constraint setup, for instance the alignment tensor. This file contains the .dat file names that contain the actual measured RDCs.

PROTOCOL

In a nutshell, the overall protocol comprises of the following steps:

1) use chemical shift information to run TALOS for the prediction of secondary structure

2) use secondary structure prediction from TALOS files for fragment picking

3) run ab initio structure prediction WITHOUT NMR constraints to get a baseline of the score distribution

4) rescore these decoys with NMR constraint data to get score distribution WITH NMR data

5) from both score distributions of the models WITH and WITHOUT NMR data, compute the optimal weight of the NMR score term

6) run ab initio structure prediction WITH NMR constraints with the optimized weight

Note that the protocol on the test server only runs the last step (step 6) and both fragment picking and constraint weight optimization has been done beforehand.

Runtimes are about 170 CPU hours for this test: (100s per model per target) x (3 targets) x (2000 decoys)

PERFORMANCE METRICS

Output files of structure prediction are a score file and a binary silent file. We look at the score file and plot the score-vs-rmsd distribution of the created models. Passes are defined by the cutoffs for all to be true: 10% of the models below the RMSD cutoff, 10% of the scores below the score cutoff and PNear higher than the PNear cutoff, defined by the first run minus 0.1. All cutoffs were defined by the first run of the protocols and adjusted over several runs.

KEY RESULTS

We compare the results against the benchmarks described in (Kuenze, Structure, 2019). 2k5u doesn't sample as low RMSDs as in the paper, from the first, single run it is unclear why. Further investigation will be required.

DEFINITIONS AND COMMENTS

LIMITATIONS

The run times are on the higher end, which is why we're only testing 3 proteins on the test server. Ideally, it would be nice to run all benchmarks from the paper on the test server, but this is computationally prohibitive. Target diversity, size and complexity of the full benchmark are well-chosen and optimized.

REVISION

revision: 61711

test_id: 713285

Scientific test: antibody_grafting

FAILURES

None

RESULTS

Briefly, the rmsd histograms plot the results for the benchmark (below) and are separated by structural region. Along the x-axis of each plot are notches showing the rmsd values for each model. The red line is the cutoff. Note that a certain number of models are expected to be above the cutoff, so having just one or a few past the line will not trigger a failure.

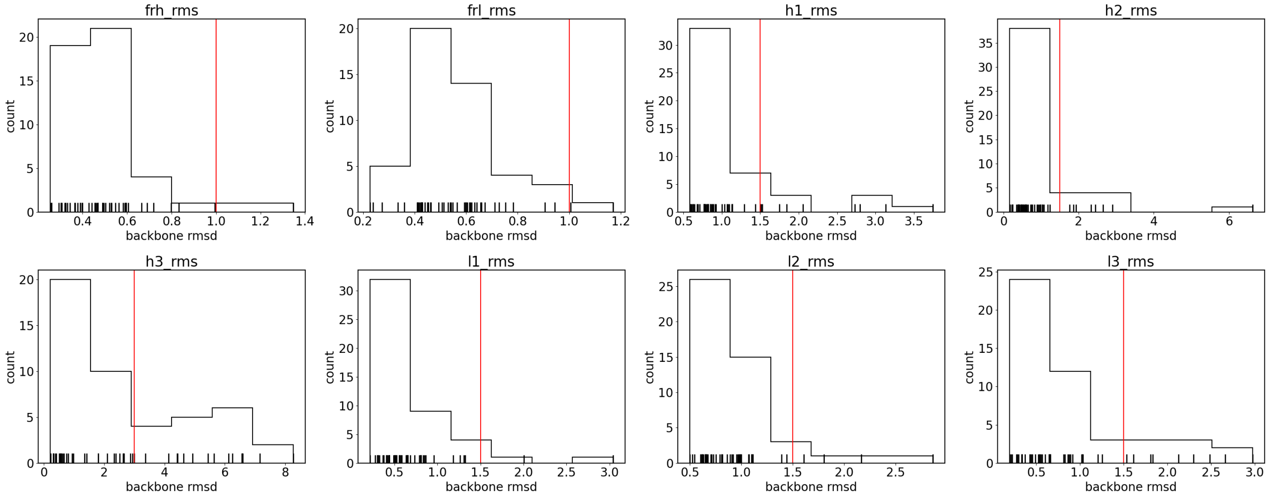

Inidivual PDBs off by more than X A

Listed below are, for each region, the PDBs missing the cutoff. Note these will only result in a test failure of some percentage greater than the cutoff fail. This list is for debugging purposes.

<u>frh_rms > 1.0 A</u>
3nps,

<u>frl_rms > 1.0 A</u>
1mfa, 2fb4,

<u>h1_rms > 1.5 A</u>
1oaq, 2r8s, 3go1, 3liz, 3lmj, 3nps, 3p0y, 3umt, 4hpy,

<u>h2_rms > 1.5 A</u>
1oaq, 1seq, 2r8s, 3go1, 3lmj, 3nps, 3oz9, 3p0y,

<u>h3_rms > 3.0 A</u>
1gig, 1oaq, 1seq, 2d7t, 2e27, 2fb4, 2vxv, 2ypv, 3giz, 3go1, 3liz, 3lmj, 3m8o, 3nps, 3umt, 4nzu,

<u>l1_rms > 1.5 A</u>
2r8s, 3giz,

<u>l2_rms > 1.5 A</u>
1gig, 1jfq, 1oaq, 2w60,

<u>l3_rms > 1.5 A</u>
2e27, 2fbj, 2xwt, 3go1, 3lmj, 3nps, 3oz9, 3p0y, 4h20,

AUTHOR AND DATE

Jeliazko Jeliazkov (jeliazkov@jhu.edu), of Prof. Jeffrey J. Gray's lab, setup this test in April 2019.

PURPOSE OF THE TEST

This test evaluates the RMSDs of regions (FRH, FRL, FRH--FRL, CDR H1, CDR H2, CDR H3, CDR L1, CDR L2, and CDR L3) grafted by the antibody grafting application (apps/public/antibody/antibody.cc).

BENCHMARK DATASET

The test evaluates 48/49 antibodies, first described in (Marze, N. A., Lyskov, S. & Gray, J. J. Improved prediction of antibody VL--VH orientation. Protein Engineering, Design and Selection 29, 409--418 2016). We exclude 3mlr because it has an atypical CDR L3.

There are two components to the test: (1) Chothia-numbered structures downloaded from the antibody database SAbDab and truncated to the Fv region (~residues 1--112) and (2) FASTA sequence files derived from these structures. The sequences are used as protocol input and the structures are used for rms evaluation.

This set of antibodies was also used for CDR-H3 loop modeling evaluation in (Weitzner, B. D. & Gray, J. J. Accurate Structure Prediction of CDR H3 Loops Enabled by a Novel Structure-Based C-Terminal Constraint. The Journal of Immunology 198, 505--515 2016).

PROTOCOL

The antibody modeling protocol is described in (Weitzner, B. D. et al. Modeling and docking of antibody structures with Rosetta. Nature Protocols 12, 401--416 2017).

The original protocol comes from (Sivasubramanian, A., Sircar, A., Chaudhury, S., & Gray, J. J. Toward high-resolution homology modeling of antibody F v regions and application to antibody-antigen docking. Proteins 74, 497-514 2009).

Here we test only the grafting step, in which an input antibody sequence is decomposed into structural regions (FRs and CDRs) then homologs for those regions are selected (by BLAST) and combined into a *single model*. Grafting is based on sequence identity and is deterministic, unless the sequences in the database change, so only a single model is produced per simulation. Grafting takes ~5 mins to run. This benchmark should take at most 200 CPU minutes.

Special note: in the full antibody modeling protocol, models are relaxed and we generate multiple models with different light-heavy chain orientations. Since we are not testing the full protocol here, there is no need to do this. We are only testing template selection and grafting.

PERFORMANCE METRICS

A single grafted model is produced per target antibody. The quality of a grafted antibody model is evaluated over four structural regions in each of two loops. The regions are a framework (FR) and three complementarity determining regions (CDRs). The metric of interest is backbone RMSD. For the FRs, this is evaluated after aligning on the regions. For the CDRs, this is calculated after FR alignment, so the CDR loops themselves are not aligned. The alignments are done with the cdr_backbone_rmsds() function in protocols/antibody/metrics.hh/cc.

For this benchmark, we ask what percentage of model regions are within X Angstrom of the native. FRs are structurally conserved across different antibodies. We expect little variation in this region, setting the threshold at 95% sub-Ansgtrom models. CDRs are a bit trickier. In the most recent blind evaluation of antibody grafting we modeled 46/55 (84%) non-H3 CDRs to sub-1.5-Angstrom accuracy. This value varies by CDR however, L3/H2 are the most erroneous whereas L1 & L2 rarely err, so we use a range of thresholds around there (L1 & L2 @ 90%, L3 @ 85%, H1 & H2 @ 80%). Finally, there is a special CDR loop (the H3) that is exceptionally diverse. It is rare to find structural homologs for this loops so we set the threshold at 65% sub-3 A models. Thresholds for the benchmark are summarized below:

FRs: 95% sub-Angstrom

non-H3 CDRs: 80--90% sub-1.5 A (depending on the CDR)

CDR H3: 60% sub-3 A

If the benchmark produces a lower percentage of sub-X-Angstrom models than described in the cutoffs, then the analysis scripts will signal "failure".

The latest assessment results are here (Weitzner, B. D., Kuroda, D., Marze, N., Xu, J. & Gray, J. J. Blind prediction performance of RosettaAntibody 3.0: Grafting, relaxation, kinematic loop modeling, and full CDR optimization. Proteins: Structure, Function and Bioinformatics 82, 1611--1623 2014). Although, these are for a small assessment.

For a full evaluation of the original protocol see: (Sivasubramanian, A., Sircar, A., Chaudhury, S., & Gray, J. J. Toward high-resolution homology modeling of antibody F v regions and application to antibody-antigen docking. Proteins 74, 497-514 2009).

KEY RESULTS

Most models should have ~ 1 Angstrom RMSD in most regions. See Blind prediction performance of RosettaAntibody 3.0 above for details.

DEFINITIONS AND COMMENTS

PDB ID 3MLR was removed from the benchmark because of its peculiar L3, but could potentially be investigated further.

LIMITATIONS

I have not tested if relaxing improves/worsens results.

REVISION

revision:61598

test_id: 689285

status: passed

Scientific test: antibody_h3_modeling

FAILURES

None

RESULTS

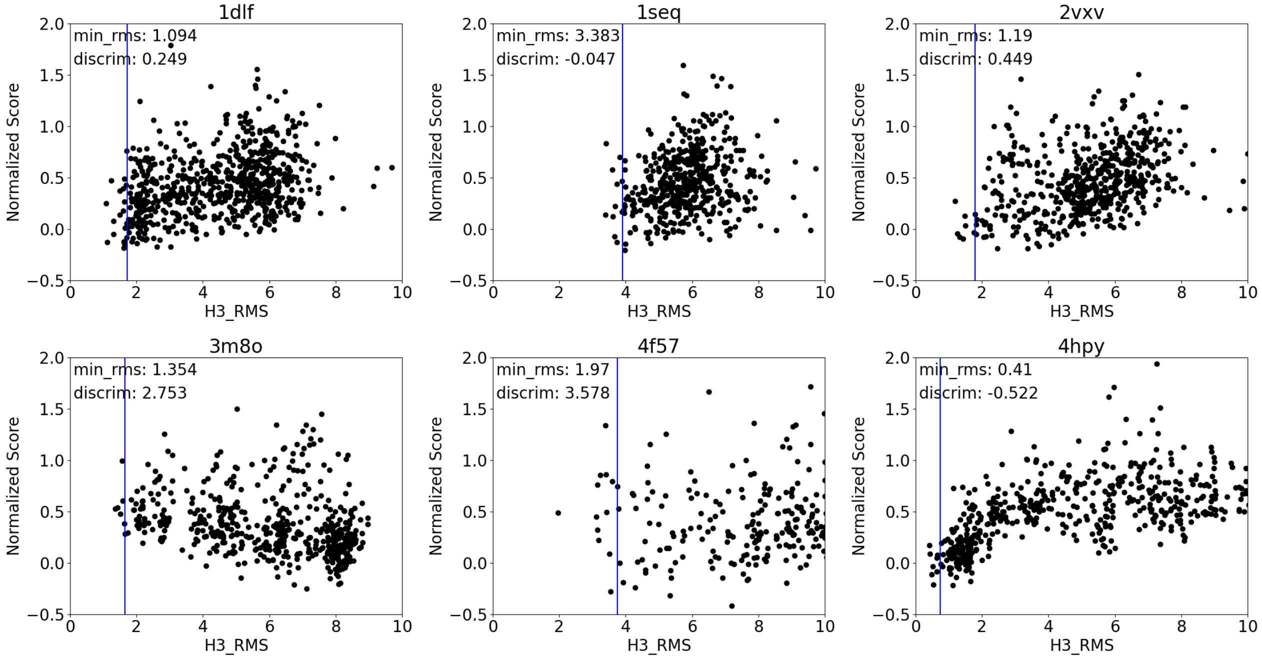

AUTHOR AND DATE

Antibody CDR-H3 loop modeling scientific test first implemented June, 2019 by Jeliazko Jeliazkov (Gray Lab, JHU, jeliazkov@jhu.edu).

PURPOSE OF THE TEST

This test seeks to evalute the executable antibody_h3. This application models the CDR-H3 loop (typically on homology frameworks, but also on crystal structures). Here we test its ability to predict loop conformation on crystal structures by evaluating minimum rmsd and discrimination. Minimum RMSD indicates whether or not we are sampling native-like loop conformations. Discrimination indicates whether the score function can distinguish native from non-native. Both metrics are compared to 99-th percentile value determined by bootstrap resampling of previous simulations.

BENCHMARK DATASET

The dataset comprises six antibody targets from Weitzner and Gray (J. Immunol. 2016). The targets are of varying difficulty and CDR H3 length. 1DLF (12 residues) and 4HPY (13 residues) are easy targets. 1SEQ (16 residues) and 4F57 (18 residues) are hard targets. 2VXV (14 residues) and 3M8O (10 resiudes) are intermediate targets. Native structures are un-relaxed crystals. Inputs for modeling are relaxed with constraints (no ramping).

PROTOCOL

The Rosetta Antibody CDR-H3 loop modeling protocol is describe in our publication (Weitzner, Jeliazkov, Lyskov, et al., Nat. Protocol. 2017):

Briefly, this protocol has three stages (1) de novo CDR-H3 loop modeling, (2) VH-VL orientation refinement, (3) CDR-H3 loop refinement. Throughout the protocol the CDR-H3 loops is constrained to occupy the kink conformation observed in ~90% of antibodies. The benchmark is rather time consuming, taking ~1 hour per model. We aim to produce 500 models for 6 antibodies, so the runtime is 3000 CPU hours.

PERFORMANCE METRICS

To consider a run successful, for antibody_h3 run, we would like to produce a low energy, low rmsd model. Thus we need to assess two measures: (1) minimum rmsd achieved (do we sample a native like state) and (2) discrimination (do low rmsd models have low scores) We use discrimintaiton (typically lower is better, ideally negative values) as defined by Conway et al. Prot. Sci 2014, although our rmsd

bins have adjusted spacing at min(rmsd) + (0, 0.5, 1, 1.5, 2, 4). We derive cutoffs for these metric from bootstrap resampling previous simulations (see below). The discrimnation or minimum rmsd test for each antibody is "passed" if the value is lower than that observed in the 99th percentile of our resampled simulations.

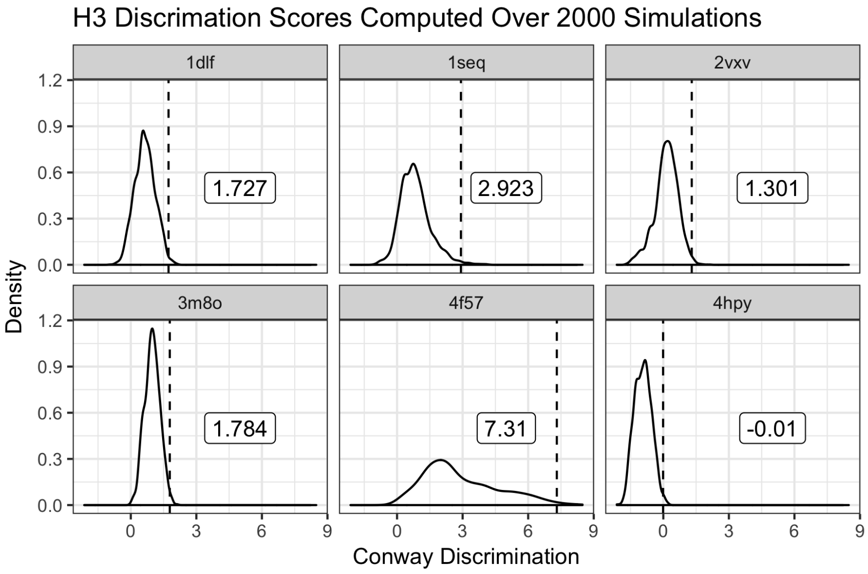

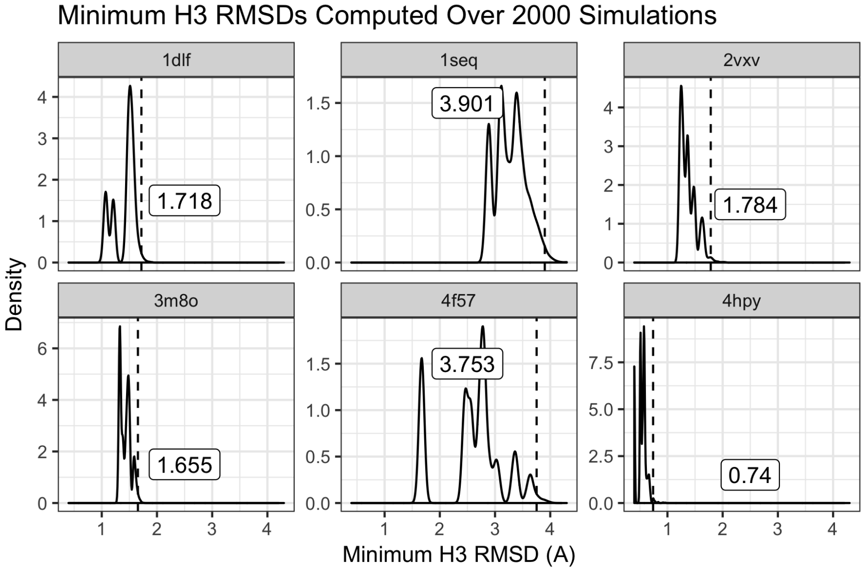

The exception is 3m8o whose discrimination score cutoff was adjusted after changes to the antibody code by Jeliazko in revision 61279 created a permanent scientific failure for this target. The cutoff was adjusted to 3.5 after looking at the discrimination score in the 12 consecutive failures of this test.

KEY RESULTS

Antibody CDR-H3 loop structure prediction is a challenging task. The current test assesses two easy (1DLF [12 residues] and 4HPY [13 residues]), two medium (2VXV [14 residues] and 3M8O [10 residues]), and two hard (1SEQ [16 residues] and 4F57 [18 residues]) targets. For the easy/medium cases we expect a minimum rmsd between 0 and 2 Angstroms, whereas for the hard cases the rmsd will be greater than that. We expect discrimination to always be below zero for 4hpy. For 1dlf and 2vxv, discrimination should be below 2. For the others discrimination will be worse. Unfortunately, discrimination is not the most stable metric, so we are exploring alternatives.

DEFINITIONS AND COMMENTS

This test is a first stab at an antibody CDR-H3 loop modeling scientific test. More work should be done to determine better targets (other Abs? more Abs? different protocol?) and metrics. In particular, discrimination varies across simulations.

LIMITATIONS

The full assessments for Rosetta Antibody typically cover the entire protocol from homology modeling to H3 modeling and consist of ~50 targets. Due to time considerations, we obviously do not test the full protocol over 50 antibodies here. Instead we focus on the

crucial H3 modeling stage.

REVISION

revision:61592

test_id: 687737

status: passed

Scientific test: antibody_snugdock

FAILURES

None

RESULTS

Funnel plots are not used to assess quality, but may helpful to the user.

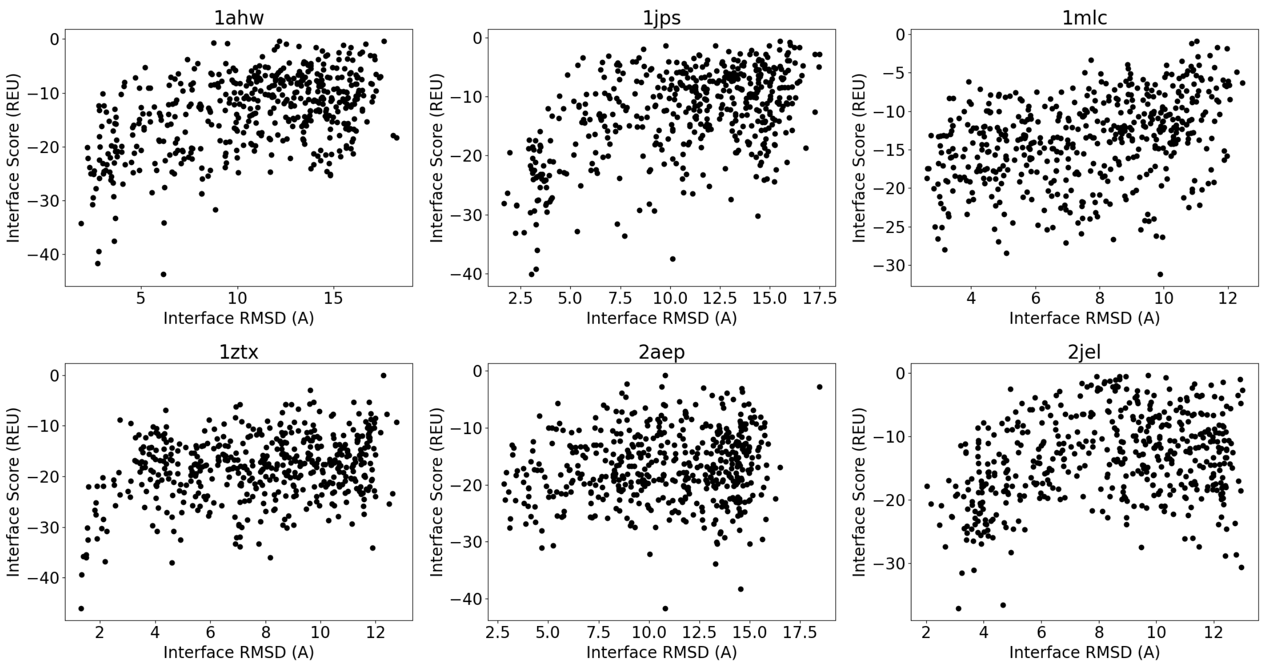

Two conditions must be met to pass the test. (1) At least one model of acceptable quality must be among all models and (2) either enough medium quality or enough acceptable quality models are in the top 10 (lowest interface energy) models.

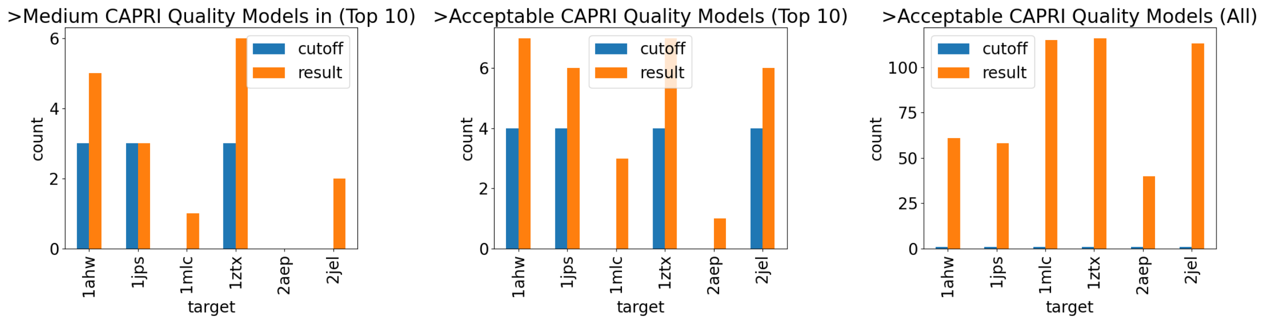

AUTHOR AND DATE

SnugDock scientific test first implemented June, 2019 (revised Oct, 2020) by Jeliazko Jeliazkov (Gray Lab, JHU, jeliazkov@jhu.edu).

PURPOSE OF THE TEST

This test seeks to evalute the executable snugdock. Briefly, this application simulates antibody--antigen interactions by local docking with CDR loop refinement. A simulation consists of fifty cycles of randomly selected moves, including, re-docking the relative orientation of the VH--VL and Ab--Ag are refined, remodeling the CDR H3/H2 loops in the context of the Ag interaction, side-chain repacking, and minimization of all relevant DOF (Ab--Ag interface, VH--VL interface, CDR loops, and side chains).

Here we test the protocol's ability to predict Ab--Ag interactions starting from ensembles of homology models and the unbound antigen crystal structure (when possible). We evaluate success/failure based on the CAPRI quality criteria of the lowest energy (based on interface score) models.

BENCHMARK DATASET

The dataset comprises six antibody targets from Sircar and Gray (PLoS Comp. Bio. (2010)). The targets are of varying difficulty (1AHW, 1JPS, 1MLC, and 1ZTX should be relatively easy - either sampling near-native states or having a funnel - whereas 2AEP and 2JEL should be challenging - no funnel, no near-native sampling). Native structures are crystals. Inputs for modeling are RosettaAntibody homology models and relaxed (unbound when possible) antigen.

PROTOCOL

The Rosetta SnugDock protocol is described in our publication (Weitzner, Jeliazkov, Lyskov, et al., Nat. Protocol. 2017). The benchmark is rather time consuming, taking ~1 hour per model. We aim to produce 500 models for 6 antibodies, so the runtime is 3000 CPU hours.

PERFORMANCE METRICS

To consider a simulation successful, we would like to sample native states (low rmsd) and identify those states based on low interface energy. For this test to be passed, all simulations must produce at least one model of acceptable quality (according to the CAPRI criteria). Additionally, for the four easy targets, we expect low-energy models to have medium or acceptable quality. Thus, 1ahw, 1jps, and 1ztx must either produce at least 4 acceptable quality models or at least 3 medium quality models in the 10 lowest energy models (by interface score). 2jel, a slightly more challenging target must produce at least 4 acceptable quality models.

KEY RESULTS

Antibody--antigen bound structure prediction is a challenging task. The current test assesses only six of a possible fifteen targets, which should display a breadth of behavior. For the easier targets (1AHW, 1JPS, 1MLC, and 1ZTX) we expect to observe either near-native (sub 2-Angstrom) sampling of the interface or a good funnel (negative discrimintation). For the harder targets (2AEP and 2JEL) we will not observe either.

LIMITATIONS

The full assessment for Rosetta SnugDock typically covers the entire protocol from homology modeling to H3 modeling to antigen docking and consists of 15 targets. Due to time considerations, we obviously do not test the full protocol over 15 antibody-antigen complexes here. Instead we focus on six representative targets.

REVISION

revision:61588

test_id: 686998

status: passed

Scientific test: cofactor_binding_sites

FAILURE:

False
The test passed! You can learn more about CoupledMoves at https://www.rosettacommons.org/docs/latest/coupled-moves

RESULTS

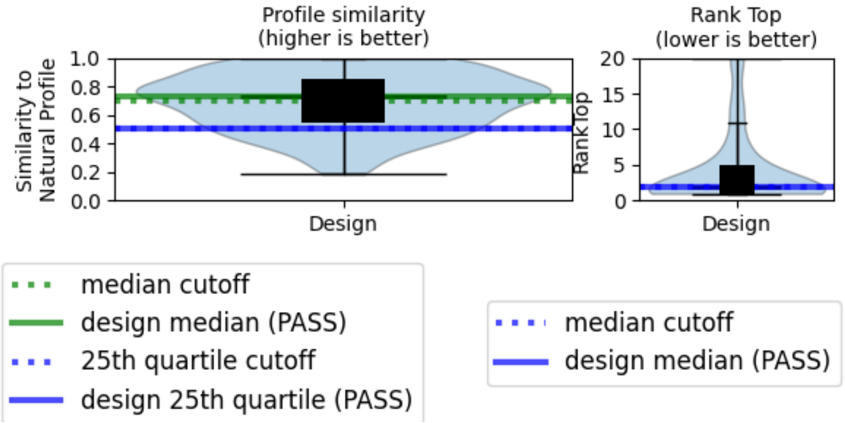

AUTHOR AND DATE

Author: Amanda Loshbaugh (aloshbau@gmail.com)

PI: Tanja Kortemme (kortemme@cgl.ucsf.edu)

Date test first added to Scientific Benchmark: February 2019

PURPOSE OF THE TEST

Evolutionary sequence alignments for seven naturally occurring protein families that each bind a specific cofactor the Cofactor binding site dataset, which is made up of sequence profiles resulting from natural evolution evolutionary sequence profiles of cofactor-binding protein families has the advantage of clearly identifying consensus binding positions.

BENCHMARK DATASET

The cofactor binding sites dataset is comprised of seven separate protein families, with the conserved small molecule cofactor binding sites of each family. This dataset is described in detail elsewhere.[3]

In brief, each protein family satisfies three criteria: 1) there exists "at least one representative crystal structure bound to the cognate ligand to use as input for design, 2) the protein family has a large number of diverse sequences such that the binding site is not completely conserved, and 3) all members of the protein family are capable of binding the cognate ligand using the same ligand binding site. Ligand binding positions are defined as any amino acid position with a side-chain heavy atom within 6Å of any heavy atom in the co-factor ligand. Natural sequences of these binding sites were obtained using the protein family alignment from Pfam and filtered to remove all redundant sequences.[3]

This dataset that does not involve experimental characterization of binding affinity. Because natural proteins are under many different selection pressures, the affinity of natural sites is usually just good enough rather than as tight as possible.

PROTOCOL

The CoupledMoves protocol is described at https://www.rosettacommons.org/docs/wiki/coupled-moves.

PERFORMANCE METRICS

Tolerated sequence space, for each position in a given protein, describes the diversity of amino acids compatible with the protein's function,[2] and is calculated from the known natural sequence profiles. We quantify Rosetta's ability to recapitulate the natural sequence profiles at the defined ligand binding positions by two metrics, position profile similarity (PPS) and rank top (RT). Metrics are calculated per position.

PPS measures the similarity of the distribution of amino acid frequencies between two datasets, the known profile (from natural sequence alignment) and the profile generated by Rosetta design.

RT measures the rank, in the design profile, of the amino acid most frequently observed in the known profile.

When benchmarked on this dataset in [1], CoupledMoves achieved PPS with median ~0.72 on this dataset, and 25th percentile ~0.56. Accordingly, thresholds of 0.7 (green) and 0.5 (blue) are chosen for the median and 25th percentile in this test.

KEY RESULTS

This benchmark describes the ability of the Rosetta protocol CoupledMoves to recapitulate sequence profiles of naturally evolved proteins.

LIMITATIONS

Sequence profiles in the cofactor dataset result from natural evolution, rather than experimental screening. Natural evolution includes additional selection pressures beyond affinity (function, stability, kinetics), the full consequences of which are difficult to categorize and quantify, meaning that the sequence profiles for natural binding site positions may be influenced by factors beyond those modeled by Rosetta. However, using evolutionary sequence profiles of cofactor-binding protein families has the advantage of clearly identifying consensus binding positions.

More data, in the form of more natural ligand binding protein families, would improve this benchmark.

REFERENCES

[1] Loshbaugh, A. L. and Kortemme, T. Comparison of Rosetta flexible-backbone computational protein design methods on binding interactions. Manuscript in preparation. 2019.

[2] Humphris, E.L. and T. Kortemme, Prediction of protein-protein interface sequence diversity using flexible backbone computational protein design. Structure, 2008. 16(12): p. 1777-88.

[3] Ollikainen, N., de Jong, R. M., and Kortemme, T. Coupling protein side-chain and backbone flexibility improves the re-design of protein-ligand specificity. PLoS Comput Biol, 2015.

REVISION

revision:61592

test_id: 687728

status: passed

Scientific test: ddg_ala_scan

FAILURES

None

RESULTS

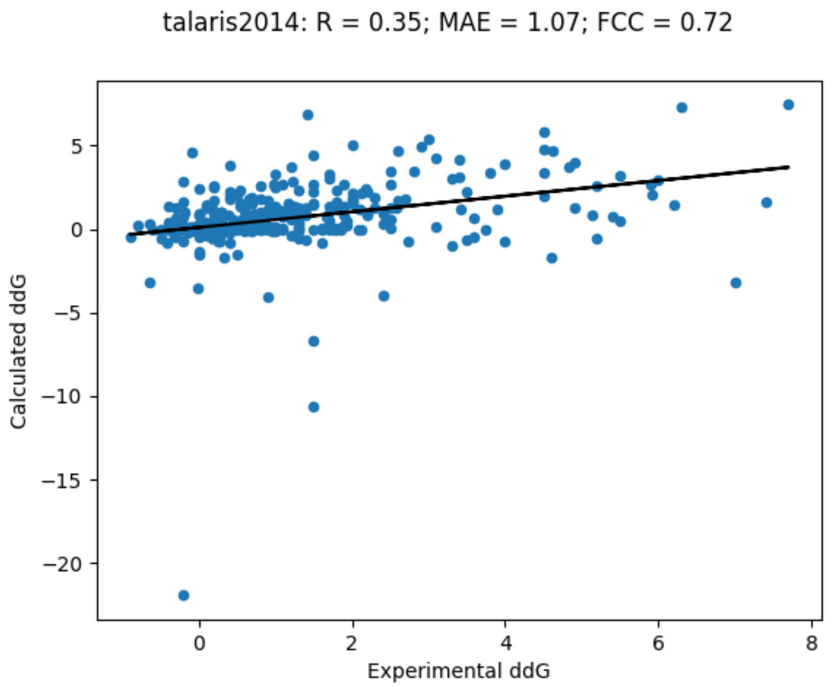

AUTHOR AND DATE

Set up by Ajasja Ljubetic (ajasja.ljubetic@gmail.com), Baker lab, Sept. 2019

This is a port of the Kortemme lab benchamrk (https://github.com/Kortemme-Lab/ddg).

Original benchmark done by Shane Connor, Kyle Barlows, Andrew Leaver-Fay, Tanja Kortemme & David Baker.

Metrics used in evaluation

R: correlation coefficient (>.33)

MAE: Mean Absolute Error (<1.1)

FCC: Fraction Correctly Classifed (>.72)

PURPOSE OF THE TEST

The benchmark test the correlation between predicted and experimental ddG upon a mutation from a native residue to Ala.

The alanine scanning protocol avoids any perturbation of the backbone or side chains, other than the residue being mutated, which is placed into a low-energy rotamer using the Rosetta â€œpackerâ€. This minimal perturbation relies on the fact that the overall protein structure is unlikely to change much after a single point mutation to alanine, making the input crystal structure a good approximation for the mutant structure.

As the alanine scanning protocol does not perturb the protein backbone or side chains (other than the mutant residue), this protocol is not suitable for use on mutations outside of the interface. A mutation outside of the interface will result in a negligible change in total score without the use of a more intensive sampling protocol.

As in Î”Î”G, the metrics used to measure success in this benchmark are: i) the linear correlation (Pearson coefficient) between experimental and predicted values; ii) the mean absolute error (MAE) of same; and iii) the FCC (fraction correctly classifed). FCC is stability classification accuracy, which measures whether a mutation was correctly predicted to be stabilizing, destabilizing, or neutral.

BENCHMARK DATASET

The benchmark is comprised of experimental data for 381 mutations. The datasets are taken from the following publications (PubMed IDs are specified):

7504735, 9571026, 10970748, 9050852, 1281426, 2479414, 9480775, 8494892, 7739054, 9425068, 8784199, 7654692,

10678837, 10880432, 8332602, 10452608, 2402498, 9500785,

11123892, 9878445, 9579662, 8703938, 8263942, 9609690,

10338006

The input files were creating using the instructions found here: https://github.com/Kortemme-Lab/ddg/tree/master/protocols/alanine-scanning

No minimisation is done on the input structures.

This benchmark includes:

a previously published set of alanine mutations in 19 different protein-protein interfaces with known crystal structures (see Kortemme & Baker, 2002);

scripts to run a new RosettaScripts protocol which has been designed to emulate the protocol described in Kortemme & Baker (2002);

an analysis script that output the metrics used for analysis. The script also outputs a scatterplot plotting experimental Î”Î”G values against predicted values (in whichever scoring unit is used by the protocol);

PROTOCOL

The protocol uses Rosetta scripts to change a residue from it's native type to an Ala. The ddG of this mutation is calculated.

There is no minimization/relaxation step of the entire structure.

PERFORMANCE METRICS

The performance metrics are the the correlation coefficient (R) between the experimental and calculated ddG values. The cutoffs were chosen so that the benchmark passed in 74cba0b67deb889d2ae4e4cf519fba9ae9210 commit of Rosetta.

KEY RESULTS

The correlation is compared to experimental data. The correlation should be better than 0.33 for all the energy score terms.

DEFINITIONS AND COMMENTS

LIMITATIONS

Some experimental values are listed as >4. These are currently ignored.

There is no minimization/relax after the mutation is performed. Adding a minimization and repacking might improve the correlation.

Also currently only talaris2014 is tested.

Technically resfiles are not needed (the XML script could be rewritten to take the position IDs directly.)

REFERENCES

Kortemme, T, Baker, D. A simple physical model for binding energy hot spots in proteinâ€“protein complexes. Proc Natl Acad Sci U S A. 2002 Oct 29;99(22):14116-21. Epub 2002 Oct 15. doi: 10.1073/pnas.202485799.

Kortemme T, Kim DE, Baker D. Computational alanine scanning of protein-protein interfaces. Sci STKE. 2004 Feb 3;2004(219):pl2. doi: 10.1126/stke.2192004pl2.

REVISION

revision:61603

test_id: 690468

status: passed

Scientific test: design_fast

FAILURES

None

RESULTS

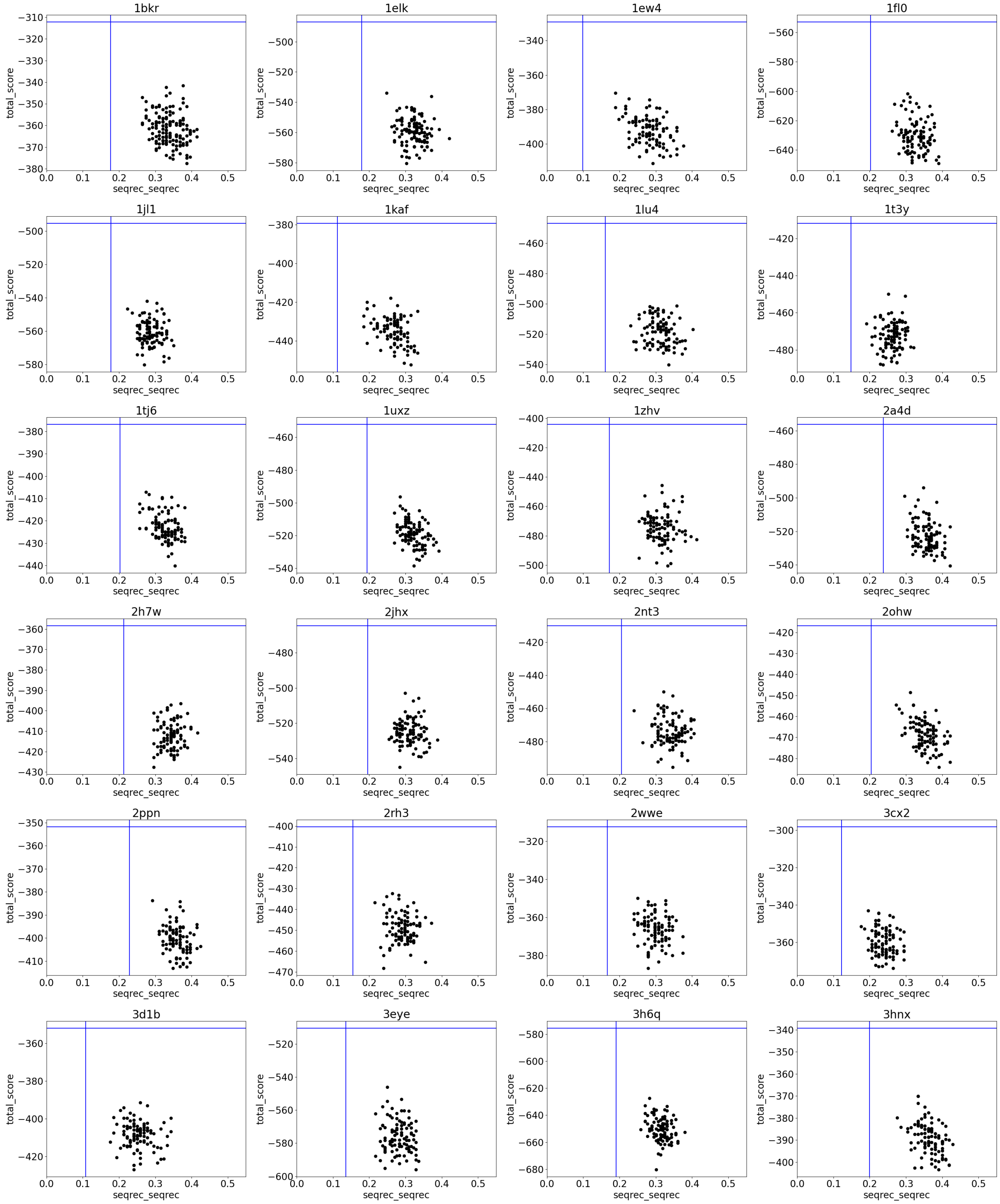

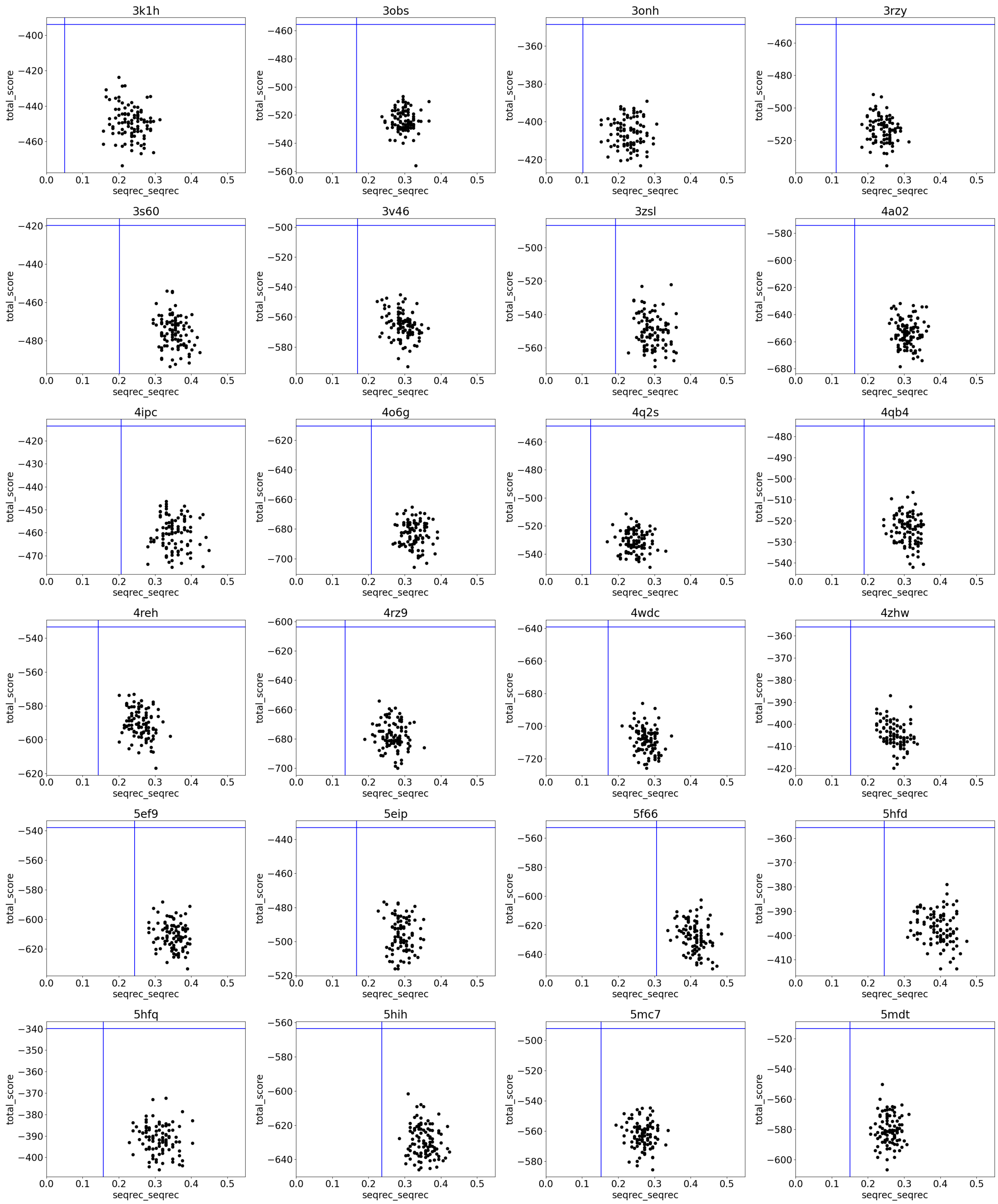

AUTHOR AND DATE

This benchmark was set up by Julia Koehler Leman (julia.koehler.leman@gmail.com), PI Richard Bonneau, March 2020

Input data and command lines are from Chris Bahl and Jack Maguire.

PURPOSE OF THE TEST

The benchmark tests how well FastDesign can recover native sequences on the benchmark set.

BENCHMARK DATASET

The benchmark set contains 48 proteins between 102 and 176 residues, originally used by Frank DiMaio for his improvements to the energy function. The set covers alpha-helical bundles, beta-sheets proteins and mixed alpha/beta folds.

PROTOCOL

The protocol runs FastDesign in RosettaScripts currently with 1 iteration, nstruct 100, no extrachi. Probably should try 5 iterations as originally suggested. 1 iteration generates a decoy in about 2000 seconds. This makes this protocol run for about 48 x 100 x 2000 / 3600 = 2666 CPU hours.

PERFORMANCE METRICS

We use sequence recovery between the native and the design computed via SimpleMetrics in RosettaScripts. The cutoffs were defined for sequence recovery, for each protein take the minimum minus 2 stdev. For the score, per protein take the maximum plus 5 stdev.

KEY RESULTS

The sequence recovery metric has been used for many years to benchmark design applications. Historically, sequence recoveries are somewhere between 30% and 60% at the maximum. It is difficult for the scorefunction to recapitulate native sequences accurately. It might be worth noting that we do not expect 100% sequence recovery even with a "perfect" energy function and "perfect" optimizer, since evolution optimizes proteins for marginal stability (to allow for degradation) and for other things (function, genetic code, amino acid costs/abundances), while we're trying to optimize for high stability (and maximize the stability of the designed state, without knowing what we're doing to the stability of alternative conformations).

DEFINITIONS AND COMMENTS

LIMITATIONS

The benchmark set only consists of small, soluble proteins. It would be good to know how design performs on larger proteins and more complex folds. For the quality metrics, rotamer recovery could be considered as well.

REVISION

revision:61592

test_id: 687751

status: passed

Scientific test: dock_glycans

FAILURES

None

RESULTS

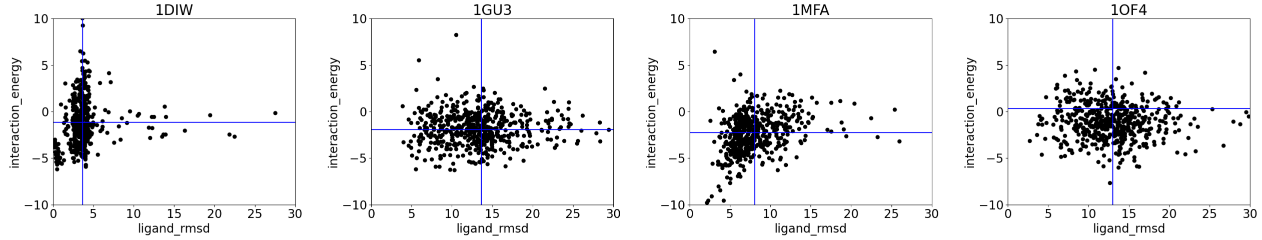

AUTHOR AND DATE

Labonte <JWLabonte@jhu.edu>, Gray Lab, 2019.06

PURPOSE OF THE TEST

This benchmark confirms that the dock_glycans protocol is able to determine high-quality glycoligand models by CAPRI metrics in a bound–unbound local, flexible docking run.

BENCHMARK DATASET

There are 4 protein–glycoligand complexes in the benchmark set, which were selected from a much larger set found in [Anita K. Nivedha, *et al. J. Chem. Theory Comput.* **2016**, *12* 2892–901]. The four selected complexes were initially chosen for their variety of size and success in the protocol.

The input files are relaxed, native `.pdb` files.

PROTOCOL

The initial protocol being run here is the dock_glycans protocol published with [Labonte, J.W.; Adolf-Bryfogle, J.; Schief, W.R.; Gray, J.J. â€œResidue-Centric Modeling and Design of Saccharide and Glycoconjugate Structures.â€ *J. Comput. Chem.* **2017**, *38*, 276â€“287]. 500 decoys are generated for each of the four input structures. An individual constraint file with a single site constraint makes sure that the sugar doesn't move out of the binding pocket.

PERFORMANCE METRICS

Interface energy is plotted *vs.* ligand RMSD. Pass/fail is currently determined by having at least 30% of the decoys below the mean for both ligand RMSD and interface energy, i.e. the lower left quadrant.

KEY RESULTS

DEFINITIONS AND COMMENTS

LIMITATIONS

After we publish our results with an enhanced GlycanDock protocol on the entire benchmark, we will expand the test here. Ideally, cutoffs should be determined by decoys considered to be of high-quality by CAPRI metrics.

REVISION

revision:61603

test_id: 690509

status: passed

Scientific test: docking

FAILURES

None

RESULTS

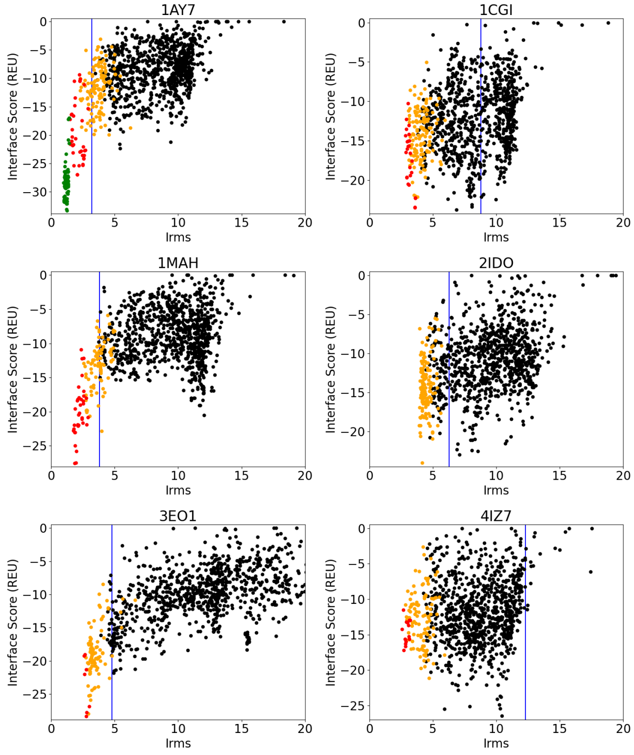

AUTHOR AND DATE

Adapted for the current benchmarking framework by Shourya S. Roy Burman (ssrburman@gmail.com; Gray Lab), Sep 2019

PURPOSE OF THE TEST

This benchmark is meant to test how well we discriminate native protein-protein binding orientations from decoys based on the interface score term by performing standard protein-protein docking experiments across a diverse set of protein-protein complexes.

BENCHMARK DATASET

The dataset consists of 10 protein-protein complexes extracted from the Docking Benchmark 5.0 (Vreven, T. et al. J. Mol. Biol., 2015). The set contains 4 rigid (conformational change < 1.5 Ang), 4 medium-flexible (conformational change between 1.5 and 2.2 Ang), and 2 difficult (conformational change > 2.2 Ang).

Structure preparation:

Proteins were extracted directly from the PDB according to the PDB ID of the native structure. They were then cleaned to ensure that both the bound and the unbound structures have the same residues. The unbound structures were superimposed on the bound and then the smaller partner (ligand) was moved away by 15 Ang and rotated by 60 degrees to scramble the interface

PROTOCOL

Protocol (as per the publication below): "RosettaDock is a Monte Carlo-plus-minimization algorithm consisting of a low-resolution stage, which simulates conformer selection during the formation of the encounter complex, followed by a high-resolution stage, which simulates induced fit in the bound complex. To produce a variety of starting states for the different trajectories, the ligand (the smaller protein) is first randomly rotated and translated about the receptor (the larger protein). In the low-resolution stage, side chains are replaced by coarse-grained "pseudoatoms", allowing the ligand to efficiently sample the interface by rigid-body movements in a smoothened energy landscape. These rigid-body moves are coupled with backbone conformation swaps where the current backbone conformations of the ligand and the receptor are swapped with different ones from a pre-generated ensemble of conformations. In the high-resolution stage, the side chains are reintroduced to the putative encounter complex and those at the interface are packed for tight binding. There is minimal rigid-body motion in this second stage."

Publication:

The methodological details of the protocol, RosettaDock 4.0 and the performance on the benchmark have been thoroughly discussed in Marze, N. A., Roy Burman, S. S. et al. Bioinfo., 2018.

CPU hours:

The benchmark takes ~150 hours. The debug mode takes ~4 mins.

PERFORMANCE METRICS

Usually, to assess the performance of a docking simulation, the number of structures with a CAPRI-acceptable or better ranking are analyzed. CAPRI model rankings are based on a combination of factors like fraction of native contacts, ligand RMSD, and interface RMSD and are described in detail in Lensink, Wodak et al. 2019 PSFBI - Table 3. The protocol computes the CAPRI rankings for each model (as well as some of the metrics it is based on), which are written into the score file ("CAPRI_rank"). Rankings are the following:

0 - incorrect model

1 - acceptable model

2 - medium quality model

3 - high quality model

The output models are resampled via bootstrap to remove possible sampling biases. Docking complexes can also be classified via the N5 metric, classifying N5 >= 3 as successful. N5 >= 3 means that at least 3 out of the top 5 scoring models should be acceptable or better according to CAPRI metrics. In this test, we report this metric in the result.txt file but don't use it for a pass/fail criterion because most targets would fail according to it. This scientific test passes if all targets pass the following metrics:

(1) the highest CAPRI ranking sampled for any model should be equal or higher than the cutoff ranking (as computed in the first run) AND

(2) the interface RMSD of the top-scoring model should be equal or lower than the cutoff I_rmsd (as computed in the first run + 2A)

KEY RESULTS

Unbound structures are docked and compared to the bound native structure.

DEFINITIONS AND COMMENTS

For speed, this test does not use conformational enembles of unbound proteins. For best results, use ensembles as described in the article.

LIMITATIONS

This is a small part of a much larger standard docking benchmark used in the docking community (Vreven, T. et al. J. Mol. Biol., 2015). More targets from that benchmark will help.

REVISION

revision:61582

test_id: 687172

status: passed

Scientific test: enzyme_design

FAILURES

(NONE)

RESULTS

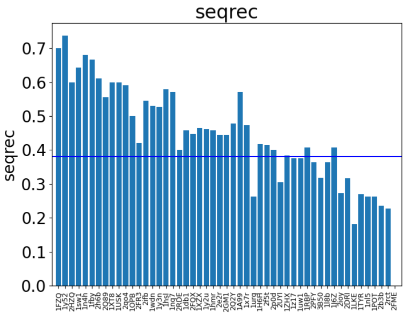

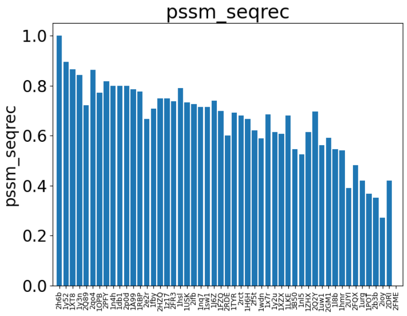

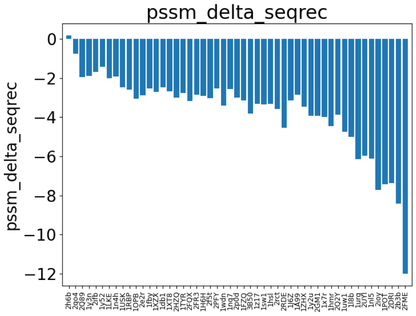

Protein	pssm_delta_seqrec	pssm_seqrec	seqrec
AVERAGE	-3.682	0.652	0.443
1A99	-2.857	0.786	0.571
1FZQ	-3.150	0.700	0.700
1H6H	-2.917	0.667	0.417
1J6Z	-3.148	0.741	0.407
1LKE	-2.000	0.682	0.182
1OPB	-3.045	0.773	0.500
1POT	-7.421	0.368	0.263
1RBP	-2.593	0.778	0.407
1TYR	-2.769	0.692	0.269
1USK	-2.467	0.733	0.600
1XT8	-2.667	0.867	0.600
1XZX	-2.714	0.607	0.464
1ZHX	-3.462	0.615	0.385
1db1	-2.486	0.800	0.457
1fby	-2.542	0.708	0.667
1hmr	-4.458	0.542	0.458
1hsl	-3.316	0.789	0.579
1l8b	-5.000	0.545	0.364
1n4h	-1.920	0.800	0.680
1nl5	-6.105	0.526	0.263
1nq7	-2.571	0.714	0.571
1sw1	-3.357	0.714	0.643
1urg	-6.158	0.421	0.263
1uw1	-4.750	0.562	0.375
1wdn	-3.412	0.588	0.529
1x7r	-4.000	0.684	0.474
1y2u	-3.923	0.615	0.462
1y3n	-1.895	0.842	0.526
1y52	-1.421	0.895	0.737
1z17	-3.312	0.750	0.375
2DRI	-7.368	0.421	0.316
2FME	-12.000	0.000	0.000
2FQX	-3.172	0.483	0.448
2FR3	-2.842	0.737	0.421
2GM1	-3.926	0.593	0.444
2HZQ	-3.000	0.750	0.600
2PFY	-2.545	0.818	0.364
2Q2Y	-3.870	0.696	0.478

2Q89	-1.944	0.722	0.556
2RDE	-4.550	0.600	0.400
2UYI	-5.957	0.391	0.304
2b3b	-8.412	0.353	0.235
2e2r	-2.889	0.667	0.444
2f5t	-3.034	0.621	0.414
2h6b	0.167	1.000	0.611
2ifb	-1.682	0.727	0.545
2ioy	-7.727	0.273	0.273
2p0d	-3.000	0.800	0.400
2qo4	-0.773	0.864	0.591
2rct	-3.591	0.682	0.227
3B50	-3.818	0.545	0.318

AUTHOR AND DATE

Adapted for the current benchmarking framework by Rocco Moretti (rmorettiase@gmail.com; Meiler Lab), Sep 2018

PURPOSE OF THE TEST

This benchmark tests how well the enzyme design code is able to recapitulate native-like sequences when run over a set of cocrystal structures of small-molecule binding proteins with their native substrates.

BENCHMARK DATASET

There are 50 proteins in this set, chosen for being a high-quality structures of proteins binding to their native substrates. This benchmark set (and the basic protocol) is partly described in Nivon et al. (2014) "Automating human intuition for protein design." The input PDBs are from the previous benchmark tests, so their provenance is not 100% clear, but I believe that they have been downloaded from the RCSBV, minimally cleaned, and subjected to the all-atom constrainted relax protocol of Nivon et al. (2013) (probably under the score12/enzdes scorefunction).

PROTOCOL

The protocol follows more-or-less that of Nivon et al. (2014), updated for RosettaScripts XML. Briefly, the residues surrounding the ligand (design within 6 Ang (8 if pointed toward ligand) and repack within 10 (12) Ang) are subjected to 2 cycles of softpack/hardmin followed by 1 cycle of hardpack/hardmin. The protein-ligand interactions are upweighted by 1.8-fold for those residues being designed.

One big change from the publication is that the scorefunction being used is the (currently default) REF2015, rather than the older scorefunction used in the paper/in previous benchmarks. (It's the intention that the score function be updated based on whatever the current default is.)

Currently we are only running one output structure for each input, which results in the test taking somewhere around 25-50 CPU hours.

PERFORMANCE METRICS

The original test only looked at percent sequence recovery at designed positions; whether or not the design recaputulated the identical residue type as the input. ("seqrec") The concept is that, as the structures are native binders, their current sequence should be close to optimal for binding the ligand.

This reimplementation added two new metrics, based on matching the design result to a PSSM of the input protein. This PSSM was generated with the 2.6.0 version of psiblast, using the BLAST nr database from 21May2014 (yes, both the psiblast and nr database were old when this was done in late 2018) with the following command:

psiblast -query 1A99.fasta -db /path/to/db.21May2014/nr -out_pssm 1A99.chk -out_ascii_pssm 1A99.pssm -save_pssm_after_last_round

The first PSSM-based metric ("pssm_seqrec") is a percent recovery metric, but instead of attempting to match the input sequence exactly, it counts as a "success" matching any amino acid which has a favorable score in the PSSM. This metric is described in DeLuca et al. 2011. This is a 0-100% normalized scale which better allows for modest changes (e.g. S->T) which are permitted evolutionarily.

The second PSSM-based metric ("pssm_delta_seqrec") looks at the per-residue change in the PSSM score compared to the "native" input sequence. (Where more positive is a "better" design.) This attempts to capture the magnitude of the mutational change.

The benchmark set is summarized by averaging the scores for each protein. (This average is on a per-protein basis, and is not weighted by the number of positions mutated.)

The current tested cutoffs is based only on the match/fail seqrec metric, and is taken directly from the previous iteration of this benchmark. This value is somewhat arbitrary, and was set based on what didn't give overly noisy results when the benchmark was being run.

KEY RESULTS

I am unaware of any objective level one could compare the results to. Generally you'd be limited to a comparative performance based on prior results.

There's currently no analysis of outliers or per-protein performance, over and above anything discussed in Nivon et al. (2014).

DEFINITIONS AND COMMENTS

Comparing different runs and different scorefunctions, you're probably looking for something which is consistenty higher.

There's a bit of noise in the runs, so you may need to compare several runs to get better sense of how different scorefunctions compare.

LIMITATIONS

While the dataset attempted to be comprehensive (all structures which fit the criteria) when it was produced, the quality/breadth of structures these days may be better.

The preparation of the input structures may be another issue. The input structures may have been minimized/relax with an older version of the scorefunction. Updating the structure preparation may improve benchmark performance.

The run-to-run variablility is rather high, which might indicate that the benchmark could be improved by running several output structures for each input, rather than just the current one.

The exact protocol being used may not be optimal for ligand binding design, and a different packing/minimization scheme might improve performance.

Finally, keep in mind the intrinsic limitations of sequence-recovery based protocols. While low values are likely bad, you wouldn't necessarily expect the metric to saturate near a "perfect" score, as native proteins are optimized for more than just ligand-binding affinity, so even family PSSM profiles may not match the ideal design that Rosetta would be aiming for.

REVISION

revision:61592

test_id: 687664

status: passed

Scientific test: FlexPepDock

FAILURES

None

RESULTS

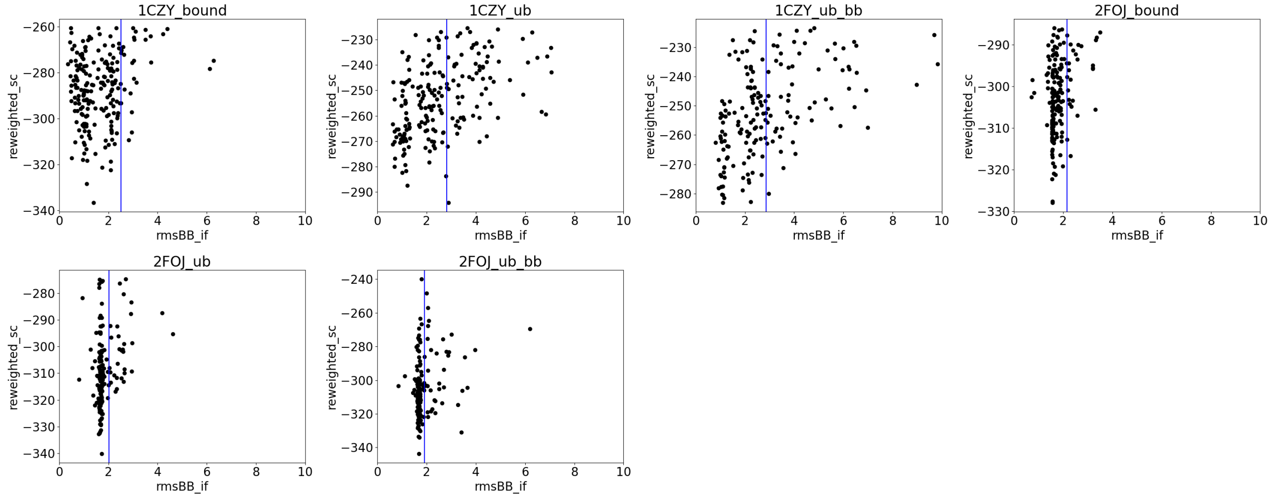

AUTHOR AND DATE

Configured by Ziv Ben-Aharon @ FurmanLab. June 2019. PI: Ora Furman-Schueler.

Contact info: ziv.benaharon1@mail.huji.ac.il, oraf@ekmd.huji.ac.il

PURPOSE OF THE TEST

This test showcases the execution of the FlexPepDock refine protocol on 2 example targets.

Results compare executions starting from different receptor conformations:

(1) the bound (native) receptor conformation,

(2) the unbound (free) receptor conformation,

and (3) the unbound receptor conformation, including receptor backbone minimization.

The same native peptide starting conformation is used for all three simulations.

BENCHMARK DATASET

The dataset includes:

1CZY_bound - the bound (native) conformation. Receptor chain C, peptide E.

1CZY_ub - this is receptor 1CA4 chain C with peptide from 1CZY:E

1CZY_ub_bb - same as above but with backbone minimization protocl.

2FOJ_bound - the bound (native) conformation. Receptor chain A, peptide B.

2FOJ_ub - this is receptor 2FOJ chain A with peptide from 2FOJ:B

2FOJ_ub_bb - same as above but with backbone minimization protocl.

PROTOCOL

Rosetta FlexPepDock is a high-resolution peptide-protein docking protocol that is able to refine a coarse starting structure of a peptide-protein complex, to a near-native model of the interaction. The full degrees of freedom of the peptide are optimized (rigid body orientation, peptide backbone and side chains). Optionally, the receptor backbone can be minimized during optimization.

For more information, read:

1) Raveh, B., London, N. & Schueler-Furman, O.: Sub-angstrom modeling of complexes between flexible peptides and globular proteins. Proteins (2010). https://onlinelibrary.wiley.com/doi/full/10.1002/prot.22716, and

2) Alam, N. & Schueler-Furman, O. Modeling peptide-protein structure and binding using monte carlo sampling approaches: Rosetta flexpepdock and flexpepbind. in Methods in Molecular Biology (2017).

Running this benchmark takes approximately 70 CPU hours.

PERFORMANCE METRICS

A passing test means that at least 8 out of the top10-scoring points are below the interface RMSD cutoff. The cutoff was determined as the maximum of the top10-scoring points in revision 61451 from 2020-Oct-12. The interface RMSD is represented by the rmsBB_if tag in the score file.

KEY RESULTS

The default implementation of FlexPepDock can reliably refine peptide conformations to near-native resolution for starting structures that are up to 4-5 Angstrom away from the native conformation. An extended range can be obtained by including an ab initio search of the peptide backbone conformation (see Raveh et al. PLoSOne 2011), or by coupling the FlexPepDock refinement step to a low-resolution fast rigid body search using other approaches (e.g. FFT, as implemented in PIPER-FlexPepDock, Alam PlosCB 2017).

DEFINITIONS AND COMMENTS

We use the reweighted_score to rank different models: this score term is the sum of total score, interface score and peptide score, providing more weight to the energy terms contributed by the peptide, compared to the energy of the full complex. Alternatively, interface score (I_Sc) may be used. Both terms will reduce the influence of possible conformational changes far away from the binding site that introduce noise.

LIMITATIONS

The protocol needs an approximate starting conformation of the peptide. This starting structure may be obtained from structures of homologous complexes, or from low-resolution docking protocols.

REVISION

revision:61592

test_id: 687686

status: passed

Scientific test: fragments_picking

FAILURES

None

RESULTS

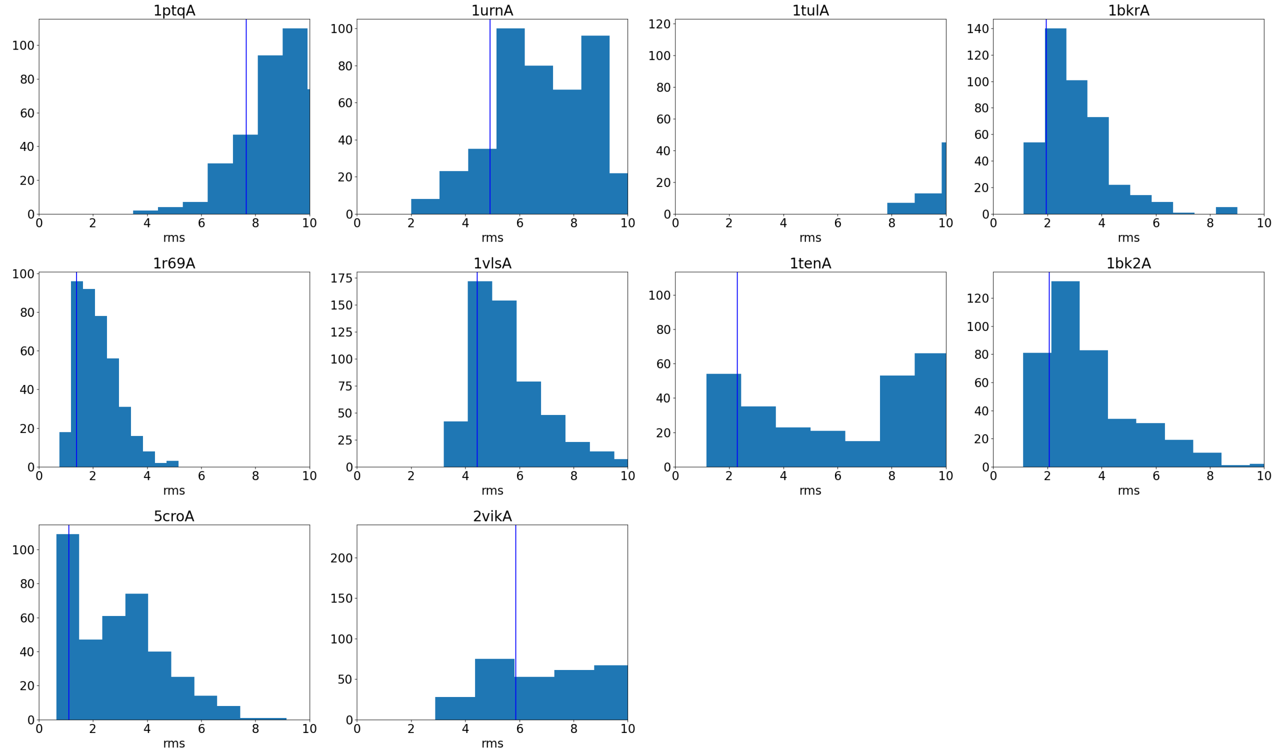

AUTHOR AND DATE

The test was set up by Justyna Krys (juchxd@gmail.com) in Dominik Gront's (dgront@gmail.com) lab on June 2019.

PURPOSE OF THE TEST

We test if the FragmentPicker picks fragments correctly. After running the FragmentPicker we run the ab-initio protocol with the RMSD to test the quality of the fragments.

BENCHMARK DATASET

The set has 10 proteins and contains alpha, beta and alpha-beta proteins with various lengths.

We pick some proteins from the BENCHMARK62 database.

Input files for the FragmentPiker are: pdb file, sequence profile in .profile format from blast, secondary structure predictions for proteins from three predictors (psipred, porter and rdb(=SAM)), homolog file to excluded them from the VALL database, scoring-multirama.wgths, quota.def, .vall database.

Input files for the ab initio protocol are the pdb file, ideal-pdb which is referenced to calculate rmsd, fragment files generated in the first step of test.

PROTOCOL

The FragmentPicker picks structural fragments for single proteins from a database according to sequence and structure (from structure predictors') similarity. Then fragments are used in the ab initio protocol to build a model of protein structure.

The FragmentPicker protocol is described in (Gront D, Kulp DW, Vernon RM, Strauss CEM, Baker D (2011) Generalized Fragment Picking in Rosetta: Design, Protocols and Applications. PLOS ONE 6(8))

This protocol requires about 2,000 CPU hours.

PERFORMANCE METRICS

We use the rmsd between the native and decoys calculated in ab initio simulations. The score function contains the rmsd to the native as one of the scoring terms. Such a simulation therefore reflects how well the native can be reconstructed by the given set of fragments.

10th percentile of rmsd should be lower then cutoff to pass the test. If its higher then the test will fail.

The ab initio protocol was run and statistics from 10,000 decoys where made to define proper cutoffs.

KEY RESULTS

The baseline is based on fragments published in (Gront D, Kulp DW, Vernon RM, Strauss CEM, Baker D (2011) Generalized Fragment Picking in Rosetta: Design, Protocols and Applications. PLOS ONE 6(8))

There are no outliers in the dataset.

DEFINITIONS AND COMMENTS

The test is sensitive to input files - sequence profile and secondary structure predicions.

LIMITATIONS

The benchmark assumes the input files remains constant. The benchmark doesn't test the impact of secondary structure predictions, sequence databases and psiblast parameters.

One could include the full fragment picking procedure in the test - this would require psiblast runs. This is actually part of another test called make_fragments, set up by Dan Farrell.

One could include more and more varied protein cases in the benchmark.

REVISION

revision:61611

test_id: 691563

status: passed

Scientific test: glycan_dock

FAILURES

5OYE

RESULTS

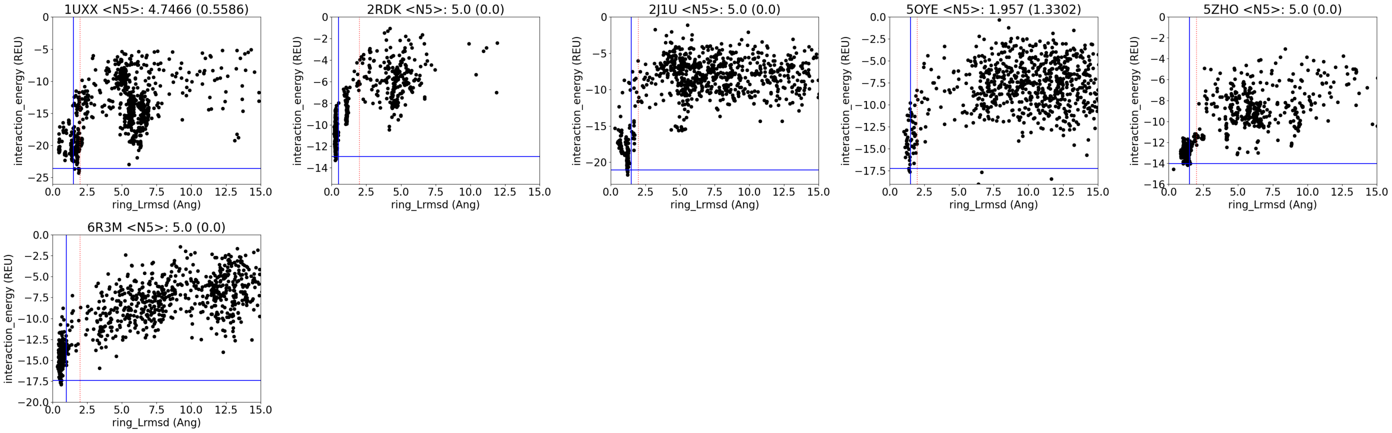

AUTHOR AND DATE

Morgan L. Nance (morganlnance@gmail.com; June 2021)

Relevant publication

DOI: 10.1021/acs.jpcb.1c00910

Development and Evaluation of GlycanDock: A Protein–Glycoligand Docking Refinement Algorithm in Rosetta

Morgan L. Nance, Jason W. Labonte, Jared Adolf-Bryfogle, and Jeffrey J. Gray

The Journal of Physical Chemistry B 2021 125 (25), 6807-6820

-When "benchmark paper" is mentioned, it is referring to this publication

PURPOSE OF THE TEST

This test ensures that the GlycanDock protein-glycoligand docking refinement algorithm performs, at a minimum, on par with its observed performance during its original benchmarking. This test should catch if GlycanDock performance falls on a selection of 6 targets from the benchmark set.

BENCHMARK DATASET

This test uses 6 of the original 65 unbound protein-glycoligand targets from the benchmark paper. The unbound protein targets (opposed to the bound protein-glycoligand crystals) are used in this test to provide a more accurate measure of GlycanDock performance in realistic docking refinement scenarios.

Input protein-glycoligand structures were all originally used as input for the GlycanDock benchmark paper. The constraints were also used in the benchmark (though here, for simplicity, each constraint used for each

carbohydrate unit of the glycoligand are combined into a single .cst file). The reference native structures were also those used in the benchmark paper.

The unbound protein structure was aligned onto the bound protein structure using PyMOL. The coordinates for the glycoligand from the bound structure and the coordinates of the unbound protein structure were kept (to serve as the "native" unbound protein-glycoligand structure). This structure was then pre-packed. Finally, the glycoligand was randomly but systematically perturbed in rigid-body and glycosidic torsion angle space to reach the desired 7 +/- 0.1 Ang initial ring-RMSD.

The following 6 targets were selected for scientific benchmarking. See key results for detailed information on how the 6 targets performed in the benchmark paper.

*1UXX (unbound protein 1GMM)

-- CtCBM6 - xylopentaose

-- Reason for selection: Passed bootstrap_N5 >= 3 cutoff in benchmark but with relatively high sstandard deviation. CBM target represents a common protein of interest in glycobiology.

*2RDK (unbound protein 2Z21)

-- Cyanovirin - N-dimannose

-- Reason for selection: Performed perfectly in benchmark. Cyanovirin is an anti-viral glyco-binder of scientific interest.

*2J1U (unbound protein 2J1R)

-- Strep lectin - Blood Group A-tetrasaccharide

-- Reason for selection: Strong success with a glycoligand that is an important blood group antigen and is branched

*5OYE (unbound protein 5OYC)

-- CjGH5 - xyloglucan

-- Reason for selection: A difficult target (a hexasaccharide with two exocyclic branch points) that showed promise (bootstrap_N5 close to 3). This target serves as a potential indicator if Rosetta sampling and/or scoring improves

*5ZHO (unbound protein 5ZHG)

-- G4P RVC VP8* - A histo-blood group

-- Reason for selection: Strong success with a glycoligand that is an important blood group antigen and is branched. The human group C rotavirus VP8* protein is also an important target

*6R3M (unbound protein 1V0A)

-- CtCBM11 - beta-1,3-1,4-mixed-linked tetrasaccharide

-- Reason for selection: Just above the bootstrap_N5 cutoff for docking success, but with significant standard deviation. Glycoligand is of mixed linkage and serves as an interesting target for docking and glycosidic torsion angle sampling

PROTOCOL

The default GlycanDock docking refinement algorithm is applied to each input structure. Briefly, a small, random rigid-body rotation and perturbation is applied to the glycoligand's center-of-mass, and a small, random uniform perturbation is applied to each glycosidic torsion angle. The Metropolis criterion is not applied. Then, inner cycles of rigid-body and glycosidic torsion angle sampling occur while outer cycles control ramping the fa_atr and fa_rep score terms. The structure is minimized after every other sampling move, whereas packing occurs after every sampling move.

See the Methods section and Supplemental of the benchmark paper for more information on the GlycanDock algorithm

For the code, see src/protocols/glycan_docking/*

Approximately 1100 CPU hours

1UXX: 495 +/- 35 sec per model

2RDK: 358 +/- 19 sec per model

2J1U: 608 +/- 49 sec per model

5OYE: 1204 +/- 199 sec per model

5ZHO: 579 +/- 41 sec per model

6R3M: 642 +/- 153 sec per model

1000 models per target

((avg seconds * nstruct) * ...) / (60 sec * 60 minutes to convert to hours)

--> ((495*1000)+(358*1000)+(608*1000)+(1204*1000)+(579*1000)+(642*1000))/3600.

PERFORMANCE METRICS

We evaluate performance using the average N5 after bootstrap case resampling (bootstrap_N5). N5 means the number of near-native models (sub 2 Ang ring-RMSD) ranked within the 5-top-scoring. Ideal N5 is 5, making ideal bootstrap_N5 = 5.0. An N5 or bootstrap_N5 of 0 means that NO near-native models were ranked within the top-5.

Bootstrap case resampling is performed as follows. For a given target, 5000 sets of resampled models are generated by randomly selecting 1000 models with replacement from the original set of models. The subset of models are then ranked by score (here, interaction_energy), and the N5 is determined. This process gives us 5000 N5 values per target, the average of which is our bootstrap_N5 value.

bootstrap_N5 >= 3 is considered a docking success. This was the performance cutoff used in the benchmark paper. For this scientific test, the cutoff for bootstrap_N5 differs per target.

This test also tracks if at least one of the 5-top-scoring models has a ring-RMSD (ring_Lrmsd) that is below the specified cutoff per target. These ring-RMSD values also come from the benchmark paper. However, the general cutoff for a near-native model is < 2.0 Ang ring-RMSD. The ring-RMSD metric check is not a pass/fail metric. ring-RMSD (ring_Lrmsd) is defined as the root-mean-squared deviation of all carbohydrate ring atoms of the glycoligand compared to the native/reference structure.

The following must be true for this scientific test to pass:

*1UXX: bootstrap_N5 >= 4

*2RDK: bootstrap_N5 >= 5

*2J1U: bootstrap_N5 >= 4

*5OYE: bootstrap_N5 >= 2 (Note: benchmark paper defined success as bootstrap_N5 >= 3)

*5ZHO: bootstrap_N5 >= 4

*6R3M: bootstrap_N5 >= 3 (Note: possibly too stringent given its bootstrap_N5 std dev (see above))

The cutoffs chosen are based on the target's performance in the benchmark.

The x-axis is the ring-RMSD (ring_Lrmsd) in Angstroms. The y-axis is the interaction energy (REU). Interaction energy is calculated by taking the total_score of the complex and subtracting off the total_score of the unbound complex (i.e. bound energy - unbound energy). One simulates the unbound complex by translating the glycoligand 1000 Ang away from the protein receptor.

The blue horizontal line marks the interaction_energy of the top-5 scoring model (i.e. the interaction_energy cutoff for the 5-top-scoring models). It is NOT a pass/fail metric.

The blue vertial line marks the ring_Lrmsd cutoff for that particular target (ranges from 0.5 to 1.5). It is NOT a pass/fail metric.

The red dashed vertical line marks the 2 Ang ring_Lrmsd cutoff for a model to be considered near-native (a general and universal model quality cutoff for protein-glycoligand complexes). It is involved in the calculation of bootstrap_N5, which is a pass/fail metric.

The title of each plot states the target ID, the bootstrap_N5 value and the standard deviation in parentheses.

KEY RESULTS

Results are compared to past performance as measured in the initial benchmarking of GlycanDock. Measuring performance requires the experimental structure of the protein-glycoligand complex, which is used here.

The following docking results are from the GlycanDock benchmark paper (NOTE, where nstruct was 2k; see the unbound benchmark set at initial ring-RMSD 7 +/- 0.1 Ang). Additional result details to look for in this scientific test are also given (beyond ensuring that all specified bootstrap_N5 cutoffs per target are hit/surpassed):

*1UXX (unbound protein 1GMM)

-- CtCBM6 - xylopentaose

-- # near-natives: 73

-- N5: 5

-- bootstrap_N5: 4.023

-- bootstrap_N5 std: 0.929

-- Future improvement desired: Better bootstrap_N5 discrimination (i.e. closer to 5)

-- What to look for: a good funnel toward near-native models with a secondary but less favorable funnel toward non-native-like models around 6 Ang

*2RDK (unbound protein 2Z21)

-- Cyanovirin - N-dimannose

-- # near-natives: 70

-- N5: 5

-- bootstrap_N5: 5.000

-- bootstrap_N5 std: 0.000

-- Future improvement desired: More near-native models generated

-- What to look for: A very strong funnel toward sub-Angstrom models

*2J1U (unbound protein 2J1R)

-- Strep lectin - Blood Group A-tetrasaccharide

-- # near-natives: 31

-- N5: 5

-- bootstrap_N5: 4.658

-- bootstrap_N5 std: 0.693

-- Future improvement desired: More near-native models

-- What to look for: A strong funnel toward near-native models, but with a comparatively large cloud of non-native-like models scored worse than the native-like ones

*5OYE (unbound protein 5OYC)

-- CjGH5 - xyloglucan

-- # near-natives: 13

-- N5: 5

-- bootstrap_N5: 2.665

-- bootstrap_N5 std: 1.357

-- Future improvement desired: Better bootstrap_N5 discrimination

-- What to look for: A weak funnel toward near-native models, with multiple non-native-like false positive models. This is due to the two carbohydrate units that are exocyclically connected to the glycoligand chain having multiple low-energy conformations

*5ZHO (unbound protein 5ZHG)

-- G4P RVC VP8* - A histo-blood group

-- # near-natives: 57

-- N5: 5

-- bootstrap_N5: 4.746

-- bootstrap_N5 std: 0.528

-- Future improvement desired: More near-native models

-- What to look for: A weak funnel toward near-native models, but with many near-native models generated. Potentially will see non-native-like models up to 12 Ang scored as favorably as the native-like models

*6R3M (unbound protein 1V0A)

-- CtCBM11 - beta-1,3-1,4-mixed-linked tetrasaccharide

-- # near-natives: 59

-- N5: 5

-- bootstrap_N5: 3.322

-- bootstrap_N5 std: 1.189

-- Future improvement desired: Better bootstrap_N5 discrimination

-- What to look for: A funnel toward near-native models, but with a handful of potential false positives within 2-4 Ang ring-RMSD

Target 5OYE has a bootstrap_N5 cutoff of >= 2 (instead of >= 3) in this scientific test in order to catch a single case where GlycanDock performance on more difficult targets increases, if it does.

DEFINITIONS AND COMMENTS

Nothing will work without the -include_sugars flag!

The input structures used here have already been run through Rosetta once (scored and a .pdb file dumped) so that the carbohydrate residues are converted into Rosetta formatting. This process requires flags such as -include_sugars -lock_rings -alternate_3_letter_codes pdb_sugar -maintain_links.

If the sugar has a branch point, there MUST be a TER record that separates the branched residue from the rest of the carbohydrate chain. See 2J1U and 5OYE for an example.

I used the ref2015 scorefunction for benchmarking only. Note that the GlycanDockProtocol.cc file sets fa_intra_rep_nonprotein to the weight of fa_rep (generally 0.55) and sugar_bb to 0.5 (but could be overrided if user provides different weights in the flags file).

-lock_rings if set to True by default, meaning that minimization should NOT alter carbohydrate nu angles.

Strengths of the benchmark are that (1) the protein structures used are the unbound coordinates (more realistic) and (2) the initial conformation of the glycoligand is deviated up to 7 Ang ring-RMSD (also more realistic)

LIMITATIONS

Only 6 of the original 65 targets are tested here, with a bias toward CBM/lectin protein receptors.

Notably, in the benchmark paper, each target for each initial ring-RMSD bin had 10 unique starting conformations (e.g. initial ring-RMSD bin 7 +/- 0.1 Ang target 1ABC input-1.pdb, input-2.pdb, ..., input-10.pdb). Here in this scientific test, only one input conformation is used as input for each target. The single input structure chosen per target that resulted in the best performance in the benchmark paper was chosen as the representative input structure per target here in this sci test. Therefore, the results per target are biased toward out-performing the results seen in the benchmark paper (at least in terms of # of near-native models generated).

This scientific test uses nstruct=1000 for time consideration instead of the nstruct=2000 used in the benchmark paper.

More targets from the benchmark that represent other protein receptors of interest, such as an antibody

More computing power would allow for more models generated, preferably up to nstruct=2000 to match the benchmark

Running a parallel of this benchmark using the beta_nov16 scorefunction to see performance differences

REVISION

revision: 61711

test_id: 713286

Scientific test: glycan_structure_prediction

FAILURES

RESULTS

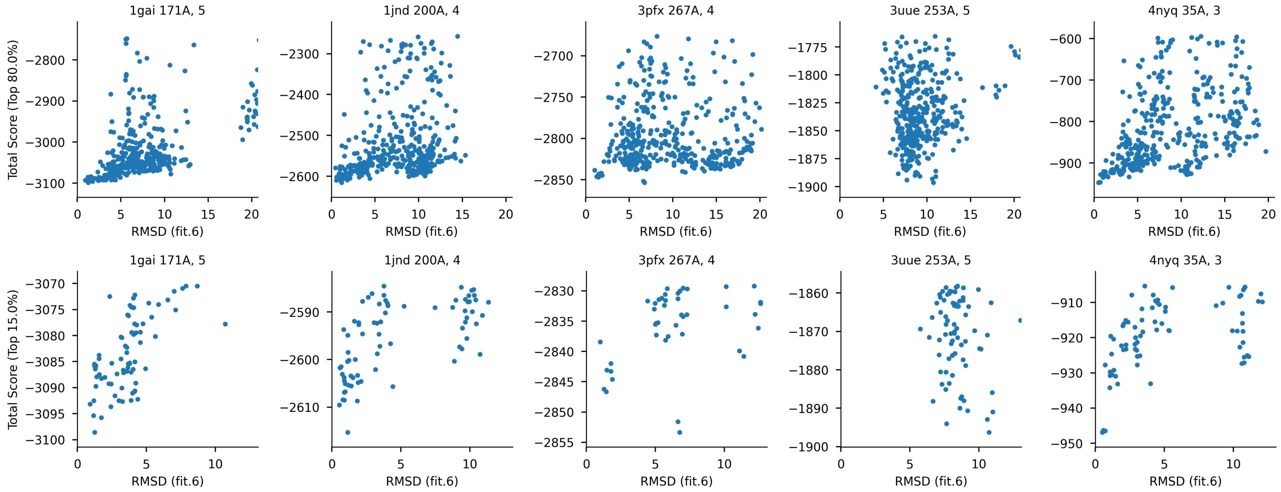

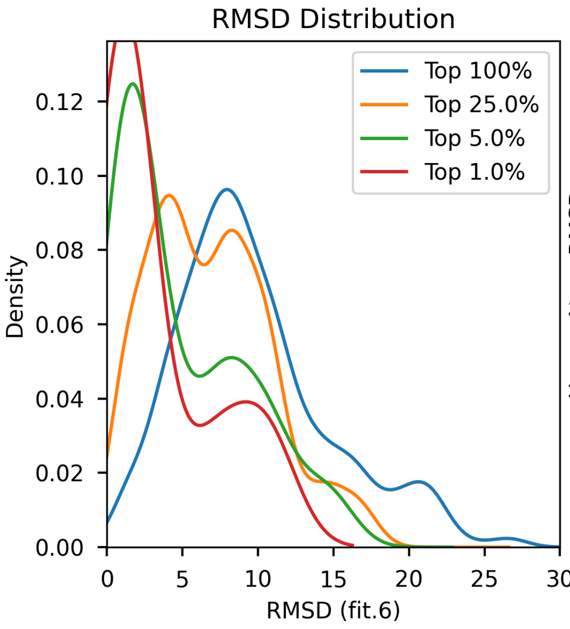

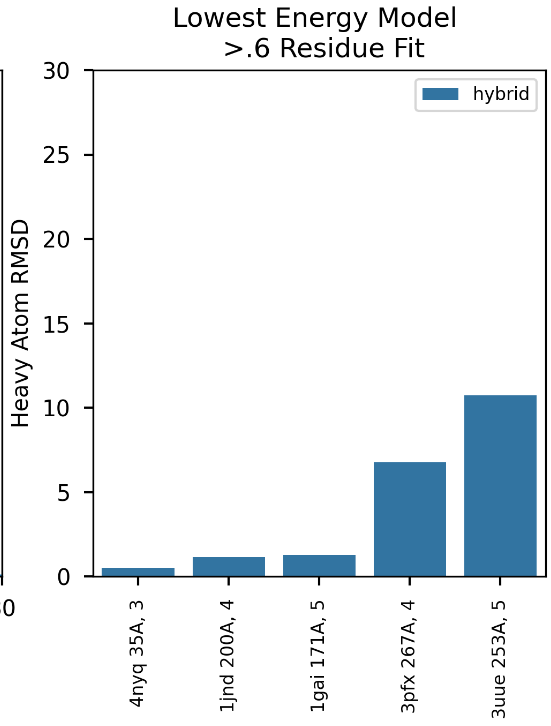

AUTHOR

Jared Adolf-Bryfogle (jadolfbr@gmail.com) 9/5/2019

PURPOSE OF THE TEST

This test ensures that the GlycanTreeModeler retains its scientific performance. This application is intended to predict the structures of glycan trees on a protein surface

What does the benchmark test and why?

The benchmark tests 5 input structures from our 26 total used for (Adolf-Bryfogle, Labonte et. al, 2021).

The benchmark uses Symmetry to represent the original crystal environment of the inputs, as well as crystal densities.

Densities are used to determine the fit of each residue into the actual density - which is ultimately used to calculate RMSDs.

The XML script includes a number of SimpleMetrics that we use to determine performance.

Currently, the test is setup in a way to determine sampling of near-native structures.

The plots help to determine the overall performance of the benchmark.

BENCHMARK DATASET

How many proteins are in the set?

- Five proteins, each test modeling a single glycan tree. These all had density under 100 mb, so we can actually have them in the repository.

What dataset are you using? Is it published? If yes, please add a citation.

- The dataset will be published in the paper (Adolf-Bryfogle, Labonte et. al 2021).

What are the input files? How were the they created?

- The inputs are refined PDBs. Each input was relaxed in parallel for 10 structures a piece.

- Relax was done in the presence of the actual crystal density built from phenix.maps, using the fast_elec_dens score term.

PROTOCOL

State and briefly describe the protocol.

The protocol starts with randomizing all glycan backbone torsions and then modeling the glycan tree from the roots out to the leaves in layers.

Besides layer 1, all other residues start as virtual - and as the tree is built up, these residues are un-virtualized.

A layer is defined as the number of residues to the root, and this allows us to model the correct residues together.

Each build cycle has a GlycanSampler run internally, which is essentially a WeightedSampler made up of different moves.

These moves consist of:

- random purturbations (of small, medium, large moves)

- packing of glycans and protein residue neighbors

- conformer sampling based on a new bioinformatic analysis of the PDB

- sampling based on the sugar_bb energy term as probabilities

- minimization on a random residue and it's glycan children

- shear moves

Is there a publication that describes the protocol?

Some of the original sampling can be found in the RosettaCarbohydrates publication, listed in citations.

The core of the protocol is currently being benchmarked, with a paper to come in a few months.

How many CPU hours does this benchmark take approximately?

Estimates run at about 950 CPU hours.

PERFORMANCE METRICS

What are the performance metrics used and why were they chosen?

Except for 3UUE, which is not considered for a pass/fail, the following must be true for this test to pass:

- 1JND and 4NYQ must have at least one model < 1.0 A

- 1GAI and 3PFX must have at least one model < 5.0 A

These were determined based on a a similar benchmark for the paper. If we had more computational power, these cutoffs would be much more rigorous. 3UUE has the least rigorous cutoffs and lacks a good score funnel with REF2015

How do you define a pass/fail for this test?

Failure of any of the above.

How were any cutoffs defined?

Arbitrarily, like so much else in Rosetta. These are based on the performance of Rosetta in predicting the crystal structure of these proteins during spring 2019.

KEY RESULTS

What is the baseline to compare things to - experimental data or a previous Rosetta protocol?

Past iterations of this test.

Describe outliers in the dataset.

Note that 3uue is the worst performing glycan, and its cutoffs are not high. It is the only one that does not have a good funnel.

DEFINITIONS AND COMMENTS

State anything you think is important for someone else to replicate your results.

N/A.

LIMITATIONS

What are the limitations of the benchmark? Consider dataset, quality measures, protocol etc.

Computational power. 1k nstruct is usually the bare minimum (for these benchmarks, we have had to reduce that to 500 as well).

For a particular input, we recommend 5-10k.

How could the benchmark be improved?

More processing power. Cutoffs for score vs RMSD

What goals should be hit to make this a "good" benchmark?

Improved cutoffs once the full benchmarking (for the main glycan modeling paper) is complete.

Increased nstruct once new nodes come online.

REVISION

revision:61603

test_id: 692306

status: passed

Scientific test: ligand_docking

SAMPLING FAILURES

betanov16: 0

ligand: 1
4W7T
ref2015: 0

talaris: 0

SCORING FAILURES

betanov16: 27
3FKL 4QMQ 2VTS 3U5K 4BQH 5D7C 5AOJ 3K5V 4CHN 5D7P 4CJF 5AM0 4A9N 4XT2 4CCU 4GB2 3PE2 5CTU 3FLY 3BLL 1ZZL 4KTN 4FPJ 4WUA 3UWK 4UAL 2BR1
ligand: 22
3FKL 4QMQ 2VTS 4MCC 5D7C 3K5V 5AM0 4CCU 3ZXZ 1EYQ 5CTU 3FLY 3BLL 4FA2 4J93 3P0Q 3TLL 4KTN 4FPJ 4WUA 3UWK 3ZO4
ref2015: 20
2VTS 4BQH 5D7C 3R5N 4CHN 5D7P 4XT2 3PE2 1EYQ 5CTU 3FLY 3BLL 1ZZL 4KTN 4FPJ 3UWK 3ZO4 4ZBQ 2BR1 3VRI
talaris: 26
3FKL 4QMQ 2VTS 4MCC 5D7C 3R5N 5AOJ 3K5V 5D7P 5AM0 4CCU 4GB2 3PE2 1EYQ 4UWC 5CTU 3FLY 3BLL 4F1L 4FA2 1ZZL 4KTN 4FPJ 3UWK 4I4F 2BR1

RESULTS

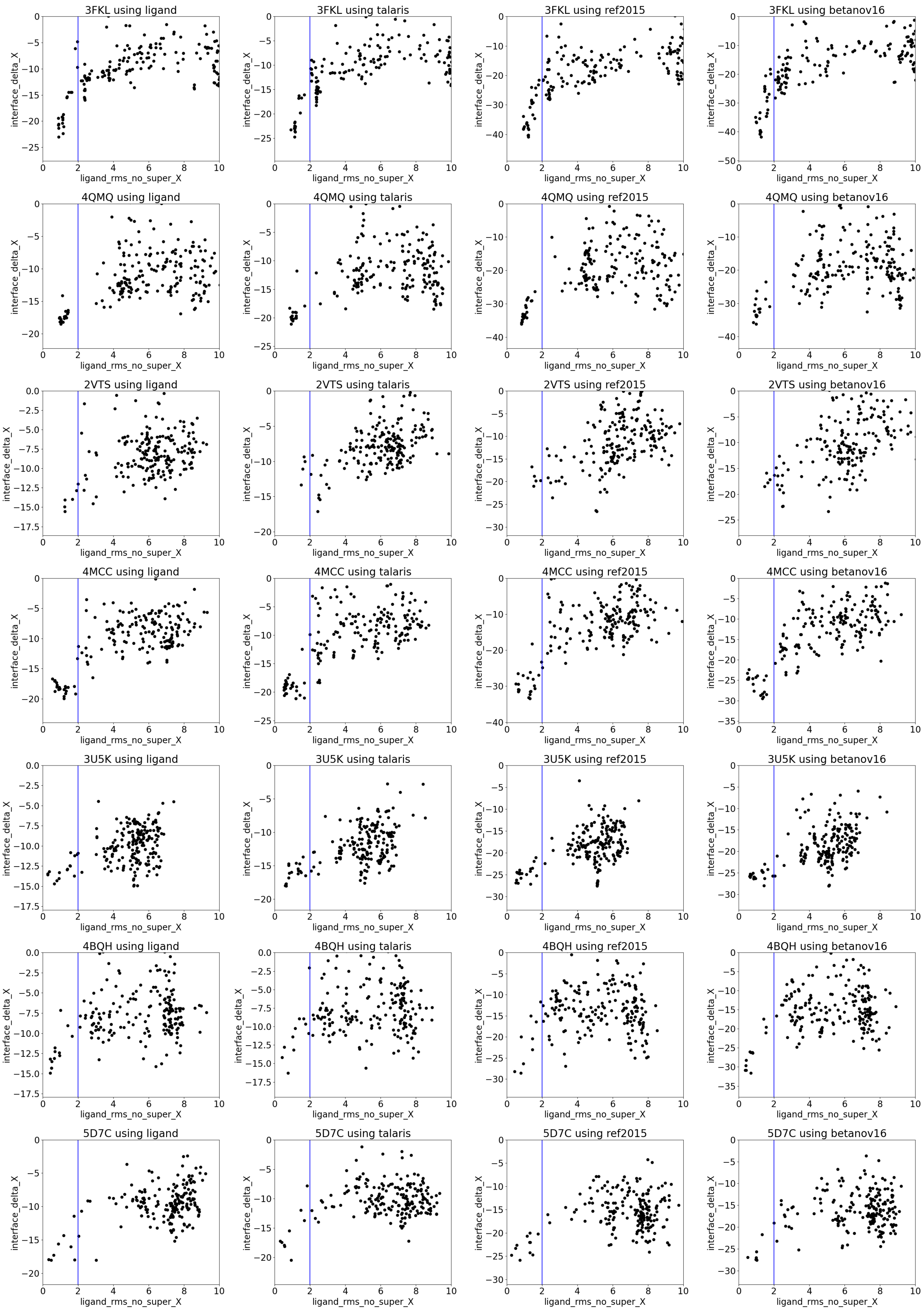
3FKL using ligand
3FKL using talaris
3FKL using ref2015
3FKL using betanov16
4QMQ using ligand
4QMQ using talaris
4QMQ using ref2015
4QMQ using betanov16
2VTS using ligand
2VTS using talaris
2VTS using ref2015
2VTS using betanov16
4MCC using ligand
4MCC using talaris
4MCC using ref2015
4MCC using betanov16
3U5K using ligand
3U5K using talaris
3U5K using ref2015
3U5K using betanov16
4BQH using ligand
4BQH using talaris
4BQH using ref2015
4BQH using betanov16
5D7C using ligand
5D7C using talaris
5D7C using ref2015
5D7C using betanov16
interface_delta_X
ligand_rms_no_super_X

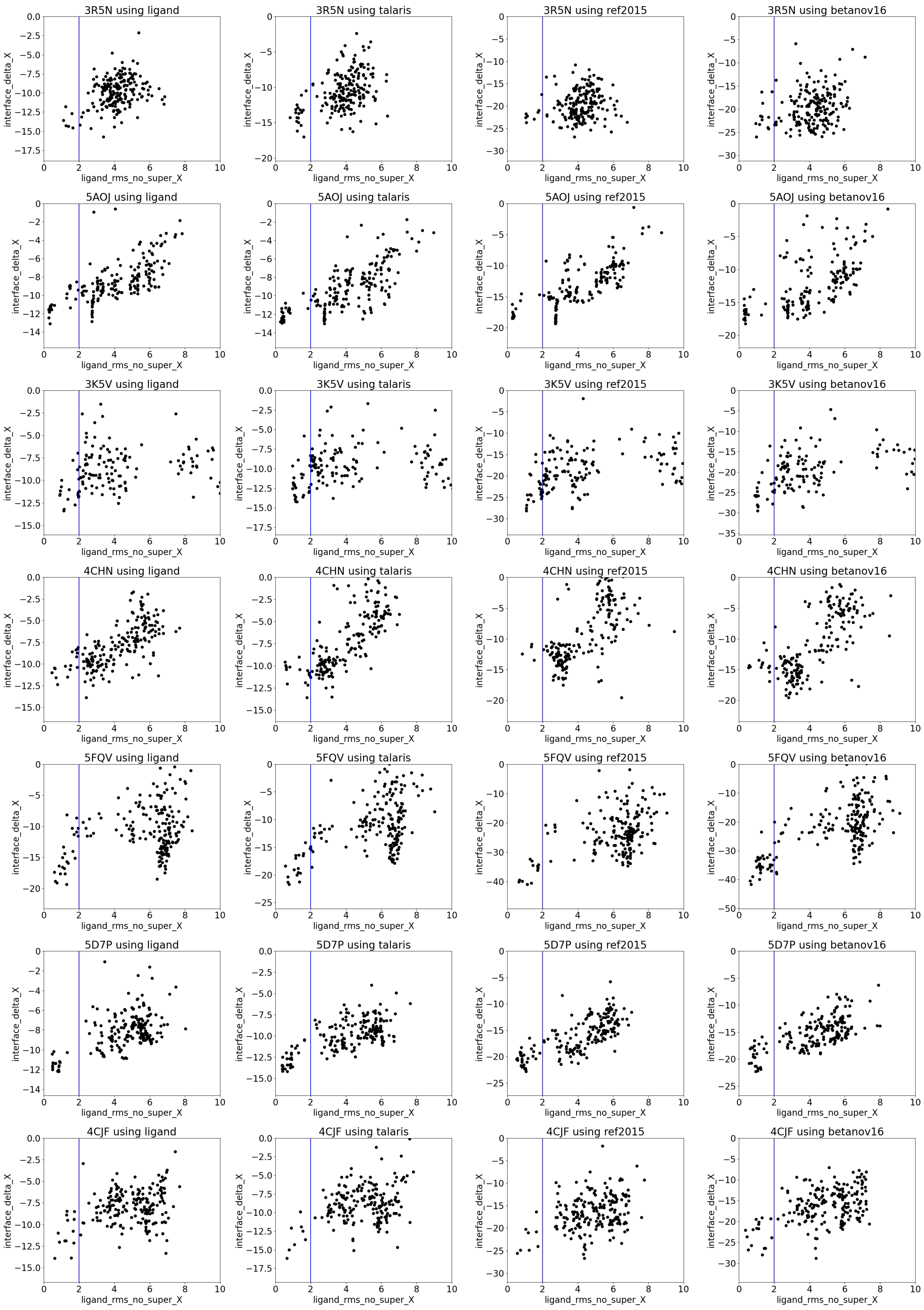
3R5N using ligand
3R5N using talaris
3R5N using ref2015
3R5N using betanov16
5AOJ using ligand
5AOJ using talaris
5AOJ using ref2015
5AOJ using betanov16
3K5V using ligand
3K5V using talaris
3K5V using ref2015
3K5V using betanov16
4CHN using ligand
4CHN using talaris
4CHN using ref2015
4CHN using betanov16
5FQV using ligand
5FQV using talaris
5FQV using ref2015
5FQV using betanov16
5D7P using ligand
5D7P using talaris
5D7P using ref2015
5D7P using betanov16
4CJF using ligand
4CJF using talaris
4CJF using ref2015
4CJF using betanov16
interface_delta_X
ligand_rms_no_super_X

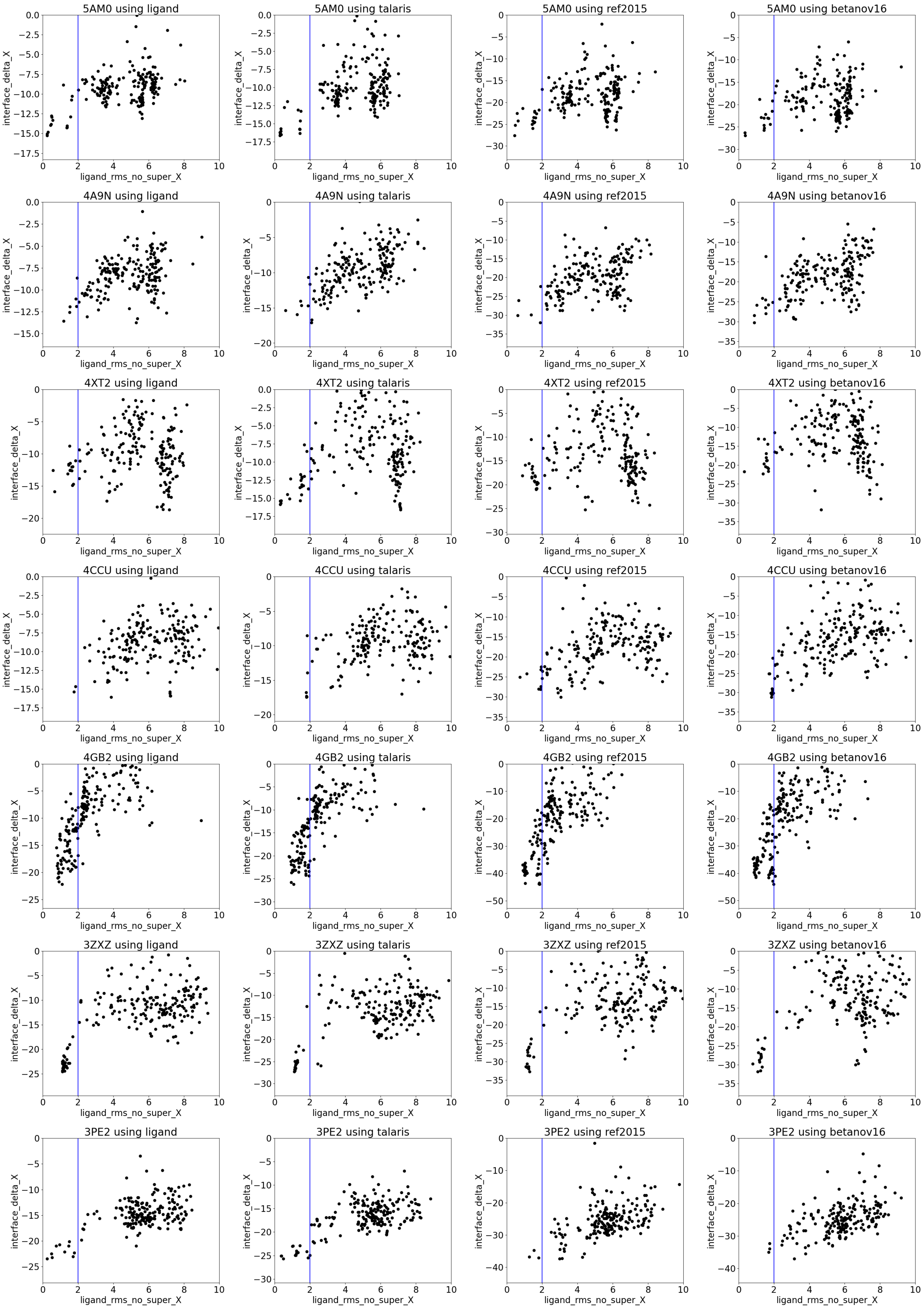
5AM0 using ligand
5AM0 using talaris
5AM0 using ref2015
5AM0 using betanov16
4A9N using ligand
4A9N using talaris
4A9N using ref2015
4A9N using betanov16
4XT2 using ligand
4XT2 using talaris
4XT2 using ref2015
4XT2 using betanov16
4CCU using ligand
4CCU using talaris
4CCU using ref2015
4CCU using betanov16
4GB2 using ligand
4GB2 using talaris
4GB2 using ref2015
4GB2 using betanov16
3ZXZ using ligand
3ZXZ using talaris
3ZXZ using ref2015
3ZXZ using betanov16
3PE2 using ligand
3PE2 using talaris
3PE2 using ref2015
3PE2 using betanov16
interface_delta_X
ligand_rms_no_super_X

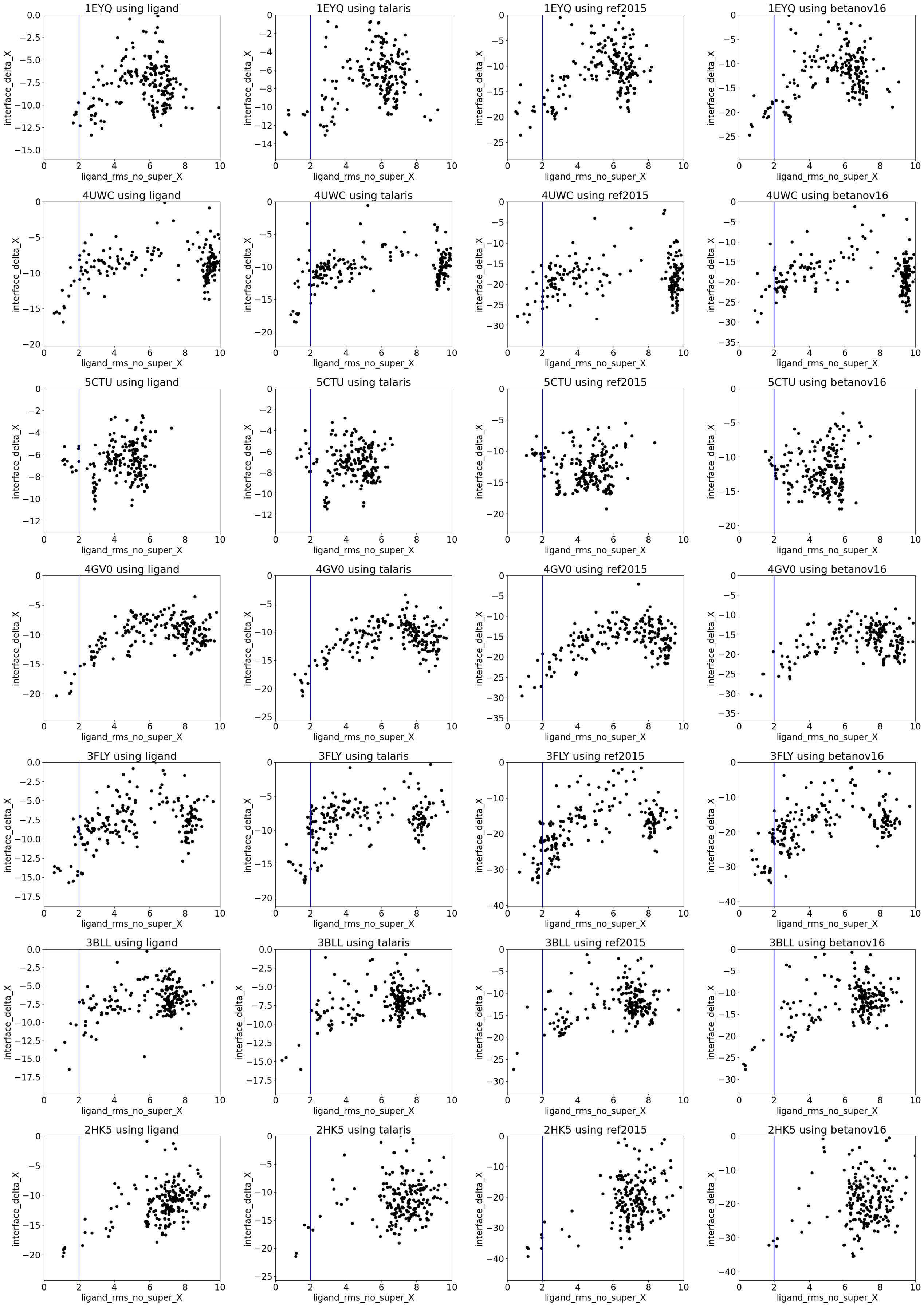

1EYQ using ligand
1EYQ using talaris
1EYQ using ref2015
1EYQ using betanov16
4UWC using ligand
4UWC using talaris
4UWC using ref2015
4UWC using betanov16
5CTU using ligand
5CTU using talaris
5CTU using ref2015
5CTU using betanov16
4GV0 using ligand
4GV0 using talaris
4GV0 using ref2015
4GV0 using betanov16
3FLY using ligand
3FLY using talaris
3FLY using ref2015
3FLY using betanov16
3BLL using ligand
3BLL using talaris
3BLL using ref2015
3BLL using betanov16
2HK5 using ligand
2HK5 using talaris
2HK5 using ref2015
2HK5 using betanov16
interface_delta_X
ligand_rms_no_super_X

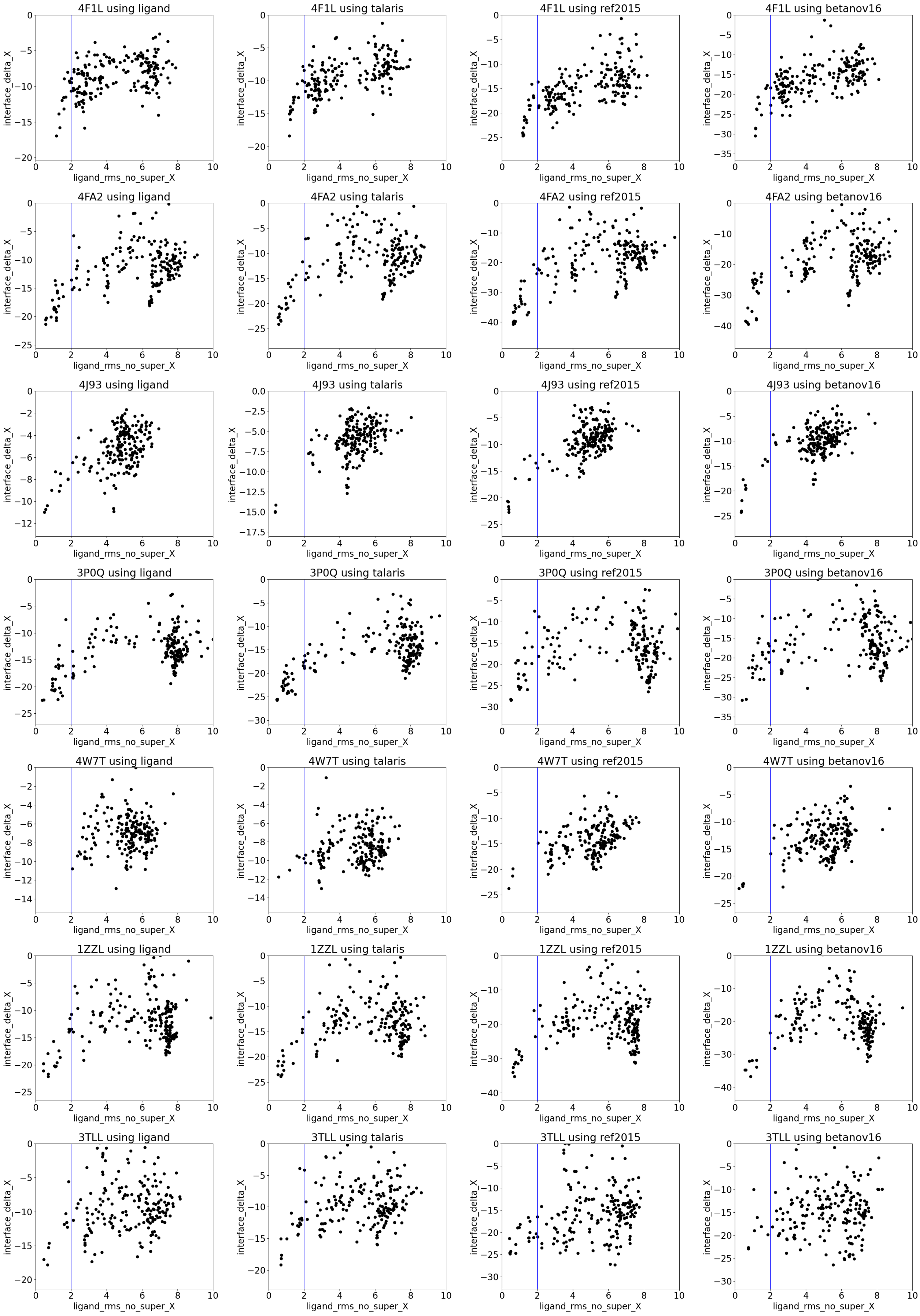
4F1L using ligand
4F1L using talaris
4F1L using ref2015
4F1L using betanov16
4FA2 using ligand
4FA2 using talaris
4FA2 using ref2015
4FA2 using betanov16
4J93 using ligand
4J93 using talaris
4J93 using ref2015
4J93 using betanov16
3P0Q using ligand
3P0Q using talaris
3P0Q using ref2015
3P0Q using betanov16
4W7T using ligand
4W7T using talaris
4W7T using ref2015
4W7T using betanov16
1ZZL using ligand
1ZZL using talaris
1ZZL using ref2015
1ZZL using betanov16
3TLL using ligand
3TLL using talaris
3TLL using ref2015
3TLL using betanov16
interface_delta_X
ligand_rms_no_super_X

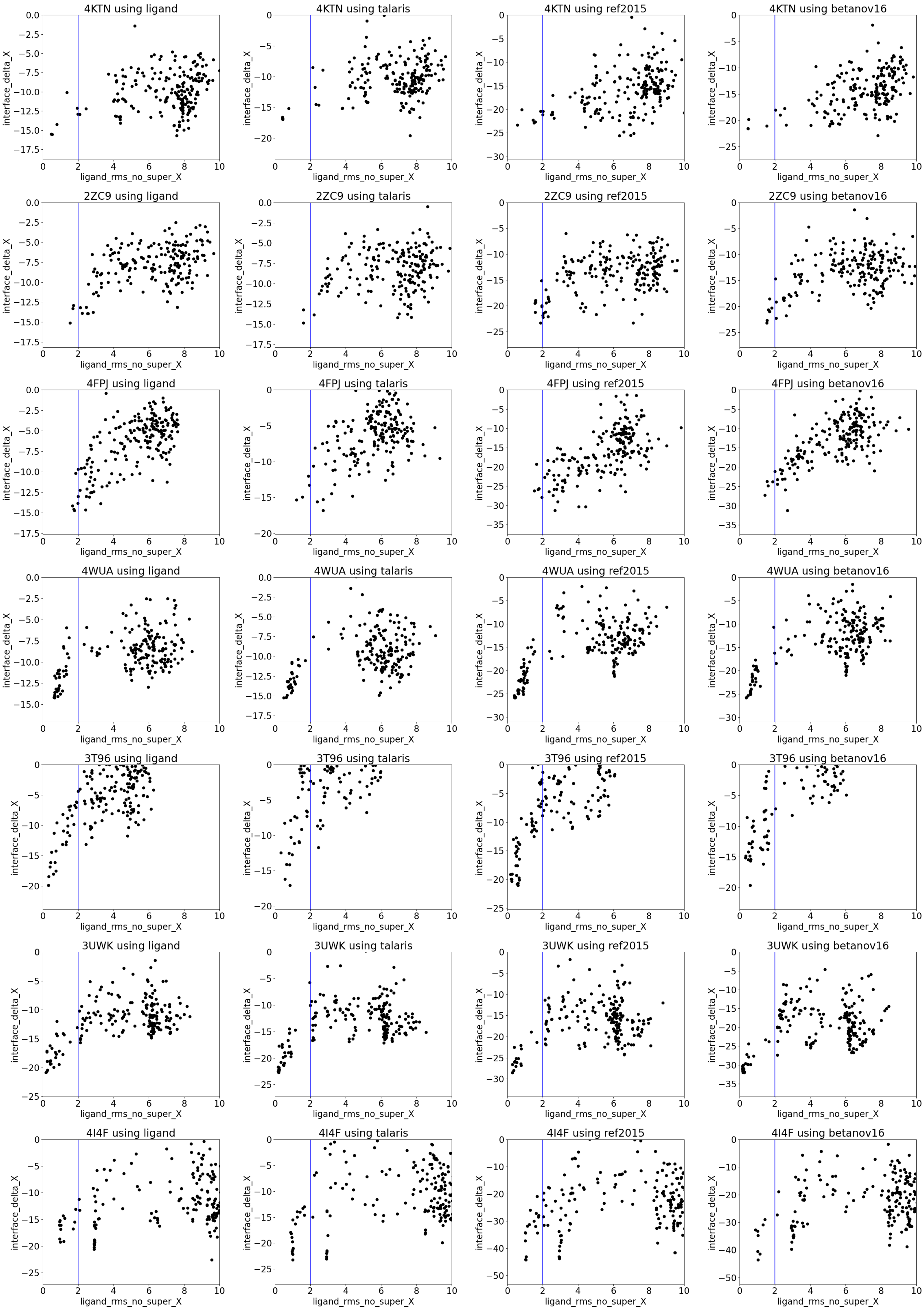
4KTN using ligand
4KTN using talaris
4KTN using ref2015
4KTN using betanov16
2ZC9 using ligand
2ZC9 using talaris
2ZC9 using ref2015
2ZC9 using betanov16
4FPJ using ligand
4FPJ using talaris
4FPJ using ref2015
4FPJ using betanov16
4WUA using ligand
4WUA using talaris
4WUA using ref2015
4WUA using betanov16
3T96 using ligand
3T96 using talaris
3T96 using ref2015
3T96 using betanov16
3UWK using ligand
3UWK using talaris
3UWK using ref2015
3UWK using betanov16
4I4F using ligand
4I4F using talaris
4I4F using ref2015
4I4F using betanov16
interface_delta_X
ligand_rms_no_super_X

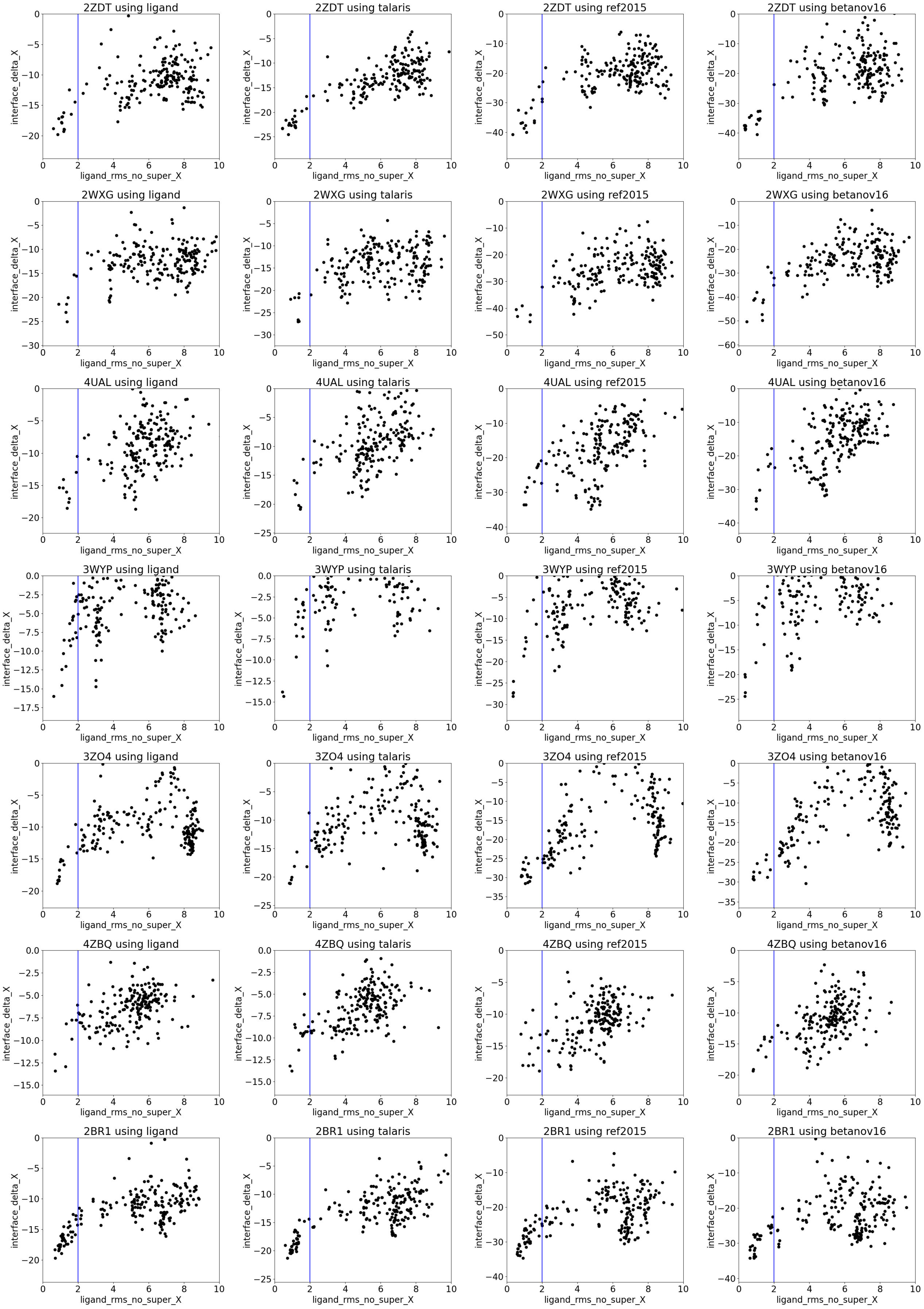
2ZDT using ligand
2ZDT using talaris
2ZDT using ref2015
2ZDT using betanov16
2WXG using ligand
2WXG using talaris
2WXG using ref2015
2WXG using betanov16
4UAL using ligand
4UAL using talaris
4UAL using ref2015
4UAL using betanov16
3WYP using ligand
3WYP using talaris
3WYP using ref2015
3WYP using betanov16
3ZO4 using ligand
3ZO4 using talaris
3ZO4 using ref2015
3ZO4 using betanov16
4ZBQ using ligand
4ZBQ using talaris
4ZBQ using ref2015
4ZBQ using betanov16
2BR1 using ligand
2BR1 using talaris
2BR1 using ref2015
2BR1 using betanov16
interface_delta_X
ligand_rms_no_super_X

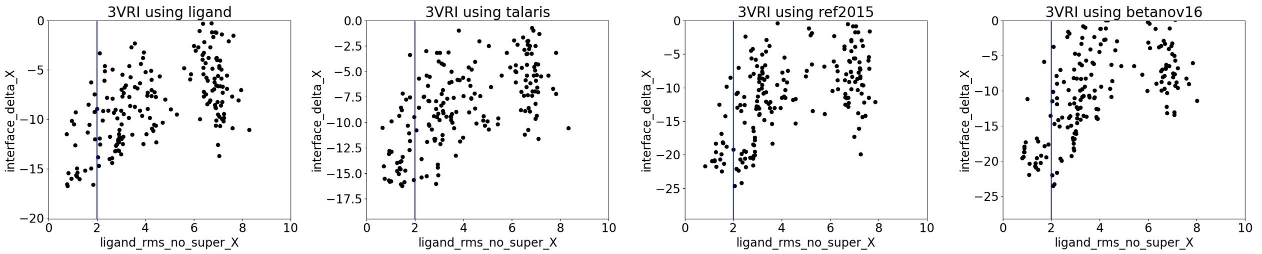

AUTHOR AND DATE

Adapted for the current benchmarking framework by Shannon Smith (shannon.t.smith.1@vanderbilt.edu; Meiler Lab), Feb 2019

PURPOSE OF THE TEST

This benchmark is meant to test how well we discriminate native small molecule binding orientations from decoys based on the interface score term by performing standard ligand docking experiments across 50 diverse protein-ligand complexes.

BENCHMARK DATASET

The dataset consists of 50 protein-ligand complexes extracted from the Diverse Platinum Dataset (Friedrich, N. et al. J. Chem. Inf. Model, 2017). In addition to the filters described thoroughly in Friedrich et al., the maximum resolution allowed was reduced to 2.0A, drug-like ligands were chosen using standard Lipinski Rules (Lipinski, C.A., et al. Advanced Drug Delivery Reviews, 1997), and visually inspected for unrealistic orientations and crystallographic artefact. This dataset was also filtered to eliminate cofactors or multiple ligands witin the binding site. The full list of protein-ligand complexes is given in the 1.submit.py file by PDB ID.

PROTOCOL

Structure preparation:

Proteins were extracted directly from the PDB according to their PDBIDs, underwent minimal cleaning to remove the ligand. Note that no prior relax or other structural manipulations were performed prior to beginning the docking run.

Ligand preparation:

Initial ligand structures were downloaded from the Protein Data Bank using the ligand ideal SDF. Note that this is not the same as the structure from the input cocrystal structure in order to minimize conformational bias towards a particular pre-generated pose. Ligand files were cleaned using OpenBabel (J. Cheminf. 2011, 3, 33), run through BCL Conformer Generator (Kothiwale, Meiler. J. Cheminform., 2015) and generated Rosetta-readable parameter files according to the following scripts:

BCL Conformer Generator

bcl.exe molecule:ConformerGenerator -rotamer_library cod -top_models 100 -ensemble_filenames NAME.sdf -conformers_single_file NAME_conformers.sdf -conformation_comparer 'Dihedral(method=Max)' 30 -max_iterations 1000

Rosetta Parameter File Generation:

/programs/x86_64-linux/rosetta/3.8/main/source/scripts/python/public/molfile_to_params.py -n $NAME -p $NAME --mm-as-virt --conformers-in-one-file NAME_conformers.sdf --chain X

This protocol currently uses 50 protein-ligand complexes, each containing the input file containing the cleaned protein + input ligand PDB (target_input.pdb), the native protein-ligand complex for RMSD calculations (target_native.pdb), the ligand params file (target_ligand.params) and the ligand conformer library file pointed to by the params file (target_ligand_conformers.pdb).

NOTE: the protein and ligand preparation steps were performed previously and are given as the input in the data/ directory. In other words, these steps are not performed each time this benchmark is run.

Each test takes ~8 CPU hours x 50 tests = ~400 CPU hours.

PERFORMANCE METRICS

**Need to run several tests to see what we think should be the cutoff.

The big question that this benchmark intends to test is how well we discriminate native versus non-native binding poses. A run is determined successful if there is an near-native (<2A) structure within the top 1percent of models based on the interface_delta_X score.

Sampling failure is defined as having no sub-2A output structures.

Scoring failure is defined as not having a sub-2A output structure within the top 10% ranked by interface score (this is a pretty large margin and may adjust accordingly).

KEY RESULTS

This benchmark is meant to be a longitudinal test to determine how changes in the scorefunction impact small-molecule docking performance.

Performance varies greatly across different test cases, so I am not sure how to go about grading overall performance. This also makes it difficult to define a binary pass/fail criteria to the entire benchmark.

DEFINITIONS AND COMMENTS

There is a bit of noise in the runs, so you may need to compare several runs to get better sense of how different scorefunctions compare.

LIMITATIONS

The run-to-run variablility is rather high, which might indicate that the benchmark could be improved by running several output structures for each input, rather than just the current one.

This benchmark currently utilizes the ligand scorefunction (an off-shoot of the score12 environment), as this is currently the best protocol for ligand docking in Rosetta. Could be updated for talaris2014, but performance has been shown to deteriorate in the newer scoring environments, notably ref2015 and later.

REVISION

revision:61531

test_id: 674791

status: passed

Scientific test: ligand_scoring_ranking

FAILURES

Number of failed cases out of 57: 2
4ty7 3o9i

RESULTS

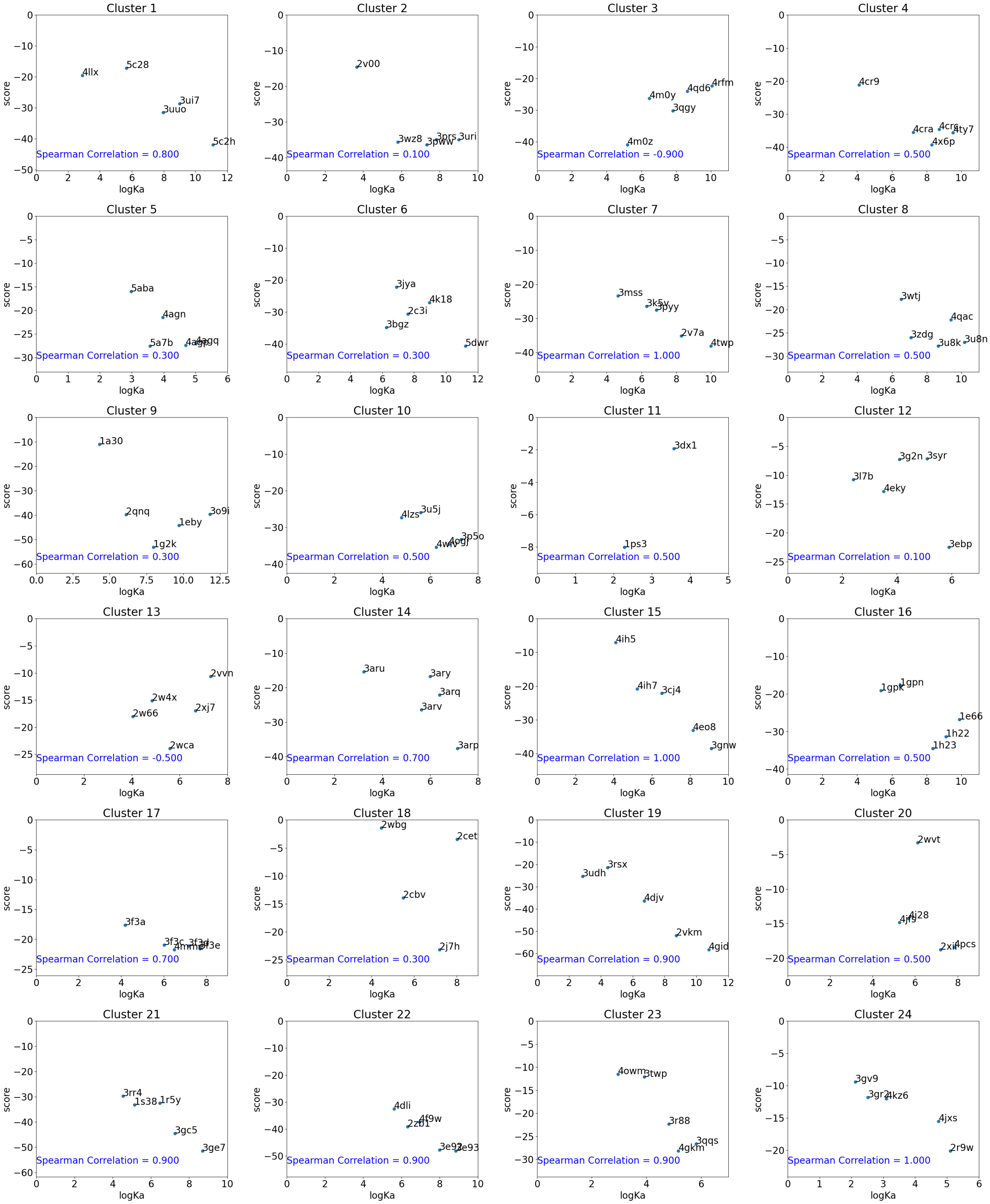

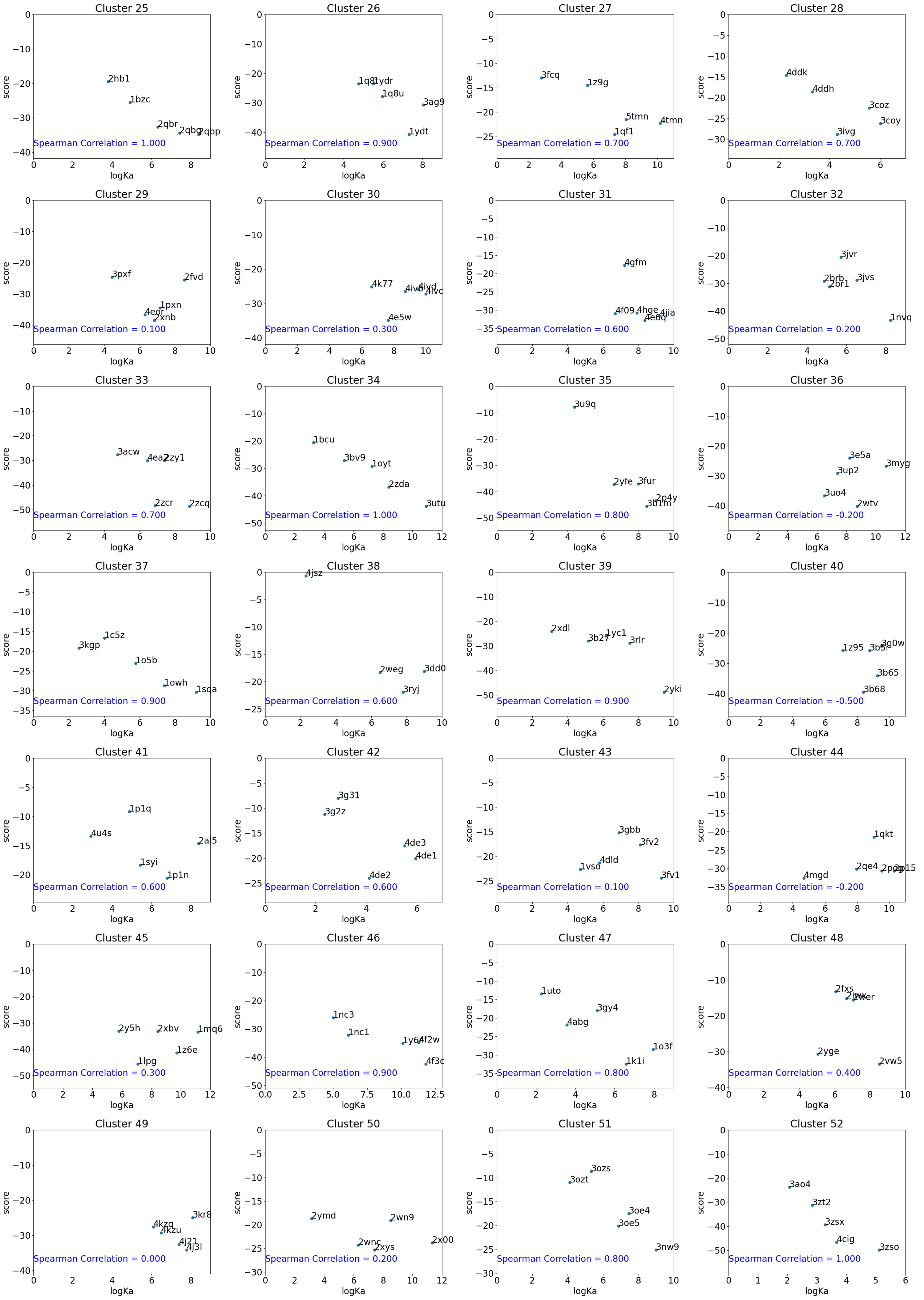

Cluster 25
2hb1
1bzc
2qbr
2qbq
2qbp
Spearman Correlation = 1.000
Cluster 26
1q8t
1ydr
1q8u
3ag9
1ydt
Spearman Correlation = 0.900
Cluster 27
3fcq
1z9g
5tmn
4tmn
1qf1
Spearman Correlation = 0.700
Cluster 28
4ddk
4ddh
3coz
3coy
3ivg
Spearman Correlation = 0.700
Cluster 29
3pxf
2fvd
1pxn
4eor
2xnb
Spearman Correlation = 0.100
Cluster 30
4k77
4ivb
4ivd
4ivc
4e5w
Spearman Correlation = 0.300
Cluster 31
4gfm
4f09
4hge
4jia
4e6q
Spearman Correlation = 0.600
Cluster 32
3jvr
2brb
3jvs
2br1
1nvq
Spearman Correlation = 0.200
Cluster 33
3acw
4ea2
2zy1
2zcr
2zcq
Spearman Correlation = 0.700
Cluster 34
1bcu
3bv9
1oyt
2zda
3utu
Spearman Correlation = 1.000
Cluster 35
3u9q
2yfe
3fur
2p4y
3b1m
Spearman Correlation = 0.800
Cluster 36
3e5a
3myg
3up2
3uo4
2wtv
Spearman Correlation = -0.200
Cluster 37
1c5z
3kgp
1o5b
1owh
1sqa
Spearman Correlation = 0.900
Cluster 38
4jsz
2weg
3dd0
3ryj
Spearman Correlation = 0.600
Cluster 39
2xdl
1yc1
3b27
3rlr
2yki
Spearman Correlation = 0.900
Cluster 40
3g0w
1z95
3b5r
3b65
3b68
Spearman Correlation = -0.500
Cluster 41
1p1q
4u4s
2al5
1syi
1p1n
Spearman Correlation = 0.600
Cluster 42
3g31
3g2z
4de3
4de1
4de2
Spearman Correlation = 0.600
Cluster 43
3gbb
3fv2
4dld
1vso
3fv1
Spearman Correlation = 0.100
Cluster 44
1qkt
2qe4
2pog
2p15
4mgd
Spearman Correlation = -0.200
Cluster 45
2y5h
2xbv
1mq6
1z6e
1lpg
Spearman Correlation = 0.300
Cluster 46
1nc3
1nc1
1y6q
4f2w
4f3c
Spearman Correlation = 0.900
Cluster 47
1uto
3gy4
4abg
1o3f
1k1i
Spearman Correlation = 0.800
Cluster 48
2fxs
2iwx
2wer
2yge
2vw5
Spearman Correlation = 0.400
Cluster 49
3kr8
4kzq
4kzu
4j21
4j3l
Spearman Correlation = 0.000
Cluster 50
2ymd
2wn9
2wnc
2xys
2x00
Spearman Correlation = 0.200
Cluster 51
3ozs
3ozt
3oe4
3oe5
3nw9
Spearman Correlation = 0.800
Cluster 52
3ao4
3zt2
3zsx
4cig
3zso
Spearman Correlation = 1.000
score
logKa

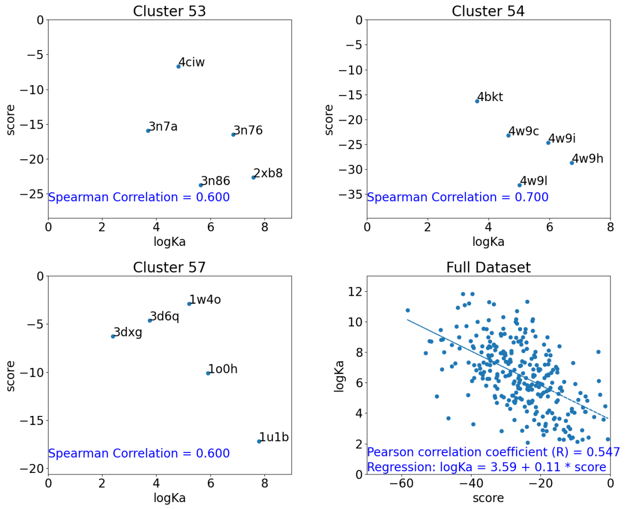

AUTHOR AND DATE

Shannon Smith, shannon.t.smith.1@vanderbilt.edu, PI: Jens Meiler, June 2019.

PURPOSE OF THE TEST

This benchmark tests our ability to correctly correlate experimental binding affinities and computed Rosetta protein-ligand interface scores. This test is meant to use the same dataset and success metrics as those in the Comparative Assessment of Scoring Functions 2016 (CASF). This is a diverse dataset that allows us to compare Rosetta protocols directly to other software suites on the same tests.

This benchmark is meant to be different than the previously-implemented "ligand_docking" benchmark, as this is only looking at the score function instead of performing a docking protocol with full sampling.

BENCHMARK DATASET

285 native, co-crystal structures of 57 protein-ligand complexes, i.e. 57 proteins (=targets) with varying ligands. The full list with associated logKa values and target information can be found in CoreSet.dat file.

CASF-2016 dataset: Comparative Assessment of Scoring Functions: The CASF-2016 Update. (J Chem Inf Model. 2019 Feb 25;59(2):895-913. doi: 10.1021/acs.jcim.8b00545.)

Input files are those directly taken from the CASF dataset. These are the co-crystal structures downloaded directly from the RCSB and ligand params files are generated using molfile_to_params.py

PROTOCOL

This protocol is implemented in RosettaScripts and takes native protein-ligand complexes, runs a minimization at the interface using the FinalMinimizer mover, then calculates the score using the InterfaceScoreCalculator mover.

Output scores for analysis are taken from the 'interface_delta_X' term without any additional filters/calculations.

A publication describing this benchmark and protocol is available at (Smith Shannon T, Meiler Jens: â€œAssessing multiple score functions in Rosetta for drug discoveryâ€, PlosOne, 2020, https://doi.org/10.1371/journal.pone.0240450)

This is a fast test. Only takes ~30 seconds max / structure * 285 ~ 2 hours.

PERFORMANCE METRICS

This test has two components: scoring and ranking. Scoring power refers to a score functions' ability to linearly correlate experimental binding affinities and calculated interface energies in Rosetta and uses a Pearson correlation to determine success. The ranking power test refers to a score functions' ability to correctly rank compounds against a single target and uses a Spearman correlation to determine success.

These metrics were chosen simply by using the same analysis scheme as CASF making these results directly comparable to protocols/score functions used in other softwares.

Values for correlation can range between -1 and 1 where 1 is a perfect correlation.

We ran this test-set twice to obtain an average Spearman correlation for each target. In each test, we now compare the Spearman correlations of each target to the corresponding value. We are looking for major changes in correlation between runs, for better or worse. Success or failure is based on how many target cases are we deviating more than 0.2 from the standard for each respective target.

Overall success is defined as 75% of targets, we maintain within 0.2 of original value. Change these values as necessary.

Cutoffs were defined in the first test run. I should probably do more thorough analysis on previous runs, but looking at the most recent two, correlations between most targets has a standard deviation <0.2.

KEY RESULTS

In previous runs, ~75% of targets had Spearman correlations greater than 0.25 - this is not defined as a pass/fail but simply stated.

Previous Rosetta protocols.

DEFINITIONS AND COMMENTS

Analysis scripts were distributed by CASF, which made it easy to implement here (seriously a lot of copy/paste) meaning the analyses used here can be directly compared to others running the CASF benchmark.

The given XML uses old ligand score functions (which do work better than the newer ones across all tests), but to assess score functions that are currently being developed, I will likely move this to the new ones in the near future.

LIMITATIONS

The scoring test is rather vague as this takes the scores of all 285 complexes and determines how linearly correlated these scores are to experimental values. In such a diverse dataset, it is very difficult to capture information about specific interactions that might be outliers.

In the ranking test, there are clusters where ligands are in different binding pockets. Typically in a drug discovery campaign, groups acquire compound series that are directed towards the same pocket and there are slight chemical changes to tease out SAR information. In some of the ranking tests, those with lower correlations can be attributed to this. It would be nice to have a ranking test where we simply look at similar compound series that greater mimic hit-to-lead optimization efforts.

The dataset relies strictly on cocrystal structures, which can present issues when working with ligands due to strange geometries, non-ideal bonds or connectivities, etc.

More specific analysis, but probably not here. AKA Which cases do we see consistent good or poor results? Types of interactions that are characterized poorly? Ligand side: functional groups, descriptor classification? Protein side: particular problem residues, protein families, dynamics? It will be good to have this running on a consistent basis to see if these results are consistent. Currently, we only output 1 minimized structure (hopefully at the local minimum). In what I have seen before, results do not change much when we output more decoys, but this is something to keep in mind when looking at the results over time.

A technical thing: to make sure our analyses are consistent with CASF-published results, I used their scripts with no modification aside from making this compatible with the benchmark server setup. This means that I needed to include several python packages (pandas, sklearn and scipy), which I hope does not cause problems on the server.

This is a fast and easy benchmark that can yield information about specific protein-ligand interactions that the score function mischaracterizes. I hope that by running these tests consistently, we can better tease more specific information about where our score functions need improvement.

Again, I really want to emphasize that provided here are the datasets and analysis scripts that were used in the latest CASF assessment where we can compare these results to >30 other software suites that are popular in computational drug discovery.

REVISION

revision:61592

test_id: 687748

status: passed

Scientific test: loop_modeling_ccd_12res

FAILURES

None

RESULTS

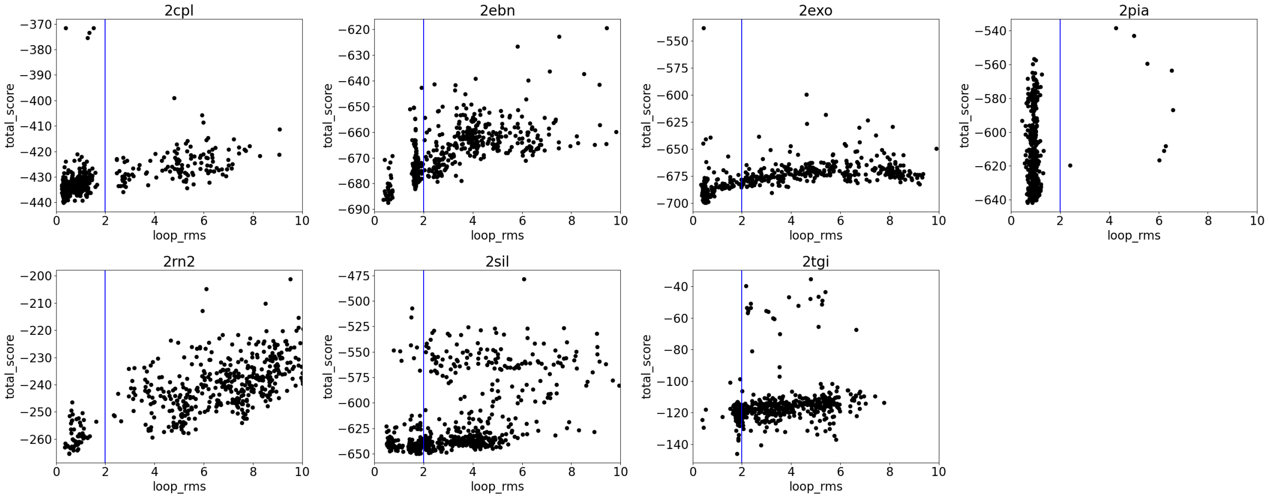

AUTHOR AND DATE

Phuong T. Nguyen, tranphuonguns@gmail.com, PI: Vladimir Yarov-Yarovoy, Jun 2019

PURPOSE OF THE TEST

This scientific benchmark tests loop modeling protocol using different algorithms:

- classical methods: cyclic coordinate descent (CCD) and kinematic loop closure (KIC)

- more recent kinematic-based methods: Next-generation kic (NGK) and kic with fragments (KIC-FRAG)

BENCHMARK DATASET

A standard 12-residue mini benchmark set taken from the Kortemme's lab full benchmark set: https://guybrush.ucsf.edu/benchmarks/benchmarks/loop_modeling

This set of proteins was manually curated to select 7 proteins with different sizes ranging from 100-400 amino acids.

Input proteins were preminimized with Rosetta, native coordinates of loop regions were removed along with sidechains of nearby residues within 10A.

Non-native conformations of these loops were then added as a starting conformation. Reference native structures are also provided for analysis.

PROTOCOL

The NGK and KIC-FRAG protocols run LoopModelerMover through RosettaScripts interface. This Mover currently only supports kinematic-based sampling. This was used in the Kortemme lab's benchmark sets, but with much older Rosetta version and scoring functions (score12, talaris2013, talaris2014). Here ref2015 scoring function is used. Note that, despite simulation configuration is called as config="kic", this mover appears to invoke ramping on repulsive and rama by default, which is NGK method.

RMSDMetric was used to calculate the difference in generated loop conformations compared to native.

No superposition was performed as the protocol does not move other parts of input proteins.

The classic protocols CCD and KIC run through loopmodel application. Rmsd calculation is integrated in the application.

The CCD and KIC-FRAG used fragments insertion while classical KIC and NGK do not.

Runtime (for nstruct = 500):

Total: 2456 CPU hours

NGK: 571 CPU hours | KIC-FRAG:766 CPU hours | KIC: 621 CPU hours | CCD: 498 CPU hours

PERFORMANCE METRICS

The total_score and rmsd (loop_rms) to the native were used as metrics. These values are plotted with expectation of forming a funnel shape. However, in actual modeling process, we don't know the native conformation, which makes the score vs rmsd plot not very useful. We rely mostly on the far-perfect Rosetta scoring function to make a decision. Here we chose 10% cutoff for score vs rmsd to describe ultimate pass/fail. The test will pass if we can find any structures in the top 10% scoring models that has rmsd to the native less than a cutoff value. We use a cutoff of 2.0A for all loop modeling protocols and all targets.

KEY RESULTS

Using nstruct = 500, we were able to observe somewhat funnel shapes in the total_score vs loop_rms plots across different targets with all four algorithms.

We also observed sub-angstrom accuracy for several targets.

The 2TGI structure appeared to be a difficult case and required a higher cutoff value to pass.

DEFINITIONS AND COMMENTS

All methods need both ends of loop regions to perform kinematic sampling, thus cannot model loop regions at termini. Cyclic coordinate descent (CCD) is the only method can do the job, with the condition not using kinematic sampling in the refinement stage. To do that, users need to specify "refine_ccd", instead of "refine_kic" in the running flags.

Phuong T. Nguyen (tigerous) is not an expert in Rosetta loop modeling.

LIMITATIONS

The dataset is not big and diverse enough to draw a full conclusion, but appear to be decent enough to describe a working algorithm.

REVISION

revision:61592

test_id: 687735

status: passed

Scientific test: loop_modeling_kic_12res

FAILURES

None

RESULTS

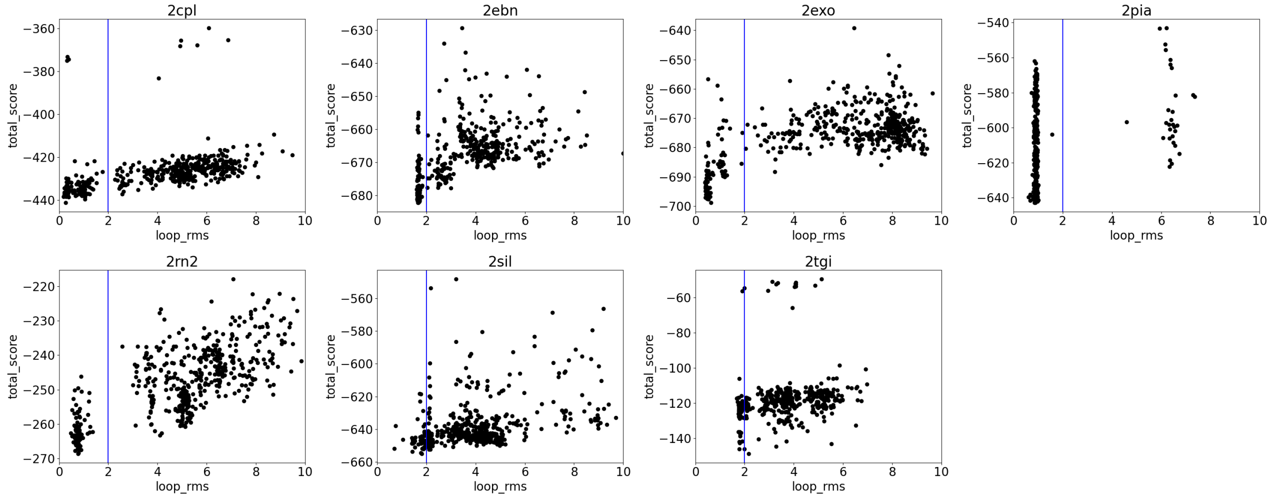

AUTHOR AND DATE

Phuong T. Nguyen, tranphuonguns@gmail.com, PI: Vladimir Yarov-Yarovoy, Jun 2019

PURPOSE OF THE TEST

This scientific benchmark tests loop modeling protocol using different algorithms:

- classical methods: cyclic coordinate descent (CCD) and kinematic loop closure (KIC)

- more recent kinematic-based methods: Next-generation kic (NGK) and kic with fragments (KIC-FRAG)

BENCHMARK DATASET

A standard 12-residue mini benchmark set taken from the Kortemme's lab full benchmark set: https://guybrush.ucsf.edu/benchmarks/benchmarks/loop_modeling

This set of proteins was manually curated to select 7 proteins with different sizes ranging from 100-400 amino acids.

Input proteins were preminimized with Rosetta, native coordinates of loop regions were removed along with sidechains of nearby residues within 10A.

Non-native conformations of these loops were then added as a starting conformation. Reference native structures are also provided for analysis.

PROTOCOL

The NGK and KIC-FRAG protocols run LoopModelerMover through RosettaScripts interface. This Mover currently only supports kinematic-based sampling. This was used in the Kortemme lab's benchmark sets, but with much older Rosetta version and scoring functions (score12, talaris2013, talaris2014). Here ref2015 scoring function is used. Note that, despite simulation configuration is called as config="kic", this mover appears to invoke ramping on repulsive and rama by default, which is NGK method.

RMSDMetric was used to calculate the difference in generated loop conformations compared to native.

No superposition was performed as the protocol does not move other parts of input proteins.

The classic protocols CCD and KIC run through loopmodel application. Rmsd calculation is integrated in the application.

The CCD and KIC-FRAG used fragments insertion while classical KIC and NGK do not.

Runtime (for nstruct = 500):

Total: 2456 CPU hours

NGK: 571 CPU hours | KIC-FRAG:766 CPU hours | KIC: 621 CPU hours | CCD: 498 CPU hours

PERFORMANCE METRICS

The total_score and rmsd (loop_rms) to the native were used as metrics. These values are plotted with expectation of forming a funnel shape. However, in actual modeling process, we don't know the native conformation, which makes the score vs rmsd plot not very useful. We rely mostly on the far-perfect Rosetta scoring function to make a decision. Here we chose 10% cutoff for score vs rmsd to describe ultimate pass/fail. The test will pass if we can find any structures in the top 10% scoring models that has rmsd to the native less than a cutoff value. We use a cutoff of 2.0A for all loop modeling protocols and all targets.

KEY RESULTS

Using nstruct = 500, we were able to observe somewhat funnel shapes in the total_score vs loop_rms plots across different targets with all four algorithms.

We also observed sub-angstrom accuracy for several targets.

The 2TGI structure appeared to be a difficult case and required a higher cutoff value to pass.

DEFINITIONS AND COMMENTS

All methods need both ends of loop regions to perform kinematic sampling, thus cannot model loop regions at termini. Cyclic coordinate descent (CCD) is the only method can do the job, with the condition not using kinematic sampling in the refinement stage. To do that, users need to specify "refine_ccd", instead of "refine_kic" in the running flags.

Phuong T. Nguyen (tigerous) is not an expert in Rosetta loop modeling.

LIMITATIONS

The dataset is not big and diverse enough to draw a full conclusion, but appear to be decent enough to describe a working algorithm.

REVISION

revision:61600

test_id: 689964

status: passed

Scientific test: loop_modeling_kic_fragments_12res

FAILURES

None

RESULTS

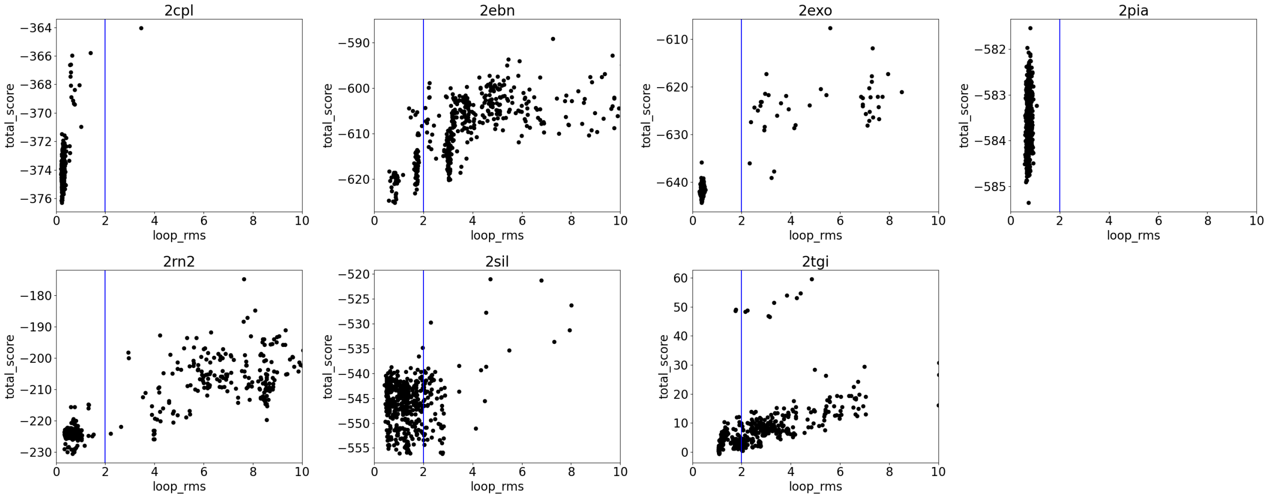

AUTHOR AND DATE

Phuong T. Nguyen, tranphuonguns@gmail.com, PI: Vladimir Yarov-Yarovoy, Jun 2019

PURPOSE OF THE TEST

This scientific benchmark tests loop modeling protocol using different algorithms:

- classical methods: cyclic coordinate descent (CCD) and kinematic loop closure (KIC)

- more recent kinematic-based methods: Next-generation kic (NGK) and kic with fragments (KIC-FRAG)

BENCHMARK DATASET

A standard 12-residue mini benchmark set taken from the Kortemme's lab full benchmark set: https://guybrush.ucsf.edu/benchmarks/benchmarks/loop_modeling

This set of proteins was manually curated to select 7 proteins with different sizes ranging from 100-400 amino acids.

Input proteins were preminimized with Rosetta, native coordinates of loop regions were removed along with sidechains of nearby residues within 10A.

Non-native conformations of these loops were then added as a starting conformation. Reference native structures are also provided for analysis.

PROTOCOL

The NGK and KIC-FRAG protocols run LoopModelerMover through RosettaScripts interface. This Mover currently only supports kinematic-based sampling. This was used in the Kortemme lab's benchmark sets, but with much older Rosetta version and scoring functions (score12, talaris2013, talaris2014). Here ref2015 scoring function is used. Note that, despite simulation configuration is called as config="kic", this mover appears to invoke ramping on repulsive and rama by default, which is NGK method.

RMSDMetric was used to calculate the difference in generated loop conformations compared to native.

No superposition was performed as the protocol does not move other parts of input proteins.

The classic protocols CCD and KIC run through loopmodel application. Rmsd calculation is integrated in the application.

The CCD and KIC-FRAG used fragments insertion while classical KIC and NGK do not.

Runtime (for nstruct = 500):

Total: 2456 CPU hours

NGK: 571 CPU hours | KIC-FRAG:766 CPU hours | KIC: 621 CPU hours | CCD: 498 CPU hours

PERFORMANCE METRICS

The total_score and rmsd (loop_rms) to the native were used as metrics. These values are plotted with expectation of forming a funnel shape. However, in actual modeling process, we don't know the native conformation, which makes the score vs rmsd plot not very useful. We rely mostly on the far-perfect Rosetta scoring function to make a decision. Here we chose 10% cutoff for score vs rmsd to describe ultimate pass/fail. The test will pass if we can find any structures in the top 10% scoring models that has rmsd to the native less than a cutoff value. We use a cutoff of 2.0A for all loop modeling protocols and all targets.

KEY RESULTS

Using nstruct = 500, we were able to observe somewhat funnel shapes in the total_score vs loop_rms plots across different targets with all four algorithms.

We also observed sub-angstrom accuracy for several targets.

The 2TGI structure appeared to be a difficult case and required a higher cutoff value to pass.

DEFINITIONS AND COMMENTS

All methods need both ends of loop regions to perform kinematic sampling, thus cannot model loop regions at termini. Cyclic coordinate descent (CCD) is the only method can do the job, with the condition not using kinematic sampling in the refinement stage. To do that, users need to specify "refine_ccd", instead of "refine_kic" in the running flags.

Phuong T. Nguyen (tigerous) is not an expert in Rosetta loop modeling.

LIMITATIONS

The dataset is not big and diverse enough to draw a full conclusion, but appear to be decent enough to describe a working algorithm.

REVISION

revision:61603

test_id: 690517

status: passed

Scientific test: loop_modeling_ngk_12res

FAILURES

None

RESULTS

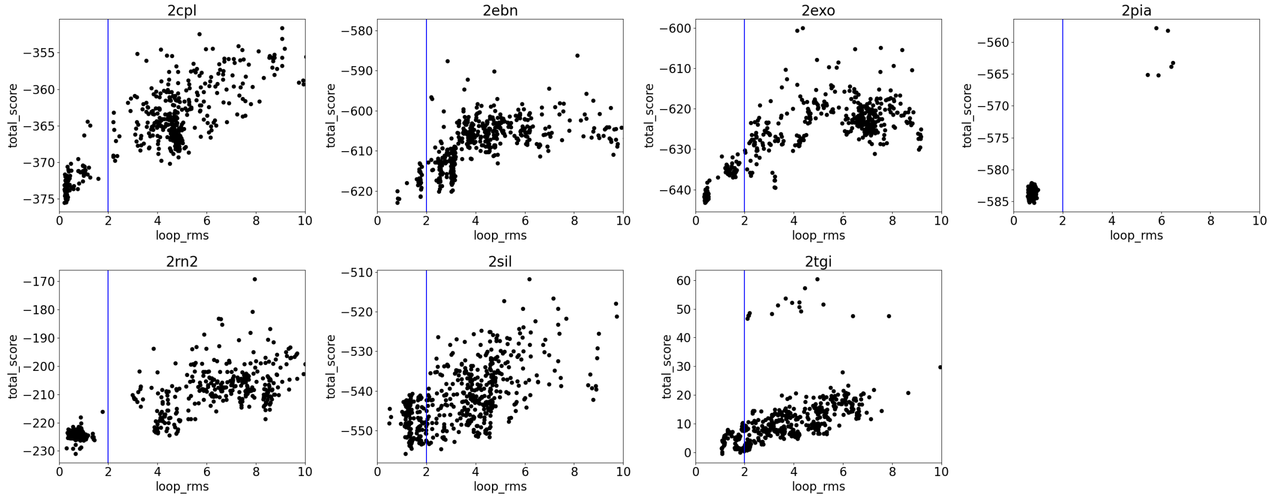

AUTHOR AND DATE

Phuong T. Nguyen, tranphuonguns@gmail.com, PI: Vladimir Yarov-Yarovoy, Jun 2019

PURPOSE OF THE TEST

This scientific benchmark tests loop modeling protocol using different algorithms:

- classical methods: cyclic coordinate descent (CCD) and kinematic loop closure (KIC)

- more recent kinematic-based methods: Next-generation kic (NGK) and kic with fragments (KIC-FRAG)

BENCHMARK DATASET

A standard 12-residue mini benchmark set taken from the Kortemme's lab full benchmark set: https://guybrush.ucsf.edu/benchmarks/benchmarks/loop_modeling

This set of proteins was manually curated to select 7 proteins with different sizes ranging from 100-400 amino acids.

Input proteins were preminimized with Rosetta, native coordinates of loop regions were removed along with sidechains of nearby residues within 10A.

Non-native conformations of these loops were then added as a starting conformation. Reference native structures are also provided for analysis.

PROTOCOL

The NGK and KIC-FRAG protocols run LoopModelerMover through RosettaScripts interface. This Mover currently only supports kinematic-based sampling. This was used in the Kortemme lab's benchmark sets, but with much older Rosetta version and scoring functions (score12, talaris2013, talaris2014). Here ref2015 scoring function is used. Note that, despite simulation configuration is called as config="kic", this mover appears to invoke ramping on repulsive and rama by default, which is NGK method.

RMSDMetric was used to calculate the difference in generated loop conformations compared to native.

No superposition was performed as the protocol does not move other parts of input proteins.

The classic protocols CCD and KIC run through loopmodel application. Rmsd calculation is integrated in the application.

The CCD and KIC-FRAG used fragments insertion while classical KIC and NGK do not.

Runtime (for nstruct = 500):

Total: 2456 CPU hours

NGK: 571 CPU hours | KIC-FRAG:766 CPU hours | KIC: 621 CPU hours | CCD: 498 CPU hours

PERFORMANCE METRICS

The total_score and rmsd (loop_rms) to the native were used as metrics. These values are plotted with expectation of forming a funnel shape. However, in actual modeling process, we don't know the native conformation, which makes the score vs rmsd plot not very useful. We rely mostly on the far-perfect Rosetta scoring function to make a decision. Here we chose 10% cutoff for score vs rmsd to describe ultimate pass/fail. The test will pass if we can find any structures in the top 10% scoring models that has rmsd to the native less than a cutoff value. We use a cutoff of 2.0A for all loop modeling protocols and all targets.

KEY RESULTS

Using nstruct = 500, we were able to observe somewhat funnel shapes in the total_score vs loop_rms plots across different targets with all four algorithms.

We also observed sub-angstrom accuracy for several targets.

The 2TGI structure appeared to be a difficult case and required a higher cutoff value to pass.

DEFINITIONS AND COMMENTS

All methods need both ends of loop regions to perform kinematic sampling, thus cannot model loop regions at termini. Cyclic coordinate descent (CCD) is the only method can do the job, with the condition not using kinematic sampling in the refinement stage. To do that, users need to specify "refine_ccd", instead of "refine_kic" in the running flags.

Phuong T. Nguyen (tigerous) is not an expert in Rosetta loop modeling.

LIMITATIONS

The dataset is not big and diverse enough to draw a full conclusion, but appear to be decent enough to describe a working algorithm.

REVISION

revision:61603

test_id: 690515

status: passed

Scientific test: make_fragments

FAILURES

None

RESULTS

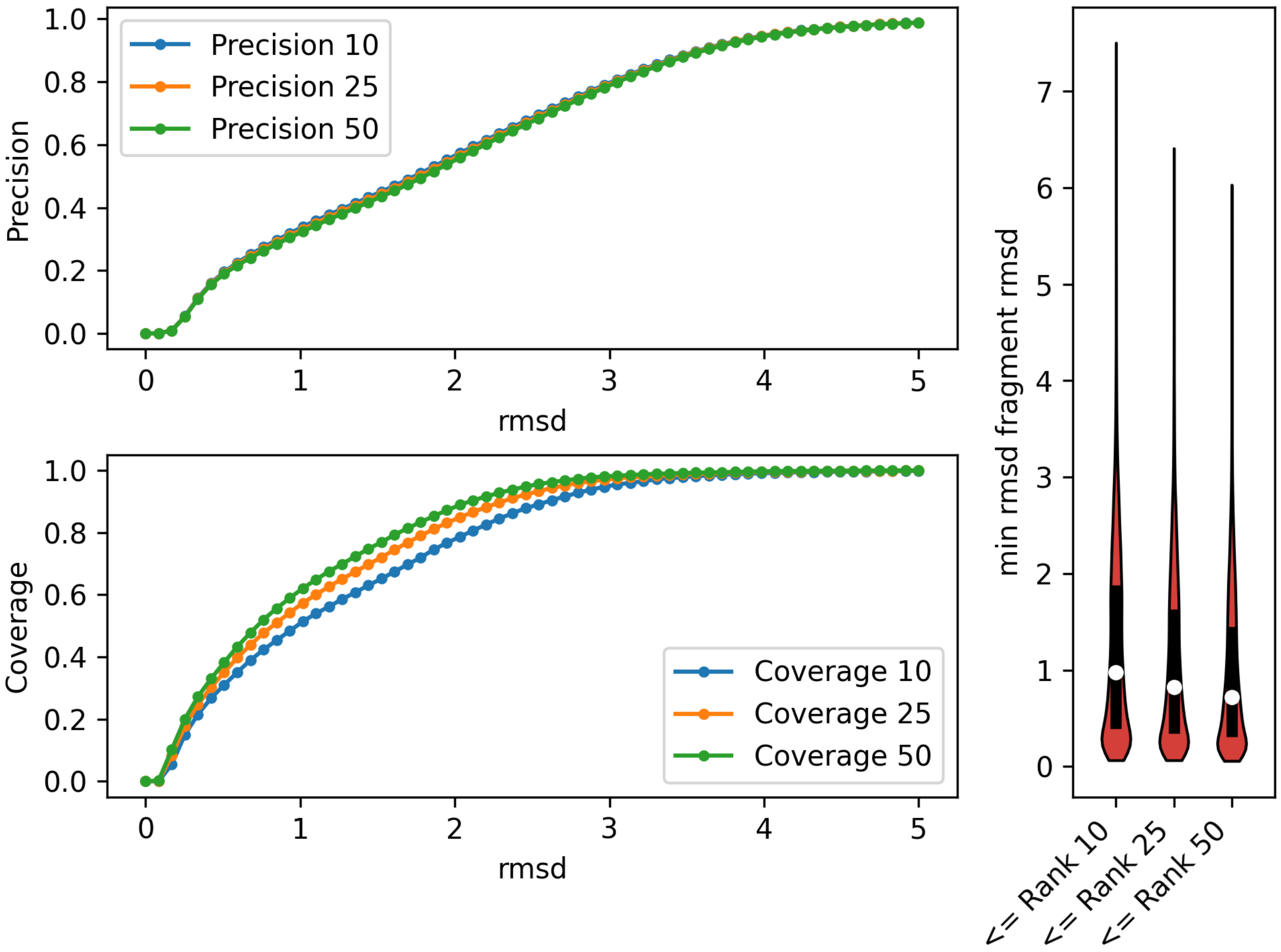

AUTHOR AND DATE

Author: Daniel Farrell

Email: danpf@uw.edu

PI: Frank DiMaio

@DD-MM-YYYY 30-06-2019

PURPOSE OF THE TEST

This tests how fragments are made and the results of fragment picking

What does the benchmark test and why?

This test benchmarks the whole 'make fragments' pipeline which includes:

```
- Running blast
- Running psipred
- Building the nr database
- running rosetta fragment picker
```

Why test the whole pipeline instead of just fragment_picker?

```
- Most improvements to the pipeline (unpublished so far) have mainly been thanks to:
    - Improved databases
    - Improved SS prediction
    - Alternative methods of PSSM generation
    - Addition of additional outside information
- Database related questions often come up, this will give us a baseline to compare against
    - The NR database is unversioned -- This gives us, and the community a version to benchmark with
- The make-fragments pipeline relys on a lot of non-rosetta downloads, and therefore has a tendency to break
```

therefore we can come up a sort of pros and cons list for whole pipeline vs rosetta part only:

```
- Pros for whole pipeline:
    - Scientifically -- fragment picking is the sum of the whole, and testing only the rosetta part with fixed inputs is quite a narrow part of the whole.
    - Most community documentation is based on 'make_fragments.pl' and it has a tendency to break, this gives us a chance to catch bugs sooner
    - Because most improvements have been database/outside input improvements, a full pipeline benchmark will help developers have something concrete to compare improvements to.
    - When someone inevitably updates the pipeline, they will be able to easily compare results using a full pipeline test like this.
- Cons against the whole pipeline:
    - It takes a very long time to run
    - It is multithreaded (which is incompatible with CONDOR)
    - Because outside programs are run -- we are scientifically checking the sum of the whole, rather than just changes to rosetta.
```

In the end, there is no right or wrong answer, however, due to a signifigant number of forum, and email questions relating to the install

of fragment picking to local machines + the hopes that someone will be able to easily update the 2 perl scripts to something more manageable

(like python) I have chosen to benchmark the whole pipeline.

BENCHMARK DATASET

How many proteins are in the set?

From casp12: 51 proteins

From casp13: 14 proteins

Total: 65 proteins

What dataset are you using? Is it published? If yes, please add a citation.

casp12 and casp13

What are the input files? How were the they created?

They are pulled pdbs from the casp website and manually renamed by me (Daniel Farrell). The set has the format:

```
{
 "casp_12": {
  "target_id": {
   "pdb_text": "pdb_text...",
   "target_sequence": "MYSEQVENCE..."
  }
 }
 ...
```

PROTOCOL

State and briefly describe the protocol.

The input file is a sequence, we take the sequence and run 'make_fragments.pl' on it. This tests how the most general/public facing fragment picking protocol works. The perl script runs and we get 3mers and 9mers from it. The 9mers are used for testing purposes and are compared against the native pdb if a full 9 residue match exists. those results are curated and plotted.

make_fragments.pl works briefly by:

```
- run `psiblast` for pssm generation
- run `psipred` for ss-prediction
- run `psiblast` for homolog detection
- run sparksX for phi psi and solvent accessibility predictions
- run `fragment_picker` to finally pick fragments
```

The protocol isn't ideal (multiple psiblast runs), however this is what most people use to pick fragments (if not robetta). The main differences between this and robetta are alternative methods of ss-prediction, and robetta has integrated the hhsuite into their fragment picking methods.

I unfortunately am unaware of the accuracy of the picker without sparksX or psiblast etc. This will simply be a baseline due to the fact that this is essentially a snapshot of the protocol from 5-7 years ago, and most of the people that built it have moved on.

Is there a publication that describes the protocol?

Gront, D., Kulp, D. W., Vernon, R. M., Strauss, C. E. M. & Baker, D. Generalized Fragment Picking in Rosetta : Design , Protocols and Applications. 6, (2011).

How many CPU hours does this benchmark take approximately?

First the nr database has to be built (~20 cores x 1-2 days). Then each total amount of running `make\_fragments.pl` takes about 1 (or 2-3) cores 3-10 hrs. For a total of ~2000 hrs. Different

`psiblast` calls can take up to 3 cores (for unknown reasons) but this should work.

PERFORMANCE METRICS

What are the performance metrics used and why were they chosen?

I plotted `coverage` and `precision` based on many fragment picking papers such as:

```
de Oliveira SH, Shi J, Deane CM. Building a better fragment library for de novo protein structure prediction. PLoS One. 2015;10(4):e0123998. Published 2015 Apr 22. doi:10.1371/journal
```

`precision` is defined as proportion of good fragments in the library (at various cutoff to define 'good')

`coverage` is defined as the percentage of target residues represented by at least one good fragment in the library

```
coverages = []
for every rmsd_cutoff in 0->5 (60 bins):
 total = 0
 cov_count = 0
 For every residue ( aka fragment set ):
  for every fragment_cutoff in [10, 25, 50]:
   total += 1
   if the minimum rmsd in the fragments from 0->cutoff is less than the rmsd_cutoff:
    cov_count += 1
 coveraged.append(cov_count/total)
```

I also plotted violin plots to show overall distributions of all plotted fragments at different cutoffs (most rosetta protocols use the top 25 fragments, but we pick 200).

How do you define a pass/fail for this test?

We can set a pass/fail based on the median and quartile ranges of the top 25 fragments + looking at the deviations from run to run. With an identical database the files that we make are deterministic so we can use this test to determine the changes in all aspects of fragment-picking.

How were any cutoffs defined?

By the current results.

KEY RESULTS

What is the baseline to compare things to - experimental data or a previous Rosetta protocol?

The baseline is the solved crystal/nmr structures from casp

Describe outliers in the dataset.

There are no outliers.

DEFINITIONS AND COMMENTS

State anything you think is important for someone else to replicate your results.

```
- Ask Sergey Lyskov to the stored `nr` database.  We pegged it to `May 25 2019`.  the ncbi   does not store these databases.  (@sergey maybe we should provide a public link here)
- There are some executables (sparksX, psiblast, psipred) that we should probably host ourselves or else they might dissapear someday.
- Most of the improvements in fragment picking over the last few years have been database related, improvements in ss prediction and addition of hhblits (not included in this protocol
```

LIMITATIONS

What are the limitations of the benchmark? Consider dataset, quality measures, protocol etc.

This benchmark is specifically crafted to test our capabilities at getting near-rmsd fragments. For some protocols having near-native fragments may not be enough (ie abinitio). Additionally we are limited to the older version of make fragments as the new one has been integrated into robetta and is not yet ready for public release.

How could the benchmark be improved?

```
- Newer fragment picker pipeline from robetta
- multithreaded fragment\_picker
- A non-casp dataset
- less blast/psiblast runs
```

What goals should be hit to make this a "good" benchmark?

REVISION

revision:61592

test_id: 687733

status: passed

Scientific test: mhc_epitope_energy

FAILURES

None

RESULTS

mhc_epitope

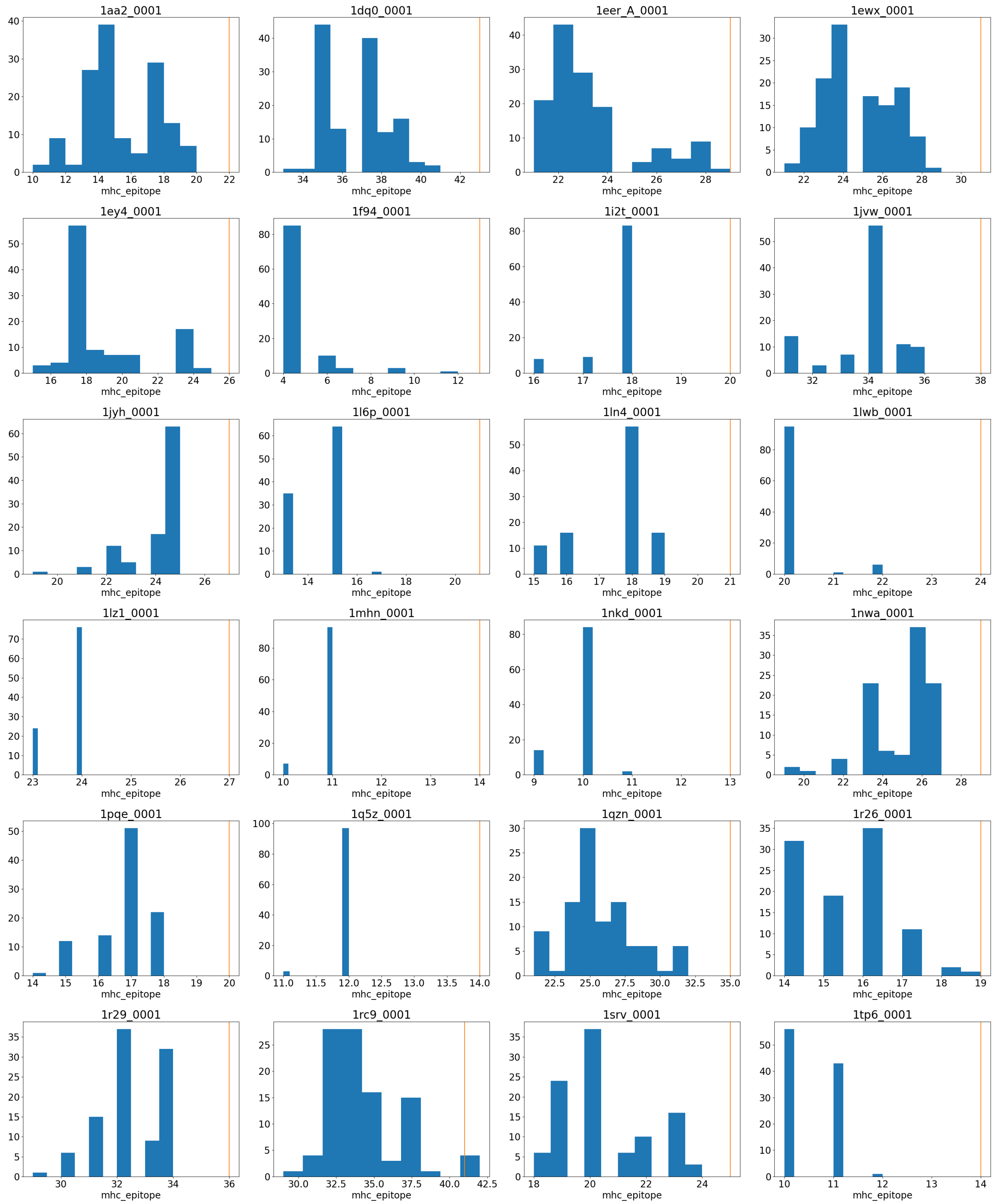

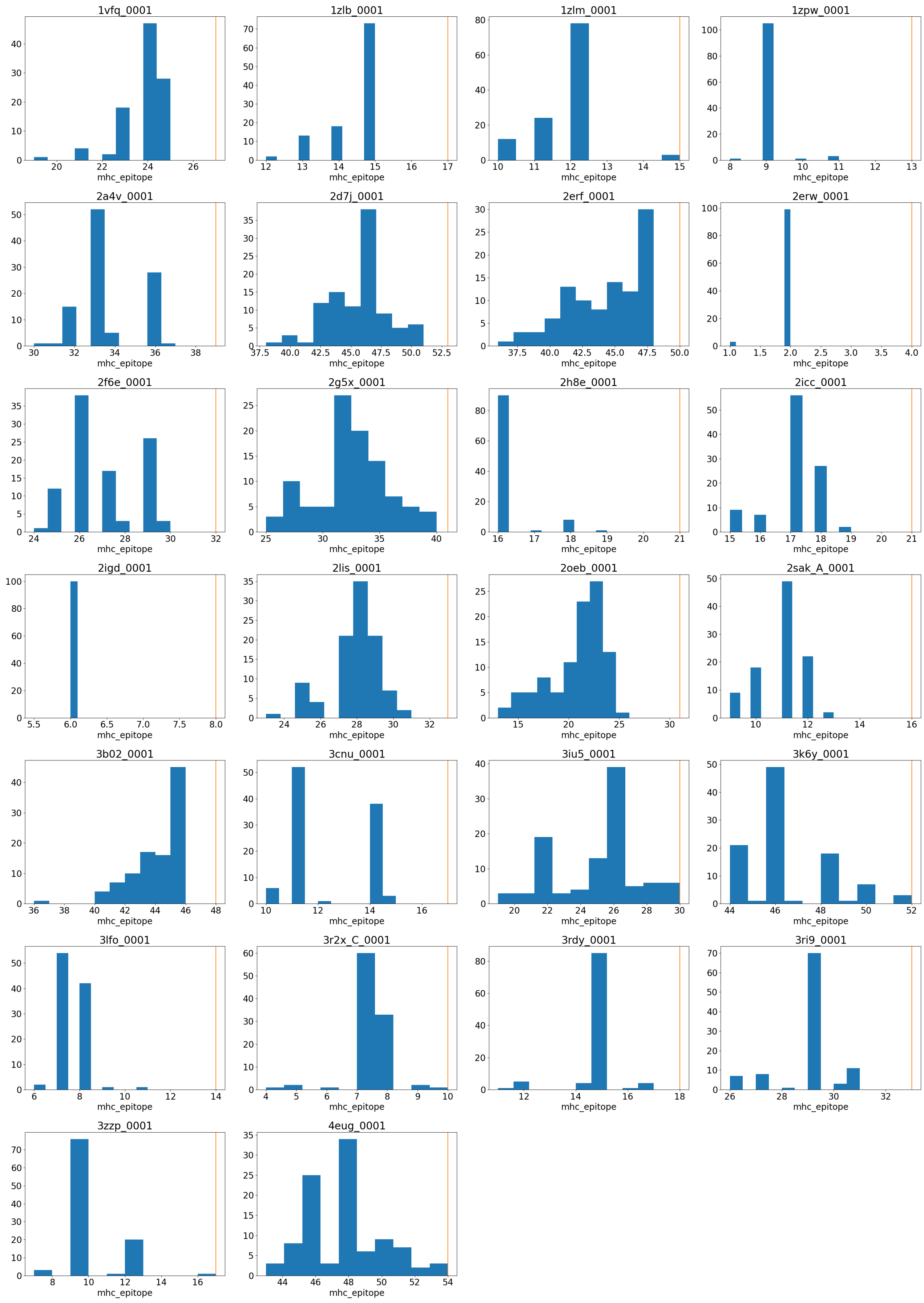

delta_mhc_epitope

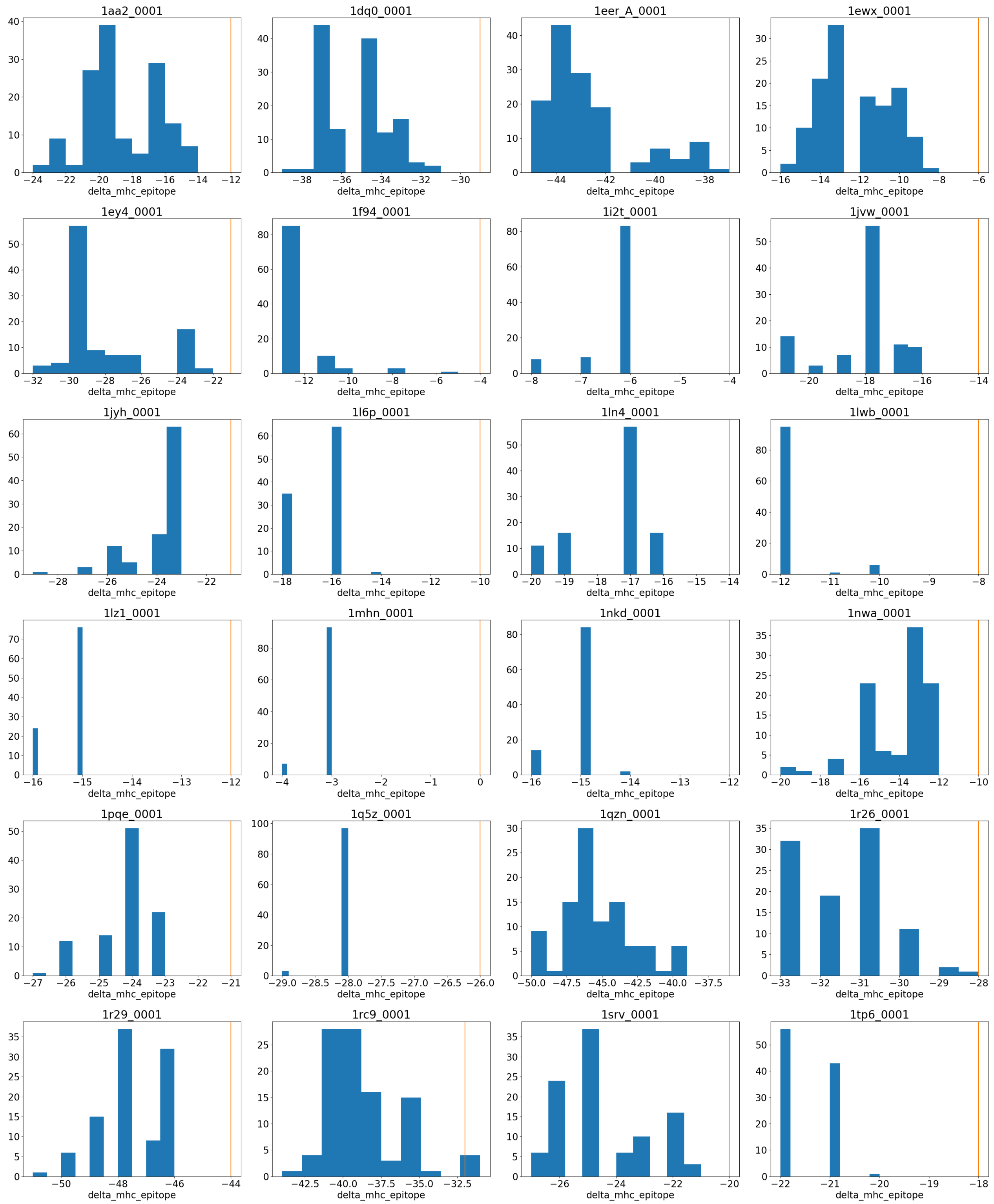
1aa2_0001
1dq0_0001
1eer_A_0001
1ewx_0001
1ey4_0001
1f94_0001
1i2t_0001
1jvw_0001
1jyh_0001
1l6p_0001
1ln4_0001
1lwb_0001
1lz1_0001
1mhn_0001
1nkd_0001
1nwa_0001
1pqe_0001
1q5z_0001
1qzn_0001
1r26_0001
1r29_0001
1rc9_0001
1srv_0001
1tp6_0001
delta_mhc_epitope

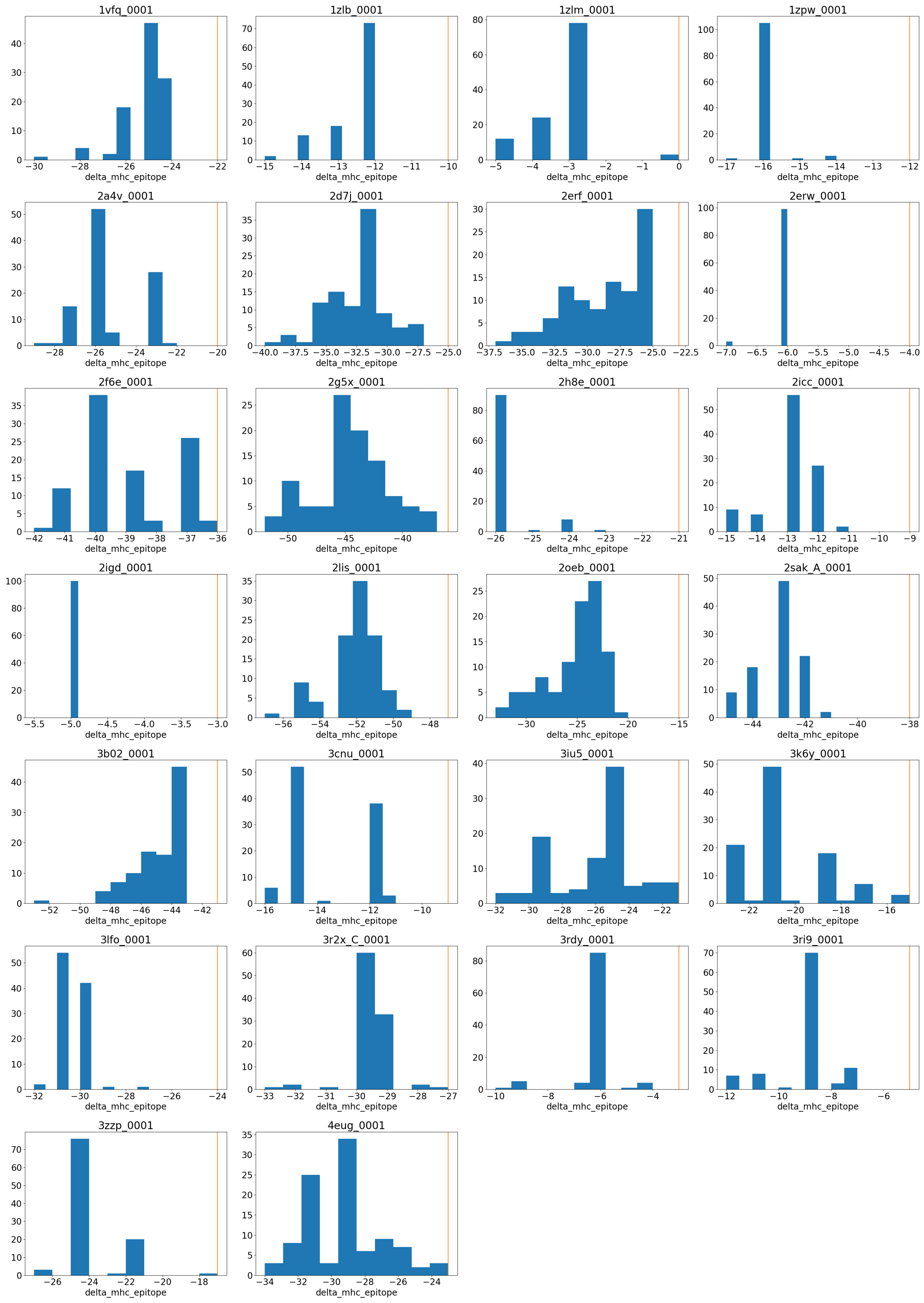

base_total_score_vs_mhc_epitope

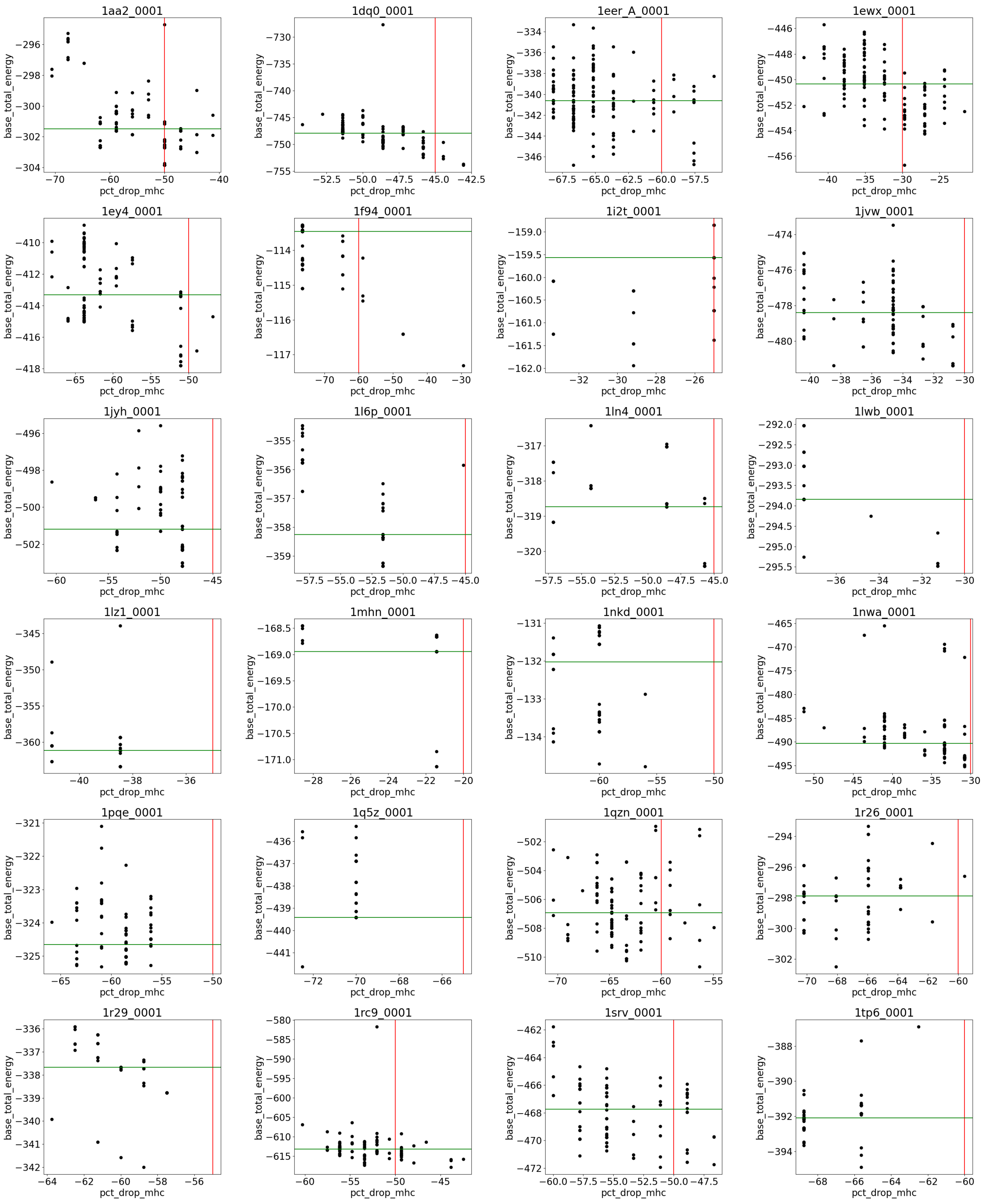
1aa2_0001
1dq0_0001
1eer_A_0001
1ewx_0001
1ey4_0001
1f94_0001
1i2t_0001
1jvw_0001
1jyh_0001
1l6p_0001
1ln4_0001
1lwb_0001
1lz1_0001
1mhn_0001
1nkd_0001
1nwa_0001
1pqe_0001
1q5z_0001
1qzn_0001
1r26_0001
1r29_0001
1rc9_0001
1srv_0001
1tp6_0001
base_total_energy
pct_drop_mhc

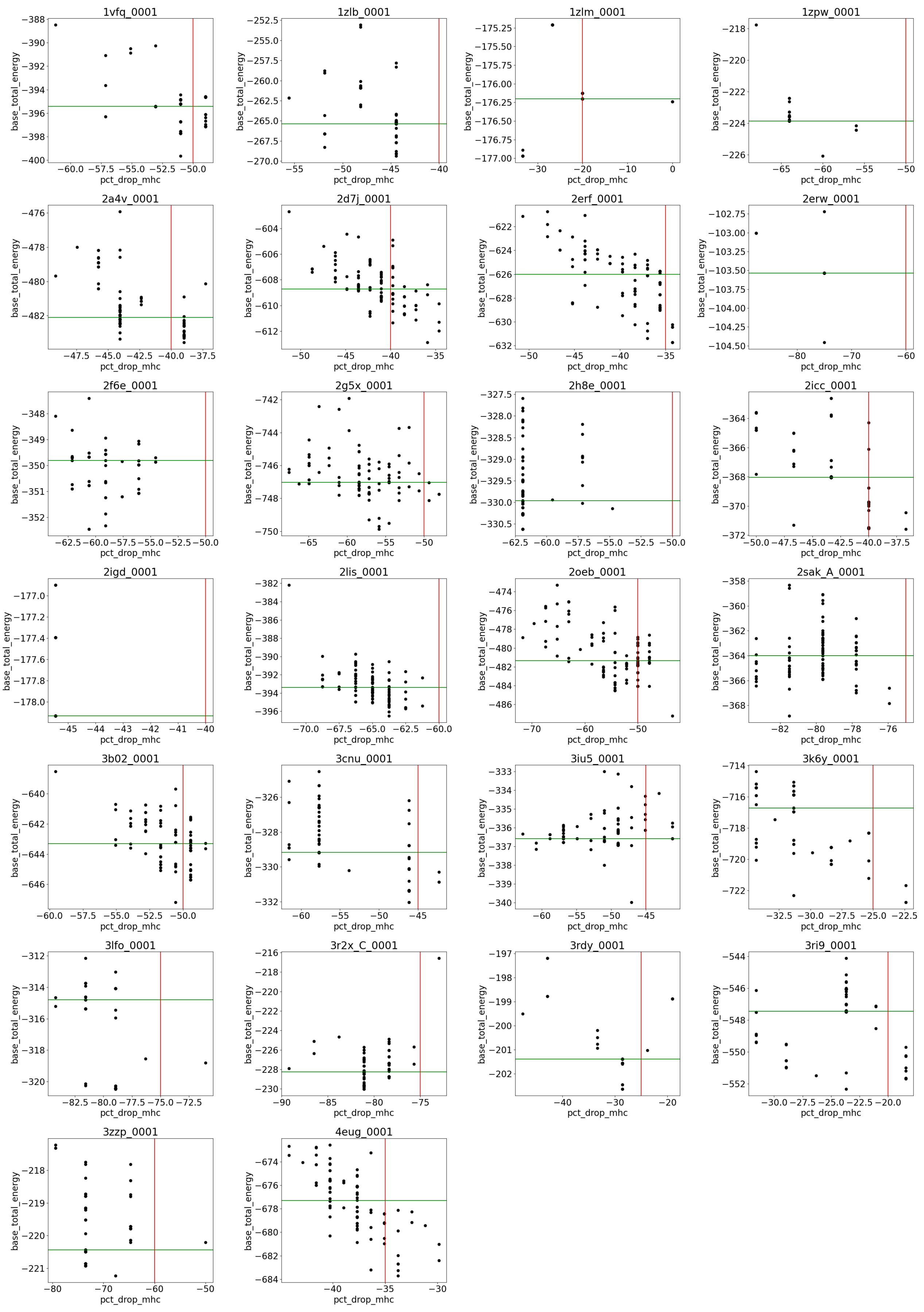

sequence_recovery_vs_mhc_epitope

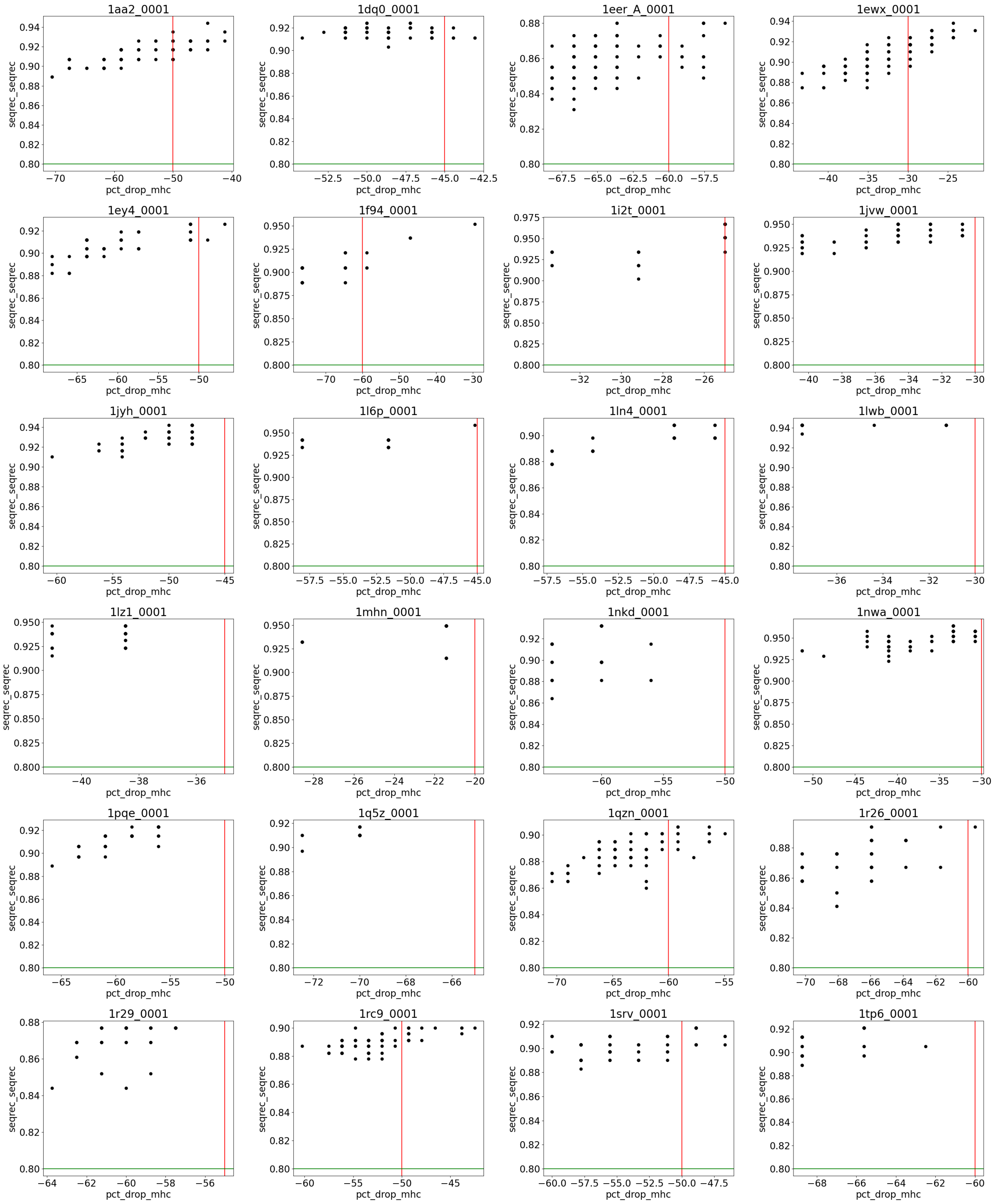
1aa2_0001
1dq0_0001
1eer_A_0001
1ewx_0001
1ey4_0001
1f94_0001
1i2t_0001
1jvw_0001
1jyh_0001
1l6p_0001
1ln4_0001
1lwb_0001
1lz1_0001
1mhn_0001
1nkd_0001
1nwa_0001
1pqe_0001
1q5z_0001
1qzn_0001
1r26_0001
1r29_0001
1rc9_0001
1srv_0001
1tp6_0001
seqrec_seqrec
pct_drop_mhc

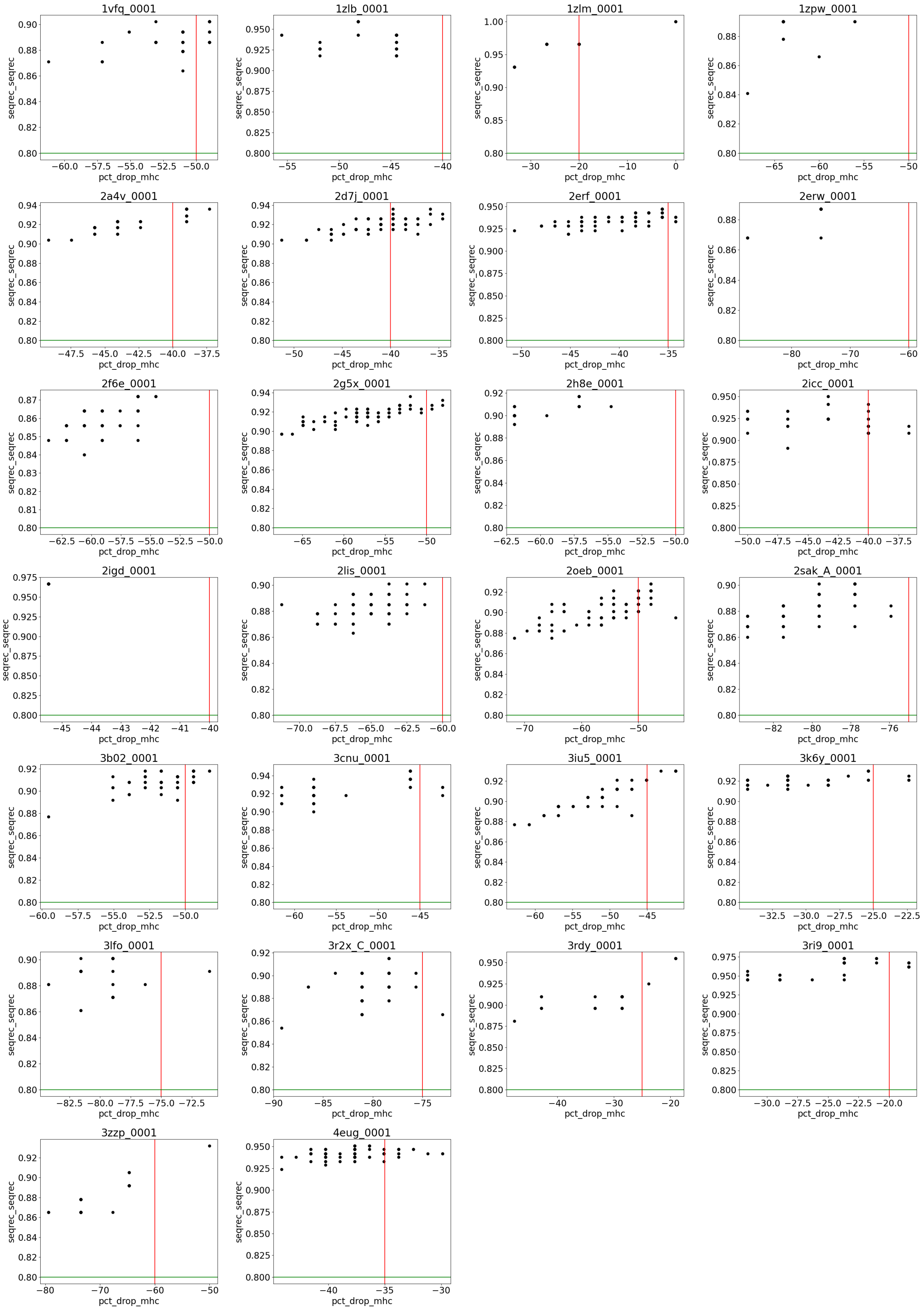

core_sequence_recovery_vs_mhc_epitope

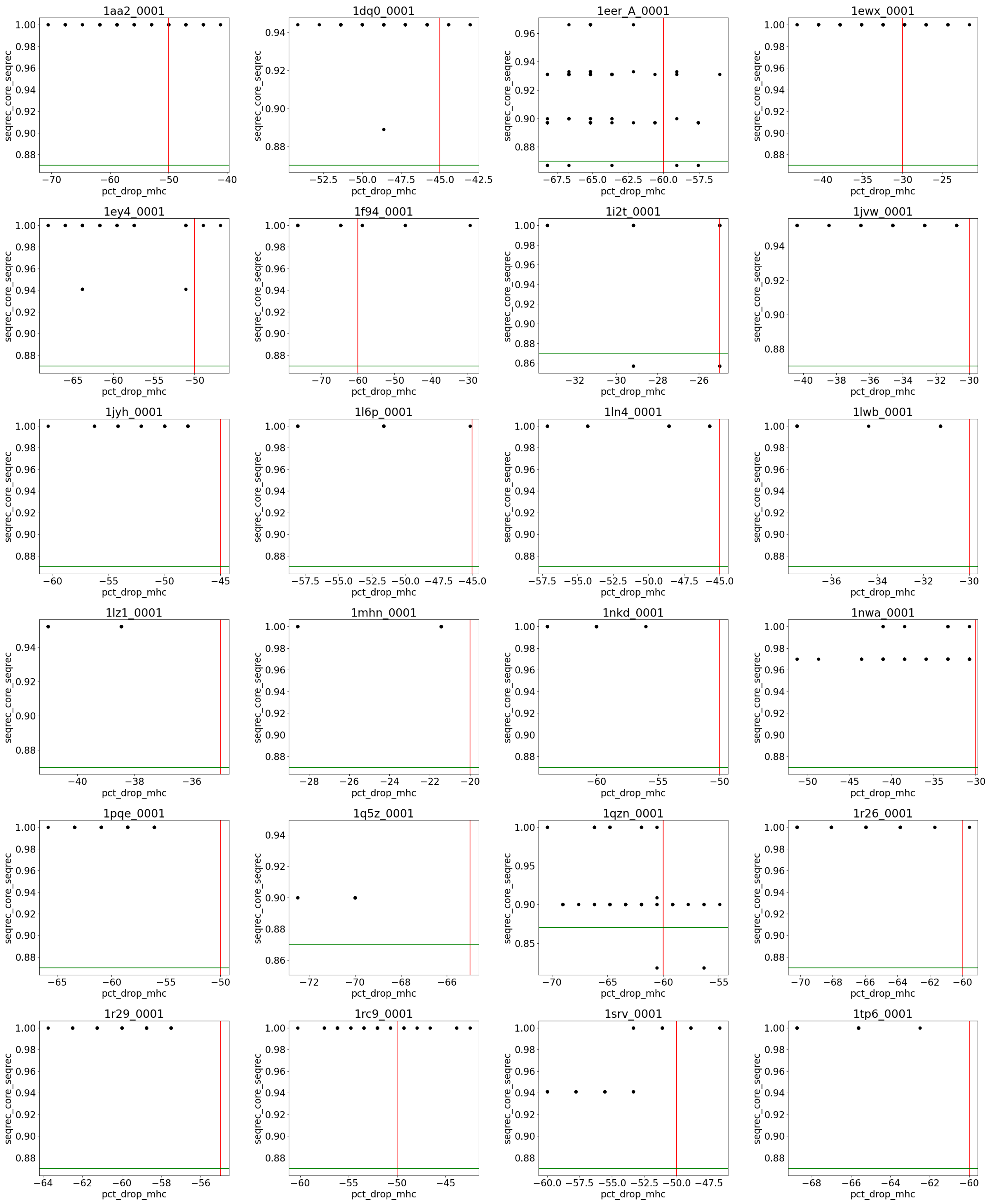

1aa2_0001
1dq0_0001
1eer_A_0001
1ewx_0001
1ey4_0001
1f94_0001
1i2t_0001
1jvw_0001
1jyh_0001
1l6p_0001
1ln4_0001
1lwb_0001
1lz1_0001
1mhn_0001
1nkd_0001
1nwa_0001
1pqe_0001
1q5z_0001
1qzn_0001
1r26_0001
1r29_0001
1rc9_0001
1srv_0001
1tp6_0001
seqrec_core_seqrec
pct_drop_mhc

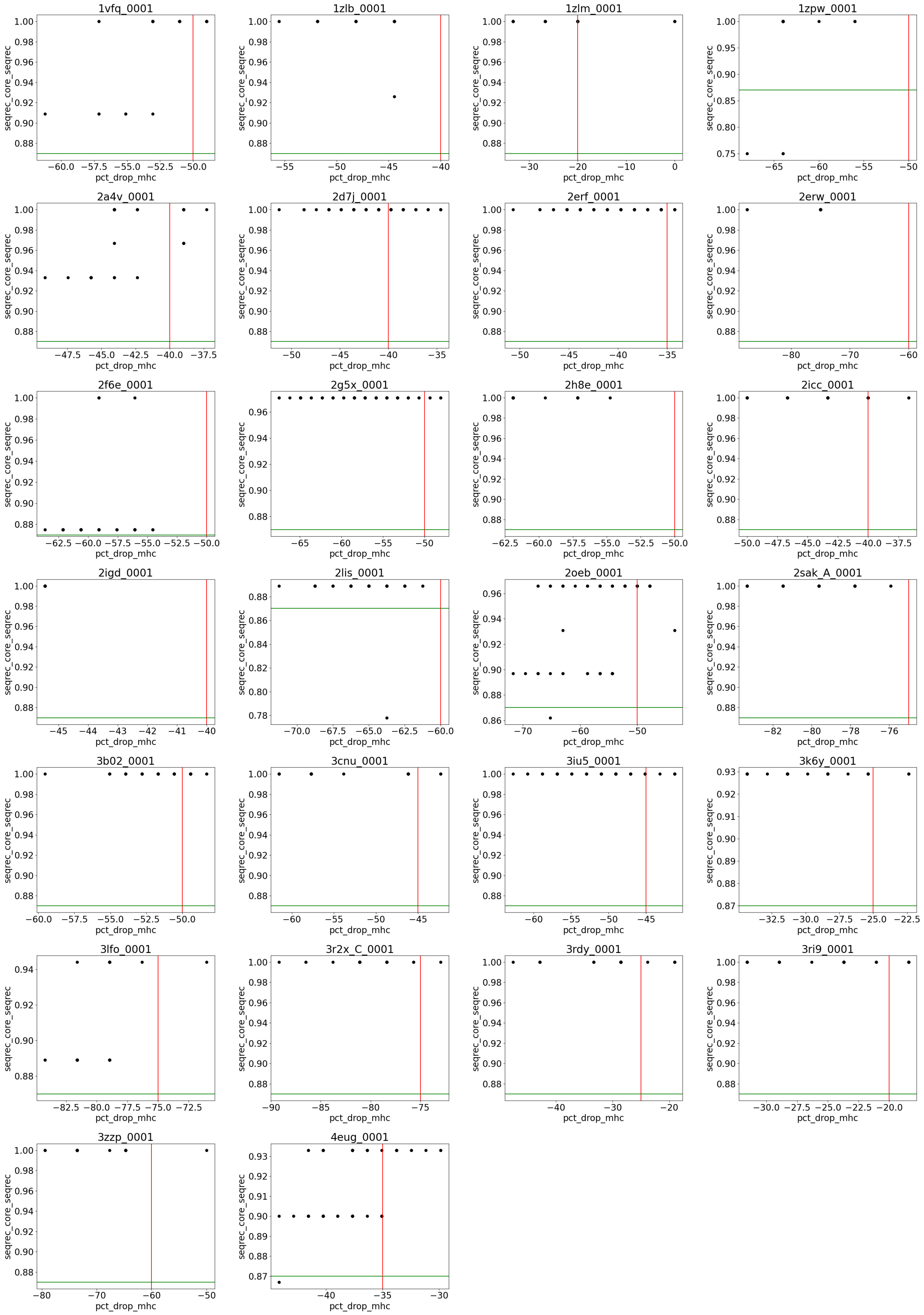

delta_packstat_vs_mhc_epitope

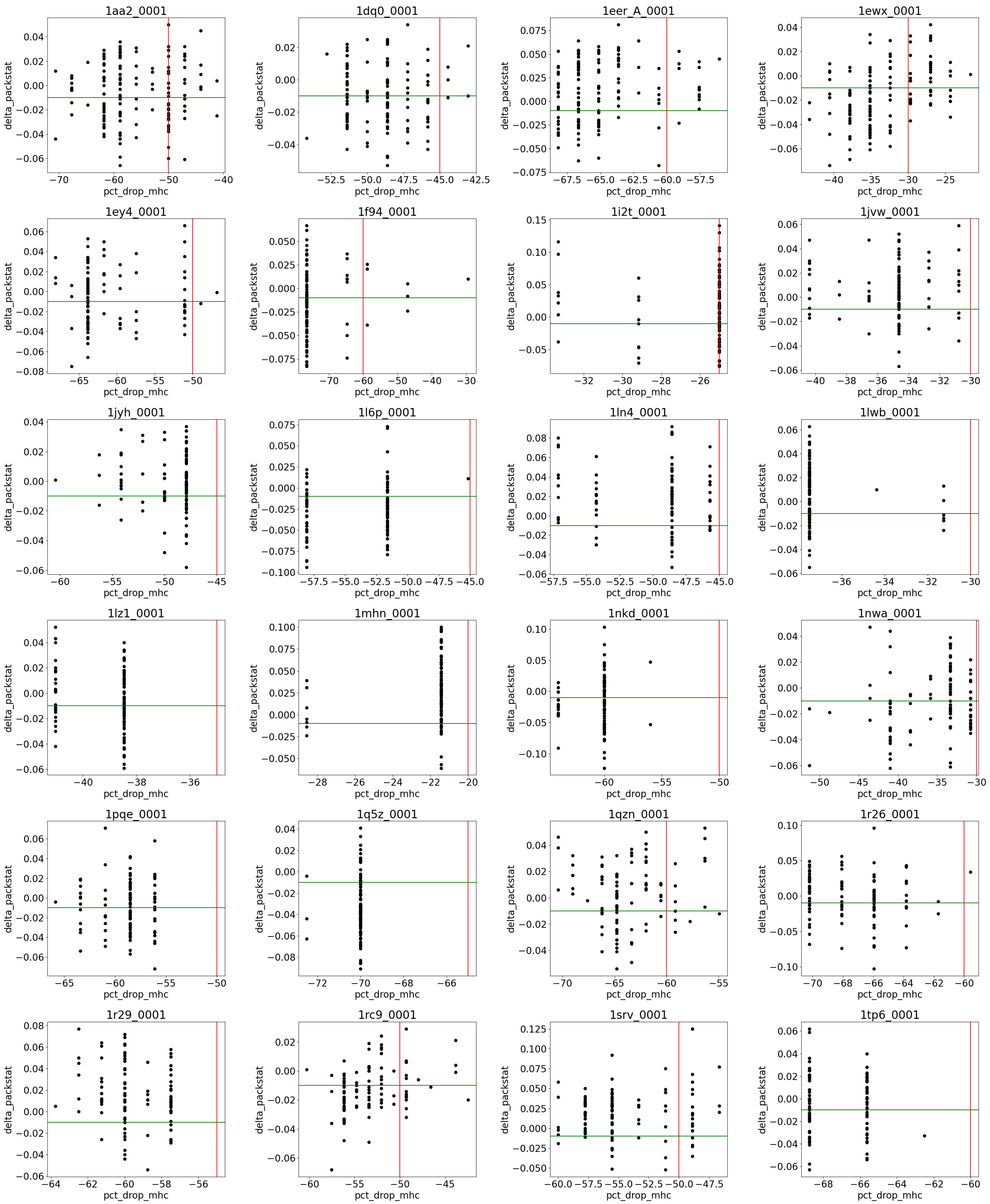
1aa2_0001
1dq0_0001
1eer_A_0001
1ewx_0001
1ey4_0001
1f94_0001
1i2t_0001
1jvw_0001
1jyh_0001
1l6p_0001
1ln4_0001
1lwb_0001
1lz1_0001
1mhn_0001
1nkd_0001
1nwa_0001
1pqe_0001
1q5z_0001
1qzn_0001
1r26_0001
1r29_0001
1rc9_0001
1srv_0001
1tp6_0001
delta_packstat
pct_drop_mhc

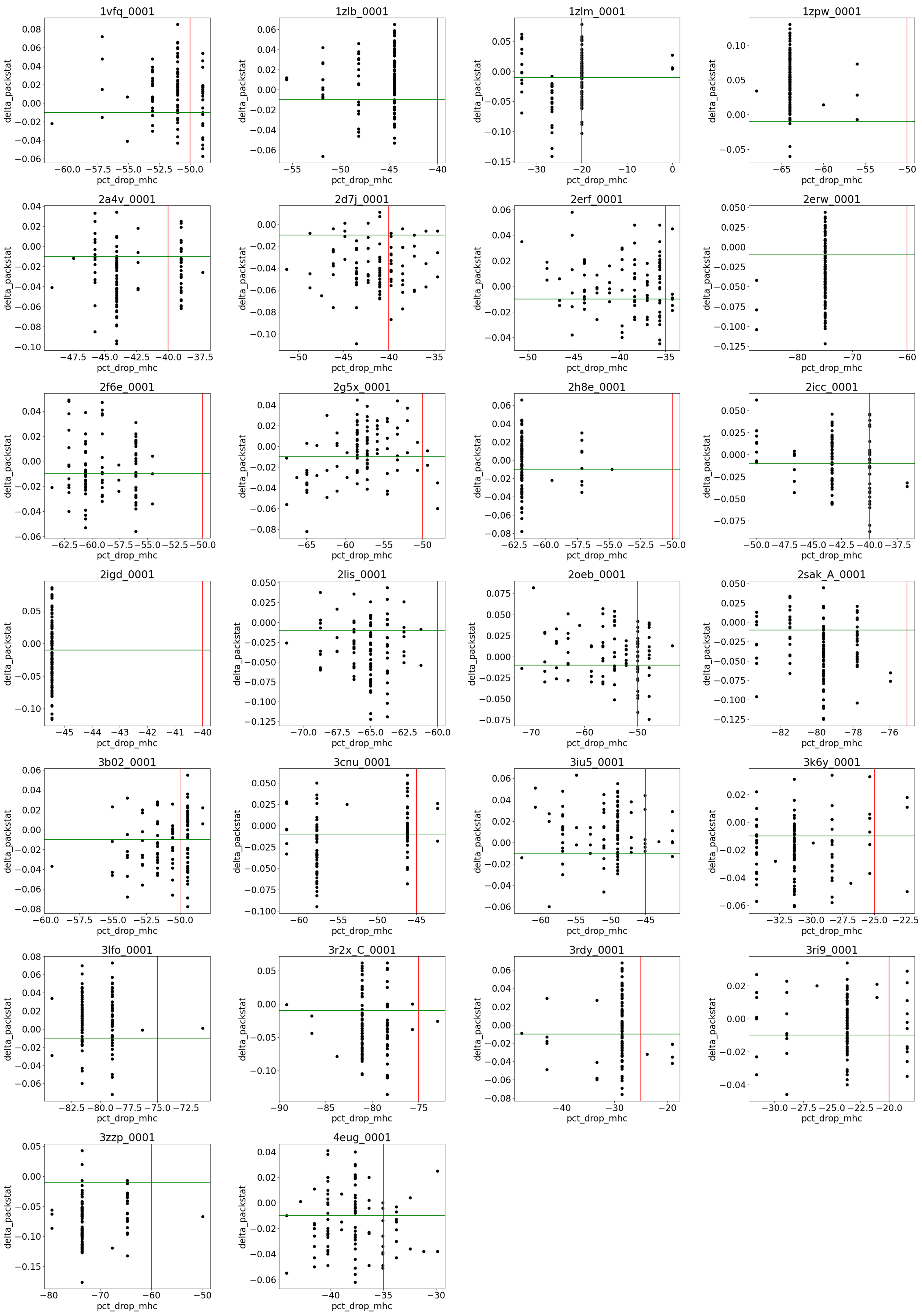

delta_buried_unsat_vs_mhc_epitope

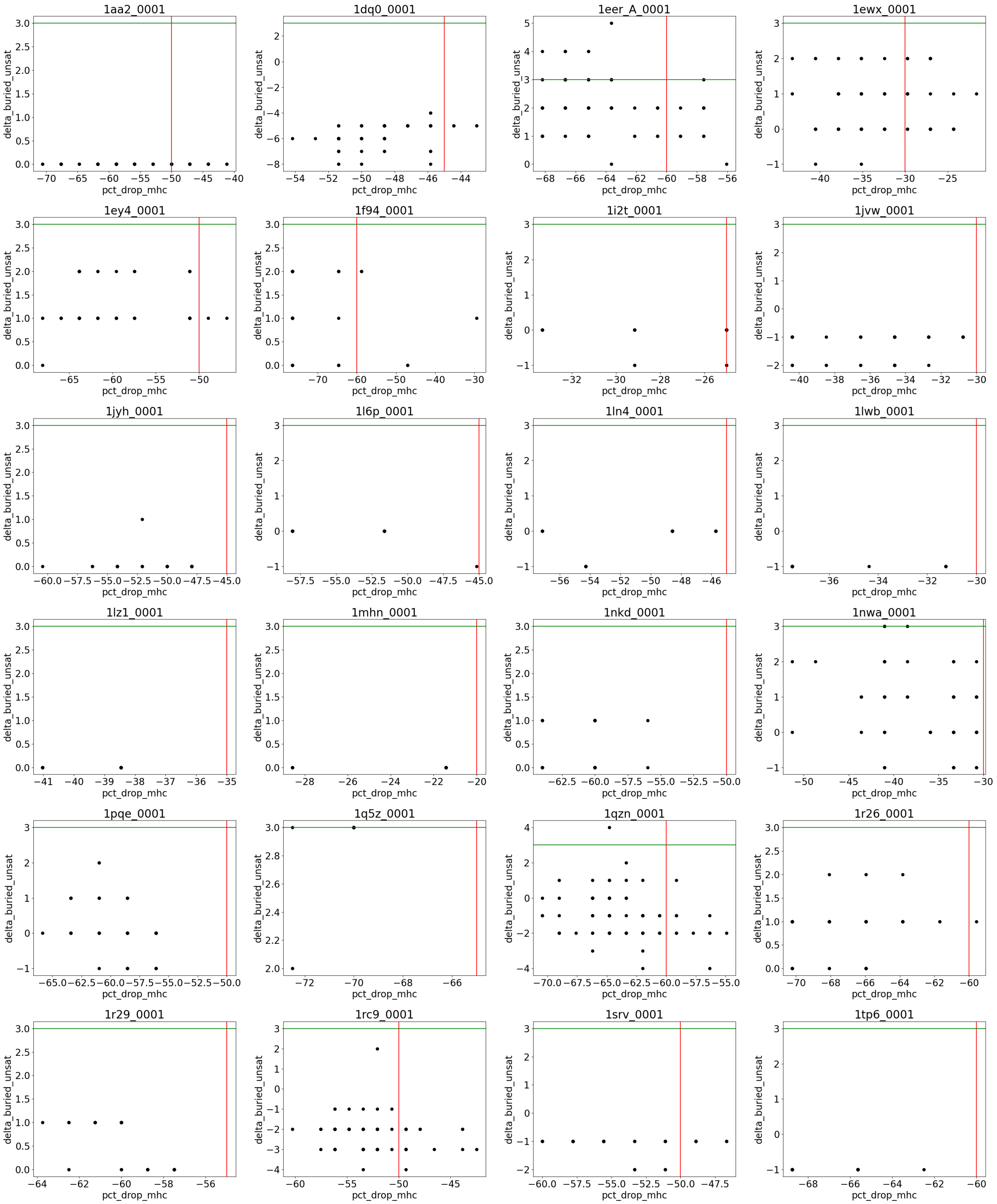
1aa2_0001
1dq0_0001
1eer_A_0001
1ewx_0001
1ey4_0001
1f94_0001
1i2t_0001
1jvw_0001
1jyh_0001
1l6p_0001
1ln4_0001
1lwb_0001
1lz1_0001
1mhn_0001
1nkd_0001
1nwa_0001
1pqe_0001
1q5z_0001
1qzn_0001
1r26_0001
1r29_0001
1rc9_0001
1srv_0001
1tp6_0001
delta_buried_unsat
pct_drop_mhc

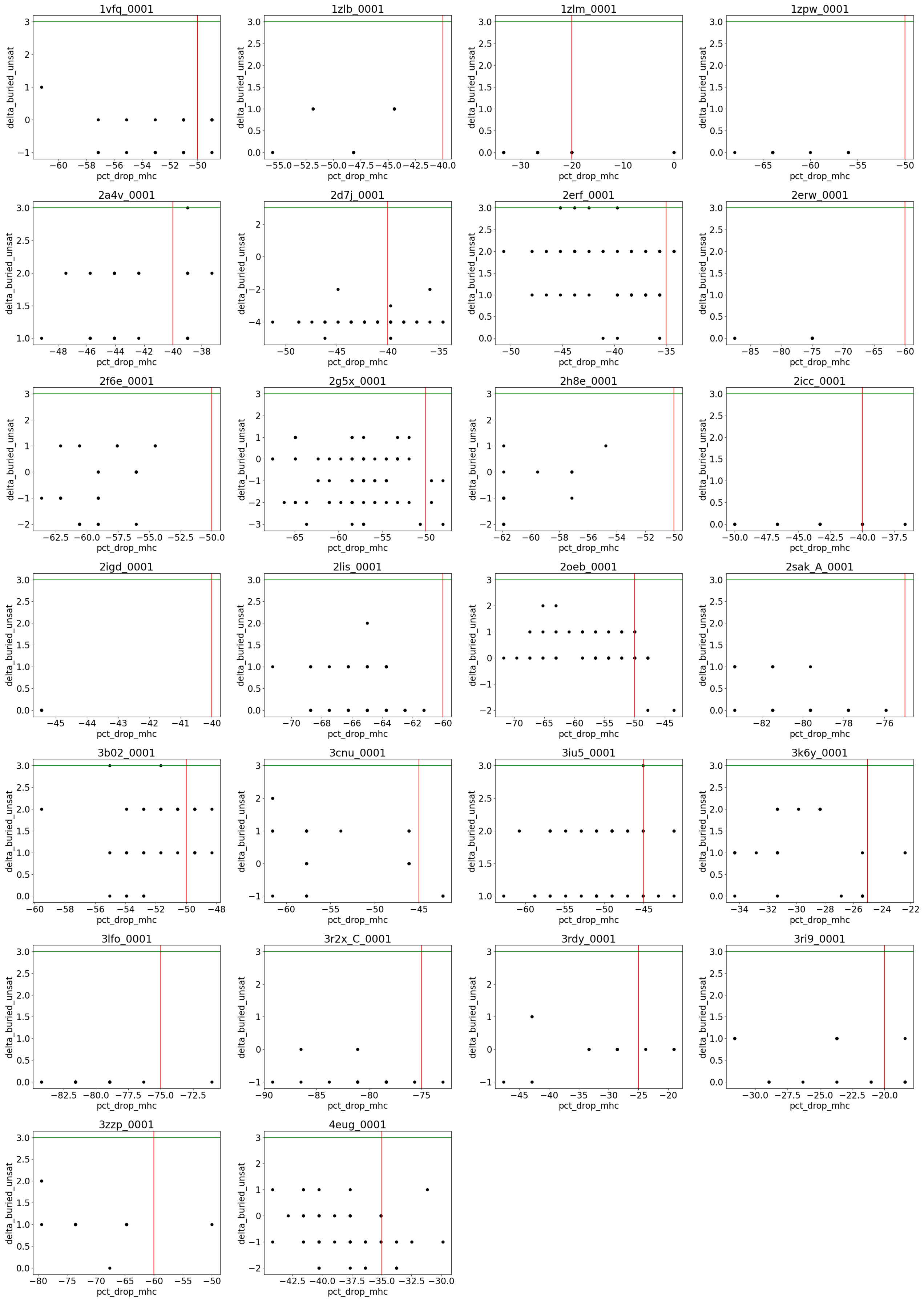

delta_netcharge_vs_mhc_epitope

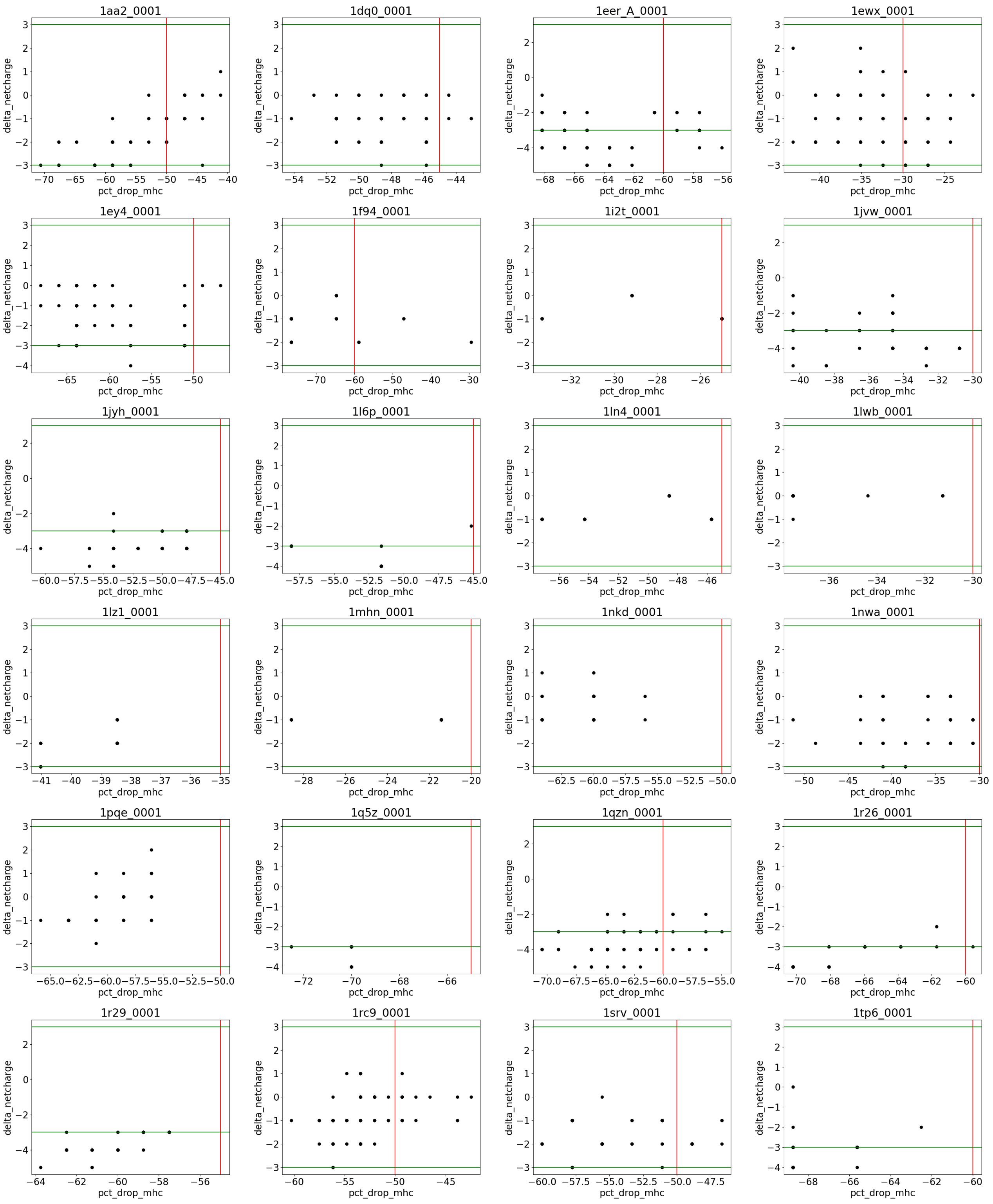
1aa2_0001
1dq0_0001
1eer_A_0001
1ewx_0001
1ey4_0001
1f94_0001
1i2t_0001
1jvw_0001
1jyh_0001
1l6p_0001
1ln4_0001
1lwb_0001
1lz1_0001
1mhn_0001
1nkd_0001
1nwa_0001
1pqe_0001
1q5z_0001
1qzn_0001
1r26_0001
1r29_0001
1rc9_0001
1srv_0001
1tp6_0001
delta_netcharge
pct_drop_mhc

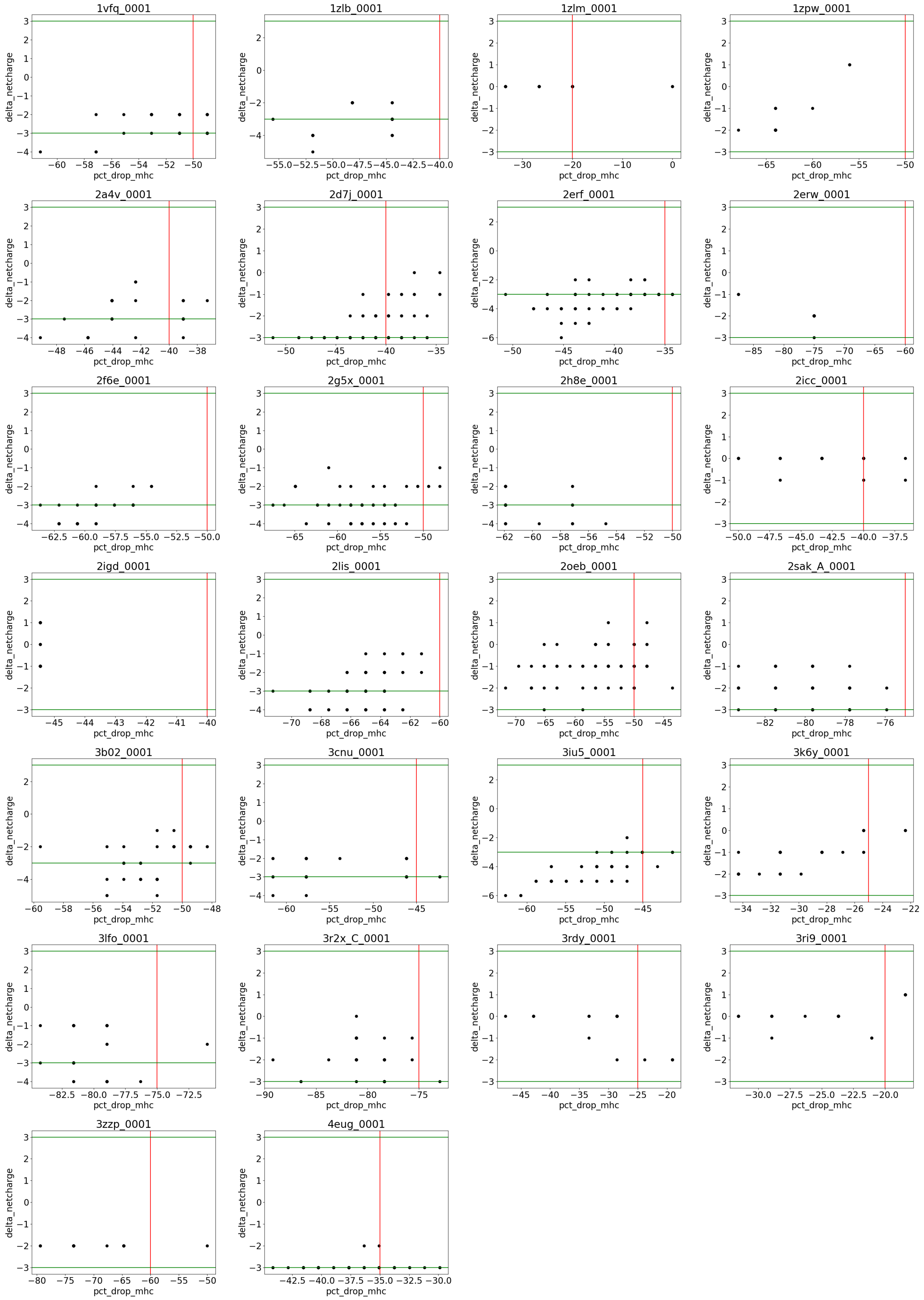

## AUTHOR

This test was set up by Brahm Yachnin (Sagar Khare's lab, Rutgers Universty), in collaboration with Chris Bailey-Kellogg (Dartmouth

College).

brahm.yachnin@rutgers.edu

cbk@cs.dartmouth.edu

khare@chem.rutgers.edu

PURPOSE OF THE TEST

Protein de-immunization, involving the removal of T-cell epitopes that can trigger an immune response, is critical to the success of protein-based therapeutics/biologics. This test is designed to monitor Rosetta's ability to de-immunize proteins while maintaining good structural properties. In addition to watching for a decrease in mhc_epitope score, a ProPred-based metric for how immunogenic the protein is, we are monitoring five additional parameters that track the "goodness" of the resulting structure (see below).

BENCHMARK DATASET

The benchmark dataset includes 50 proteins, which were originally selected for a similar study (Choi et al. (2013) Structure-based Redesign of Proteins for Minimal T-cell Epitope Content. J. Comp. Chem. 34:879-891.), in which a different Rosetta scoreterm (described in the aforementioned paper, but never merged) was used for computational de-immunization. Our scientific test aims to mirror this study using the new (as of 2019) Rosetta implementation of packer-compatible ProPred-based de-immunization.

A manuscript describing this protocol and benchmark is currently (Febuary 2020) in preparation.

The input PDB files were downloaded from the PDB and relaxed using fast_relax with coordinate constraints using the ref2015 scorefunction. The resfiles were derived from PSI-BLAST PSSMs, generated using the online PSI-BLAST tools (version 2.8.1). The default PSI-BLAST settings were used, and three iterations of PSI-BLAST were performed to obtain the PSSM. Resfiles were generated using the mhc_gen_db.py tool located in tools/mhc_energy_tools using the PSSMs as input, as follows:

mhc_gen_db.py --propred --pssm PDB_NAME.pssm --pssm_thresh 1 --res_out PDB_NAME_thresh1.res --pdb PDB_NAME.pdb --firstres NUMBER_OF_FIRST_RESIDUE_IN_PDB

(PSSM thresholds of 2 and 3 were generated in the same way.)

The .comp files, used to setup the AAComposition score, were generated for each PDB file based on the number of native positive and negative charges for that protein.

PROTOCOL

This test designs (all residue fixed backbone design) and minimizes a set of proteins using the ref2015 scorefunction with the MHCEpitopeEnergy scoreterm (mhc_epitope) turned on to perform immune epitope prediction and elimination. The test uses the "base"/default ProPred configuration. In order to follow our recommended settings, FavorNative constraints are turned on, and a PSSM-based resfile is used to restrict design space to evolutionarily probable residues. In addition, the AAComposition scoreterm is used to maintain the number of positive and negative charges in the protein, thereby preventing the explosion of positively and negatively charged residues which is typical in de-immunization protocols. The PSSM was generated using PSI-BLAST, and at each position, any residue type with a score of 1 or higher in the PSSM (plus the native identity) is allowed. A number of quality metrics are evaluated and compared to the native structure after design.

Note that in "debug" mode, the PSSM threshold is increased to 3, which results in a much quicker runtime because of the smaller allowed design space.

The publication of this protocol is currently (February 2020) in preparation.

The protocol takes approximately 2000 CPU hours in release mode, running the entire benchmark with an nstruct of 100.

PERFORMANCE METRICS

First, we assess whether the "degree" of de-immunization is maintained by comparing the distribution of mhc_epitope and delta_mhc_epitope scores to a cutoff determined for each target based on typical results. 90% of decoys should be lower than the preset cutoff. The cutoff (determined per target) is denoted by the ORANGE lines in the plots below.

In addition to mhc_epitope, which is used to measure "how deimmunized" the protein is, we looked at five orthogonal metrics to measure the "goodness" of the resulting designs. We are essentially asking if some of the more de-immunized designs also "good" as evaluated by these metrics. For a subtest to be considered a pass, a certain fraction of poses (determined for each subtest) must have a sufficient percent drop in mhc_epitope score (determined for each target, and indicated by the RED lines in the plots below) AND must ALSO meet the following thresholds (denoted by the GREEN lines in the plots below):

1. total_score (ref2015 without any contributions from constraints) must be in the top 50th percentile of the total_score values obtained during design of that target.

2. Sequence recovery (is the sequence native-like?) must be greater than 80%, and sequence recovery of "core" regions must be greater than 87%.

3. packstat (is the protein well-packed?) must be no more than 0.01 worse than native.

4. Buried unsatisfied hydrogen bonds (are more of these introduced as a result of de-immunization?) must increase by no more than 3.

5. Net charge (does de-immunization of the protein introduce extra charges?) must change by no more than 3 in either direction.

total_score and packstat are measurements of the "goodness" of the protein structure and packing. Sequence recovery is important as we want to minimally disrupt the protein sequence in this design case, as the goal is to maintain structure and activity. As de-immunization tends to replace hydrophobic residues with polar and charged residues, buried unsatisfied hydrogen bonds is a metric used to identify introduction of unsatisfied polar residues in the protein core. Finally, because de-immunization tends to often replace residues with Asp or Glu, net charge is used to monitor if the sequence incorporates a large number of additional charged residues which may destabilize the packing or activity of the protein.

The test passes overall if all subtests pass. Note that while the subtests are classified as passes or fails in debug mode, these results are not meaningful and should be ignored. (The "overall" test will automatically pass in debug mode, as long as the test scripts complete successfully.)

KEY RESULTS

The aim of this test is to ensure that we can continue to get good performance in both protein de-immunization and orthogonal structure quality metrics. We do not have an experimentally validated result to compare against, nor an established computational test set to compare to. The key result should be that we are able to obtain similarly de-immunized proteins in structures that also show high quality metrics (as compared to the native). If future versions of Rosetta fail to obtain structures that are both de-immunized and good quality structures, this implies that the underlying protocol must be modified to restore this balanced behaviour.

The cutoffs that are selected are somewhat arbitrary, aiming to capture the current performance. If performance improves in the future, these cutoffs could be made to be more stringent to reflect this improvement.

In the future, major updates to Rosetta (e.g. a new scorefunction) should be modified within the rosetta_script used in this test to determine if this results in better or worse behaviour during de-immunization. If there is no change or results improve, the test should be permanently modified to make use of the new data.

DEFINITIONS AND COMMENTS

De-immunization, in this context, means the act of removal T-cell epitopes from a protein. With the ongoing rise of biologics in the pharmaceutical industry, as well as in academic research, the ability to de-immunize targets without sacrificing protein structure, function, or stability is an increasingly important field of research. To maximize results, it is critical to remove the strongest epitopes while minimally mutating the native protein.

LIMITATIONS

Because this benchmark is based entirely on computationally derived trends, we do not have the ability to show if we are generating real, active, de-immunized proteins. A case where we assess if Rosetta is able to generate structures that match experimentally validated cases would be a substantial improvement, but would require extensive work on a large number of these targets.

REVISION

revision:61592

test_id: 687687

status: passed

Scientific test: mp_f19_ddG_of_mutation

FAILURES

None

RESULTS

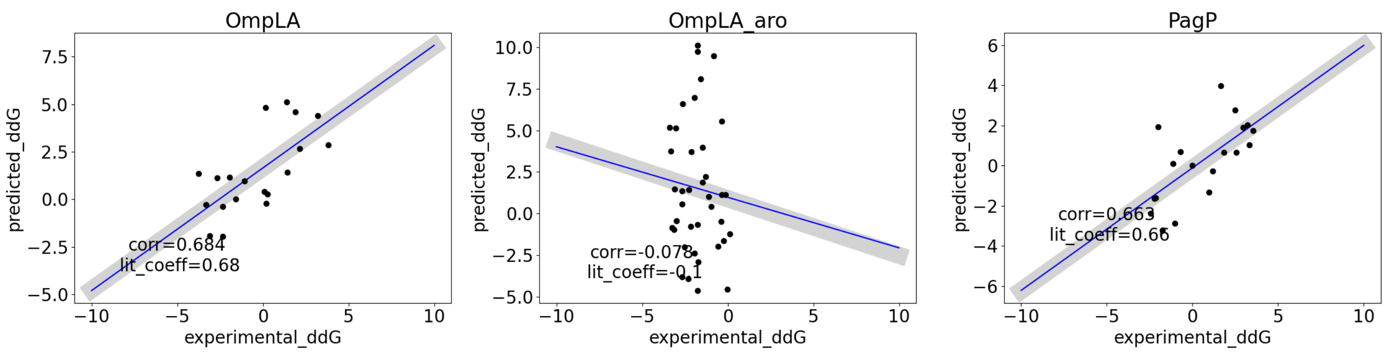

AUTHOR AND DATE

Rebecca F. Alford (ralford3@jhu.edu)

PI: Jeffrey J. Gray (Johns Hopkins ChemBE)

Test created 6/6/19

PURPOSE OF THE TEST

The purpose of this test is to evaluate the scientific performance of franklin2019, the default energy function for membrane protein structure prediction and design. Specifically, this test evaluates the ability of the energy function to reproduce experimentally measured ddG of mutation values.

BENCHMARK DATASET

The dataset includes three sets of ddG measurements taken in two protein scaffolds: the Outer Membrane Protein Phospholipiase A (OmpLA; 1qd6) [1] and the Palmitoyl transferase PagP (3gp6) [3]. The ddG measurements for OmpLA and PagP capture the change in free energy upon mutation from alanine to one of the 19 canonical amino acids at a lipid facing site on the protein. The ddG measurements for OmpLA_aro capture the change in free energy upon mutation from alanine to the aromatic amino acids Trp, Tyr, and Phe [2].

The references for each set of measurements are given here:

[1] Moon CP, Fleming KG (2011) "Side-chain hydrophobicity scale derived from transmembrane protein folding into lipid bilayers" Proc Natl Acad Sci 108(25):10174-7.

[2] McDonald, S. K. & Fleming, K. G. Aromatic Side Chain Water-to-Lipid Transfer Free Energies Show a Depth Dependence across the Membrane Normal. J. Am. Chem. Soc. 138, 7946–7950 (2016).

[3] Marx DC, Fleming KG (2017) "Influence of Protein Scaffold on Side-Chain Transfer Free Energies" Biophysical Journal 113(3):597-604

PROTOCOL

This test uses the ddG prediction protocol described in Alford & Koehler Leman et al. [3]. Here, a mutation is introduced at the host site and the side chains are optimized within 8Å of the mutated residue. Then, the ddG of mutation is computed as the difference in energy between the mutant and native conformations.

The ddG of mutation protocol is described in:

(Alford RF, Koehler Leman J, Weitzner BD, Duran AM, Tiley DC, Elazar A, Gray JJ (2015) "An integrated framework advancing membrane protein modeling and design" PLoS Comput. Biol. 11(9):e1004398.)

The test results for OmpLA and PagP for franklin2019 are described in:

(Alford RF, Fleming PJ, Fleming KG, Gray JJ (2019) "Protein structure prediction and design in a biologically-realistic implicit membrane" Submitted.)

The test results for OmpLA_aro for franklin2019 are described in:

(Alford, R. F. & Gray, J. J. Diverse scientific benchmarks for implicit membrane energy functions. bioRxiv [Preprint] 2020.06.23.168021 (2020). doi:10.1101/2020.06.23.168021)

The test should take approximately 1 CPU hour.

PERFORMANCE METRICS

The performance metric for this test is the Pearson correlation coefficient between the experimentally measured and predicted ddG of mutation values. Pass/fail is defined by the MAE (mean absolute error) falling within +/- 0.5 REU of the values established in Alford et al. 2019.

KEY RESULTS

The Pearson correlation coefficients for Franklin2019 ddG predictions are R = 0.68 / -0.1 / 0.66 for OmpLA, OmpLA_aro and PagP, respectively. These results are described in detail in the benchmark paper Alford et al. 2019.

DEFINITIONS AND COMMENTS

The starting structures were downloaded from the Orientations in Membrane proteins database and the DLPC lipid composition parameters were used for calculations.

LIMITATIONS

It should be noted that these ddG measurements are high quality data. This is because the measurements were taken in a reversibly folding scaffold and the lipid composition between the experiment and similation are consistent. Possible improvements are to use the MAE of the ddG values directly as a quality metric instead of just for the correlation. Further possible improvements are discussed in Chapter 4 of Rebecca's PhD thesis.

REVISION

revision: 61711

test_id: 712002

Scientific test: mp_f19_decoy_discrimination

FAILURES

None

RESULTS

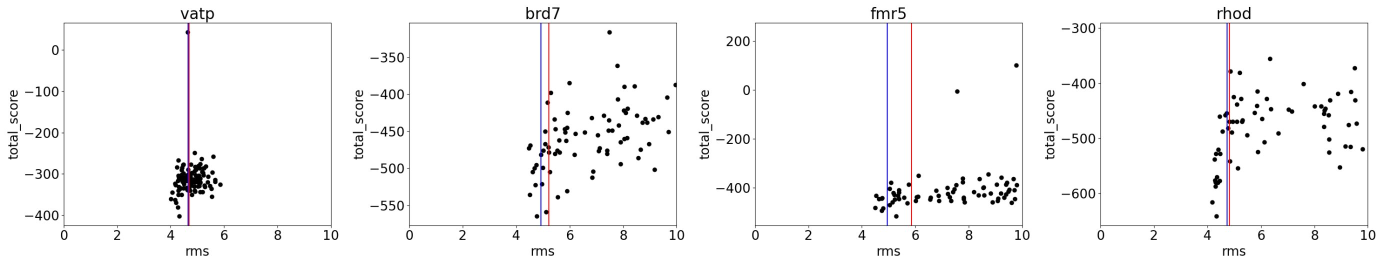

AUTHOR AND DATE

Rebecca F. Alford (ralford3@jhu.edu)

PI: Jeffrey J. Gray (Johns Hopkins ChemBE)

Test created 6/6/19

PURPOSE OF THE TEST

The purpose of this test is to evaluate the scientific performance of franklin2019, the default energy function for membrane protein structure prediction and design. Specifically, this probes the ability of franklin2019 to discriminate near-native from non-native decoys.

BENCHMARK DATASET

The benchmark dataset includes four targets: V-ATPase (VATP; 2bl2), Bacteriorhodopsin (BRD7; 1py6), Fumarate Reductase (FMR5; 1qla), and Rhodopsin (RHOD; 1u19). For each target, we are using two sets of decoys. The first set of decoys were generated by Yarov-Yaravoy et al. 2006 [1] through ab initio folding and include 5,000 models per target. These decoys are between 5-40Å RMSD from the native crystal structure. The second set of decoys were generated by Dutagaci et al. 2017 [2] through molecular dynamics simulations and include between 75-110 decoys per target. These decoys are between 1-11 RMSD from the native crystal structure.

To balance the data, we randomly selected a subset of 100 decoys from the low-resolution set. The same set of random 100 models is used for all testing.

References for datasets:

1. Yarov-Yaravoy V, Schonbrun J, Baker D (2006) "Multipass membrane protein structure prediction using Rosetta" Proteins 62(4):1010-25

2. Dutagaci B, Wittayanaraku K, Mori t, Feig M (2017) "Discrimination of native-like states of membrane proteins with implicit membrane-based scoring functions" 13(6):3049-3059.

The input files are the PDB coordinate files for each decoy and a span file generated using the mp_span_from_pdb application. The PBD coordinates were downloaded from the Orientations of Proteins in Membranes database.

PROTOCOL

Each decoy is refined using the RosettaMPRelax protocol with the franklin2019 energy function. The franklin2019 energy function is described in (Alford, R. F., Fleming, P. J., Fleming, K. G. & Gray, J. J. Protein Structure Prediction and Design in a Biologically Realistic Implicit Membrane. Biophys. J. 118, 2042–2055 (2020)) and the RosettaMPRelax protocol is described in (Alford RF, Koehler Leman J, Weitzner BD, Duran Am, Tilley DC, Elazar A, Gray JJ (2015) "An integrated framework advancing membrane protein modeling and design" PLoS Comput Biol 11(9): e1004398.)

This benchmark test takes approximately 2,000 CPU hours.

PERFORMANCE METRICS

We use the Boltzmann-weighted RMS (Wrms) metric to evaluate decoy discrimination. We chose this metric because it identifies the average RMS, accounting for the likelihood of those structures occurring in nature according to their energies. This metric is further described in:

(Bhardwaj G, Mulligan VK, & Bahl CD et al. (2016) "Accurate de novo design of hyperstable constrained peptides" Nature 538(7625):329-335)

Pass/fail is defined by comparison of calculated Wrms values for this benchmark with previously established values. The test passes if Wrms is within 0.5Å of the established value.

In the plot generated by this test, the sampled RMS is shown as a blue line and the Wrms is shown as a red line.

KEY RESULTS

The calculated Wrms values are compared with baseline Wrms values from the soluble ref2015 energy function and previous versions of the membrane energy function.

DEFINITIONS AND COMMENTS

The membrane normal and center, in addition to the coordinate frame setup are described in:

(Alford RF, Koehler Leman J, Weitzner BD, Duran Am, Tiley DC, Elazar A, Gray JJ (2015) "An integrated framework advancing membrane protein modeling and design" PLoS Comput. Biol. 11(9):e1004398.)

LIMITATIONS

The PNear and Wrms metrics evaluate discrimination based on the root-mean-squared-deviation from the coordinates of the native crystal structure. While this is a good general metric for proteins, it looses information about the membrane. I am also not in favor or rerunning Rosetta scoring for the analysis step, this should either be done as a second step during the submission or directly in the XML script. One inconsistency here is that the fa_water_to_bilayer score term has a sizeable difference between the scoring and re-scoring step, which needs to get looked into.

Further Lactose Permease (LTPA; 1pv6) was taken out, not sure why.

REVISION

revision: 61711

test_id: 711948

Scientific test: mp_f19_energy_landscape

FAILURES

None

RESULTS

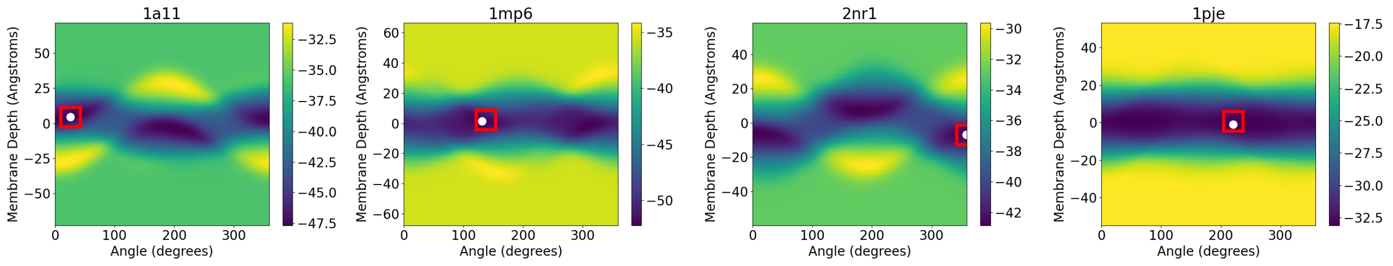

AUTHOR AND DATE

Rebecca F. Alford (ralford3@jhu.edu)

PI: Jeffrey J. Gray (Johns Hopkins ChemBE)

Test created 6/6/19

PURPOSE OF THE TEST

The purpose of this test is to evaluate the scientific performance of franklin2019, the default energy function for membrane protein structure prediction and design. Specifically, the computed insertion depths and tilt angles of 5 peptides are compared to experimental values.

BENCHMARK DATASET

The benchmark dataset includes five transmembrane peptides. Each peptide has an NMR structure and a tilt angle measured by solid-state NMR. The first four targets (2nr1, 1a11, 1mp6, and 1pje) were taken from [1]. The fifth target, WALP23 was taken from [2].

1. Ulmschneider MB, Sansom MSP, Di Nola A (2005) "Evaluating tilt angles of membrane-associated helices: comparison of computational and NMR techniques" Biophys J 90(5): 1650-1660

2. Holt A, Koehorst RBM, Rutters-Meijneke T, Gelb MH, Rijkers DTS, Hemminga MA, Antoinette-Killian J (2009) "Tilt and rotation angles of a transmembrane model peptide as studied by fluorescence spectroscopy" Biophys J 97(8): 2258-2266

The input files are the PDB coordinates for each peptide downloaded from the Protein Databank. Instead of spanfiles, we specify the "single_TM_mode" option, indicating that the protein is a single-TM segment. The PDB coordinate files were cleaned using the `clean_pdb.py` script.

PROTOCOL

This scientific benchmark identifies low-free energy peptide orientation by calculating an energy landscape: a mapping between all possible orientations relative to the membrane and their energies. Orientation is defined by

two coordinates: distance between the center of the membrane and center-of-mass of the peptide, and (2) angle between the membrane normal and helical axis. The protocol first applies side-chain packing and minimization to resolve steric clashes in the peptide structure. Then, rigid body moves are applied to sample all combinations of angle and depth values. Membrane depth values are sampled between -60Å and 60Å with a 1Å step size and tilt angles are sampled between 0-360 degree with a 1 degree step size.

For each peptide, the protocol takes approximately 1-2 CPU hours (for a total 10 CPU hours).

The membrane energy landscape sampling protocol is described in:

(Alford, R. F., Fleming, P. J., Fleming, K. G. & Gray, J. J. Protein Structure Prediction and Design in a Biologically Realistic Implicit Membrane. Biophys. J. 118, 2042–2055 (2020).)

PERFORMANCE METRICS

There are two key performance metrics for this test. The first is the water-to-bilayer partitioning energy of the peptide, calculated as the energy difference between a peptide submerged in water and a peptide oriented vertically in the membrane. The test passes if the calculated partitioning energy is within +/- 1REU of the calculated value from the franklin2019 score function.

The second metric is the minimum energy tilt angle and membrane depth. The test passes if the calculated orientation is within +/- 2Å and +/- 10˚ of the experimentally determined orientation as defined in the cutoffs file.

KEY RESULTS

The white dots in the plots are the computed minima in the energy landscape, not the experimentally determined values. The result.txt file contains which measurements are failing, if any. The water-to-bilayer partitioning energy, delta G should be less than 0 for all peptides to ensure that the peptides partition into the membrane. Further, the low-energy peptide orientations should generally be between 0-45 degrees tilted relative to the membrane normal and no more than +/- 5Å from the membrane center. Exceptions here are WALP23 with an 82˚ angle and 2nr1 with a distance of 6.7Å from the membrane center. Note that these values have been experimentally determined.

DEFINITIONS AND COMMENTS

The membrane normal and center, in addition to the coordinate frame setup are described in:

Alford RF, Koehler Leman J, Weitzner BD, Duran AM, Tiley DC, Elazar A, Gray JJ (2015) "An integrated framework advancing membrane protein modeling and design" PLoS Comput. Biol. 11(9):e1004398.

LIMITATIONS

Better quantification metrics for the full energy landscape would be beneficial. WALP23 was taken out because it fails hard in angle_min and moderately in z_min. The data remains in the scientific test code even though it is neither tested nor plotted. It would be beneficial if this could be looked at.

REVISION

revision: 61711

test_id: 711957

Scientific test: mp_f19_sequence_recovery

FAILURES

None

RESULTS

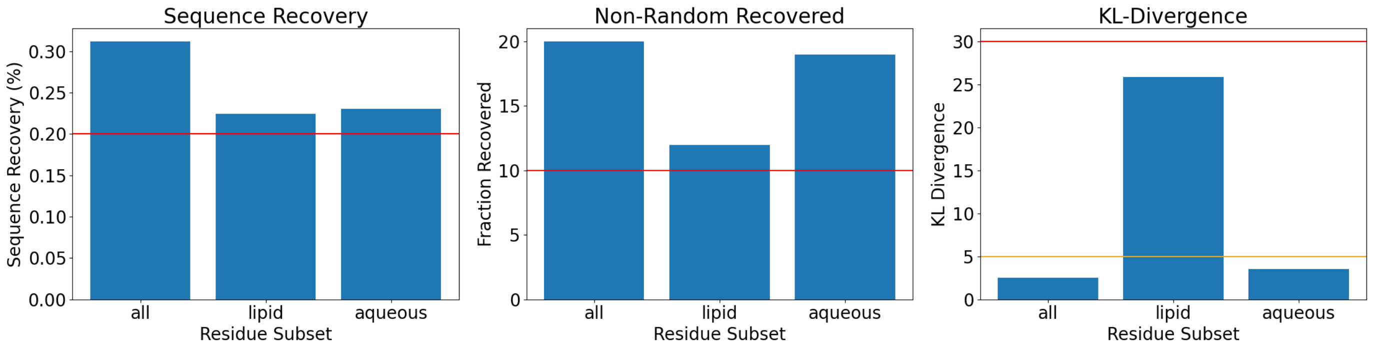

AUTHOR AND DATE

Rebecca F. Alford (ralford3@jhu.edu)

PI: Jeffrey J. Gray (Johns Hopkins ChemBE)

Test created 6/6/19

PURPOSE OF THE TEST

The purpose of this test is to evaluate the scientific performance of franklin2019, the default energy function for membrane protein structure prediction and design.

BENCHMARK DATASET

The benchmark dataset includes 130 alpha-helical and beta-barrel transmembrane proteins, with <25% sequence identity and better than 3.0Å resolution. The dataset is a subset of proteins from [1] which have assigned lipid compositions. The dataset modifications are detailed in [2].

([1] Koehler Leman J, Lyskov S, Bonneau R (2017) "Computing structure-based lipid accessibility of membrane proteins with mp_lipid_acc in RosettaMP" BMC Bioinformatics 18:115)

([2] Alford, R. F., Fleming, P. J., Fleming, K. G. & Gray, J. J. Protein Structure Prediction and Design in a Biologically Realistic Implicit Membrane. Biophys. J. 118, 2042–2055 (2020).)

The inputs are PDB coordinate files and spanning topology definition files for each protein. The PDB coordinate input files were downloaded from the Orientations of Proteins in Membranes Database. The spanning topology definition files were generated using the mp_span_from_pdb application.

PROTOCOL

To evaluate sequence recovery, the fixed-backbone Rosetta design protocol is used to search for low energy sequences. The protocol is described in:

(Leaver-Fay A, O'Meara MJ, Tyka M, Jacak R, Song Y, Kellogg EH, Thompson J, Davis IW, Pache RA, Lyskov S, Gray JJ, Kortemme T, Richardson JS, Havranek JJ, Snoeyink J, Baker D, Kuhlman B (2013) "Scientific benchmarks for guiding macromolecular energy function improvement" Methods in enzymology 523:109-143)

The benchmark will take approximately 500 CPU hours.

PERFORMANCE METRICS

To evaluate the performance of this benchmark, we computed three metrics. The first metric is sequence recovery which is the fraction of correctly designed positions relative to the number of available positions. Realistic energy functions will maximize the recovery rate, with ideal values ranging from 35-50%. Note, perfect sequence recovery is seldom possible because other factors constrain protein sequences including functional and evolutionary pressures.

The second metric is the recovery rate for individual amino acids relative to the background probability of guessing a random amino acid type (1 in 20 types, or 5%). Here, a higher value is better.

The third metric is the Kullback-Leibler divergence which is a measure of the divergence of the amino acid distribution in the designed sequences from the distribution in the native sequences. Unlike recovery and non-random rates, the goal is to minimize the KL-divergence. Ideal values for a membrane protein set are under 5.0, delineated by the yellow solid line on the plot.

For sequence recovery, pass/fail is defined by comparing newly computed with established values computed in [Alford et al. 2020: Protein structure prediction...], which 0.2. For Non-random recovery, a passing value is greater than 10%. A KL divergence failure is defined by a value < 5.0 for the 'all' subset and if both subsets lipid and aqueous have a KL divergence < 5.0

KEY RESULTS

The key results of this scientific test are twofold:

(1) Sequence recovery is high for all amino acid types, not just non-polar amino acids in the transmembrane region

(2) The fraction of amino acid types recovered with rates higher than random, Naa. Naa is > 75%, compared with older energy functions mpframework_fa_2007 (Barth et al. 2007) and mpframework_smooth_fa_2012 (Yarov-Yaravoy et al. 2006) for which Naa was generally less than 50%. This previously resulted in design skewed toward nonpolar amino acids, rather than sampling from a diverse palette of chemistries.

DEFINITIONS AND COMMENTS

The transmembrane, interface, and bulk solvent, as well as buried vs. surface exposed criteria are described in the following paper:

(Alford, R. F., Fleming, P. J., Fleming, K. G. & Gray, J. J. Protein Structure Prediction and Design in a Biologically Realistic Implicit Membrane. Biophys. J. 118, 2042–2055 (2020).)

LIMITATIONS

It would be great to analyze the data without a Rosetta executable, which takes longer to debug.

In general, the benchmark should be more balanced between alpha-helical and beta barrel membrane proteins. The dataset is currently ~25% beta-barrel proteins.

REVISION

revision: 61711

test_id: 711999

Scientific test: mp_dock

FAILURES

None

RESULTS

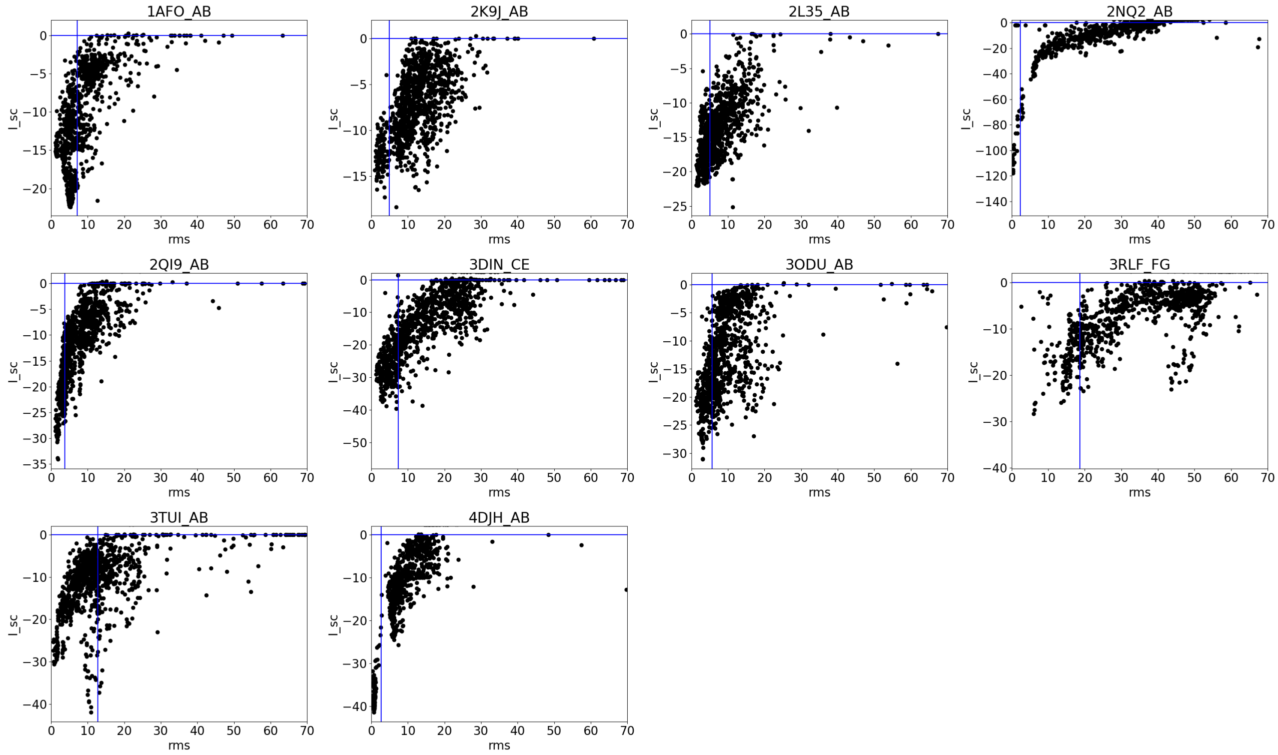

AUTHOR AND DATE

The benchmark was set up by Julia Koehler Leman (julia.koehler.leman@gmail.com) in March 2019.

The research was performed in Jeff Gray's lab, but Julia Koehler Leman is now in the lab of Richard Bonneau.

PURPOSE OF THE TEST

We test whether we can recapitulate binding interfaces of proteins inside the membrane. For this, we run local docking with the membrane scorefunction from RosettaMP.

BENCHMARK DATASET

The benchmark set contains 10 test proteins that were run for mp_dock from the RosettaMP framework paper (Alford, Koehler Leman et. al, PlosCompBio, 2015). Note that the mp_dock application runs local docking (NOT global docking) and the mp_dock application could be further optimized. The paper describes mp_dock and other applications as a proof-of-concept!!!

Input PDBs were downloaded from the PDBTM database (which have better membrane embedding than PDBs from the OPM database). This means that the proteins are transformed into membrane coordinates and have the correct biomolecular assembly, even though we are only looking at dimers for this application. PDBs were cleaned using clean_pdb.py from the Rosetta/tools/protein_tools/scripts directory. Spanfiles were created using the mp_span_from_pdb application in Rosetta.

The PDB files were used as natives for RMSD calculations. Additionally, we created prepacked files using the docking_prepack application in Rosetta, picking the top-scoring model from 10 prepacked decoys. Detail command lines are described in the Supplement to (Alford, Koehler Leman et. al, PlosCompBio, 2015).

PROTOCOL

The mp_dock application uses the docking_protocol but adjusted with the membrane framework RosettaMP. mp_dock runs local docking with a docking perturbation of 3A translation and 8 degrees of rotation. We create 1000 models per protein to sample the interface.

[Note that the natives shouldn't have a membrane residue (MEM), otherwise Rosetta crashes during RMSD calculation.]

On average for this benchmark set, mp_dock creates a decoy in ~70s; that is dependent on size of the protein. That makes 70s x 10 proteins x 1000 decoys = 700,000s which are <200 CPU hours.

PERFORMANCE METRICS

We use the interface score vs. RMSD. Interface score has been found to be more predictive than the total Rosetta score. Alternatively, one can also look at the fraction of native contacts (Fnat). Various docking metrics (also the interface RMSD) are output into the score file. The thresholds for various docking metrics can be found in CAPRI papers.

We define the modeling a success if the the top10 models (out of a 1000) are below a defined RMSD threshold. The RMSD threshold was defined by taking the mean RMSD of the top10 models from the first run and adding 2A.

KEY RESULTS

We use crystal structures as natives for comparison, another general method for MP docking does not really exist - except maybe a specialized tool.

Docking with this proof-of-concept application is easier for smaller systems and more difficult for larger protein or smaller interfaces. Soluble chains contribute to the interface, which can make docking easier, i.e. give more of a funnel.

1afo and 2k9j are single TM helices, they are easier to dock

2l35 is a double helix docking to a single TM helix. There is a disulfide bond connecting the N-termini of the two partners which leads to incredibly low I_sc and large RMSDs

2nq2 actually has 4 chains, for this applications, chains A+C and chains B+D were combined to A and B, respectively. Original chains C and D are the soluble parts of this dimer which create a larger docking interface and for docking results for the paper gave a really nice funnel. Removing chains C and D and therefore the soluble interface might make it more difficult to find a good docking interface, but this hypothesis was not tested.

2qi9 is a multi-helix homodimer

3din has a partner with a long loop that interacts with the top of the other partner

3odu GPCR with a small interface

3rlf very intertwined interface, which is hard to find, therefore RMSDs are large

3tui is a V-shaped transporter with an open and closed conformation. The Xray structure is in the open conformation but the closed conformation has been found to score better in Rosetta, presumably because it has a larger interface

4djh another GPCR with a small interface

DEFINITIONS AND COMMENTS

LIMITATIONS

The mp_dock app needs to be improved and to benchmark that properly, a larger dataset is needed. From that, a collection of good, intermediate and outlier proteins should be picked for continuous scientific benchmarking. The benchmark could further be improved by using ensemble docking or considering flexibility or using homology models instead of crystal structures.

TODO: look at Fnat (=fraction of native contacts): for 3tui, the V-shaped transporter, the best RMSD structure is around 10A, which looks like a bad prediction. However, the Fnat for this is around 80%, which means most of the native interface has been found.

We can only dock dimers, not multi-mers. Multi-body docking is currently not implemented.

REVISION

revision:61589

test_id: 687070

status: passed

Scientific test: mp_domain_assembly

FAILURES

None

RESULTS

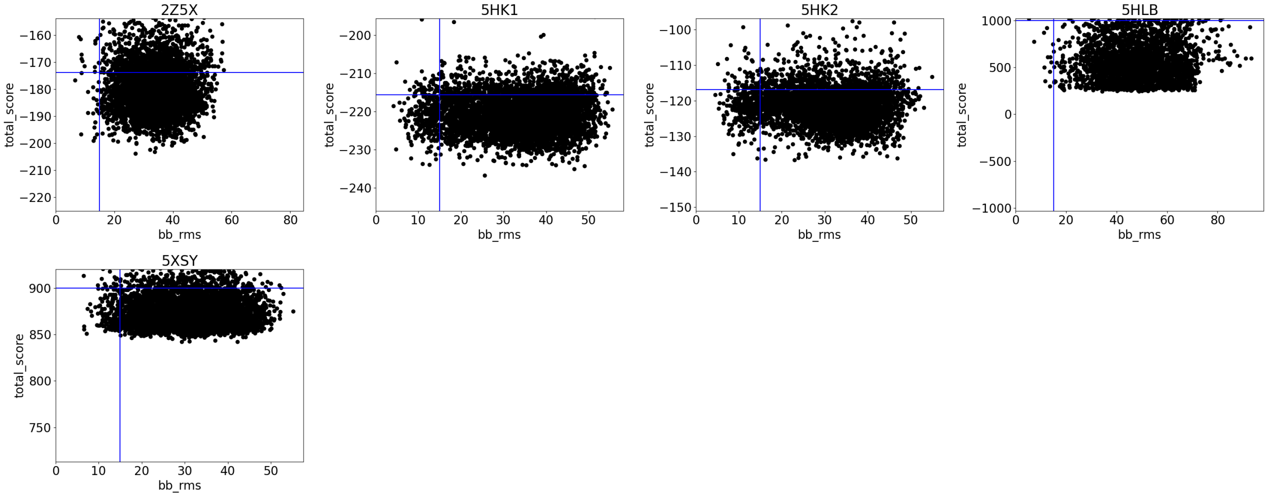

AUTHOR AND DATE

The benchmark was set up by Julia Koehler Leman (julia.koehler.leman@gmail.com) in March 2019.

The PI is Richard Bonneau.

PURPOSE OF THE TEST

This is the first method for domain assembly for membrane proteins. You can give it structures or models of protein domains and a fasta sequence and it assembles the protein into a full-length model. There aren't a lot of structures of full-length membrane proteins, this is why the benchmark set only consists of 5 proteins so far. Also, since the domains are mostly connected by flexible linkers, the energy landscape is pretty flat along those linkers, so the only thing we can test so far is how well we sample. We basically only check whether we can sample a model <10A RMSD. Also, the RMSD values get pretty large pretty quickly, because only the TM domains are superimposed, which in this benchmark set are single TM helices, which make up a minority of the protein in terms of number of atoms.

BENCHMARK DATASET

The benchmark set consists of 5 proteins - the benchmark set, method and command lines are published in (Koehler Leman & Bonneau, Biochemistry, 2017).

Structures were downloaded from the PDBTM database, where the proteins are transformed into the membrane coordinate system. We included only structures without gaps as the fasta files are created from the ATOM lines in the PDB. Structures were cleaned, the spanfile was created with mp_span_from_pdb, the membrane embedding was optimized with mp_transform, a fasta file was created from the ATOM lines, and the fasta file was used to pick fragments using Robetta, excluding homologues. These structures are the natives we compare our models to.

We then removed a few residue linkers and split the PDB files into TM domain and soluble domain. This was done via visual inspection. Linker lengths are:

2z5x: 10 res

5hk1: 6 res

5hk2: 6 res

5hlb: 9 res

5xsy: 9 res

These are used as input files. Input files for the protocol are:

-in:file:fasta 2Z5X_tr_A.fasta # fasta file

-in:file:frag3 2Z5X.frags.3.200_v1_3 # 3-residue fragments

-in:file:frag9 2Z5X.frags.9.200_v1_3 # 9-residue fragments

-in:file:native 2Z5X_tr_A_opt.pdb # native for RMSD calculation (this is done without superposition, i.e. only the TM domain remains superimposed)

-mp:setup:spanfiles 2Z5X_tr_A.span # required for RMSD calculation with native

-mp:assembly:poses 2Z5X_tr_A_opt_sol.pdb 2Z5X_tr_A_opt_tm.pdb # structures of the input domains

PROTOCOL

The protocol is described in detail in (Koehler Leman & Bonneau, Biochemistry, 2017). Briefly, it starts with the TM domain embedded in the membrane, linker residues are added towards the N-terminus, then the N-terminal domain is added, then linker residues towards the C-terminus is added, then the C-terminal domain is added to create the full-length pose. Note that this benchmark set contains only structures with one soluble domain but the protocol works with soluble domains at either terminus (it should also work with a more domains on either side, like beads on a string, the TM domain somewhere in the middle). There is an optional refinement step that runs after domain assembly, but this step was forgone for this benchmark set because of runtime constraints. We create 5000 models for each protein.

For this benchmark set, a model is generated in ~100s. 100s x 5 proteins x 5000 models = 2.5 megaseconds is <700 CPU hours.

PERFORMANCE METRICS

Since the energy landscape of flexible linkers is quite flat, it is difficult to identify a near-native model by score alone. Relaxed natives have scored better than built models, as shown in the paper, figure 2, but sampling so close to the native remains a challenge. We therefore check for RMSDs<15A for at least 2 of the models, which is 0.06%. For score, we ask whether 3 of the models are below the score cutoff which was set from the avg score during the first run, then adjusted visually. RMSD values remain large due to the lever arm effect. Cutoffs were defined by running the protocol a couple of times and adjusting the cutoffs for the test to pass.

KEY RESULTS

The proteins are all single TM helix proteins with a large soluble domain - we compare to crystal structures from the PDB. 5HK1 and 5HK2 are basically the same protein with slightly different relative domain orientations. Please also compare to figure 2 in (Koehler Leman && Bonneau, Biochemistry, 2017).

Since the energy landscape is relatively flat, we don't see a pronounced funnel, unless you include relaxed natives into the plot.

DEFINITIONS AND COMMENTS

The protocol is currently being improved for modeling dimers and all kinds of combinations of soluble and TM domains; it also includes better error handling for identifying gaps and mutations.

LIMITATIONS

It remains challenging to sample sufficiently close to the native structure, even with 100k models. The benchmark set is currently rather small. Once more structures become available, it should be updated. Quality measures could also be improved. RMSD is not a good measure as it becomes large quickly due to the lever arm effect. However, GDT might not be a good measure either since have the structures of the individual domains, which would result in a large GDT to begin with, and all we are interested in is the correct modeling of the linkers. Maybe a dihedral angle distribution might be the way to go. Ideas are appreciated, please email Julia.

REVISION

revision:61603

test_id: 690372

status: passed

Scientific test: mp_lipid_acc

FAILURES

None

RESULTS

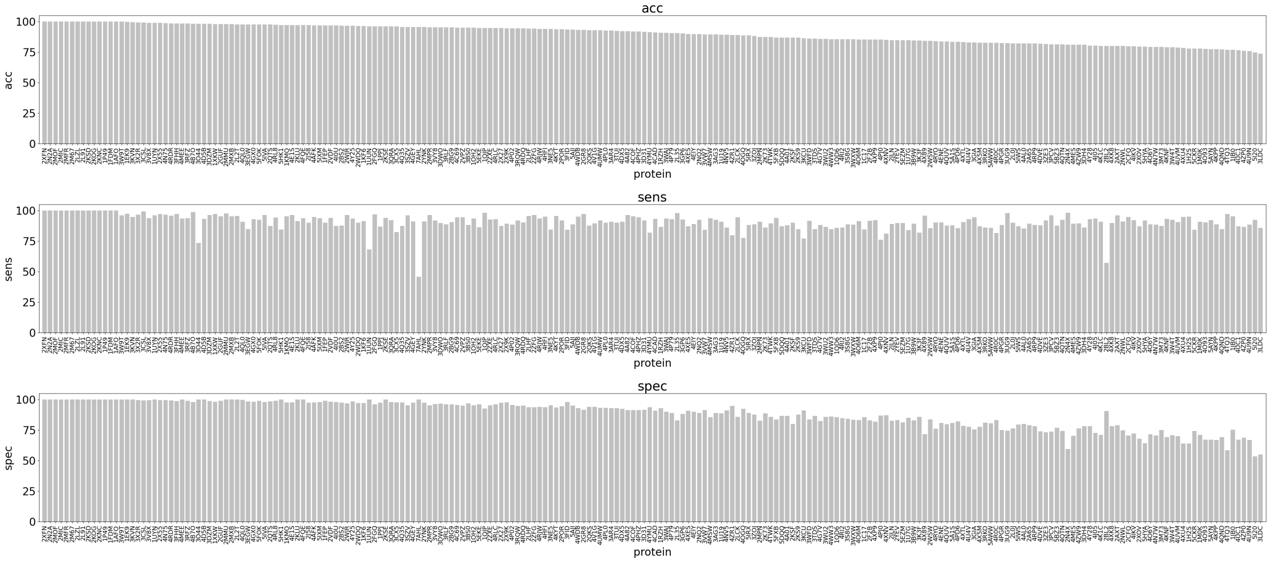

AUTHOR AND DATE

The benchmark was set up by Julia Koehler Leman (julia.koehler.leman@gmail.com) in March 2019.

The PI is Richard Bonneau.

PURPOSE OF THE TEST

The benchmark tests the quality of the concave hull algorithm that underlies the algorithm to classify lipid accessibility from structure. Identifying lipid exposed residues from a membrane protein structure differs from SASA (solvent-accessible surface area) algorithms in that pore-forming proteins would have pore-facing residues to be predicted as solvent-accessible, but wouldn't distinguish water vs. lipid as solvent. SASA algorithms would predict exposed residues as solvent-exposed, yet our mp_lipid_acc algorithm predicts residues that are purely exposed to lipid.

BENCHMARK DATASET

We test the protocol on a diverse set of 223 membrane proteins. We wanted to include structures that are helical, beta-barrels, single TM span proteins, proteins with large pores, small pores, multiple pores (aquaporin-like), oval (like chloride channels) etc. How the dataset was created is described in detail in (Koehler Leman et. al, BMC BioInfo, 2017).

The algorithm was developed by testing a small set of proteins (also in the publication). Then, the algorithm was run on the benchmark set and the output was further manually curated via PyMol. For residues that are lipid-exposed, we set the B-factor to 50, for others, the B-factor is 0. The entire benchmark set was therefore hand-curated. This dataset is given in

Rosetta/main/tests/scientific/data/mp_lipid_acc/db_hand_curated

The input dataset is the set of PDBs in

Rosetta/main/tests/scientific/data/mp_lipid_acc/db_input

the output of which is compared against the hand-curated set.

PROTOCOL

Note that computing lipid accessibility from structure is a pure geometric algorithm that does not consider any other score terms. The input is a membrane protein already transformed into membrane coordinates. The details of the algorithm are described in detail in (Koehler Leman et. al, BMC BioInfo, 2017) and the command lines are given in the supplement to the paper. Note that we are testing default parameters here, which are:

slice width 10 A

distance cutoff 10 A

shell radius 6 A

angle cutoff 65 deg

The runtime is very short and we only need to build a single model since the algorithm is deterministic. It takes about 30s per model x 223 proteins = 6690s which is <2h CPU time.

PERFORMANCE METRICS

Accuracies are computed for each protein individually: between the hand-curated set and predicted output from mp_lipid_acc. The protein-accuracy set was sorted by accuracy from largest to smallest and the list of proteins was used as input into the algorithm. Now, if at any run the accuracies are not sorted from largest to smallest any more, we call this a failure. Note that the predictions are deterministic and there is no reliance on a Monte-Carlo algorithm. Therefore, if the accuracies have changed, the underlying concave hull algorithm must have changed. Further, we will flag when accuracies have changed for a small set of proteins, as it wouldn't be flagged as a failure when all accuracies would have gone up or down by an equal amount.

We also plot the sensitivities and specificities on the set of proteins.

KEY RESULTS

DEFINITIONS AND COMMENTS

LIMITATIONS

The benchmark set is chosen well and incredibly diverse, I don't think large improvements would be expected if the benchmark set were picked 'better'. The algorithm works well but I am sure there is room for improvement. By developing the algorithm I have tested all-atoms vs. CA-only and didn't see a marked improvement, but the runtimes increased quite dramatically, so we stick to CA-atoms only. The algorithm could be improved somehow because we use relatively thick slices (10A thick) and extrapolate from that. Also, the membrane thickness should be a multiple of the slice width to avoid sparse data in the last slice. It would be nice if the slices could be smaller and the dependency to the membrane thickness could be removed. Another idea might be to use a 3D concave hull algorithm, which I am not quite sure whether and how well this would work, since we are looking at a 2D membrane plane.

REVISION

revision:61609

test_id: 691300

status: passed

Scientific test: mp_relax

FAILURES

None

RESULTS

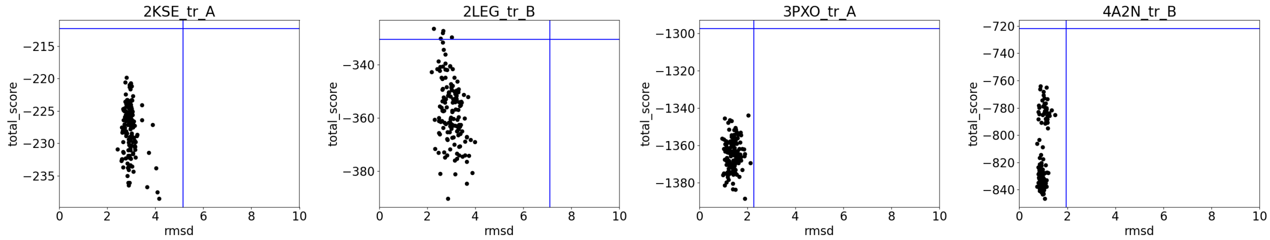

AUTHOR AND DATE

The benchmark was set up by Julia Koehler Leman (julia.koehler.leman@gmail.com) in March 2019.

The research was performed in Jeff Gray's lab, but Julia Koehler Leman is now in the lab of Richard Bonneau.

PURPOSE OF THE TEST

We test whether how much flexibility we can sample with mp_relax. The mp_relax app runs FastRelax under the hood but by using the membrane framework RosettaMP.

BENCHMARK DATASET

The benchmark set contains 4 test proteins that were run for mp_relax from the RosettaMP framework paper (Alford, Koehler Leman et. al, PlosCompBio, 2015). Note that the mp_relax application could be further optimized or compared to the newer protocol mp_range_relax that hasn't been published yet. The paper describes mp_relax and other applications as a proof-of-concept.

Input PDBs were downloaded from the PDBTM database (which have better membrane embedding than PDBs from the OPM database). This means that the proteins are transformed into membrane coordinates and have the correct biomolecular assembly, even though we are only looking at dimers for this application. PDBs were cleaned using clean_pdb.py from the Rosetta/tools/protein_tools/scripts directory. Spanfiles were created using the mp_span_from_pdb application in Rosetta.

The PDB files were used as natives for RMSD calculations. Detail command lines are described in the Supplement to (Alford, Koehler Leman et. al, PlosCompBio, 2015).

PROTOCOL

The mp_relax application runs in RosettaScript and uses the FastRelax protocol as a mover but adjusted with the membrane framework RosettaMP. We create 100 models per protein to sample protein conformations. We use the mp_framework_smooth_2012 scorefunction for scoring until something better becomes available.

[Note that the natives should have a membrane residue (MEM), otherwise Rosetta crashes during RMSD calculation.]

On average for this benchmark set, mp_relax creates a decoy in ~350s; that is dependent on size of the protein. That makes 350s x 4 proteins x 100 decoys = 140,000s which are <40 CPU hours.

PERFORMANCE METRICS

We use the total Rosetta score and RMSD to the native structure as we are interested in the sampling range and the score ranges in sampling. A passed test constitutes 90% of the decoys generated have a smaller than the defined score and RMSD cutoffs. The cutoffs were defined by looking at these ranges in a single run without outlier decoys. Specifically, for RMSD we use the maximum RMSD + stdev. For score, we use the maximum score + stdev.

KEY RESULTS

We use crystal and structures as natives for comparison. The sampling range is up to 4 Angstrom in our examples.

2kse is an NMR structure of a histidine kinase, which normally has 2 chains but is here modeled as a single chain. That's bad. Not sure why this was chosen.

2leg is the NMR structure of DsbB, not a great structure and has a long loop which flops around quite a bit

3pxo is the crystal structure of metarhodopsin, a decent structure

4a2n is a crystal sructure with relatively long loops which don't seem to be super floppy - Rosetta predicts a pretty narrow range

DEFINITIONS AND COMMENTS

LIMITATIONS

The mp_relax app needs to be improved (or moved to mp_range_relax) and to benchmark that properly, a larger dataset is needed. From that, a collection of good, intermediate and outlier proteins should be picked for continuous scientific benchmarking.

The protein embedding in the membrane should be optimized during relax. Since the FoldTree in mp_relax is such that the MEM is relaxed around the protein, doing that and superimposing the structures leads to MEM residues all over the place. The protein set is not very well picked. We need a broader set including beta-barrels and larger ranges of protein size with high-quality structures. There are NMR structures in here, which we might or might not want to avoid. We need to test multi-chain proteins in relax. And we need to test against range_relax and mp_range_relax which runs much master and provides better results.

REVISION

revision:61588

test_id: 686955

status: passed

Scientific test: mp_symdock

FAILURES

None

RESULTS

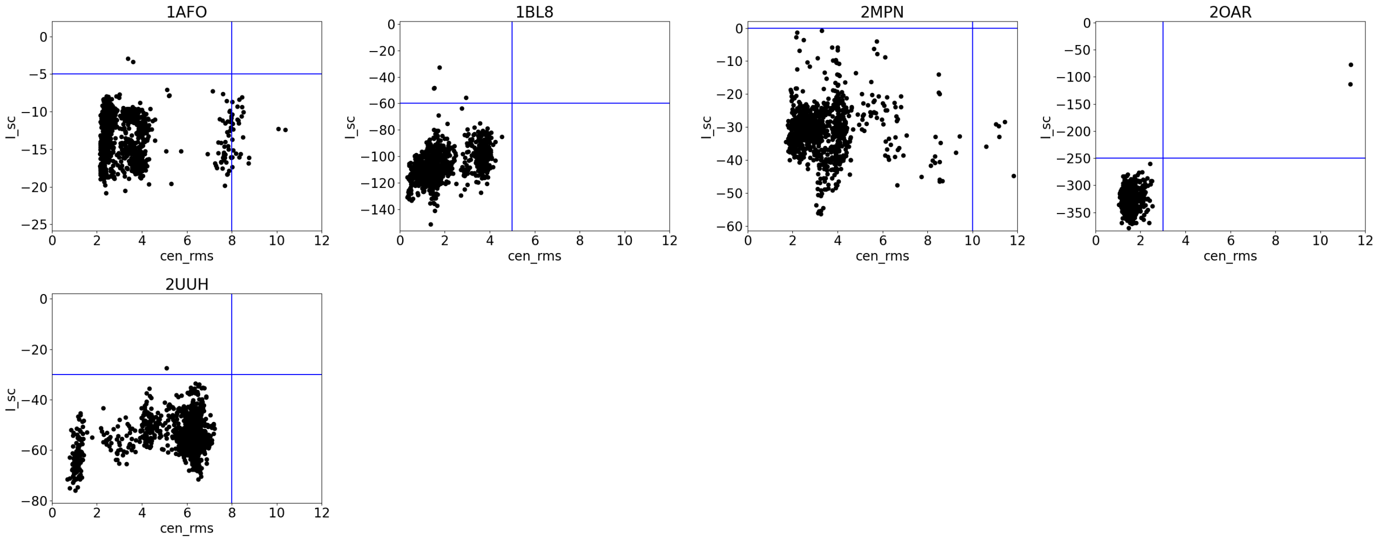

AUTHOR AND DATE

The benchmark was set up by Julia Koehler Leman (julia.koehler.leman@gmail.com) in March 2019.

The research was performed in Jeff Gray's lab, before Julia Koehler Leman moved to the lab of Richard Bonneau.

PURPOSE OF THE TEST

We test Rosetta's ability to dock symmetric membrane proteins, i.e. how well the symmetry framework and membrane framework RosettaMP interface with the docking protocol. We basically check how well Rosetta can recapitulate native subunit interfaces in symmetric membrane proteins, how much the symmetry framework samples in the membrane and how well the membrane scorefunction scores the decoys.

BENCHMARK DATASET

We test on 5 proteins that were part of the original benchmark set for testing RosettaMP described in the paper (Alford, Koehler Leman et. al. PlosCompBio 2015). This benchmark set tests C2, C3, C4 and C5 symmetries.

The input files were downloaded from the OPM database (1BL8 didn't have the correct bioassembly in PDBTM), cleaned, spanfiles were created with mp_span_from_pdb, then I used mp_range_relax to refine the structure. The outer helices in 1BL8 often came off the rest of the structure and started flopping around, the closest RMSD structures had high scores due to clashes, but I took one of those anyway as the starting structure.

PROTOCOL

The protocol is described in (Alford, Koehler Leman et. al. PlosCompBio 2015) with command lines in the paper supplement. However, some flags had to be adjusted to make this run properly.

Note that for -in:file:native I am using the relaxed native but the symmetry framework starts with the asymmetric unit as the input structure. Therefore, the RMSD will never be super close to the native (even though with the adjusted not-optimal flags the RMSDs are actually amazing compared to what I see in the figures in the paper supplement). Not-optimal flags means that to get decoys in a reasonable amount of time, I had so use some flags that should not be used (like one for docking prepack for instance). I can imagine that some code has changed for low-res and high-res filtering steps as well, the docking:ppk flag makes sure the models pass these filters which they otherwise would not.

The protocol is the MPSymDock protocol, which runs the SymDock protocol underneath, which is based on the DockingProtocol. So we have a low-res docking step before it does high-res docking. Because of this, some decoys wouldn't pass the low-res stage, therefore the score file can have a mixture of centroid and high-res decoys with differing headers. That's the reason why the plotting script goes by index and not by column tag.

Runtimes are <100s per decoys: 100s x 5 proteins x 1000 decoys = 500,000 CPU secs which is <140 CPU hours. Note that the runtimes for these are with the docking:ppk flag and will likely increase (and possibly substantially) once better flags are found.

PERFORMANCE METRICS

We use interface score I_sc vs. RMSD to the native. The native here is the relaxed OPM structure, not the symmetrized version created by the make_symdef_file script.

Cutoffs were defined by looking at the I_sc vs. RMSD distribution of the first run. The I_sc vs RMSD plots look substantially better than in the paper, not quite sure why. Differences are inclusion of the '-score:weights mpframework_docking_fa_2015.wts' flag and the '-docking::dock_ppk true'. Cutoffs need to be redone once the protocol has been optimized and better flags are found.

Note that the I_sc in a normal range would be <0, maybe in the range between -20 to 0, not in negative hundreds as we sometimes see here.

KEY RESULTS

The baseline are relaxed structures from the OPM database.

1afo - C2 symmetry of single TM helix

1bl8 - C4 symmetry of potassium channel

2mpn - C2 symmetry of 2TMspan helix - looks like a handshake

2oar - C5 symmetry of a channel

2uuh - C3 symmetry of leukotriene synthase

The RMSDs that are sampled are pretty good but the interface scores are sometimes a little too good and it needs to be figured out where this is coming from. Note that this is a proof-of-concept protocol that could and should be improved code-wise and properly benchmarked.

DEFINITIONS AND COMMENTS

The original benchmarking was done by Rebecca Alford and this benchmark set, even though the same proteins, was set up by JKLeman, so there are slight differences. For instance, while relaxing the structures, I noticed that mp_relax moved 1 protein out of the membrane. Using mp_range_relax the 1BL8 structure of the potassium channel had the outer helices flopping around, meaning they didn't keep their interface with the other helices. This makes me wonder how stable these structures are in the first place and should be played around with more!!!

LIMITATIONS

The protocol should certainly be improved code-wise. This protocol is a proof-of-concept so far, needs to be benchmarked on a larger dataset with more diverse structures and symmetries. I'd like to see some beta-barrels in the benchmark set. Quality measures are standard, so they are fine.

In an earlier version of this protocol the funnel plots looked pretty great, but again, these are docking_ppk structures and had horrible total scores due to clashes. I also noticed that the models (without docking_ppk flag) were too close and tight and therefore have horrible scores compared to the natives. So adjusting the protocol to move the structures slightly further apart (perpendicular to the symmetry axis) should give better scoring models that are closer to the native without needing the docking_ppk flag.

REVISION

revision: 61711

test_id: 713288

Scientific test: peptide_pnear_vs_ic50

FAILURES

None.

RESULTS SUMMARY

Analysis of correlation of predicted folding propensity with experimentally-measured inhibition values (which should be linear):

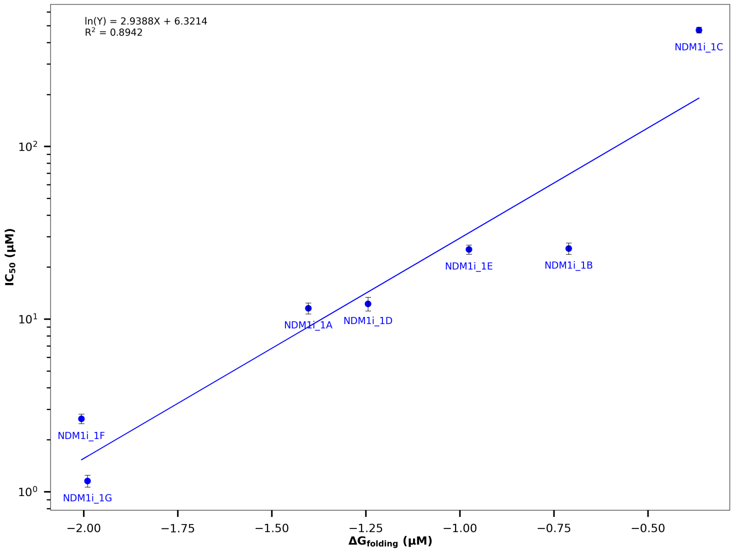

Folding funnels:

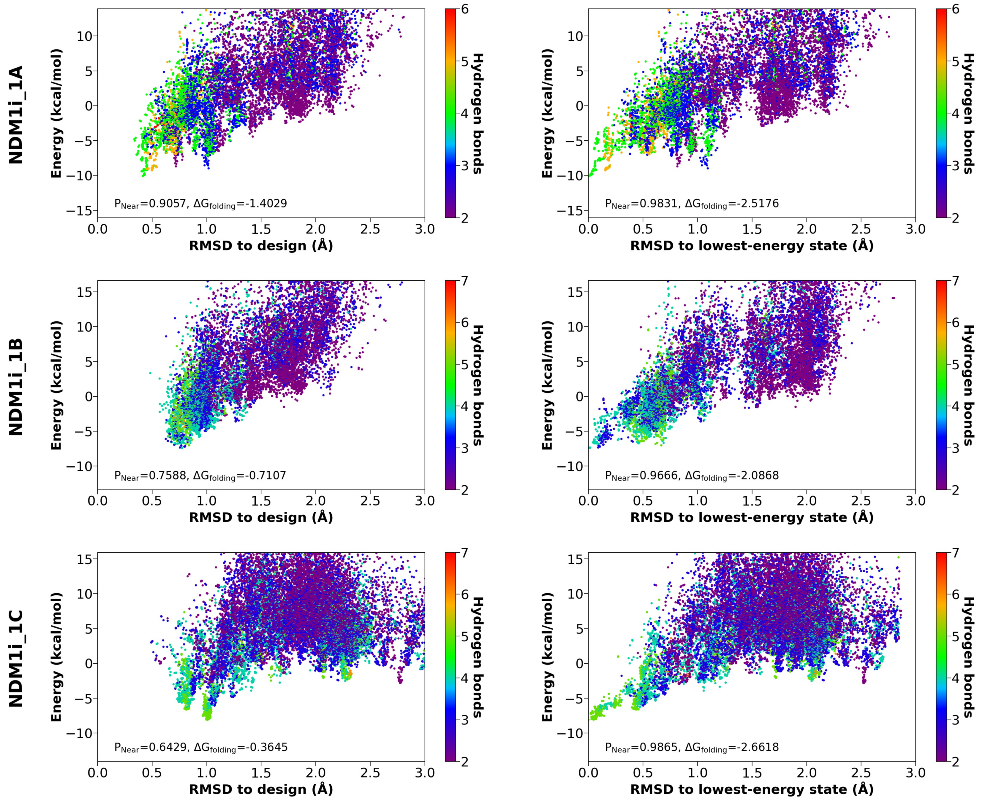

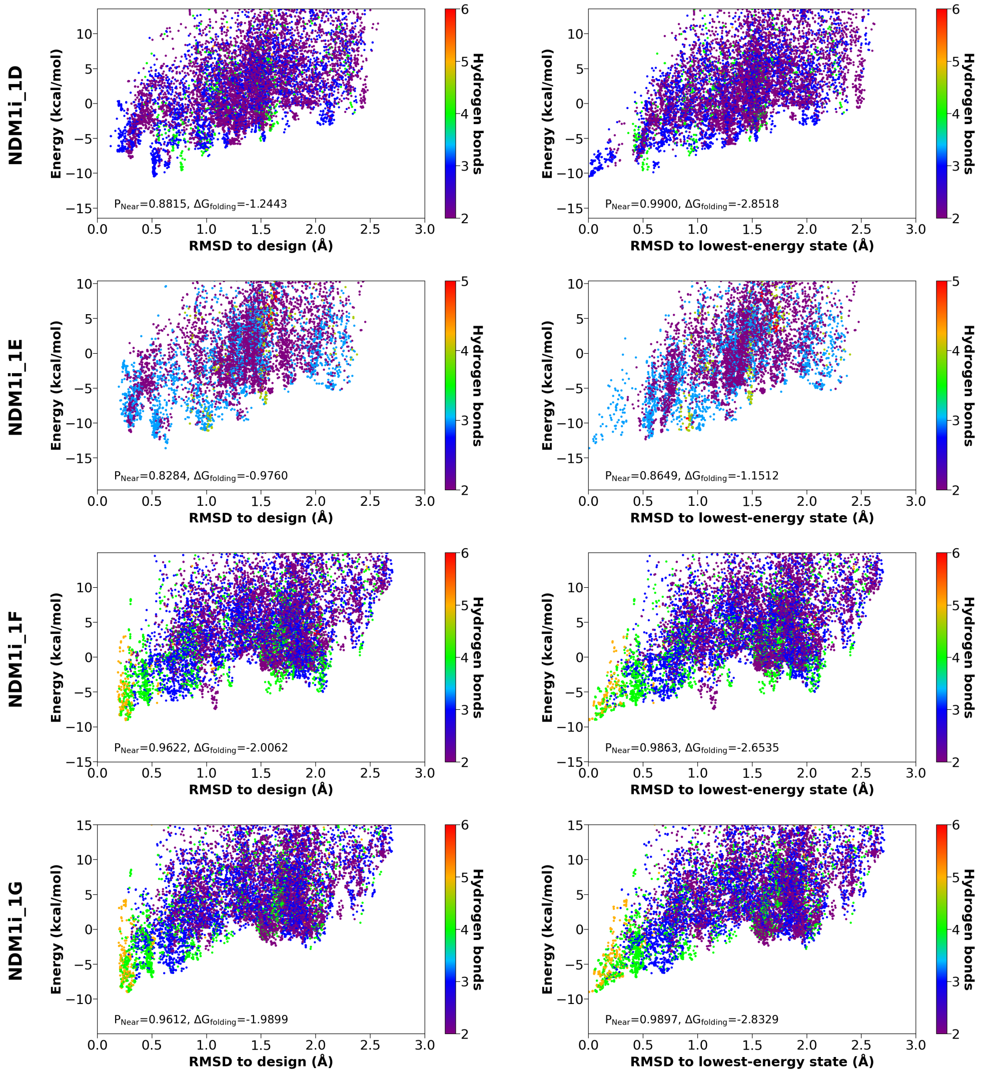

Peptide NDM1i_1A:
Total samples = 22948
Computed PNear = 0.9057
Computed PNear to lowest E = 0.9831
Computed DG_folding = -1.4029
Computed DG_folding to lowest = -2.5176
Lowest energy = -10.0815 kcal/mol
RMSD of lowest energy = 0.415 Angstroms
Lowest RMSD = 0.337 Angstroms
Highest RMSD = 2.830 Angstroms
Energy gap (minE>1.5A - minE) = 7.5346 kcal/mol

More than 18,000 samples? YES
PNear value over 0.83? YES
PNear value to lowest E over 0.83? YES
Lowest energy under 0.45 A RMSD? YES
Sampling below expected lower threshold RMSD(0.45 A)? YES
Sampling beyond 1.5 A RMSD? YES
Sampling beyond 2.5 A RMSD? YES
3+ kcal/mol energy gap? YES
OVERALL PASS? YES

Peptide NDM1i_1B:
Total samples = 26899
Computed PNear = 0.7588
Computed PNear to lowest E = 0.9666
Computed DG_folding = -0.7107
Computed DG_folding to lowest = -2.0868
Lowest energy = -7.3848 kcal/mol
RMSD of lowest energy = 0.773 Angstroms
Lowest RMSD = 0.483 Angstroms

Highest RMSD = 2.949 Angstroms
Energy gap (minE>1.5A - minE) = 5.8750 kcal/mol

More than 18,000 samples? YES
PNear value over 0.68? YES
PNear value to lowest E over 0.68? YES
Lowest energy under 0.85 A RMSD? YES
Sampling below expected lower threshold RMSD(0.7 A)? YES
Sampling beyond 1.5 A RMSD? YES
Sampling beyond 2.4 A RMSD? YES
3+ kcal/mol energy gap? YES
OVERALL PASS? YES

Peptide NDM1i_1C:
Total samples = 39787
Computed PNear = 0.6429
Computed PNear to lowest E = 0.9865
Computed DG_folding = -0.3645
Computed DG_folding to lowest = -2.6618
Lowest energy = -8.0777 kcal/mol
RMSD of lowest energy = 1.010 Angstroms
Lowest RMSD = 0.491 Angstroms
Highest RMSD = 3.135 Angstroms
Energy gap (minE>1.5A - minE) = 5.2507 kcal/mol

More than 18,000 samples? YES
PNear value over 0.57? YES
PNear value to lowest E over 0.57? YES
Lowest energy under 1.05 A RMSD? YES
Sampling below expected lower threshold RMSD(0.55 A)? YES
Sampling beyond 1.5 A RMSD? YES
Sampling beyond 2.9 A RMSD? YES
3+ kcal/mol energy gap? YES
OVERALL PASS? YES

Peptide NDM1i_1D:
Total samples = 22426
Computed PNear = 0.8815
Computed PNear to lowest E = 0.9900
Computed DG_folding = -1.2443
Computed DG_folding to lowest = -2.8518
Lowest energy = -10.4428 kcal/mol
RMSD of lowest energy = 0.520 Angstroms
Lowest RMSD = 0.122 Angstroms
Highest RMSD = 2.644 Angstroms
Energy gap (minE>1.5A - minE) = 5.0846 kcal/mol

More than 18,000 samples? YES
PNear value over 0.8? YES
PNear value to lowest E over 0.8? YES
Lowest energy under 0.55 A RMSD? YES
Sampling below expected lower threshold RMSD(0.25 A)? YES
Sampling beyond 1.5 A RMSD? YES
Sampling beyond 2.4 A RMSD? YES
3+ kcal/mol energy gap? YES
OVERALL PASS? YES

Peptide NDM1i_1E:
Total samples = 25841
Computed PNear = 0.8284
Computed PNear to lowest E = 0.8649
Computed DG_folding = -0.9760
Computed DG_folding to lowest = -1.1512
Lowest energy = -13.6068 kcal/mol
RMSD of lowest energy = 0.626 Angstroms
Lowest RMSD = 0.196 Angstroms
Highest RMSD = 2.562 Angstroms
Energy gap (minE>1.5A - minE) = 6.3215 kcal/mol

More than 18,000 samples? YES
PNear value over 0.75? YES
PNear value to lowest E over 0.75? YES

Lowest energy under 0.65 A RMSD? YES
Sampling below expected lower threshold RMSD(0.3 A)? YES
Sampling beyond 1.5 A RMSD? YES
Sampling beyond 2.2 A RMSD? YES
3+ kcal/mol energy gap? YES
OVERALL PASS? YES

Peptide NDM1i_1F:
Total samples = 23540
Computed PNear = 0.9622
Computed PNear to lowest E = 0.9863
Computed DG_folding = -2.0062
Computed DG_folding to lowest = -2.6535
Lowest energy = -9.0340 kcal/mol
RMSD of lowest energy = 0.259 Angstroms
Lowest RMSD = 0.185 Angstroms
Highest RMSD = 3.011 Angstroms
Energy gap (minE>1.5A - minE) = 3.8440 kcal/mol

More than 18,000 samples? YES
PNear value over 0.88? YES
PNear value to lowest E over 0.88? YES
Lowest energy under 0.32 A RMSD? YES
Sampling below expected lower threshold RMSD(0.22 A)? YES
Sampling beyond 1.5 A RMSD? YES
Sampling beyond 2.5 A RMSD? YES
3+ kcal/mol energy gap? YES
OVERALL PASS? YES

Peptide NDM1i_1G:
Total samples = 23794
Computed PNear = 0.9612
Computed PNear to lowest E = 0.9897
Computed DG_folding = -1.9899
Computed DG_folding to lowest = -2.8329
Lowest energy = -8.9831 kcal/mol
RMSD of lowest energy = 0.275 Angstroms
Lowest RMSD = 0.196 Angstroms
Highest RMSD = 2.703 Angstroms
Energy gap (minE>1.5A - minE) = 6.1882 kcal/mol

More than 18,000 samples? YES
PNear value over 0.9? YES
PNear value to lowest E over 0.9? YES
Lowest energy under 0.32 A RMSD? YES
Sampling below expected lower threshold RMSD(0.25 A)? YES
Sampling beyond 1.5 A RMSD? YES
Sampling beyond 2.6 A RMSD? YES
5+ kcal/mol energy gap? YES
OVERALL PASS? YES

Correlation analysis:
R-squared value from fitting greater than 0.85? YES

AUTHOR

Vikram K. Mulligan (vmulligan@flatironinstitute.org), Center for Computational Biology, Flatiron Institute, 5 June 2020.

PURPOSE OF THE TEST

When designing peptides to bind to targets, the flexibility of the peptide is a major impediment to binding. This is because there is an entropic cost associated with ordering a disordered molecule on binding. A rigidly-structured peptide that is pre-organized in the binding-competent conformation has a lower entropic cost and can bind more tightly. When interactions between peptide and target have been optimized with Rosetta, peptide rigidity becomes the major determininant of binding affinity. Rosetta predictions of peptide folding propensity, carried out with the simple_cycpep_predict application, correlate strongly with experimentally-measured binding affinity, at least with the ref2015 energy function. This correlation has improved from talaris2013 through talaris2014 to ref2015 due to improved training of the energy function against physical properties of fluids and against fluid MD simulation (see image below). This test exists to ensure that this correlation between prediction and experiment is maintained with future versions of the energy function and with future releases of Rosetta.

Improvements from talaris2013 through ref2015.

What does the benchmark test and why?

This test benchmarks seven peptides that were previously designed to bind to and inhibit the New Delhi metallo-beta-lactamase 1 (NDM-1). (For our purposes, IC50 values for these competitive inhibitors, measured at a constant concentration of the substrate of the reaction being inhibited, are proportional to inhibitor binding affinity.) The test carries out the following steps:

1. Large-scale conformational sampling for each of the seven peptides.

2. From the energy landscape sampled, calculation of the metrics PNear and estimated Delta-G of folding (which are closely-relarted measures of propensity to be rigid in the binding-competent conformation).

3. Plotting of the logarithm of experimentally-measured IC50 values against the computed Delta-G of folding values computed in the previous step.

4. Fitting of these data to a simple linear model, and extraction of the R-squared value for the fit.

The R-squared value should not drop below 0.85.

BENCHMARK DATASET

How many proteins are in the set?

- Seven peptides, named NDM1i-1A through NDM1i-1G. (These were previously called 8res1B, 8res2, 8res3, Holmes, Holmes_l8p, Adler, and Moriarty, respectively). All have been synthesized, the IC50 value for each binding to and inhibiting the NDM-1 enzyme's hydrolysis of nitrocefin have been measured.

What dataset are you using? Is it published? If yes, please add a citation.

- These peptides are described in Mulligan _et al._ (2020). Computationally-designed peptide macrocycle inhibitors of New Delhi metallo-beta-lactamase 1. Manuscript in preparation.

What are the input files? How were the they created?

- The input for each structure prediction run is the peptide, designed with Rosetta, in PDB format, along with its sequence in ASCII text format.

- The input for the correlation analysis is the set of output Delta-G of folding values from the structure prediction runs, plus an experimentally-measured set of IC50 values.

PROTOCOL

State and briefly describe the protocol.

The simple_cycpep_predict application uses the generalized kinematic closure algorithm (GenKIC) to rapidly sample closed conformations of a heteropolymer macrocycle built from any combination of alpha-amino acids, peptoids, or other related building-blocks. Each closure attempt is relaxed using the FastRelax protocol. For small (~8 to ~10 residue) peptide macrocycles, the application can usually sample close to the native state with less expense than protein _ab initio_.

The simple_cycpep_predict application supports hierarchical MPI-based job distribution and data reduction, as well as multi-threaded parallel job execution within a node. In MPI mode, statistics about the full sampled ensemble, including the funnel quality metric PNear, are computed automatically during the data collection and reduction phase.

Is there a publication that describes the protocol?

The simple_cycpep_predict application is described in the following publications:

1. Bhardwaj G, Mulligan VK, Bahl CD, _et al._ (2016). Accurate de novo design of hyperstable constrained peptides. _Nature_ 538(7625):329-35.

2. Hosseinzadeh P, Bhardwaj G, Mulligan VK, _et al._ (2018). Comprehensive computational design of ordered peptide macrocycles. _Science_ 358(6369):1461-6.

3. Mulligan _et al._ (2020). Computationally-designed peptide macrocycle inhibitors of New Delhi metallo-beta-lactamase 1. Manuscript in preparation.

How many CPU hours does this benchmark take approximately?

Approximately 400 CPU-hours. The test runs on 4 nodes, 20 cores per node, for roughly 5 wall hours.

In debug mode, this test takes approximately 10 CPU-hours (1 node, 20 cores, for 0.5 wall hours).

PERFORMANCE METRICS

What are the performance metrics used and why were they chosen?

All of the following must be true for the test to pass:

- More than 18,000 successful samples (200 in debug mode).

- Lowest-RMSD sample within a threshold RMSD of the design model. (Varies by peptide.)

- Highest-RMSD sample outside of a thrshold RMSD of the design model. (Varies by peptide.)

- Lowest-energy sample within a threshold RMSD of the design model. (Varies by peptide.)

- Energy gap (gap between lowest-energy sample > 1.5 A and overall lowest) bigger than a threshold value. (Varies by peptide.)

- PNear greater than a threshold value. (Varies by peptide.)

- Most importantly, R-squared value for the linear relationship between ln(IC50) and Delta-G of folding is greater than 0.85.

How do you define a pass/fail for this test?

Failure of any of the above results in an overall failure.

How were any cutoffs defined?

These are based on Rosetta performance as of 5 June 2020. The observed R-squared value reported in Mulligan _et al._ (2020) is 0.9.

KEY RESULTS

What is the baseline to compare things to - experimental data or a previous Rosetta protocol?

Past iterations of this test and performance reported in Mulligan _et al._ (2020). (See Figure 3 in that paper.)

Describe outliers in the dataset.

NDM1i-1C should have the lowest PNear value and highest Delta-G of folding; NDM1i-1F and NDM1i-1G should have the highest PNear value and lowest Delta-G of folding. These are the worst and best binders, respectively.

DEFINITIONS AND COMMENTS

State anything you think is important for someone else to replicate your results.

For full reproducibility, see the 92-page supplement to Mulligan _et al._ (2020).

LIMITATIONS

What are the limitations of the benchmark? Consider dataset, quality measures, protocol etc.

The parameters of the correlation are expected to vary from target to target, and for any given target, there are very few data. (Chemically synthesizing a candidate peptide binder and testing its affinity are inherently low-throughput experimental techniques.)

How could the benchmark be improved?

More peptides, and more targets.

What goals should be hit to make this a "good" benchmark?

Well, I think it's pretty good as it is.

REVISION

revision:61592

test_id: 687669

status: passed

Scientific test: Protein Data Bank diagnostic

AUTHOR AND DATE

Steven M Lewis, smlewi@gmail.com, Cyrus Biotechnology, Oct 2018

Sergey Lyskov, sergey.lyskov@gmail.com, Gray lab, Johns Hopkins University, Oct 2018

PURPOSE OF THE TEST

The PDB diagnostic was run on **175,420** PDB files. **169,174** files passed the test and **6,246** failed! This test tries to load every PDB file in the PDB database and classifies the failures that occur. The command line below shows what was done; broadly all versions of this test examine load-time problems and more expensive versions (`-PDB_diagnostic::skip_pack_and_min false`) also check for errors during scoring, packing, and minimization.

"Hunting down these bugs is the most fun thing you can do on a Thursday morning" - Andy Watkins, probably.

An individual PDB passes or fails this test based on whether it errors out or completes the diagnostic. The test as a whole passes or fails based on a "reference results" system, like an expected result in a unit test. About 700 PDBs and 1200 CIFs fail at the time of this writing; the purpose of the test is to document the failures and watch for new ones, so PDBs failing in an expected manner does not constitute an overall test failure. The test will fail if PDBs pass or fail **UNEXPECTEDLY**, where the expectation is defined by the reference results (see below).

If you find that this page is telling you the test failed because there are **FEWER** errors: **GREAT**! You fixed some bugs! You can update the reference results following the instructions at the bottom of the page.

If you find that there are **MORE** failures than expected, especially non-timeout failures, consider it a warning that recent code changes may have introduced bugs into the PDB reading machinery. If you don't know what's going on: post to Slack or devel. **DO NOT** just update the reference results in this case.

If you want to know more about what this test does - pester Steven Lewis to write proper documentation.

Command line used: `/home/benchmark/benchmark/W.jazz.hpc-b/rosetta.Jazz.hpc-b/_commits_/main/source/test/timelimit.py 8 /home/benchmark/benchmark/W.jazz.hpc-b/rosetta.Jazz.hpc-b/_commits_/main/source/bin/PDB_diagnostic.default.linuxclangrelease -no_color -out:file:score_only /dev/null -jd2::delete_old_poses true -ignore_unrecognized_res false -load_PDB_components true -packing::pack_missing_sidechains false -packing::repack_only true -s {input_file} -ignore_zero_occupancy false -in:file:obey_ENDMDL true -PDB_diagnostic::skip_pack_and_min true -PDB_diagnostic::reading_only true`

6,246 total PDBs failed with the following error codes:

```
4,984 「unrecognized_residue」
  450 「fill_missing_atoms」
  259 「unknown」
  196 「multiple_disulfides」
   94 「missing_disulfide_partner」
   61 「bad_patch」
   48 「unknown_atom_name」
   34 「duplicate_atom_name」
   31 「reroot_disconnected」
   20 「exceed_timeout」
   17 「ld_chirality」
   16 「zero_atom_restype」
   12 「nu_conformer」
    5 「no_usable_coords」
    4 「merge_with_next」
    4 「insufficient_mainchain」
    3 「partly_recognized」
    2 「cutpoint_neighbour」
    2 「base_of_chi」
    2 「incompatible_w_polymer」
    1 「zero_length_xyzVector」
    1 「alias_missing_atom」
```

Test marked as **FAILED** due to following errors:

```
PDB 1A08 was passing test before but now failed with 「duplicate_atom_name」 error! Its run-log could be found in 「1A08」
PDB 1A09 was passing test before but now failed with 「duplicate_atom_name」 error! Its run-log could be found in 「1A09」
PDB 1A1A was passing test before but now failed with 「duplicate_atom_name」 error! Its run-log could be found in 「1A1A」
PDB 1A1V was passing test before but now failed with 「unrecognized_residue」 error! Its run-log could be found in 「1A1V」
PDB 1AC3 was passing test before but now failed with 「bad_patch」 error! Its run-log could be found in 「1AC3」
PDB 1AFC was passing test before but now failed with 「unrecognized_residue」 error! Its run-log could be found in 「1AFC」
```

PDB 1AMJ was passing test before but now failed with ⌜fill_missing_atoms⌟ error! Its run-log could be found in ⌜1AMJ⌟
PDB 1APO was passing test before but now failed with ⌜fill_missing_atoms⌟ error! Its run-log could be found in ⌜1APO⌟
PDB 1ASO was passing test before but now failed with ⌜fill_missing_atoms⌟ error! Its run-log could be found in ⌜1ASO⌟
PDB 1ASP was passing test before but now failed with ⌜fill_missing_atoms⌟ error! Its run-log could be found in ⌜1ASP⌟
PDB 1ASQ was passing test before but now failed with ⌜fill_missing_atoms⌟ error! Its run-log could be found in ⌜1ASQ⌟
PDB 1AX0 was passing test before but now failed with ⌜fill_missing_atoms⌟ error! Its run-log could be found in ⌜1AX0⌟
PDB 1AX1 was passing test before but now failed with ⌜fill_missing_atoms⌟ error! Its run-log could be found in ⌜1AX1⌟
PDB 1AX2 was passing test before but now failed with ⌜fill_missing_atoms⌟ error! Its run-log could be found in ⌜1AX2⌟
PDB 1AXY was passing test before but now failed with ⌜fill_missing_atoms⌟ error! Its run-log could be found in ⌜1AXY⌟
PDB 1AXZ was passing test before but now failed with ⌜fill_missing_atoms⌟ error! Its run-log could be found in ⌜1AXZ⌟
PDB 1AZX was passing test before but now failed with ⌜unrecognized_residue⌟ error! Its run-log could be found in ⌜1AZX⌟
PDB 1B30 was passing test before but now failed with ⌜unknown⌟ error! Its run-log could be found in ⌜1B30⌟
PDB 1B3V was passing test before but now failed with ⌜unknown⌟ error! Its run-log could be found in ⌜1B3V⌟
PDB 1B3W was passing test before but now failed with ⌜fill_missing_atoms⌟ error! Its run-log could be found in ⌜1B3W⌟
PDB 1B3X was passing test before but now failed with ⌜unknown⌟ error! Its run-log could be found in ⌜1B3X⌟
PDB 1B3Y was passing test before but now failed with ⌜unknown⌟ error! Its run-log could be found in ⌜1B3Y⌟
PDB 1B3Z was passing test before but now failed with ⌜unknown⌟ error! Its run-log could be found in ⌜1B3Z⌟
PDB 1BCX was passing test before but now failed with ⌜unknown⌟ error! Its run-log could be found in ⌜1BCX⌟
PDB 1BIQ was passing test before but now failed with ⌜fill_missing_atoms⌟ error! Its run-log could be found in ⌜1BIQ⌟
PDB 1BL9 was passing test before but now failed with ⌜fill_missing_atoms⌟ error! Its run-log could be found in ⌜1BL9⌟
PDB 1BVV was passing test before but now failed with ⌜unknown⌟ error! Its run-log could be found in ⌜1BVV⌟
PDB 1BYD was passing test before but now failed with ⌜unrecognized_residue⌟ error! Its run-log could be found in ⌜1BYD⌟
PDB 1C23 was passing test before but now failed with ⌜bad_patch⌟ error! Its run-log could be found in ⌜1C23⌟
PDB 1C5I was passing test before but now failed with ⌜unknown⌟ error! Its run-log could be found in ⌜1C5I⌟
PDB 1CB8 was passing test before but now failed with ⌜fill_missing_atoms⌟ error! Its run-log could be found in ⌜1CB8⌟
PDB 1CF5 was passing test before but now failed with ⌜fill_missing_atoms⌟ error! Its run-log could be found in ⌜1CF5⌟
PDB 1CPX was passing test before but now failed with ⌜fill_missing_atoms⌟ error! Its run-log could be found in ⌜1CPX⌟
PDB 1D40 was passing test before but now failed with ⌜unrecognized_residue⌟ error! Its run-log could be found in ⌜1D40⌟
PDB 1D41 was passing test before but now failed with ⌜unrecognized_residue⌟ error! Its run-log could be found in ⌜1D41⌟
PDB 1D76 was passing test before but now failed with ⌜unrecognized_residue⌟ error! Its run-log could be found in ⌜1D76⌟
PDB 1DBG was passing test before but now failed with ⌜fill_missing_atoms⌟ error! Its run-log could be found in ⌜1DBG⌟
PDB 1DBO was passing test before but now failed with ⌜fill_missing_atoms⌟ error! Its run-log could be found in ⌜1DBO⌟
PDB 1DGO was passing test before but now failed with ⌜unrecognized_residue⌟ error! Its run-log could be found in ⌜1DGO⌟
PDB 1DOA was passing test before but now failed with ⌜reroot_disconnected⌟ error! Its run-log could be found in ⌜1DOA⌟
PDB 1DWA was passing test before but now failed with ⌜fill_missing_atoms⌟ error! Its run-log could be found in ⌜1DWA⌟
PDB 1DWF was passing test before but now failed with ⌜fill_missing_atoms⌟ error! Its run-log could be found in ⌜1DWF⌟
PDB 1DWG was passing test before but now failed with ⌜fill_missing_atoms⌟ error! Its run-log could be found in ⌜1DWG⌟
PDB 1DWH was passing test before but now failed with ⌜fill_missing_atoms⌟ error! Its run-log could be found in ⌜1DWH⌟
PDB 1DWI was passing test before but now failed with ⌜fill_missing_atoms⌟ error! Its run-log could be found in ⌜1DWI⌟
PDB 1DWJ was passing test before but now failed with ⌜fill_missing_atoms⌟ error! Its run-log could be found in ⌜1DWJ⌟
PDB 1E03 was passing test before but now failed with ⌜unrecognized_residue⌟ error! Its run-log could be found in ⌜1E03⌟
PDB 1E0X was passing test before but now failed with ⌜unknown⌟ error! Its run-log could be found in ⌜1E0X⌟
PDB 1E4M was passing test before but now failed with ⌜fill_missing_atoms⌟ error! Its run-log could be found in ⌜1E4M⌟
PDB 1E5N was passing test before but now failed with ⌜unknown⌟ error! Its run-log could be found in ⌜1E5N⌟
PDB 1E6Q was passing test before but now failed with ⌜fill_missing_atoms⌟ error! Its run-log could be found in ⌜1E6Q⌟
PDB 1E6S was passing test before but now failed with ⌜fill_missing_atoms⌟ error! Its run-log could be found in ⌜1E6S⌟
PDB 1E6X was passing test before but now failed with ⌜fill_missing_atoms⌟ error! Its run-log could be found in ⌜1E6X⌟
PDB 1E70 was passing test before but now failed with ⌜fill_missing_atoms⌟ error! Its run-log could be found in ⌜1E70⌟
PDB 1E71 was passing test before but now failed with ⌜fill_missing_atoms⌟ error! Its run-log could be found in ⌜1E71⌟
PDB 1E72 was passing test before but now failed with ⌜fill_missing_atoms⌟ error! Its run-log could be found in ⌜1E72⌟
PDB 1E73 was passing test before but now failed with ⌜fill_missing_atoms⌟ error! Its run-log could be found in ⌜1E73⌟
PDB 1E8X was passing test before but now failed with ⌜nu_conformer⌟ error! Its run-log could be found in ⌜1E8X⌟
PDB 1E98 was passing test before but now failed with ⌜duplicate_atom_name⌟ error! Its run-log could be found in ⌜1E98⌟
PDB 1E99 was passing test before but now failed with ⌜duplicate_atom_name⌟ error! Its run-log could be found in ⌜1E99⌟
PDB 1E9B was passing test before but now failed with ⌜duplicate_atom_name⌟ error! Its run-log could be found in ⌜1E9B⌟
PDB 1E9D was passing test before but now failed with ⌜duplicate_atom_name⌟ error! Its run-log could be found in ⌜1E9D⌟
PDB 1EG6 was passing test before but now failed with ⌜unrecognized_residue⌟ error! Its run-log could be found in ⌜1EG6⌟
PDB 1EL2 was passing test before but now failed with ⌜unrecognized_residue⌟ error! Its run-log could be found in ⌜1EL2⌟
PDB 1ELN was passing test before but now failed with ⌜unrecognized_residue⌟ error! Its run-log could be found in ⌜1ELN⌟
PDB 1EQ8 was passing test before but now failed with ⌜fill_missing_atoms⌟ error! Its run-log could be found in ⌜1EQ8⌟
PDB 1F4Y was passing test before but now failed with ⌜unrecognized_residue⌟ error! Its run-log could be found in ⌜1F4Y⌟
PDB 1F91 was passing test before but now failed with ⌜fill_missing_atoms⌟ error! Its run-log could be found in ⌜1F91⌟
PDB 1F9O was passing test before but now failed with ⌜unrecognized_residue⌟ error! Its run-log could be found in ⌜1F9O⌟
PDB 1FA2 was passing test before but now failed with ⌜unrecognized_residue⌟ error! Its run-log could be found in ⌜1FA2⌟
PDB 1FGG was passing test before but now failed with ⌜bad_patch⌟ error! Its run-log could be found in ⌜1FGG⌟
PDB 1FH7 was passing test before but now failed with ⌜fill_missing_atoms⌟ error! Its run-log could be found in ⌜1FH7⌟
PDB 1FH8 was passing test before but now failed with ⌜fill_missing_atoms⌟ error! Its run-log could be found in ⌜1FH8⌟
PDB 1FH9 was passing test before but now failed with ⌜unknown⌟ error! Its run-log could be found in ⌜1FH9⌟
PDB 1FHD was passing test before but now failed with ⌜fill_missing_atoms⌟ error! Its run-log could be found in ⌜1FHD⌟
PDB 1FQ7 was passing test before but now failed with ⌜fill_missing_atoms⌟ error! Its run-log could be found in ⌜1FQ7⌟
PDB 1G22 was passing test before but now failed with ⌜unrecognized_residue⌟ error! Its run-log could be found in ⌜1G22⌟
PDB 1GA8 was passing test before but now failed with ⌜unrecognized_residue⌟ error! Its run-log could be found in ⌜1GA8⌟
PDB 1GAI was passing test before but now failed with ⌜unrecognized_residue⌟ error! Its run-log could be found in ⌜1GAI⌟
PDB 1GJN was passing test before but now failed with ⌜fill_missing_atoms⌟ error! Its run-log could be found in ⌜1GJN⌟
PDB 1GNY was passing test before but now failed with ⌜unknown⌟ error! Its run-log could be found in ⌜1GNY⌟
PDB 1GOQ was passing test before but now failed with ⌜unknown⌟ error! Its run-log could be found in ⌜1GOQ⌟
PDB 1GOR was passing test before but now failed with ⌜unknown⌟ error! Its run-log could be found in ⌜1GOR⌟
PDB 1GQJ was passing test before but now failed with ⌜unknown⌟ error! Its run-log could be found in ⌜1GQJ⌟
PDB 1GQL was passing test before but now failed with ⌜unknown⌟ error! Its run-log could be found in ⌜1GQL⌟
PDB 1GQT was passing test before but now failed with ⌜unknown⌟ error! Its run-log could be found in ⌜1GQT⌟
PDB 1GYE was passing test before but now failed with ⌜unknown⌟ error! Its run-log could be found in ⌜1GYE⌟

PDB 1H12 was passing test before but now failed with 「fill_missing_atoms」 error! Its run-log could be found in 「1H12」
PDB 1H4G was passing test before but now failed with 「unknown」 error! Its run-log could be found in 「1H4G」
PDB 1H4H was passing test before but now failed with 「unknown」 error! Its run-log could be found in 「1H4H」
PDB 1HM2 was passing test before but now failed with 「fill_missing_atoms」 error! Its run-log could be found in 「1HM2」
PDB 1HM3 was passing test before but now failed with 「fill_missing_atoms」 error! Its run-log could be found in 「1HM3」
PDB 1HMU was passing test before but now failed with 「fill_missing_atoms」 error! Its run-log could be found in 「1HMU」
PDB 1HMW was passing test before but now failed with 「fill_missing_atoms」 error! Its run-log could be found in 「1HMW」
PDB 1IDX was passing test before but now failed with 「unrecognized_residue」 error! Its run-log could be found in 「1IDX」
PDB 1II1 was passing test before but now failed with 「unrecognized_residue」 error! Its run-log could be found in 「1II1」
PDB 1IOO was passing test before but now failed with 「fill_missing_atoms」 error! Its run-log could be found in 「1IOO」
PDB 1IQQ was passing test before but now failed with 「fill_missing_atoms」 error! Its run-log could be found in 「1IQQ」
PDB 1ISV was passing test before but now failed with 「unknown」 error! Its run-log could be found in 「1ISV」
PDB 1ISW was passing test before but now failed with 「unknown」 error! Its run-log could be found in 「1ISW」
PDB 1ISX was passing test before but now failed with 「unknown」 error! Its run-log could be found in 「1ISX」
PDB 1IT0 was passing test before but now failed with 「fill_missing_atoms」 error! Its run-log could be found in 「1IT0」
PDB 1IZ2 was passing test before but now failed with 「unrecognized_residue」 error! Its run-log could be found in 「1IZ2」
PDB 1J10 was passing test before but now failed with 「fill_missing_atoms」 error! Its run-log could be found in 「1J10」
PDB 1J90 was passing test before but now failed with 「unrecognized_residue」 error! Its run-log could be found in 「1J90」
PDB 1JGU was passing test before but now failed with 「fill_missing_atoms」 error! Its run-log could be found in 「1JGU」
PDB 1JKU was passing test before but now failed with 「fill_missing_atoms」 error! Its run-log could be found in 「1JKU」
PDB 1JKV was passing test before but now failed with 「fill_missing_atoms」 error! Its run-log could be found in 「1JKV」
PDB 1JS5 was passing test before but now failed with 「unrecognized_residue」 error! Its run-log could be found in 「1JS5」
PDB 1JS7 was passing test before but now failed with 「unrecognized_residue」 error! Its run-log could be found in 「1JS7」
PDB 1K1H was passing test before but now failed with 「unknown_atom_name」 error! Its run-log could be found in 「1K1H」
PDB 1K9F was passing test before but now failed with 「fill_missing_atoms」 error! Its run-log could be found in 「1K9F」
PDB 1KO3 was passing test before but now failed with 「fill_missing_atoms」 error! Its run-log could be found in 「1KO3」
PDB 1KTG was passing test before but now failed with 「fill_missing_atoms」 error! Its run-log could be found in 「1KTG」
PDB 1L8N was passing test before but now failed with 「unknown」 error! Its run-log could be found in 「1L8N」
PDB 1LLP was passing test before but now failed with 「fill_missing_atoms」 error! Its run-log could be found in 「1LLP」
PDB 1MBN was passing test before but now failed with 「fill_missing_atoms」 error! Its run-log could be found in 「1MBN」
PDB 1MC9 was passing test before but now failed with 「unknown」 error! Its run-log could be found in 「1MC9」
PDB 1MMM was passing test before but now failed with 「fill_missing_atoms」 error! Its run-log could be found in 「1MMM」
PDB 1MMS was passing test before but now failed with 「fill_missing_atoms」 error! Its run-log could be found in 「1MMS」
PDB 1MUW was passing test before but now failed with 「fill_missing_atoms」 error! Its run-log could be found in 「1MUW」
PDB 1MWJ was passing test before but now failed with 「unrecognized_residue」 error! Its run-log could be found in 「1MWJ」
PDB 1MXD was passing test before but now failed with 「fill_missing_atoms」 error! Its run-log could be found in 「1MXD」
PDB 1MYR was passing test before but now failed with 「fill_missing_atoms」 error! Its run-log could be found in 「1MYR」
PDB 1N9F was passing test before but now failed with 「fill_missing_atoms」 error! Its run-log could be found in 「1N9F」
PDB 1N9H was passing test before but now failed with 「fill_missing_atoms」 error! Its run-log could be found in 「1N9H」
PDB 1N9I was passing test before but now failed with 「fill_missing_atoms」 error! Its run-log could be found in 「1N9I」
PDB 1N9X was passing test before but now failed with 「fill_missing_atoms」 error! Its run-log could be found in 「1N9X」
PDB 1NAE was passing test before but now failed with 「unknown」 error! Its run-log could be found in 「1NAE」
PDB 1NAZ was passing test before but now failed with 「fill_missing_atoms」 error! Its run-log could be found in 「1NAZ」
PDB 1NH3 was passing test before but now failed with 「unrecognized_residue」 error! Its run-log could be found in 「1NH3」
PDB 1NIR was passing test before but now failed with 「fill_missing_atoms」 error! Its run-log could be found in 「1NIR」
PDB 1NQ9 was passing test before but now failed with 「unrecognized_residue」 error! Its run-log could be found in 「1NQ9」
PDB 1NTQ was passing test before but now failed with 「unrecognized_residue」 error! Its run-log could be found in 「1NTQ」
PDB 1NZ1 was passing test before but now failed with 「unknown」 error! Its run-log could be found in 「1NZ1」
PDB 1OD8 was passing test before but now failed with 「fill_missing_atoms」 error! Its run-log could be found in 「1OD8」
PDB 1OFL was passing test before but now failed with 「fill_missing_atoms」 error! Its run-log could be found in 「1OFL」
PDB 1OFM was passing test before but now failed with 「fill_missing_atoms」 error! Its run-log could be found in 「1OFM」
PDB 1OMW was passing test before but now failed with 「reroot_disconnected」 error! Its run-log could be found in 「1OMW」
PDB 1P60 was passing test before but now failed with 「unrecognized_residue」 error! Its run-log could be found in 「1P60」
PDB 1P61 was passing test before but now failed with 「unrecognized_residue」 error! Its run-log could be found in 「1P61」
PDB 1P7P was passing test before but now failed with 「bad_patch」 error! Its run-log could be found in 「1P7P」
PDB 1PAJ was passing test before but now failed with 「fill_missing_atoms」 error! Its run-log could be found in 「1PAJ」
PDB 1PAK was passing test before but now failed with 「fill_missing_atoms」 error! Its run-log could be found in 「1PAK」
PDB 1PCN was passing test before but now failed with 「fill_missing_atoms」 error! Its run-log could be found in 「1PCN」
PDB 1PCO was passing test before but now failed with 「fill_missing_atoms」 error! Its run-log could be found in 「1PCO」
PDB 1PX8 was passing test before but now failed with 「unknown」 error! Its run-log could be found in 「1PX8」
PDB 1PYY was passing test before but now failed with 「unrecognized_residue」 error! Its run-log could be found in 「1PYY」
PDB 1PZ2 was passing test before but now failed with 「unknown」 error! Its run-log could be found in 「1PZ2」
PDB 1QD3 was passing test before but now failed with 「fill_missing_atoms」 error! Its run-log could be found in 「1QD3」
PDB 1QE7 was passing test before but now failed with 「unrecognized_residue」 error! Its run-log could be found in 「1QE7」
PDB 1QH6 was passing test before but now failed with 「unknown」 error! Its run-log could be found in 「1QH6」
PDB 1QH7 was passing test before but now failed with 「unknown」 error! Its run-log could be found in 「1QH7」
PDB 1QK0 was passing test before but now failed with 「fill_missing_atoms」 error! Its run-log could be found in 「1QK0」
PDB 1QW8 was passing test before but now failed with 「unknown」 error! Its run-log could be found in 「1QW8」
PDB 1QY9 was passing test before but now failed with 「fill_missing_atoms」 error! Its run-log could be found in 「1QY9」
PDB 1R0B was passing test before but now failed with 「multiple_disulfides」 error! Its run-log could be found in 「1R0B」
PDB 1R87 was passing test before but now failed with 「unknown」 error! Its run-log could be found in 「1R87」
PDB 1R9S was passing test before but now failed with 「bad_patch」 error! Its run-log could be found in 「1R9S」
PDB 1RK2 was passing test before but now failed with 「fill_missing_atoms」 error! Its run-log could be found in 「1RK2」
PDB 1RKD was passing test before but now failed with 「fill_missing_atoms」 error! Its run-log could be found in 「1RKD」
PDB 1RKS was passing test before but now failed with 「fill_missing_atoms」 error! Its run-log could be found in 「1RKS」
PDB 1RMY was passing test before but now failed with 「unrecognized_residue」 error! Its run-log could be found in 「1RMY」
PDB 1S4G was passing test before but now failed with 「fill_missing_atoms」 error! Its run-log could be found in 「1S4G」
PDB 1S5N was passing test before but now failed with 「fill_missing_atoms」 error! Its run-log could be found in 「1S5N」
PDB 1SR5 was passing test before but now failed with 「unrecognized_residue」 error! Its run-log could be found in 「1SR5」
PDB 1STO was passing test before but now failed with 「fill_missing_atoms」 error! Its run-log could be found in 「1STO」
PDB 1T0Q was passing test before but now failed with 「fill_missing_atoms」 error! Its run-log could be found in 「1T0Q」

PDB 1T0R was passing test before but now failed with ⌜fill_missing_atoms⌟ error! Its run-log could be found in ⌜1T0R⌟
PDB 1T0S was passing test before but now failed with ⌜fill_missing_atoms⌟ error! Its run-log could be found in ⌜1T0S⌟
PDB 1T3I was passing test before but now failed with ⌜unrecognized_residue⌟ error! Its run-log could be found in ⌜1T3I⌟
PDB 1TEQ was passing test before but now failed with ⌜fill_missing_atoms⌟ error! Its run-log could be found in ⌜1TEQ⌟
PDB 1UQY was passing test before but now failed with ⌜unknown⌟ error! Its run-log could be found in ⌜1UQY⌟
PDB 1UQZ was passing test before but now failed with ⌜unknown⌟ error! Its run-log could be found in ⌜1UQZ⌟
PDB 1UR1 was passing test before but now failed with ⌜unknown⌟ error! Its run-log could be found in ⌜1UR1⌟
PDB 1UR2 was passing test before but now failed with ⌜unknown⌟ error! Its run-log could be found in ⌜1UR2⌟
PDB 1US2 was passing test before but now failed with ⌜unknown⌟ error! Its run-log could be found in ⌜1US2⌟
PDB 1UX7 was passing test before but now failed with ⌜unknown⌟ error! Its run-log could be found in ⌜1UX7⌟
PDB 1UXX was passing test before but now failed with ⌜unknown⌟ error! Its run-log could be found in ⌜1UXX⌟
PDB 1UY2 was passing test before but now failed with ⌜unknown⌟ error! Its run-log could be found in ⌜1UY2⌟
PDB 1UY3 was passing test before but now failed with ⌜unknown⌟ error! Its run-log could be found in ⌜1UY3⌟
PDB 1UY4 was passing test before but now failed with ⌜unknown⌟ error! Its run-log could be found in ⌜1UY4⌟
PDB 1UYZ was passing test before but now failed with ⌜unknown⌟ error! Its run-log could be found in ⌜1UYZ⌟
PDB 1UZY was passing test before but now failed with ⌜fill_missing_atoms⌟ error! Its run-log could be found in ⌜1UZY⌟
PDB 1V0K was passing test before but now failed with ⌜fill_missing_atoms⌟ error! Its run-log could be found in ⌜1V0K⌟
PDB 1V0L was passing test before but now failed with ⌜unknown⌟ error! Its run-log could be found in ⌜1V0L⌟
PDB 1V0M was passing test before but now failed with ⌜fill_missing_atoms⌟ error! Its run-log could be found in ⌜1V0M⌟
PDB 1V0N was passing test before but now failed with ⌜fill_missing_atoms⌟ error! Its run-log could be found in ⌜1V0N⌟
PDB 1V6U was passing test before but now failed with ⌜unknown⌟ error! Its run-log could be found in ⌜1V6U⌟
PDB 1V6V was passing test before but now failed with ⌜unknown⌟ error! Its run-log could be found in ⌜1V6V⌟
PDB 1V6W was passing test before but now failed with ⌜unknown⌟ error! Its run-log could be found in ⌜1V6W⌟
PDB 1V6X was passing test before but now failed with ⌜unknown⌟ error! Its run-log could be found in ⌜1V6X⌟
PDB 1VBR was passing test before but now failed with ⌜fill_missing_atoms⌟ error! Its run-log could be found in ⌜1VBR⌟
PDB 1VEW was passing test before but now failed with ⌜fill_missing_atoms⌟ error! Its run-log could be found in ⌜1VEW⌟
PDB 1VQ5 was passing test before but now failed with ⌜unrecognized_residue⌟ error! Its run-log could be found in ⌜1VQ5⌟
PDB 1VQP was passing test before but now failed with ⌜unrecognized_residue⌟ error! Its run-log could be found in ⌜1VQP⌟
PDB 1W2H was passing test before but now failed with ⌜duplicate_atom_name⌟ error! Its run-log could be found in ⌜1W2H⌟
PDB 1W9B was passing test before but now failed with ⌜fill_missing_atoms⌟ error! Its run-log could be found in ⌜1W9B⌟
PDB 1W9D was passing test before but now failed with ⌜fill_missing_atoms⌟ error! Its run-log could be found in ⌜1W9D⌟
PDB 1W9T was passing test before but now failed with ⌜unknown⌟ error! Its run-log could be found in ⌜1W9T⌟
PDB 1WD4 was passing test before but now failed with ⌜fill_missing_atoms⌟ error! Its run-log could be found in ⌜1WD4⌟
PDB 1WU5 was passing test before but now failed with ⌜fill_missing_atoms⌟ error! Its run-log could be found in ⌜1WU5⌟
PDB 1WU6 was passing test before but now failed with ⌜unknown⌟ error! Its run-log could be found in ⌜1WU6⌟
PDB 1X38 was passing test before but now failed with ⌜fill_missing_atoms⌟ error! Its run-log could be found in ⌜1X38⌟
PDB 1X39 was passing test before but now failed with ⌜fill_missing_atoms⌟ error! Its run-log could be found in ⌜1X39⌟
PDB 1X9D was passing test before but now failed with ⌜unrecognized_residue⌟ error! Its run-log could be found in ⌜1X9D⌟
PDB 1XNK was passing test before but now failed with ⌜unrecognized_residue⌟ error! Its run-log could be found in ⌜1XNK⌟
PDB 1XU5 was passing test before but now failed with ⌜fill_missing_atoms⌟ error! Its run-log could be found in ⌜1XU5⌟
PDB 1XWQ was passing test before but now failed with ⌜unknown⌟ error! Its run-log could be found in ⌜1XWQ⌟
PDB 1XYA was passing test before but now failed with ⌜fill_missing_atoms⌟ error! Its run-log could be found in ⌜1XYA⌟
PDB 1XYL was passing test before but now failed with ⌜fill_missing_atoms⌟ error! Its run-log could be found in ⌜1XYL⌟
PDB 1XYM was passing test before but now failed with ⌜fill_missing_atoms⌟ error! Its run-log could be found in ⌜1XYM⌟
PDB 1YOE was passing test before but now failed with ⌜fill_missing_atoms⌟ error! Its run-log could be found in ⌜1YOE⌟
PDB 271D was passing test before but now failed with ⌜unrecognized_residue⌟ error! Its run-log could be found in ⌜271D⌟
PDB 2A2Z was passing test before but now failed with ⌜unrecognized_residue⌟ error! Its run-log could be found in ⌜2A2Z⌟
PDB 2A30 was passing test before but now failed with ⌜unrecognized_residue⌟ error! Its run-log could be found in ⌜2A30⌟
PDB 2AT1 was passing test before but now failed with ⌜fill_missing_atoms⌟ error! Its run-log could be found in ⌜2AT1⌟
PDB 2B0E was passing test before but now failed with ⌜unrecognized_residue⌟ error! Its run-log could be found in ⌜2B0E⌟
PDB 2B46 was passing test before but now failed with ⌜unknown⌟ error! Its run-log could be found in ⌜2B46⌟
PDB 2B4F was passing test before but now failed with ⌜unknown⌟ error! Its run-log could be found in ⌜2B4F⌟
PDB 2BFG was passing test before but now failed with ⌜fill_missing_atoms⌟ error! Its run-log could be found in ⌜2BFG⌟
PDB 2BMZ was passing test before but now failed with ⌜unknown⌟ error! Its run-log could be found in ⌜2BMZ⌟
PDB 2BNJ was passing test before but now failed with ⌜unknown⌟ error! Its run-log could be found in ⌜2BNJ⌟
PDB 2BUM was passing test before but now failed with ⌜fill_missing_atoms⌟ error! Its run-log could be found in ⌜2BUM⌟
PDB 2BUT was passing test before but now failed with ⌜fill_missing_atoms⌟ error! Its run-log could be found in ⌜2BUT⌟
PDB 2BUX was passing test before but now failed with ⌜fill_missing_atoms⌟ error! Its run-log could be found in ⌜2BUX⌟
PDB 2C01 was passing test before but now failed with ⌜ld_chirality⌟ error! Its run-log could be found in ⌜2C01⌟
PDB 2C40 was passing test before but now failed with ⌜unknown⌟ error! Its run-log could be found in ⌜2C40⌟
PDB 2C7F was passing test before but now failed with ⌜unknown⌟ error! Its run-log could be found in ⌜2C7F⌟
PDB 2C8N was passing test before but now failed with ⌜unknown⌟ error! Its run-log could be found in ⌜2C8N⌟
PDB 2CCH was passing test before but now failed with ⌜bad_patch⌟ error! Its run-log could be found in ⌜2CCH⌟
PDB 2CDC was passing test before but now failed with ⌜fill_missing_atoms⌟ error! Its run-log could be found in ⌜2CDC⌟
PDB 2CG9 was passing test before but now failed with ⌜bad_patch⌟ error! Its run-log could be found in ⌜2CG9⌟
PDB 2CNC was passing test before but now failed with ⌜unknown⌟ error! Its run-log could be found in ⌜2CNC⌟
PDB 2D3Y was passing test before but now failed with ⌜unrecognized_residue⌟ error! Its run-log could be found in ⌜2D3Y⌟
PDB 2D43 was passing test before but now failed with ⌜unknown⌟ error! Its run-log could be found in ⌜2D43⌟
PDB 2D44 was passing test before but now failed with ⌜unknown⌟ error! Its run-log could be found in ⌜2D44⌟
PDB 2DP7 was passing test before but now failed with ⌜unrecognized_residue⌟ error! Its run-log could be found in ⌜2DP7⌟
PDB 2DPB was passing test before but now failed with ⌜unrecognized_residue⌟ error! Its run-log could be found in ⌜2DPB⌟
PDB 2E2Q was passing test before but now failed with ⌜fill_missing_atoms⌟ error! Its run-log could be found in ⌜2E2Q⌟
PDB 2F55 was passing test before but now failed with ⌜unrecognized_residue⌟ error! Its run-log could be found in ⌜2F55⌟
PDB 2F5V was passing test before but now failed with ⌜fill_missing_atoms⌟ error! Its run-log could be found in ⌜2F5V⌟
PDB 2FAF was passing test before but now failed with ⌜unrecognized_residue⌟ error! Its run-log could be found in ⌜2FAF⌟
PDB 2FAH was passing test before but now failed with ⌜unrecognized_residue⌟ error! Its run-log could be found in ⌜2FAH⌟
PDB 2FBW was passing test before but now failed with ⌜unrecognized_residue⌟ error! Its run-log could be found in ⌜2FBW⌟
PDB 2GD4 was passing test before but now failed with ⌜unrecognized_residue⌟ error! Its run-log could be found in ⌜2GD4⌟
PDB 2GJW was passing test before but now failed with ⌜unrecognized_residue⌟ error! Its run-log could be found in ⌜2GJW⌟
PDB 2GSM was passing test before but now failed with ⌜fill_missing_atoms⌟ error! Its run-log could be found in ⌜2GSM⌟
PDB 2GXB was passing test before but now failed with ⌜unrecognized_residue⌟ error! Its run-log could be found in ⌜2GXB⌟

PDB 2H2Q was passing test before but now failed with 「unrecognized_residue」 error! Its run-log could be found in 「2H2Q」
PDB 2HOX was passing test before but now failed with 「fill_missing_atoms」 error! Its run-log could be found in 「2HOX」
PDB 2HUO was passing test before but now failed with 「fill_missing_atoms」 error! Its run-log could be found in 「2HUO」
PDB 2I5S was passing test before but now failed with 「unrecognized_residue」 error! Its run-log could be found in 「2I5S」
PDB 2IMH was passing test before but now failed with 「bad_patch」 error! Its run-log could be found in 「2IMH」
PDB 2IX8 was passing test before but now failed with 「bad_patch」 error! Its run-log could be found in 「2IX8」
PDB 2JAU was passing test before but now failed with 「duplicate_atom_name」 error! Its run-log could be found in 「2JAU」
PDB 2JAX was passing test before but now failed with 「bad_patch」 error! Its run-log could be found in 「2JAX」
PDB 2KQD was passing test before but now failed with 「unknown」 error! Its run-log could be found in 「2KQD」
PDB 2KQE was passing test before but now failed with 「unknown」 error! Its run-log could be found in 「2KQE」
PDB 2LZV was passing test before but now failed with 「unrecognized_residue」 error! Its run-log could be found in 「2LZV」
PDB 2NC5 was passing test before but now failed with 「unknown」 error! Its run-log could be found in 「2NC5」
PDB 2NO1 was passing test before but now failed with 「unrecognized_residue」 error! Its run-log could be found in 「2NO1」
PDB 2NQY was passing test before but now failed with 「unknown」 error! Its run-log could be found in 「2NQY」
PDB 2NX3 was passing test before but now failed with 「unknown」 error! Its run-log could be found in 「2NX3」
PDB 2NXG was passing test before but now failed with 「unknown」 error! Its run-log could be found in 「2NXG」
PDB 2O8D was passing test before but now failed with 「unrecognized_residue」 error! Its run-log could be found in 「2O8D」
PDB 2OC8 was passing test before but now failed with 「unrecognized_residue」 error! Its run-log could be found in 「2OC8」
PDB 2ORH was passing test before but now failed with 「unrecognized_residue」 error! Its run-log could be found in 「2ORH」
PDB 2P10 was passing test before but now failed with 「bad_patch」 error! Its run-log could be found in 「2P10」
PDB 2P1S was passing test before but now failed with 「unrecognized_residue」 error! Its run-log could be found in 「2P1S」
PDB 2P37 was passing test before but now failed with 「unrecognized_residue」 error! Its run-log could be found in 「2P37」
PDB 2P39 was passing test before but now failed with 「unrecognized_residue」 error! Its run-log could be found in 「2P39」
PDB 2QJE was passing test before but now failed with 「unrecognized_residue」 error! Its run-log could be found in 「2QJE」
PDB 2QKH was passing test before but now failed with 「unrecognized_residue」 error! Its run-log could be found in 「2QKH」
PDB 2QZ2 was passing test before but now failed with 「unknown」 error! Its run-log could be found in 「2QZ2」
PDB 2QZ3 was passing test before but now failed with 「unknown」 error! Its run-log could be found in 「2QZ3」
PDB 2QZY was passing test before but now failed with 「unrecognized_residue」 error! Its run-log could be found in 「2QZY」
PDB 2TMK was passing test before but now failed with 「duplicate_atom_name」 error! Its run-log could be found in 「2TMK」
PDB 2UUS was passing test before but now failed with 「unrecognized_residue」 error! Its run-log could be found in 「2UUS」
PDB 2UWN was passing test before but now failed with 「unrecognized_residue」 error! Its run-log could be found in 「2UWN」
PDB 2V1E was passing test before but now failed with 「fill_missing_atoms」 error! Its run-log could be found in 「2V1E」
PDB 2V1F was passing test before but now failed with 「fill_missing_atoms」 error! Its run-log could be found in 「2V1F」
PDB 2V1G was passing test before but now failed with 「fill_missing_atoms」 error! Its run-log could be found in 「2V1G」
PDB 2V4V was passing test before but now failed with 「fill_missing_atoms」 error! Its run-log could be found in 「2V4V」
PDB 2V5E was passing test before but now failed with 「unrecognized_residue」 error! Its run-log could be found in 「2V5E」
PDB 2V8E was passing test before but now failed with 「unrecognized_residue」 error! Its run-log could be found in 「2V8E」
PDB 2V92 was passing test before but now failed with 「nu_conformer」 error! Its run-log could be found in 「2V92」
PDB 2VAH was passing test before but now failed with 「unrecognized_residue」 error! Its run-log could be found in 「2VAH」
PDB 2VAI was passing test before but now failed with 「unrecognized_residue」 error! Its run-log could be found in 「2VAI」
PDB 2VGD was passing test before but now failed with 「unknown」 error! Its run-log could be found in 「2VGD」
PDB 2VLC was passing test before but now failed with 「unrecognized_residue」 error! Its run-log could be found in 「2VLC」
PDB 2VM0 was passing test before but now failed with 「fill_missing_atoms」 error! Its run-log could be found in 「2VM0」
PDB 2VP5 was passing test before but now failed with 「unrecognized_residue」 error! Its run-log could be found in 「2VP5」
PDB 2VRQ was passing test before but now failed with 「unknown」 error! Its run-log could be found in 「2VRQ」
PDB 2VWJ was passing test before but now failed with 「unrecognized_residue」 error! Its run-log could be found in 「2VWJ」
PDB 2W5F was passing test before but now failed with 「unknown」 error! Its run-log could be found in 「2W5F」
PDB 2W5O was passing test before but now failed with 「unknown」 error! Its run-log could be found in 「2W5O」
PDB 2WW1 was passing test before but now failed with 「unrecognized_residue」 error! Its run-log could be found in 「2WW1」
PDB 2WW3 was passing test before but now failed with 「unrecognized_residue」 error! Its run-log could be found in 「2WW3」
PDB 2WWI was passing test before but now failed with 「duplicate_atom_name」 error! Its run-log could be found in 「2WWI」
PDB 2WXD was passing test before but now failed with 「fill_missing_atoms」 error! Its run-log could be found in 「2WXD」
PDB 2WYS was passing test before but now failed with 「unknown」 error! Its run-log could be found in 「2WYS」
PDB 2WZE was passing test before but now failed with 「unknown」 error! Its run-log could be found in 「2WZE」
PDB 2X2I was passing test before but now failed with 「fill_missing_atoms」 error! Its run-log could be found in 「2X2I」
PDB 2X87 was passing test before but now failed with 「fill_missing_atoms」 error! Its run-log could be found in 「2X87」
PDB 2X8S was passing test before but now failed with 「unknown」 error! Its run-log could be found in 「2X8S」
PDB 2XU9 was passing test before but now failed with 「fill_missing_atoms」 error! Its run-log could be found in 「2XU9」
PDB 2XY5 was passing test before but now failed with 「unrecognized_residue」 error! Its run-log could be found in 「2XY5」
PDB 2XYL was passing test before but now failed with 「unknown」 error! Its run-log could be found in 「2XYL」
PDB 2Y24 was passing test before but now failed with 「unknown」 error! Its run-log could be found in 「2Y24」
PDB 2Y64 was passing test before but now failed with 「unknown」 error! Its run-log could be found in 「2Y64」
PDB 2Y6K was passing test before but now failed with 「unknown」 error! Its run-log could be found in 「2Y6K」
PDB 2Y6L was passing test before but now failed with 「unknown」 error! Its run-log could be found in 「2Y6L」
PDB 2YAF was passing test before but now failed with 「fill_missing_atoms」 error! Its run-log could be found in 「2YAF」
PDB 2YAH was passing test before but now failed with 「fill_missing_atoms」 error! Its run-log could be found in 「2YAH」
PDB 2YAM was passing test before but now failed with 「fill_missing_atoms」 error! Its run-log could be found in 「2YAM」
PDB 2YAO was passing test before but now failed with 「fill_missing_atoms」 error! Its run-log could be found in 「2YAO」
PDB 2YAQ was passing test before but now failed with 「fill_missing_atoms」 error! Its run-log could be found in 「2YAQ」
PDB 2YAR was passing test before but now failed with 「fill_missing_atoms」 error! Its run-log could be found in 「2YAR」
PDB 2YD8 was passing test before but now failed with 「unrecognized_residue」 error! Its run-log could be found in 「2YD8」
PDB 2YDP was passing test before but now failed with 「fill_missing_atoms」 error! Its run-log could be found in 「2YDP」
PDB 2YDT was passing test before but now failed with 「fill_missing_atoms」 error! Its run-log could be found in 「2YDT」
PDB 2YGQ was passing test before but now failed with 「unrecognized_residue」 error! Its run-log could be found in 「2YGQ」
PDB 2Z2C was passing test before but now failed with 「bad_patch」 error! Its run-log could be found in 「2Z2C」
PDB 2Z6Q was passing test before but now failed with 「unrecognized_residue」 error! Its run-log could be found in 「2Z6Q」
PDB 357D was passing test before but now failed with 「unknown」 error! Its run-log could be found in 「357D」
PDB 367D was passing test before but now failed with 「unrecognized_residue」 error! Its run-log could be found in 「367D」
PDB 3A72 was passing test before but now failed with 「unknown」 error! Its run-log could be found in 「3A72」
PDB 3AKG was passing test before but now failed with 「unknown」 error! Its run-log could be found in 「3AKG」
PDB 3AKH was passing test before but now failed with 「unknown」 error! Its run-log could be found in 「3AKH」

PDB 3BVT was passing test before but now failed with 「unrecognized_residue」 error! Its run-log could be found in 「3BVT」
PDB 3BVU was passing test before but now failed with 「unrecognized_residue」 error! Its run-log could be found in 「3BVU」
PDB 3BVV was passing test before but now failed with 「unrecognized_residue」 error! Its run-log could be found in 「3BVV」
PDB 3BVW was passing test before but now failed with 「unrecognized_residue」 error! Its run-log could be found in 「3BVW」
PDB 3BVX was passing test before but now failed with 「unrecognized_residue」 error! Its run-log could be found in 「3BVX」
PDB 3BWH was passing test before but now failed with 「fill_missing_atoms」 error! Its run-log could be found in 「3BWH」
PDB 3BXD was passing test before but now failed with 「fill_missing_atoms」 error! Its run-log could be found in 「3BXD」
PDB 3C6Q was passing test before but now failed with 「unknown」 error! Its run-log could be found in 「3C6Q」
PDB 3C7F was passing test before but now failed with 「unknown」 error! Its run-log could be found in 「3C7F」
PDB 3C7G was passing test before but now failed with 「unknown」 error! Its run-log could be found in 「3C7G」
PDB 3C7H was passing test before but now failed with 「unknown」 error! Its run-log could be found in 「3C7H」
PDB 3CA0 was passing test before but now failed with 「fill_missing_atoms」 error! Its run-log could be found in 「3CA0」
PDB 3CIK was passing test before but now failed with 「reroot_disconnected」 error! Its run-log could be found in 「3CIK」
PDB 3CU1 was passing test before but now failed with 「unrecognized_residue」 error! Its run-log could be found in 「3CU1」
PDB 3CUI was passing test before but now failed with 「unrecognized_residue」 error! Its run-log could be found in 「3CUI」
PDB 3CUJ was passing test before but now failed with 「unrecognized_residue」 error! Its run-log could be found in 「3CUJ」
PDB 3CWH was passing test before but now failed with 「fill_missing_atoms」 error! Its run-log could be found in 「3CWH」
PDB 3D5Z was passing test before but now failed with 「unknown」 error! Its run-log could be found in 「3D5Z」
PDB 3D61 was passing test before but now failed with 「unknown」 error! Its run-log could be found in 「3D61」
PDB 3DTU was passing test before but now failed with 「fill_missing_atoms」 error! Its run-log could be found in 「3DTU」
PDB 3ENZ was passing test before but now failed with 「fill_missing_atoms」 error! Its run-log could be found in 「3ENZ」
PDB 3EVJ was passing test before but now failed with 「unrecognized_residue」 error! Its run-log could be found in 「3EVJ」
PDB 3FP0 was passing test before but now failed with 「unrecognized_residue」 error! Its run-log could be found in 「3FP0」
PDB 3FYT was passing test before but now failed with 「fill_missing_atoms」 error! Its run-log could be found in 「3FYT」
PDB 3FYU was passing test before but now failed with 「fill_missing_atoms」 error! Its run-log could be found in 「3FYU」
PDB 3G00 was passing test before but now failed with 「unrecognized_residue」 error! Its run-log could be found in 「3G00」
PDB 3G0R was passing test before but now failed with 「unrecognized_residue」 error! Its run-log could be found in 「3G0R」
PDB 3G2D was passing test before but now failed with 「unrecognized_residue」 error! Its run-log could be found in 「3G2D」
PDB 3G38 was passing test before but now failed with 「unrecognized_residue」 error! Its run-log could be found in 「3G38」
PDB 3GA6 was passing test before but now failed with 「unrecognized_residue」 error! Its run-log could be found in 「3GA6」
PDB 3GCH was passing test before but now failed with 「unknown」 error! Its run-log could be found in 「3GCH」
PDB 3GO6 was passing test before but now failed with 「fill_missing_atoms」 error! Its run-log could be found in 「3GO6」
PDB 3GO7 was passing test before but now failed with 「fill_missing_atoms」 error! Its run-log could be found in 「3GO7」
PDB 3HKN was passing test before but now failed with 「unrecognized_residue」 error! Its run-log could be found in 「3HKN」
PDB 3I3Y was passing test before but now failed with 「unknown」 error! Its run-log could be found in 「3I3Y」
PDB 3IJ8 was passing test before but now failed with 「fill_missing_atoms」 error! Its run-log could be found in 「3IJ8」
PDB 3IJ9 was passing test before but now failed with 「fill_missing_atoms」 error! Its run-log could be found in 「3IJ9」
PDB 3IVA was passing test before but now failed with 「nu_conformer」 error! Its run-log could be found in 「3IVA」
PDB 3J4P was passing test before but now failed with 「unrecognized_residue」 error! Its run-log could be found in 「3J4P」
PDB 3KCG was passing test before but now failed with 「unrecognized_residue」 error! Its run-log could be found in 「3KCG」
PDB 3KL5 was passing test before but now failed with 「fill_missing_atoms」 error! Its run-log could be found in 「3KL5」
PDB 3KLE was passing test before but now failed with 「unrecognized_residue」 error! Its run-log could be found in 「3KLE」
PDB 3KLH was passing test before but now failed with 「duplicate_atom_name」 error! Its run-log could be found in 「3KLH」
PDB 3KRW was passing test before but now failed with 「reroot_disconnected」 error! Its run-log could be found in 「3KRW」
PDB 3KRX was passing test before but now failed with 「reroot_disconnected」 error! Its run-log could be found in 「3KRX」
PDB 3KVR was passing test before but now failed with 「unknown」 error! Its run-log could be found in 「3KVR」
PDB 3KVV was passing test before but now failed with 「unknown」 error! Its run-log could be found in 「3KVV」
PDB 3KVY was passing test before but now failed with 「unknown」 error! Its run-log could be found in 「3KVY」
PDB 3L4J was passing test before but now failed with 「bad_patch」 error! Its run-log could be found in 「3L4J」
PDB 3L4K was passing test before but now failed with 「bad_patch」 error! Its run-log could be found in 「3L4K」
PDB 3LDJ was passing test before but now failed with 「unrecognized_residue」 error! Its run-log could be found in 「3LDJ」
PDB 3M9X was passing test before but now failed with 「unknown」 error! Its run-log could be found in 「3M9X」
PDB 3MA0 was passing test before but now failed with 「unknown」 error! Its run-log could be found in 「3MA0」
PDB 3MMD was passing test before but now failed with 「unknown」 error! Its run-log could be found in 「3MMD」
PDB 3MSD was passing test before but now failed with 「unknown」 error! Its run-log could be found in 「3MSD」
PDB 3MSG was passing test before but now failed with 「unknown」 error! Its run-log could be found in 「3MSG」
PDB 3MUA was passing test before but now failed with 「unknown」 error! Its run-log could be found in 「3MUA」
PDB 3MUI was passing test before but now failed with 「unknown」 error! Its run-log could be found in 「3MUI」
PDB 3N1X was passing test before but now failed with 「fill_missing_atoms」 error! Its run-log could be found in 「3N1X」
PDB 3NJ3 was passing test before but now failed with 「unknown」 error! Its run-log could be found in 「3NJ3」
PDB 3NPE was passing test before but now failed with 「fill_missing_atoms」 error! Its run-log could be found in 「3NPE」
PDB 3OB0 was passing test before but now failed with 「unrecognized_residue」 error! Its run-log could be found in 「3OB0」
PDB 3OMA was passing test before but now failed with 「fill_missing_atoms」 error! Its run-log could be found in 「3OMA」
PDB 3OMI was passing test before but now failed with 「fill_missing_atoms」 error! Its run-log could be found in 「3OMI」
PDB 3OMN was passing test before but now failed with 「fill_missing_atoms」 error! Its run-log could be found in 「3OMN」
PDB 3PER was passing test before but now failed with 「fill_missing_atoms」 error! Its run-log could be found in 「3PER」
PDB 3PF7 was passing test before but now failed with 「fill_missing_atoms」 error! Its run-log could be found in 「3PF7」
PDB 3PMA was passing test before but now failed with 「unrecognized_residue」 error! Its run-log could be found in 「3PMA」
PDB 3PSC was passing test before but now failed with 「reroot_disconnected」 error! Its run-log could be found in 「3PSC」
PDB 3PVU was passing test before but now failed with 「reroot_disconnected」 error! Its run-log could be found in 「3PVU」
PDB 3PVW was passing test before but now failed with 「reroot_disconnected」 error! Its run-log could be found in 「3PVW」
PDB 3Q1G was passing test before but now failed with 「fill_missing_atoms」 error! Its run-log could be found in 「3Q1G」
PDB 3Q7G was passing test before but now failed with 「unrecognized_residue」 error! Its run-log could be found in 「3Q7G」
PDB 3Q8K was passing test before but now failed with 「fill_missing_atoms」 error! Its run-log could be found in 「3Q8K」
PDB 3QBA was passing test before but now failed with 「unrecognized_residue」 error! Its run-log could be found in 「3QBA」
PDB 3QDX was passing test before but now failed with 「fill_missing_atoms」 error! Its run-log could be found in 「3QDX」
PDB 3QDY was passing test before but now failed with 「fill_missing_atoms」 error! Its run-log could be found in 「3QDY」
PDB 3QEF was passing test before but now failed with 「unknown」 error! Its run-log could be found in 「3QEF」
PDB 3QRC was passing test before but now failed with 「unrecognized_residue」 error! Its run-log could be found in 「3QRC」
PDB 3RCY was passing test before but now failed with 「unknown」 error! Its run-log could be found in 「3RCY」
PDB 3RDK was passing test before but now failed with 「fill_missing_atoms」 error! Its run-log could be found in 「3RDK」

PDB 3RN9 was passing test before but now failed with 「fill_missing_atoms」 error! Its run-log could be found in 「3RN9」
PDB 3RNA was passing test before but now failed with 「fill_missing_atoms」 error! Its run-log could be found in 「3RNA」
PDB 3RNB was passing test before but now failed with 「fill_missing_atoms」 error! Its run-log could be found in 「3RNB」
PDB 3RNC was passing test before but now failed with 「fill_missing_atoms」 error! Its run-log could be found in 「3RNC」
PDB 3RNE was passing test before but now failed with 「fill_missing_atoms」 error! Its run-log could be found in 「3RNE」
PDB 3RNG was passing test before but now failed with 「fill_missing_atoms」 error! Its run-log could be found in 「3RNG」
PDB 3S5Y was passing test before but now failed with 「fill_missing_atoms」 error! Its run-log could be found in 「3S5Y」
PDB 3S5Z was passing test before but now failed with 「fill_missing_atoms」 error! Its run-log could be found in 「3S5Z」
PDB 3SAS was passing test before but now failed with 「unknown_atom_name」 error! Its run-log could be found in 「3SAS」
PDB 3SAT was passing test before but now failed with 「unknown_atom_name」 error! Its run-log could be found in 「3SAT」
PDB 3SAU was passing test before but now failed with 「unknown_atom_name」 error! Its run-log could be found in 「3SAU」
PDB 3T5G was passing test before but now failed with 「reroot_disconnected」 error! Its run-log could be found in 「3T5G」
PDB 3T5I was passing test before but now failed with 「reroot_disconnected」 error! Its run-log could be found in 「3T5I」
PDB 3TA2 was passing test before but now failed with 「nu_conformer」 error! Its run-log could be found in 「3TA2」
PDB 3TI3 was passing test before but now failed with 「fill_missing_atoms」 error! Its run-log could be found in 「3TI3」
PDB 3TI8 was passing test before but now failed with 「fill_missing_atoms」 error! Its run-log could be found in 「3TI8」
PDB 3TIA was passing test before but now failed with 「fill_missing_atoms」 error! Its run-log could be found in 「3TIA」
PDB 3UER was passing test before but now failed with 「unrecognized_residue」 error! Its run-log could be found in 「3UER」
PDB 3UG4 was passing test before but now failed with 「unknown」 error! Its run-log could be found in 「3UG4」
PDB 3UG5 was passing test before but now failed with 「unknown」 error! Its run-log could be found in 「3UG5」
PDB 3UGW was passing test before but now failed with 「unrecognized_residue」 error! Its run-log could be found in 「3UGW」
PDB 3USU was passing test before but now failed with 「fill_missing_atoms」 error! Its run-log could be found in 「3USU」
PDB 3UZS was passing test before but now failed with 「reroot_disconnected」 error! Its run-log could be found in 「3UZS」
PDB 3V8V was passing test before but now failed with 「unrecognized_residue」 error! Its run-log could be found in 「3V8V」
PDB 3V97 was passing test before but now failed with 「unrecognized_residue」 error! Its run-log could be found in 「3V97」
PDB 3VAF was passing test before but now failed with 「unrecognized_residue」 error! Its run-log could be found in 「3VAF」
PDB 3VAG was passing test before but now failed with 「unrecognized_residue」 error! Its run-log could be found in 「3VAG」
PDB 3VAH was passing test before but now failed with 「unrecognized_residue」 error! Its run-log could be found in 「3VAH」
PDB 3VAI was passing test before but now failed with 「unrecognized_residue」 error! Its run-log could be found in 「3VAI」
PDB 3VAJ was passing test before but now failed with 「unrecognized_residue」 error! Its run-log could be found in 「3VAJ」
PDB 3VAK was passing test before but now failed with 「unrecognized_residue」 error! Its run-log could be found in 「3VAK」
PDB 3VAL was passing test before but now failed with 「unrecognized_residue」 error! Its run-log could be found in 「3VAL」
PDB 3VAM was passing test before but now failed with 「unrecognized_residue」 error! Its run-log could be found in 「3VAM」
PDB 3VPL was passing test before but now failed with 「unknown」 error! Its run-log could be found in 「3VPL」
PDB 3VSJ was passing test before but now failed with 「fill_missing_atoms」 error! Its run-log could be found in 「3VSJ」
PDB 3VSU was passing test before but now failed with 「unknown」 error! Its run-log could be found in 「3VSU」
PDB 3VSV was passing test before but now failed with 「fill_missing_atoms」 error! Its run-log could be found in 「3VSV」
PDB 3VV9 was passing test before but now failed with 「fill_missing_atoms」 error! Its run-log could be found in 「3VV9」
PDB 3VVA was passing test before but now failed with 「fill_missing_atoms」 error! Its run-log could be found in 「3VVA」
PDB 3VXC was passing test before but now failed with 「unknown」 error! Its run-log could be found in 「3VXC」
PDB 3VZM was passing test before but now failed with 「unknown」 error! Its run-log could be found in 「3VZM」
PDB 3VZN was passing test before but now failed with 「unknown」 error! Its run-log could be found in 「3VZN」
PDB 3VZO was passing test before but now failed with 「unknown」 error! Its run-log could be found in 「3VZO」
PDB 3W25 was passing test before but now failed with 「unknown」 error! Its run-log could be found in 「3W25」
PDB 3W26 was passing test before but now failed with 「unknown」 error! Its run-log could be found in 「3W26」
PDB 3W27 was passing test before but now failed with 「unknown」 error! Its run-log could be found in 「3W27」
PDB 3W28 was passing test before but now failed with 「unknown」 error! Its run-log could be found in 「3W28」
PDB 3W29 was passing test before but now failed with 「unknown」 error! Its run-log could be found in 「3W29」
PDB 3W54 was passing test before but now failed with 「fill_missing_atoms」 error! Its run-log could be found in 「3W54」
PDB 3WKX was passing test before but now failed with 「fill_missing_atoms」 error! Its run-log could be found in 「3WKX」
PDB 3WLH was passing test before but now failed with 「fill_missing_atoms」 error! Its run-log could be found in 「3WLH」
PDB 3WLJ was passing test before but now failed with 「fill_missing_atoms」 error! Its run-log could be found in 「3WLJ」
PDB 3WLK was passing test before but now failed with 「fill_missing_atoms」 error! Its run-log could be found in 「3WLK」
PDB 3WLL was passing test before but now failed with 「fill_missing_atoms」 error! Its run-log could be found in 「3WLL」
PDB 3WN0 was passing test before but now failed with 「fill_missing_atoms」 error! Its run-log could be found in 「3WN0」
PDB 3WN1 was passing test before but now failed with 「unknown」 error! Its run-log could be found in 「3WN1」
PDB 3WN2 was passing test before but now failed with 「unknown」 error! Its run-log could be found in 「3WN2」
PDB 3WP6 was passing test before but now failed with 「unknown」 error! Its run-log could be found in 「3WP6」
PDB 3WRG was passing test before but now failed with 「fill_missing_atoms」 error! Its run-log could be found in 「3WRG」
PDB 3WUE was passing test before but now failed with 「unknown」 error! Its run-log could be found in 「3WUE」
PDB 3WUG was passing test before but now failed with 「unknown」 error! Its run-log could be found in 「3WUG」
PDB 3ZFO was passing test before but now failed with 「fill_missing_atoms」 error! Its run-log could be found in 「3ZFO」
PDB 3ZFR was passing test before but now failed with 「fill_missing_atoms」 error! Its run-log could be found in 「3ZFR」
PDB 3ZKK was passing test before but now failed with 「unknown」 error! Its run-log could be found in 「3ZKK」
PDB 3ZKL was passing test before but now failed with 「unknown」 error! Its run-log could be found in 「3ZKL」
PDB 3ZSE was passing test before but now failed with 「unknown」 error! Its run-log could be found in 「3ZSE」
PDB 3ZXK was passing test before but now failed with 「unknown」 error! Its run-log could be found in 「3ZXK」
PDB 4A5G was passing test before but now failed with 「fill_missing_atoms」 error! Its run-log could be found in 「4A5G」
PDB 4A68 was passing test before but now failed with 「fill_missing_atoms」 error! Its run-log could be found in 「4A68」
PDB 4AC7 was passing test before but now failed with 「fill_missing_atoms」 error! Its run-log could be found in 「4AC7」
PDB 4AYP was passing test before but now failed with 「unrecognized_residue」 error! Its run-log could be found in 「4AYP」
PDB 4BQC was passing test before but now failed with 「unrecognized_residue」 error! Its run-log could be found in 「4BQC」
PDB 4BQF was passing test before but now failed with 「fill_missing_atoms」 error! Its run-log could be found in 「4BQF」
PDB 4C0N was passing test before but now failed with 「fill_missing_atoms」 error! Its run-log could be found in 「4C0N」
PDB 4C1P was passing test before but now failed with 「unknown」 error! Its run-log could be found in 「4C1P」
PDB 4C1T was passing test before but now failed with 「unknown」 error! Its run-log could be found in 「4C1T」
PDB 4C1U was passing test before but now failed with 「unknown」 error! Its run-log could be found in 「4C1U」
PDB 4CDR was passing test before but now failed with 「bad_patch」 error! Its run-log could be found in 「4CDR」
PDB 4CEU was passing test before but now failed with 「fill_missing_atoms」 error! Its run-log could be found in 「4CEU」
PDB 4D4Z was passing test before but now failed with 「fill_missing_atoms」 error! Its run-log could be found in 「4D4Z」
PDB 4D5I was passing test before but now failed with 「unknown」 error! Its run-log could be found in 「4D5I」

PDB 4D5J was passing test before but now failed with 「unknown」 error! Its run-log could be found in 「4D5J」
PDB 4D5O was passing test before but now failed with 「fill_missing_atoms」 error! Its run-log could be found in 「4D5O」
PDB 4D5P was passing test before but now failed with 「unknown」 error! Its run-log could be found in 「4D5P」
PDB 4D5Q was passing test before but now failed with 「unknown」 error! Its run-log could be found in 「4D5Q」
PDB 4D5V was passing test before but now failed with 「unknown」 error! Its run-log could be found in 「4D5V」
PDB 4E9V was passing test before but now failed with 「fill_missing_atoms」 error! Its run-log could be found in 「4E9V」
PDB 4EAQ was passing test before but now failed with 「duplicate_atom_name」 error! Its run-log could be found in 「4EAQ」
PDB 4EF3 was passing test before but now failed with 「fill_missing_atoms」 error! Its run-log could be found in 「4EF3」
PDB 4FLY was passing test before but now failed with 「unrecognized_residue」 error! Its run-log could be found in 「4FLY」
PDB 4FM0 was passing test before but now failed with 「unrecognized_residue」 error! Its run-log could be found in 「4FM0」
PDB 4FM1 was passing test before but now failed with 「unrecognized_residue」 error! Its run-log could be found in 「4FM1」
PDB 4FM2 was passing test before but now failed with 「unrecognized_residue」 error! Its run-log could be found in 「4FM2」
PDB 4G4N was passing test before but now failed with 「unknown_atom_name」 error! Its run-log could be found in 「4G4N」
PDB 4G4O was passing test before but now failed with 「unknown_atom_name」 error! Its run-log could be found in 「4G4O」
PDB 4G4Q was passing test before but now failed with 「unknown_atom_name」 error! Its run-log could be found in 「4G4Q」
PDB 4G4R was passing test before but now failed with 「unknown_atom_name」 error! Its run-log could be found in 「4G4R」
PDB 4G68 was passing test before but now failed with 「unknown」 error! Its run-log could be found in 「4G68」
PDB 4GBY was passing test before but now failed with 「fill_missing_atoms」 error! Its run-log could be found in 「4GBY」
PDB 4GFH was passing test before but now failed with 「bad_patch」 error! Its run-log could be found in 「4GFH」
PDB 4GMD was passing test before but now failed with 「duplicate_atom_name」 error! Its run-log could be found in 「4GMD」
PDB 4H9U was passing test before but now failed with 「fill_missing_atoms」 error! Its run-log could be found in 「4H9U」
PDB 4H9V was passing test before but now failed with 「fill_missing_atoms」 error! Its run-log could be found in 「4H9V」
PDB 4H9X was passing test before but now failed with 「fill_missing_atoms」 error! Its run-log could be found in 「4H9X」
PDB 4H9Y was passing test before but now failed with 「fill_missing_atoms」 error! Its run-log could be found in 「4H9Y」
PDB 4HA0 was passing test before but now failed with 「fill_missing_atoms」 error! Its run-log could be found in 「4HA0」
PDB 4HAK was passing test before but now failed with 「fill_missing_atoms」 error! Its run-log could be found in 「4HAK」
PDB 4HAL was passing test before but now failed with 「fill_missing_atoms」 error! Its run-log could be found in 「4HAL」
PDB 4HK8 was passing test before but now failed with 「unknown」 error! Its run-log could be found in 「4HK8」
PDB 4HK9 was passing test before but now failed with 「unknown」 error! Its run-log could be found in 「4HK9」
PDB 4HKW was passing test before but now failed with 「unknown」 error! Its run-log could be found in 「4HKW」
PDB 4HZW was passing test before but now failed with 「fill_missing_atoms」 error! Its run-log could be found in 「4HZW」
PDB 4IPN was passing test before but now failed with 「unrecognized_residue」 error! Its run-log could be found in 「4IPN」
PDB 4JCK was passing test before but now failed with 「unrecognized_residue」 error! Its run-log could be found in 「4JCK」
PDB 4JXG was passing test before but now failed with 「fill_missing_atoms」 error! Its run-log could be found in 「4JXG」
PDB 4KAS was passing test before but now failed with 「unrecognized_residue」 error! Its run-log could be found in 「4KAS」
PDB 4KAT was passing test before but now failed with 「unrecognized_residue」 error! Its run-log could be found in 「4KAT」
PDB 4L4P was passing test before but now failed with 「unknown」 error! Its run-log could be found in 「4L4P」
PDB 4LR9 was passing test before but now failed with 「fill_missing_atoms」 error! Its run-log could be found in 「4LR9」
PDB 4LRB was passing test before but now failed with 「fill_missing_atoms」 error! Its run-log could be found in 「4LRB」
PDB 4LRE was passing test before but now failed with 「fill_missing_atoms」 error! Its run-log could be found in 「4LRE」
PDB 4M4K was passing test before but now failed with 「unknown」 error! Its run-log could be found in 「4M4K」
PDB 4MAV was passing test before but now failed with 「unknown」 error! Its run-log could be found in 「4MAV」
PDB 4MDX was passing test before but now failed with 「unrecognized_residue」 error! Its run-log could be found in 「4MDX」
PDB 4MWU was passing test before but now failed with 「fill_missing_atoms」 error! Its run-log could be found in 「4MWU」
PDB 4MWY was passing test before but now failed with 「fill_missing_atoms」 error! Its run-log could be found in 「4MWY」
PDB 4N2R was passing test before but now failed with 「fill_missing_atoms」 error! Its run-log could be found in 「4N2R」
PDB 4NER was passing test before but now failed with 「fill_missing_atoms」 error! Its run-log could be found in 「4NER」
PDB 4O8O was passing test before but now failed with 「unknown」 error! Its run-log could be found in 「4O8O」
PDB 4O8P was passing test before but now failed with 「unknown」 error! Its run-log could be found in 「4O8P」
PDB 4OJI was passing test before but now failed with 「unrecognized_residue」 error! Its run-log could be found in 「4OJI」
PDB 4P9C was passing test before but now failed with 「unrecognized_residue」 error! Its run-log could be found in 「4P9C」
PDB 4PMD was passing test before but now failed with 「unknown」 error! Its run-log could be found in 「4PMD」
PDB 4PMY was passing test before but now failed with 「fill_missing_atoms」 error! Its run-log could be found in 「4PMY」
PDB 4PMZ was passing test before but now failed with 「unknown」 error! Its run-log could be found in 「4PMZ」
PDB 4PN2 was passing test before but now failed with 「unknown」 error! Its run-log could be found in 「4PN2」
PDB 4PRW was passing test before but now failed with 「unknown」 error! Its run-log could be found in 「4PRW」
PDB 4PUD was passing test before but now failed with 「unknown」 error! Its run-log could be found in 「4PUD」
PDB 4PUE was passing test before but now failed with 「unknown」 error! Its run-log could be found in 「4PUE」
PDB 4PVI was passing test before but now failed with 「unknown」 error! Its run-log could be found in 「4PVI」
PDB 4Q27 was passing test before but now failed with 「unrecognized_residue」 error! Its run-log could be found in 「4Q27」
PDB 4QB6 was passing test before but now failed with 「fill_missing_atoms」 error! Its run-log could be found in 「4QB6」
PDB 4QEH was passing test before but now failed with 「unknown」 error! Its run-log could be found in 「4QEH」
PDB 4QN6 was passing test before but now failed with 「fill_missing_atoms」 error! Its run-log could be found in 「4QN6」
PDB 4QPW was passing test before but now failed with 「unknown」 error! Its run-log could be found in 「4QPW」
PDB 4R9W was passing test before but now failed with 「unrecognized_residue」 error! Its run-log could be found in 「4R9W」
PDB 4RGE was passing test before but now failed with 「unrecognized_residue」 error! Its run-log could be found in 「4RGE」
PDB 4RGF was passing test before but now failed with 「unrecognized_residue」 error! Its run-log could be found in 「4RGF」
PDB 4RHN was passing test before but now failed with 「unknown」 error! Its run-log could be found in 「4RHN」
PDB 4RK0 was passing test before but now failed with 「unknown」 error! Its run-log could be found in 「4RK0」
PDB 4RK1 was passing test before but now failed with 「fill_missing_atoms」 error! Its run-log could be found in 「4RK1」
PDB 4RYK was passing test before but now failed with 「fill_missing_atoms」 error! Its run-log could be found in 「4RYK」
PDB 4RZT was passing test before but now failed with 「unrecognized_residue」 error! Its run-log could be found in 「4RZT」
PDB 4TU7 was passing test before but now failed with 「unrecognized_residue」 error! Its run-log could be found in 「4TU7」
PDB 4TU8 was passing test before but now failed with 「unrecognized_residue」 error! Its run-log could be found in 「4TU8」
PDB 4TU9 was passing test before but now failed with 「unrecognized_residue」 error! Its run-log could be found in 「4TU9」
PDB 4U2Z was passing test before but now failed with 「unrecognized_residue」 error! Its run-log could be found in 「4U2Z」
PDB 4U5I was passing test before but now failed with 「unknown」 error! Its run-log could be found in 「4U5I」
PDB 4UCW was passing test before but now failed with 「missing_disulfide_partner」 error! Its run-log could be found in 「4UCW」
PDB 4UZ6 was passing test before but now failed with 「unrecognized_residue」 error! Its run-log could be found in 「4UZ6」
PDB 4UZ9 was passing test before but now failed with 「unrecognized_residue」 error! Its run-log could be found in 「4UZ9」

PDB 4WEF was passing test before but now failed with 「fill_missing_atoms」 error! Its run-log could be found in 「4WEF」
PDB 4WEG was passing test before but now failed with 「fill_missing_atoms」 error! Its run-log could be found in 「4WEG」
PDB 4X7R was passing test before but now failed with 「unrecognized_residue」 error! Its run-log could be found in 「4X7R」
PDB 4X91 was passing test before but now failed with 「unrecognized_residue」 error! Its run-log could be found in 「4X91」
PDB 4XCK was passing test before but now failed with 「fill_missing_atoms」 error! Its run-log could be found in 「4XCK」
PDB 4XDA was passing test before but now failed with 「fill_missing_atoms」 error! Its run-log could be found in 「4XDA」
PDB 4XJA was passing test before but now failed with 「fill_missing_atoms」 error! Its run-log could be found in 「4XJA」
PDB 4XUQ was passing test before but now failed with 「unknown」 error! Its run-log could be found in 「4XUQ」
PDB 4XUR was passing test before but now failed with 「unknown」 error! Its run-log could be found in 「4XUR」
PDB 4XWO was passing test before but now failed with 「bad_patch」 error! Its run-log could be found in 「4XWO」
PDB 4Y27 was passing test before but now failed with 「unknown」 error! Its run-log could be found in 「4Y27」
PDB 4YGK was passing test before but now failed with 「fill_missing_atoms」 error! Its run-log could be found in 「4YGK」
PDB 4YGL was passing test before but now failed with 「fill_missing_atoms」 error! Its run-log could be found in 「4YGL」
PDB 4YWH was passing test before but now failed with 「unknown」 error! Its run-log could be found in 「4YWH」
PDB 4YZ5 was passing test before but now failed with 「fill_missing_atoms」 error! Its run-log could be found in 「4YZ5」
PDB 4Z8S was passing test before but now failed with 「fill_missing_atoms」 error! Its run-log could be found in 「4Z8S」
PDB 4ZE9 was passing test before but now failed with 「unrecognized_residue」 error! Its run-log could be found in 「4ZE9」
PDB 4ZEB was passing test before but now failed with 「unrecognized_residue」 error! Its run-log could be found in 「4ZEB」
PDB 4ZFU was passing test before but now failed with 「fill_missing_atoms」 error! Its run-log could be found in 「4ZFU」
PDB 4ZFW was passing test before but now failed with 「fill_missing_atoms」 error! Its run-log could be found in 「4ZFW」
PDB 4ZGR was passing test before but now failed with 「fill_missing_atoms」 error! Its run-log could be found in 「4ZGR」
PDB 4ZLB was passing test before but now failed with 「fill_missing_atoms」 error! Its run-log could be found in 「4ZLB」
PDB 4ZRV was passing test before but now failed with 「unrecognized_residue」 error! Its run-log could be found in 「4ZRV」
PDB 4ZTT was passing test before but now failed with 「fill_missing_atoms」 error! Its run-log could be found in 「4ZTT」
PDB 5A03 was passing test before but now failed with 「fill_missing_atoms」 error! Its run-log could be found in 「5A03」
PDB 5A6L was passing test before but now failed with 「unknown」 error! Its run-log could be found in 「5A6L」
PDB 5A6M was passing test before but now failed with 「unknown」 error! Its run-log could be found in 「5A6M」
PDB 5ACU was passing test before but now failed with 「fill_missing_atoms」 error! Its run-log could be found in 「5ACU」
PDB 5ACV was passing test before but now failed with 「fill_missing_atoms」 error! Its run-log could be found in 「5ACV」
PDB 5ACW was passing test before but now failed with 「fill_missing_atoms」 error! Its run-log could be found in 「5ACW」
PDB 5ACX was passing test before but now failed with 「fill_missing_atoms」 error! Its run-log could be found in 「5ACX」
PDB 5AE6 was passing test before but now failed with 「unknown」 error! Its run-log could be found in 「5AE6」
PDB 5AFA was passing test before but now failed with 「fill_missing_atoms」 error! Its run-log could be found in 「5AFA」
PDB 5AOG was passing test before but now failed with 「fill_missing_atoms」 error! Its run-log could be found in 「5AOG」
PDB 5B2O was passing test before but now failed with 「unrecognized_residue」 error! Its run-log could be found in 「5B2O」
PDB 5B2Q was passing test before but now failed with 「unrecognized_residue」 error! Its run-log could be found in 「5B2Q」
PDB 5BQC was passing test before but now failed with 「unrecognized_residue」 error! Its run-log could be found in 「5BQC」
PDB 5BU2 was passing test before but now failed with 「unknown」 error! Its run-log could be found in 「5BU2」
PDB 5C1M was passing test before but now failed with 「unrecognized_residue」 error! Its run-log could be found in 「5C1M」
PDB 5CJH was passing test before but now failed with 「fill_missing_atoms」 error! Its run-log could be found in 「5CJH」
PDB 5COY was passing test before but now failed with 「unrecognized_residue」 error! Its run-log could be found in 「5COY」
PDB 5CR2 was passing test before but now failed with 「unrecognized_residue」 error! Its run-log could be found in 「5CR2」
PDB 5D15 was passing test before but now failed with 「bad_patch」 error! Its run-log could be found in 「5D15」
PDB 5D4Y was passing test before but now failed with 「unknown」 error! Its run-log could be found in 「5D4Y」
PDB 5DLO was passing test before but now failed with 「unrecognized_residue」 error! Its run-log could be found in 「5DLO」
PDB 5E8F was passing test before but now failed with 「reroot_disconnected」 error! Its run-log could be found in 「5E8F」
PDB 5EV1 was passing test before but now failed with 「unrecognized_residue」 error! Its run-log could be found in 「5EV1」
PDB 5EV2 was passing test before but now failed with 「unrecognized_residue」 error! Its run-log could be found in 「5EV2」
PDB 5EV3 was passing test before but now failed with 「unrecognized_residue」 error! Its run-log could be found in 「5EV3」
PDB 5EV4 was passing test before but now failed with 「unrecognized_residue」 error! Its run-log could be found in 「5EV4」
PDB 5F2U was passing test before but now failed with 「reroot_disconnected」 error! Its run-log could be found in 「5F2U」
PDB 5F9T was passing test before but now failed with 「fill_missing_atoms」 error! Its run-log could be found in 「5F9T」
PDB 5FBD was passing test before but now failed with 「unrecognized_residue」 error! Its run-log could be found in 「5FBD」
PDB 5FBG was passing test before but now failed with 「unrecognized_residue」 error! Its run-log could be found in 「5FBG」
PDB 5FQA was passing test before but now failed with 「fill_missing_atoms」 error! Its run-log could be found in 「5FQA」
PDB 5FSD was passing test before but now failed with 「fill_missing_atoms」 error! Its run-log could be found in 「5FSD」
PDB 5FSE was passing test before but now failed with 「fill_missing_atoms」 error! Its run-log could be found in 「5FSE」
PDB 5G4H was passing test before but now failed with 「fill_missing_atoms」 error! Its run-log could be found in 「5G4H」
PDB 5G62 was passing test before but now failed with 「unrecognized_residue」 error! Its run-log could be found in 「5G62」
PDB 5GLM was passing test before but now failed with 「unknown」 error! Its run-log could be found in 「5GLM」
PDB 5GLN was passing test before but now failed with 「unknown」 error! Its run-log could be found in 「5GLN」
PDB 5GLO was passing test before but now failed with 「fill_missing_atoms」 error! Its run-log could be found in 「5GLO」
PDB 5GLQ was passing test before but now failed with 「unknown」 error! Its run-log could be found in 「5GLQ」
PDB 5GLR was passing test before but now failed with 「unknown」 error! Its run-log could be found in 「5GLR」
PDB 5GN7 was passing test before but now failed with 「fill_missing_atoms」 error! Its run-log could be found in 「5GN7」
PDB 5GQD was passing test before but now failed with 「unknown」 error! Its run-log could be found in 「5GQD」
PDB 5GQE was passing test before but now failed with 「unknown」 error! Its run-log could be found in 「5GQE」
PDB 5HJO was passing test before but now failed with 「unrecognized_residue」 error! Its run-log could be found in 「5HJO」
PDB 5HK0 was passing test before but now failed with 「unrecognized_residue」 error! Its run-log could be found in 「5HK0」
PDB 5HK3 was passing test before but now failed with 「unrecognized_residue」 error! Its run-log could be found in 「5HK3」
PDB 5HKC was passing test before but now failed with 「unrecognized_residue」 error! Its run-log could be found in 「5HKC」
PDB 5HO9 was passing test before but now failed with 「unknown」 error! Its run-log could be found in 「5HO9」
PDB 5HOF was passing test before but now failed with 「unknown」 error! Its run-log could be found in 「5HOF」
PDB 5HON was passing test before but now failed with 「unknown」 error! Its run-log could be found in 「5HON」
PDB 5HPO was passing test before but now failed with 「fill_missing_atoms」 error! Its run-log could be found in 「5HPO」
PDB 5HQJ was passing test before but now failed with 「fill_missing_atoms」 error! Its run-log could be found in 「5HQJ」
PDB 5IBQ was passing test before but now failed with 「fill_missing_atoms」 error! Its run-log could be found in 「5IBQ」
PDB 5IJC was passing test before but now failed with 「unrecognized_residue」 error! Its run-log could be found in 「5IJC」
PDB 5K6N was passing test before but now failed with 「fill_missing_atoms」 error! Its run-log could be found in 「5K6N」
PDB 5K73 was passing test before but now failed with 「fill_missing_atoms」 error! Its run-log could be found in 「5K73」
PDB 5K77 was passing test before but now failed with 「fill_missing_atoms」 error! Its run-log could be found in 「5K77」

PDB 5K8Z was passing test before but now failed with 「fill_missing_atoms」 error! Its run-log could be found in 「5K8Z」
PDB 5KK7 was passing test before but now failed with 「unrecognized_residue」 error! Its run-log could be found in 「5KK7」
PDB 5KVY was passing test before but now failed with 「unrecognized_residue」 error! Its run-log could be found in 「5KVY」
PDB 5KW1 was passing test before but now failed with 「unrecognized_residue」 error! Its run-log could be found in 「5KW1」
PDB 5KW6 was passing test before but now failed with 「unrecognized_residue」 error! Its run-log could be found in 「5KW6」
PDB 5KXB was passing test before but now failed with 「fill_missing_atoms」 error! Its run-log could be found in 「5KXB」
PDB 5KXD was passing test before but now failed with 「fill_missing_atoms」 error! Its run-log could be found in 「5KXD」
PDB 5KXE was passing test before but now failed with 「fill_missing_atoms」 error! Its run-log could be found in 「5KXE」
PDB 5L6F was passing test before but now failed with 「bad_patch」 error! Its run-log could be found in 「5L6F」
PDB 5L6G was passing test before but now failed with 「fill_missing_atoms」 error! Its run-log could be found in 「5L6G」
PDB 5LA1 was passing test before but now failed with 「fill_missing_atoms」 error! Its run-log could be found in 「5LA1」
PDB 5LA2 was passing test before but now failed with 「unknown」 error! Its run-log could be found in 「5LA2」
PDB 5LAL was passing test before but now failed with 「fill_missing_atoms」 error! Its run-log could be found in 「5LAL」
PDB 5LSH was passing test before but now failed with 「unrecognized_residue」 error! Its run-log could be found in 「5LSH」
PDB 5M1Z was passing test before but now failed with 「unknown」 error! Its run-log could be found in 「5M1Z」
PDB 5M4G was passing test before but now failed with 「fill_missing_atoms」 error! Its run-log could be found in 「5M4G」
PDB 5M4L was passing test before but now failed with 「fill_missing_atoms」 error! Its run-log could be found in 「5M4L」
PDB 5M4Q was passing test before but now failed with 「fill_missing_atoms」 error! Its run-log could be found in 「5M4Q」
PDB 5M77 was passing test before but now failed with 「fill_missing_atoms」 error! Its run-log could be found in 「5M77」
PDB 5M93 was passing test before but now failed with 「unknown」 error! Its run-log could be found in 「5M93」
PDB 5MBY was passing test before but now failed with 「fill_missing_atoms」 error! Its run-log could be found in 「5MBY」
PDB 5MBZ was passing test before but now failed with 「fill_missing_atoms」 error! Its run-log could be found in 「5MBZ」
PDB 5MC0 was passing test before but now failed with 「fill_missing_atoms」 error! Its run-log could be found in 「5MC0」
PDB 5MC3 was passing test before but now failed with 「fill_missing_atoms」 error! Its run-log could be found in 「5MC3」
PDB 5MC4 was passing test before but now failed with 「fill_missing_atoms」 error! Its run-log could be found in 「5MC4」
PDB 5MC5 was passing test before but now failed with 「fill_missing_atoms」 error! Its run-log could be found in 「5MC5」
PDB 5MQS was passing test before but now failed with 「fill_missing_atoms」 error! Its run-log could be found in 「5MQS」
PDB 5MUI was passing test before but now failed with 「fill_missing_atoms」 error! Its run-log could be found in 「5MUI」
PDB 5MUJ was passing test before but now failed with 「fill_missing_atoms」 error! Its run-log could be found in 「5MUJ」
PDB 5MWB was passing test before but now failed with 「unknown」 error! Its run-log could be found in 「5MWB」
PDB 5NF7 was passing test before but now failed with 「unrecognized_residue」 error! Its run-log could be found in 「5NF7」
PDB 5NFA was passing test before but now failed with 「unrecognized_residue」 error! Its run-log could be found in 「5NFA」
PDB 5NFV was passing test before but now failed with 「unrecognized_residue」 error! Its run-log could be found in 「5NFV」
PDB 5NH7 was passing test before but now failed with 「fill_missing_atoms」 error! Its run-log could be found in 「5NH7」
PDB 5NH8 was passing test before but now failed with 「fill_missing_atoms」 error! Its run-log could be found in 「5NH8」
PDB 5NH9 was passing test before but now failed with 「unknown」 error! Its run-log could be found in 「5NH9」
PDB 5NHD was passing test before but now failed with 「unknown」 error! Its run-log could be found in 「5NHD」
PDB 5NHE was passing test before but now failed with 「fill_missing_atoms」 error! Its run-log could be found in 「5NHE」
PDB 5NJW was passing test before but now failed with 「fill_missing_atoms」 error! Its run-log could be found in 「5NJW」
PDB 5NKU was passing test before but now failed with 「fill_missing_atoms」 error! Its run-log could be found in 「5NKU」
PDB 5NLN was passing test before but now failed with 「unknown」 error! Its run-log could be found in 「5NLN」
PDB 5NLO was passing test before but now failed with 「unknown」 error! Its run-log could be found in 「5NLO」
PDB 5NLP was passing test before but now failed with 「unknown」 error! Its run-log could be found in 「5NLP」
PDB 5NLQ was passing test before but now failed with 「unknown」 error! Its run-log could be found in 「5NLQ」
PDB 5NN8 was passing test before but now failed with 「fill_missing_atoms」 error! Its run-log could be found in 「5NN8」
PDB 5O80 was passing test before but now failed with 「fill_missing_atoms」 error! Its run-log could be found in 「5O80」
PDB 5O82 was passing test before but now failed with 「unknown」 error! Its run-log could be found in 「5O82」
PDB 5OCA was passing test before but now failed with 「unrecognized_residue」 error! Its run-log could be found in 「5OCA」
PDB 5OCP was passing test before but now failed with 「unknown」 error! Its run-log could be found in 「5OCP」
PDB 5OCZ was passing test before but now failed with 「unrecognized_residue」 error! Its run-log could be found in 「5OCZ」
PDB 5ODF was passing test before but now failed with 「unrecognized_residue」 error! Its run-log could be found in 「5ODF」
PDB 5ODM was passing test before but now failed with 「unrecognized_residue」 error! Its run-log could be found in 「5ODM」
PDB 5OE1 was passing test before but now failed with 「unrecognized_residue」 error! Its run-log could be found in 「5OE1」
PDB 5OFK was passing test before but now failed with 「unknown」 error! Its run-log could be found in 「5OFK」
PDB 5OPJ was passing test before but now failed with 「fill_missing_atoms」 error! Its run-log could be found in 「5OPJ」
PDB 5TAR was passing test before but now failed with 「reroot_disconnected」 error! Its run-log could be found in 「5TAR」
PDB 5TB5 was passing test before but now failed with 「reroot_disconnected」 error! Its run-log could be found in 「5TB5」
PDB 5TIP was passing test before but now failed with 「fill_missing_atoms」 error! Its run-log could be found in 「5TIP」
PDB 5TIQ was passing test before but now failed with 「fill_missing_atoms」 error! Its run-log could be found in 「5TIQ」
PDB 5UBQ was passing test before but now failed with 「bad_patch」 error! Its run-log could be found in 「5UBQ」
PDB 5UCY was passing test before but now failed with 「bad_patch」 error! Its run-log could be found in 「5UCY」
PDB 5UF6 was passing test before but now failed with 「unrecognized_residue」 error! Its run-log could be found in 「5UF6」
PDB 5UHZ was passing test before but now failed with 「fill_missing_atoms」 error! Its run-log could be found in 「5UHZ」
PDB 5UKM was passing test before but now failed with 「reroot_disconnected」 error! Its run-log could be found in 「5UKM」
PDB 5UN3 was passing test before but now failed with 「unknown」 error! Its run-log could be found in 「5UN3」
PDB 5UQ6 was passing test before but now failed with 「fill_missing_atoms」 error! Its run-log could be found in 「5UQ6」
PDB 5UU8 was passing test before but now failed with 「unknown」 error! Its run-log could be found in 「5UU8」
PDB 5UU9 was passing test before but now failed with 「fill_missing_atoms」 error! Its run-log could be found in 「5UU9」
PDB 5UUA was passing test before but now failed with 「unknown」 error! Its run-log could be found in 「5UUA」
PDB 5UUB was passing test before but now failed with 「fill_missing_atoms」 error! Its run-log could be found in 「5UUB」
PDB 5VC7 was passing test before but now failed with 「bad_patch」 error! Its run-log could be found in 「5VC7」
PDB 5W26 was passing test before but now failed with 「fill_missing_atoms」 error! Its run-log could be found in 「5W26」
PDB 5W2U was passing test before but now failed with 「fill_missing_atoms」 error! Its run-log could be found in 「5W2U」
PDB 5W2W was passing test before but now failed with 「fill_missing_atoms」 error! Its run-log could be found in 「5W2W」
PDB 5W2Y was passing test before but now failed with 「fill_missing_atoms」 error! Its run-log could be found in 「5W2Y」
PDB 5WD7 was passing test before but now failed with 「unrecognized_residue」 error! Its run-log could be found in 「5WD7」
PDB 5WV1 was passing test before but now failed with 「unknown」 error! Its run-log could be found in 「5WV1」
PDB 5X7Q was passing test before but now failed with 「fill_missing_atoms」 error! Its run-log could be found in 「5X7Q」
PDB 5X7R was passing test before but now failed with 「fill_missing_atoms」 error! Its run-log could be found in 「5X7R」
PDB 5X8B was passing test before but now failed with 「nu_conformer」 error! Its run-log could be found in 「5X8B」
PDB 5XOO was passing test before but now failed with 「fill_missing_atoms」 error! Its run-log could be found in 「5XOO」

PDB 5XP6 was passing test before but now failed with 「fill_missing_atoms」 error! Its run-log could be found in 「5XP6」
PDB 5XSJ was passing test before but now failed with 「fill_missing_atoms」 error! Its run-log could be found in 「5XSJ」
PDB 5XSS was passing test before but now failed with 「unknown」 error! Its run-log could be found in 「5XSS」
PDB 5Y5R was passing test before but now failed with 「bad_patch」 error! Its run-log could be found in 「5Y5R」
PDB 5Z5F was passing test before but now failed with 「unknown」 error! Its run-log could be found in 「5Z5F」
PDB 5Z5I was passing test before but now failed with 「unknown」 error! Its run-log could be found in 「5Z5I」
PDB 5ZDQ was passing test before but now failed with 「fill_missing_atoms」 error! Its run-log could be found in 「5ZDQ」
PDB 5ZDR was passing test before but now failed with 「fill_missing_atoms」 error! Its run-log could be found in 「5ZDR」
PDB 5ZF3 was passing test before but now failed with 「unknown」 error! Its run-log could be found in 「5ZF3」
PDB 5ZGE was passing test before but now failed with 「fill_missing_atoms」 error! Its run-log could be found in 「5ZGE」
PDB 5ZGF was passing test before but now failed with 「fill_missing_atoms」 error! Its run-log could be found in 「5ZGF」
PDB 5ZGI was passing test before but now failed with 「fill_missing_atoms」 error! Its run-log could be found in 「5ZGI」
PDB 5ZGQ was passing test before but now failed with 「fill_missing_atoms」 error! Its run-log could be found in 「5ZGQ」
PDB 5ZGT was passing test before but now failed with 「fill_missing_atoms」 error! Its run-log could be found in 「5ZGT」
PDB 5ZGU was passing test before but now failed with 「fill_missing_atoms」 error! Its run-log could be found in 「5ZGU」
PDB 5ZGV was passing test before but now failed with 「fill_missing_atoms」 error! Its run-log could be found in 「5ZGV」
PDB 5ZGW was passing test before but now failed with 「fill_missing_atoms」 error! Its run-log could be found in 「5ZGW」
PDB 5ZGX was passing test before but now failed with 「fill_missing_atoms」 error! Its run-log could be found in 「5ZGX」
PDB 5ZGY was passing test before but now failed with 「fill_missing_atoms」 error! Its run-log could be found in 「5ZGY」
PDB 5ZGZ was passing test before but now failed with 「fill_missing_atoms」 error! Its run-log could be found in 「5ZGZ」
PDB 5ZH1 was passing test before but now failed with 「fill_missing_atoms」 error! Its run-log could be found in 「5ZH1」
PDB 5ZII was passing test before but now failed with 「unknown」 error! Its run-log could be found in 「5ZII」
PDB 5ZKZ was passing test before but now failed with 「unknown」 error! Its run-log could be found in 「5ZKZ」
PDB 5ZQS was passing test before but now failed with 「unknown」 error! Its run-log could be found in 「5ZQS」
PDB 5ZQX was passing test before but now failed with 「unknown」 error! Its run-log could be found in 「5ZQX」
PDB 6A89 was passing test before but now failed with 「unknown」 error! Its run-log could be found in 「6A89」
PDB 6A8H was passing test before but now failed with 「unknown」 error! Its run-log could be found in 「6A8H」
PDB 6A8I was passing test before but now failed with 「unknown」 error! Its run-log could be found in 「6A8I」
PDB 6ALS was passing test before but now failed with 「unrecognized_residue」 error! Its run-log could be found in 「6ALS」
PDB 6ALT was passing test before but now failed with 「unrecognized_residue」 error! Its run-log could be found in 「6ALT」
PDB 6ALU was passing test before but now failed with 「unrecognized_residue」 error! Its run-log could be found in 「6ALU」
PDB 6AQ5 was passing test before but now failed with 「fill_missing_atoms」 error! Its run-log could be found in 「6AQ5」
PDB 6AQ6 was passing test before but now failed with 「fill_missing_atoms」 error! Its run-log could be found in 「6AQ6」
PDB 6AVL was passing test before but now failed with 「fill_missing_atoms」 error! Its run-log could be found in 「6AVL」
PDB 6B6O was passing test before but now failed with 「fill_missing_atoms」 error! Its run-log could be found in 「6B6O」
PDB 6B6P was passing test before but now failed with 「fill_missing_atoms」 error! Its run-log could be found in 「6B6P」
PDB 6B6Q was passing test before but now failed with 「fill_missing_atoms」 error! Its run-log could be found in 「6B6Q」
PDB 6BHJ was passing test before but now failed with 「unrecognized_residue」 error! Its run-log could be found in 「6BHJ」
PDB 6CJV was passing test before but now failed with 「unrecognized_residue」 error! Its run-log could be found in 「6CJV」
PDB 6D5N was passing test before but now failed with 「unknown」 error! Its run-log could be found in 「6D5N」
PDB 6D5P was passing test before but now failed with 「unknown」 error! Its run-log could be found in 「6D5P」
PDB 6D5Q was passing test before but now failed with 「unknown」 error! Its run-log could be found in 「6D5Q」
PDB 6D5R was passing test before but now failed with 「unknown」 error! Its run-log could be found in 「6D5R」
PDB 6EGW was passing test before but now failed with 「unknown」 error! Its run-log could be found in 「6EGW」
PDB 6EQ1 was passing test before but now failed with 「unrecognized_residue」 error! Its run-log could be found in 「6EQ1」
PDB 6EUF was passing test before but now failed with 「fill_missing_atoms」 error! Its run-log could be found in 「6EUF」
PDB 6F1G was passing test before but now failed with 「unknown」 error! Its run-log could be found in 「6F1G」
PDB 6F4V was passing test before but now failed with 「unrecognized_residue」 error! Its run-log could be found in 「6F4V」
PDB 6F5K was passing test before but now failed with 「fill_missing_atoms」 error! Its run-log could be found in 「6F5K」
PDB 6G09 was passing test before but now failed with 「unknown」 error! Its run-log could be found in 「6G09」
PDB 6G0B was passing test before but now failed with 「unknown」 error! Its run-log could be found in 「6G0B」
PDB 6G0N was passing test before but now failed with 「unknown」 error! Its run-log could be found in 「6G0N」
PDB 6G48 was passing test before but now failed with 「fill_missing_atoms」 error! Its run-log could be found in 「6G48」
PDB 6GCC was passing test before but now failed with 「unrecognized_residue」 error! Its run-log could be found in 「6GCC」
PDB 6HA8 was passing test before but now failed with 「bad_patch」 error! Its run-log could be found in 「6HA8」
PDB 6HBM was passing test before but now failed with 「unknown」 error! Its run-log could be found in 「6HBM」
PDB 6I9Y was passing test before but now failed with 「fill_missing_atoms」 error! Its run-log could be found in 「6I9Y」
PDB 6IDN was passing test before but now failed with 「fill_missing_atoms」 error! Its run-log could be found in 「6IDN」
PDB 6IG0 was passing test before but now failed with 「bad_patch」 error! Its run-log could be found in 「6IG0」
PDB 6ILD was passing test before but now failed with 「unknown」 error! Its run-log could be found in 「6ILD」
PDB 6ILS was passing test before but now failed with 「fill_missing_atoms」 error! Its run-log could be found in 「6ILS」
PDB 6M9C was passing test before but now failed with 「bad_patch」 error! Its run-log could be found in 「6M9C」
PDB 6N30 was passing test before but now failed with 「bad_patch」 error! Its run-log could be found in 「6N30」
PDB 6NKN was passing test before but now failed with 「fill_missing_atoms」 error! Its run-log could be found in 「6NKN」
PDB 7AT1 was passing test before but now failed with 「fill_missing_atoms」 error! Its run-log could be found in 「7AT1」
PDB 8AT1 was passing test before but now failed with 「fill_missing_atoms」 error! Its run-log could be found in 「8AT1」
PDB 8CGT was passing test before but now failed with 「unrecognized_residue」 error! Its run-log could be found in 「8CGT」
PDB 8MHT was passing test before but now failed with 「unrecognized_residue」 error! Its run-log could be found in 「8MHT」
PDB 9CGT was passing test before but now failed with 「unrecognized_residue」 error! Its run-log could be found in 「9CGT」

NOTE: 21873 PDB's passed the tests but was not listed in reference results.

To update reference results please copy the files below into the main repository:
reference-results.cif.new.json → 「main repository」 as tests/benchmark/tests/scientific/protein_data_bank_diagnostic/reference-results.cif.json
blocklist.cif.new.json → 「main repository」 as tests/benchmark/tests/scientific/protein_data_bank_diagnostic/blocklist.cif.json

REVISION

revision:61600

test_id: 689963

status: passed

Scientific test: Protein Data Bank diagnostic

AUTHOR AND DATE

Steven M Lewis, smlewi@gmail.com, Cyrus Biotechnology, Oct 2018

Sergey Lyskov, sergey.lyskov@gmail.com, Gray lab, Johns Hopkins University, Oct 2018

PURPOSE OF THE TEST

The PDB diagnostic was run on **173,664** PDB files. **166,316** files passed the test and **7,348** failed! This test tries to load every PDB file in the PDB database and classifies the failures that occur. The command line below shows what was done; broadly all versions of this test examine load-time problems and more expensive versions (`-PDB_diagnostic::skip_pack_and_min false`) also check for errors during scoring, packing, and minimization.

"Hunting down these bugs is the most fun thing you can do on a Thursday morning" - Andy Watkins, probably.

An individual PDB passes or fails this test based on whether it errors out or completes the diagnostic. The test as a whole passes or fails based on a "reference results" system, like an expected result in a unit test. About 700 PDBs and 1200 CIFs fail at the time of this writing; the purpose of the test is to document the failures and watch for new ones, so PDBs failing in an expected manner does not constitute an overall test failure. The test will fail if PDBs pass or fail **UNEXPECTEDLY**, where the expectation is defined by the reference results (see below).

If you find that this page is telling you the test failed because there are **FEWER** errors: **GREAT**! You fixed some bugs! You can update the reference results following the instructions at the bottom of the page.

If you find that there are **MORE** failures than expected, especially non-timeout failures, consider it a warning that recent code changes may have introduced bugs into the PDB reading machinery. If you don't know what's going on: post to Slack or devel. **DO NOT** just update the reference results in this case.

If you want to know more about what this test does - pester Steven Lewis to write proper documentation.

Command line used: `/home/benchmark/benchmark/W.jazz.hpc-a/rosetta.Jazz.hpc-a/_commits_/main/source/test/timelimit.py 32 /home/benchmark/benchmark/W.jazz.hpc-a/rosetta.Jazz.hpc-a/_commits_/main/source/bin/PDB_diagnostic.default.linuxclangrelease -no_color -out:file:score_only /dev/null -jd2::delete_old_poses true -ignore_unrecognized_res false -load_PDB_components true -packing::pack_missing_sidechains false -packing::repack_only true -s {input_file} -ignore_zero_occupancy false -in:file:obey_ENDMDL true -PDB_diagnostic::skip_pack_and_min false -PDB_diagnostic::reading_only false`

7,348 total PDBs failed with the following error codes:

```
4,579 「unrecognized_residue」
  457 「fill_missing_atoms」
  416 「zero_length_xyzVector」
  306 「unknown」
  271 「missing_disulfide_partner」
  208 「rotlib_file」
  203 「missing_bond」
  185 「bad_patch」
  138 「pseudobond_connection_change」
  100 「multiple_disulfides」
   92 「no_orient_atoms」
   91 「duplicate_atom_name」
   72 「aa_difference」
   67 「prepro_cyclic_pep」
   51 「unknown_atom_name」
   31 「reroot_disconnected」
   23 「zero_atom_restype」
   13 「alias_missing_atom」
   11 「nu_conformer」
   10 「unknown_hbond_acceptor」
    6 「exceed_timeout」
    5 「no_usable_coords」
    4 「base_of_chi」
    4 「merge_with_next」
    4 「insufficient_mainchain」
    1 「no_hbond_deriv」
```

Test marked as **FAILED** due to following errors:

```
PDB 1AC3 was passing test before but now failed with 「bad_patch」 error! Its run-log could be found in 「1AC3」
PDB 1AFC was passing test before but now failed with 「unrecognized_residue」 error! Its run-log could be found in 「1AFC」
```

PDB 1APO was passing test before but now failed with ⌈fill_missing_atoms⌋ error! Its run-log could be found in ⌈1APO⌋
PDB 1ASO was passing test before but now failed with ⌈fill_missing_atoms⌋ error! Its run-log could be found in ⌈1ASO⌋
PDB 1ASP was passing test before but now failed with ⌈fill_missing_atoms⌋ error! Its run-log could be found in ⌈1ASP⌋
PDB 1AX0 was passing test before but now failed with ⌈fill_missing_atoms⌋ error! Its run-log could be found in ⌈1AX0⌋
PDB 1AX1 was passing test before but now failed with ⌈fill_missing_atoms⌋ error! Its run-log could be found in ⌈1AX1⌋
PDB 1AX2 was passing test before but now failed with ⌈fill_missing_atoms⌋ error! Its run-log could be found in ⌈1AX2⌋
PDB 1AXY was passing test before but now failed with ⌈fill_missing_atoms⌋ error! Its run-log could be found in ⌈1AXY⌋
PDB 1AXZ was passing test before but now failed with ⌈fill_missing_atoms⌋ error! Its run-log could be found in ⌈1AXZ⌋
PDB 1AZX was passing test before but now failed with ⌈unrecognized_residue⌋ error! Its run-log could be found in ⌈1AZX⌋
PDB 1B30 was passing test before but now failed with ⌈unknown⌋ error! Its run-log could be found in ⌈1B30⌋
PDB 1B3V was passing test before but now failed with ⌈unknown⌋ error! Its run-log could be found in ⌈1B3V⌋
PDB 1B3W was passing test before but now failed with ⌈fill_missing_atoms⌋ error! Its run-log could be found in ⌈1B3W⌋
PDB 1B3X was passing test before but now failed with ⌈unknown⌋ error! Its run-log could be found in ⌈1B3X⌋
PDB 1B3Y was passing test before but now failed with ⌈unknown⌋ error! Its run-log could be found in ⌈1B3Y⌋
PDB 1B3Z was passing test before but now failed with ⌈unknown⌋ error! Its run-log could be found in ⌈1B3Z⌋
PDB 1BCX was passing test before but now failed with ⌈unknown⌋ error! Its run-log could be found in ⌈1BCX⌋
PDB 1BIQ was passing test before but now failed with ⌈fill_missing_atoms⌋ error! Its run-log could be found in ⌈1BIQ⌋
PDB 1BL9 was passing test before but now failed with ⌈fill_missing_atoms⌋ error! Its run-log could be found in ⌈1BL9⌋
PDB 1BVV was passing test before but now failed with ⌈unknown⌋ error! Its run-log could be found in ⌈1BVV⌋
PDB 1BYD was passing test before but now failed with ⌈unrecognized_residue⌋ error! Its run-log could be found in ⌈1BYD⌋
PDB 1C23 was passing test before but now failed with ⌈bad_patch⌋ error! Its run-log could be found in ⌈1C23⌋
PDB 1C5I was passing test before but now failed with ⌈unknown⌋ error! Its run-log could be found in ⌈1C5I⌋
PDB 1CB8 was passing test before but now failed with ⌈fill_missing_atoms⌋ error! Its run-log could be found in ⌈1CB8⌋
PDB 1CF5 was passing test before but now failed with ⌈fill_missing_atoms⌋ error! Its run-log could be found in ⌈1CF5⌋
PDB 1CPX was passing test before but now failed with ⌈fill_missing_atoms⌋ error! Its run-log could be found in ⌈1CPX⌋
PDB 1DBG was passing test before but now failed with ⌈fill_missing_atoms⌋ error! Its run-log could be found in ⌈1DBG⌋
PDB 1DBO was passing test before but now failed with ⌈fill_missing_atoms⌋ error! Its run-log could be found in ⌈1DBO⌋
PDB 1DOA was passing test before but now failed with ⌈reroot_disconnected⌋ error! Its run-log could be found in ⌈1DOA⌋
PDB 1DWA was passing test before but now failed with ⌈fill_missing_atoms⌋ error! Its run-log could be found in ⌈1DWA⌋
PDB 1DWF was passing test before but now failed with ⌈fill_missing_atoms⌋ error! Its run-log could be found in ⌈1DWF⌋
PDB 1DWG was passing test before but now failed with ⌈fill_missing_atoms⌋ error! Its run-log could be found in ⌈1DWG⌋
PDB 1DWH was passing test before but now failed with ⌈fill_missing_atoms⌋ error! Its run-log could be found in ⌈1DWH⌋
PDB 1DWI was passing test before but now failed with ⌈fill_missing_atoms⌋ error! Its run-log could be found in ⌈1DWI⌋
PDB 1DWJ was passing test before but now failed with ⌈fill_missing_atoms⌋ error! Its run-log could be found in ⌈1DWJ⌋
PDB 1E03 was passing test before but now failed with ⌈unrecognized_residue⌋ error! Its run-log could be found in ⌈1E03⌋
PDB 1E0X was passing test before but now failed with ⌈unknown⌋ error! Its run-log could be found in ⌈1E0X⌋
PDB 1E4M was passing test before but now failed with ⌈fill_missing_atoms⌋ error! Its run-log could be found in ⌈1E4M⌋
PDB 1E5N was passing test before but now failed with ⌈unknown⌋ error! Its run-log could be found in ⌈1E5N⌋
PDB 1E6Q was passing test before but now failed with ⌈fill_missing_atoms⌋ error! Its run-log could be found in ⌈1E6Q⌋
PDB 1E6S was passing test before but now failed with ⌈fill_missing_atoms⌋ error! Its run-log could be found in ⌈1E6S⌋
PDB 1E6X was passing test before but now failed with ⌈fill_missing_atoms⌋ error! Its run-log could be found in ⌈1E6X⌋
PDB 1E70 was passing test before but now failed with ⌈fill_missing_atoms⌋ error! Its run-log could be found in ⌈1E70⌋
PDB 1E71 was passing test before but now failed with ⌈fill_missing_atoms⌋ error! Its run-log could be found in ⌈1E71⌋
PDB 1E72 was passing test before but now failed with ⌈fill_missing_atoms⌋ error! Its run-log could be found in ⌈1E72⌋
PDB 1E73 was passing test before but now failed with ⌈fill_missing_atoms⌋ error! Its run-log could be found in ⌈1E73⌋
PDB 1E8X was passing test before but now failed with ⌈nu_conformer⌋ error! Its run-log could be found in ⌈1E8X⌋
PDB 1E98 was passing test before but now failed with ⌈duplicate_atom_name⌋ error! Its run-log could be found in ⌈1E98⌋
PDB 1E99 was passing test before but now failed with ⌈duplicate_atom_name⌋ error! Its run-log could be found in ⌈1E99⌋
PDB 1E9B was passing test before but now failed with ⌈duplicate_atom_name⌋ error! Its run-log could be found in ⌈1E9B⌋
PDB 1E9D was passing test before but now failed with ⌈duplicate_atom_name⌋ error! Its run-log could be found in ⌈1E9D⌋
PDB 1EIX was passing test before but now failed with ⌈alias_missing_atom⌋ error! Its run-log could be found in ⌈1EIX⌋
PDB 1EQ8 was passing test before but now failed with ⌈fill_missing_atoms⌋ error! Its run-log could be found in ⌈1EQ8⌋
PDB 1F4Y was passing test before but now failed with ⌈unrecognized_residue⌋ error! Its run-log could be found in ⌈1F4Y⌋
PDB 1F9O was passing test before but now failed with ⌈unrecognized_residue⌋ error! Its run-log could be found in ⌈1F9O⌋
PDB 1FA2 was passing test before but now failed with ⌈unrecognized_residue⌋ error! Its run-log could be found in ⌈1FA2⌋
PDB 1FGG was passing test before but now failed with ⌈bad_patch⌋ error! Its run-log could be found in ⌈1FGG⌋
PDB 1FH7 was passing test before but now failed with ⌈fill_missing_atoms⌋ error! Its run-log could be found in ⌈1FH7⌋
PDB 1FH8 was passing test before but now failed with ⌈fill_missing_atoms⌋ error! Its run-log could be found in ⌈1FH8⌋
PDB 1FH9 was passing test before but now failed with ⌈unknown⌋ error! Its run-log could be found in ⌈1FH9⌋
PDB 1FHD was passing test before but now failed with ⌈fill_missing_atoms⌋ error! Its run-log could be found in ⌈1FHD⌋
PDB 1FQ7 was passing test before but now failed with ⌈fill_missing_atoms⌋ error! Its run-log could be found in ⌈1FQ7⌋
PDB 1FTK was passing test before but now failed with ⌈unknown⌋ error! Its run-log could be found in ⌈1FTK⌋
PDB 1FW0 was passing test before but now failed with ⌈unknown⌋ error! Its run-log could be found in ⌈1FW0⌋
PDB 1GA8 was passing test before but now failed with ⌈unrecognized_residue⌋ error! Its run-log could be found in ⌈1GA8⌋
PDB 1GAI was passing test before but now failed with ⌈unrecognized_residue⌋ error! Its run-log could be found in ⌈1GAI⌋
PDB 1GJN was passing test before but now failed with ⌈fill_missing_atoms⌋ error! Its run-log could be found in ⌈1GJN⌋
PDB 1GNY was passing test before but now failed with ⌈unknown⌋ error! Its run-log could be found in ⌈1GNY⌋
PDB 1GOQ was passing test before but now failed with ⌈unknown⌋ error! Its run-log could be found in ⌈1GOQ⌋
PDB 1GOR was passing test before but now failed with ⌈unknown⌋ error! Its run-log could be found in ⌈1GOR⌋
PDB 1GQJ was passing test before but now failed with ⌈unknown⌋ error! Its run-log could be found in ⌈1GQJ⌋
PDB 1GQL was passing test before but now failed with ⌈unknown⌋ error! Its run-log could be found in ⌈1GQL⌋
PDB 1GR2 was passing test before but now failed with ⌈unknown⌋ error! Its run-log could be found in ⌈1GR2⌋
PDB 1GYE was passing test before but now failed with ⌈unknown⌋ error! Its run-log could be found in ⌈1GYE⌋
PDB 1H12 was passing test before but now failed with ⌈fill_missing_atoms⌋ error! Its run-log could be found in ⌈1H12⌋
PDB 1H3F was passing test before but now failed with ⌈unknown_atom_name⌋ error! Its run-log could be found in ⌈1H3F⌋
PDB 1H4G was passing test before but now failed with ⌈unknown⌋ error! Its run-log could be found in ⌈1H4G⌋
PDB 1H4H was passing test before but now failed with ⌈unknown⌋ error! Its run-log could be found in ⌈1H4H⌋
PDB 1HM2 was passing test before but now failed with ⌈fill_missing_atoms⌋ error! Its run-log could be found in ⌈1HM2⌋
PDB 1HM3 was passing test before but now failed with ⌈fill_missing_atoms⌋ error! Its run-log could be found in ⌈1HM3⌋
PDB 1HMU was passing test before but now failed with ⌈fill_missing_atoms⌋ error! Its run-log could be found in ⌈1HMU⌋
PDB 1HMW was passing test before but now failed with ⌈fill_missing_atoms⌋ error! Its run-log could be found in ⌈1HMW⌋

PDB 1IOO was passing test before but now failed with ⌜fill_missing_atoms⌟ error! Its run-log could be found in ⌜1IOO⌟
PDB 1IQQ was passing test before but now failed with ⌜fill_missing_atoms⌟ error! Its run-log could be found in ⌜1IQQ⌟
PDB 1ISV was passing test before but now failed with ⌜unknown⌟ error! Its run-log could be found in ⌜1ISV⌟
PDB 1ISW was passing test before but now failed with ⌜unknown⌟ error! Its run-log could be found in ⌜1ISW⌟
PDB 1ISX was passing test before but now failed with ⌜unknown⌟ error! Its run-log could be found in ⌜1ISX⌟
PDB 1IZ2 was passing test before but now failed with ⌜unrecognized_residue⌟ error! Its run-log could be found in ⌜1IZ2⌟
PDB 1J10 was passing test before but now failed with ⌜fill_missing_atoms⌟ error! Its run-log could be found in ⌜1J10⌟
PDB 1J90 was passing test before but now failed with ⌜unrecognized_residue⌟ error! Its run-log could be found in ⌜1J90⌟
PDB 1JGU was passing test before but now failed with ⌜fill_missing_atoms⌟ error! Its run-log could be found in ⌜1JGU⌟
PDB 1JKU was passing test before but now failed with ⌜fill_missing_atoms⌟ error! Its run-log could be found in ⌜1JKU⌟
PDB 1K1H was passing test before but now failed with ⌜unknown_atom_name⌟ error! Its run-log could be found in ⌜1K1H⌟
PDB 1K9F was passing test before but now failed with ⌜fill_missing_atoms⌟ error! Its run-log could be found in ⌜1K9F⌟
PDB 1KO3 was passing test before but now failed with ⌜fill_missing_atoms⌟ error! Its run-log could be found in ⌜1KO3⌟
PDB 1KTG was passing test before but now failed with ⌜fill_missing_atoms⌟ error! Its run-log could be found in ⌜1KTG⌟
PDB 1L8N was passing test before but now failed with ⌜unknown⌟ error! Its run-log could be found in ⌜1L8N⌟
PDB 1LBB was passing test before but now failed with ⌜unknown⌟ error! Its run-log could be found in ⌜1LBB⌟
PDB 1LLP was passing test before but now failed with ⌜fill_missing_atoms⌟ error! Its run-log could be found in ⌜1LLP⌟
PDB 1MBN was passing test before but now failed with ⌜fill_missing_atoms⌟ error! Its run-log could be found in ⌜1MBN⌟
PDB 1MC9 was passing test before but now failed with ⌜unknown⌟ error! Its run-log could be found in ⌜1MC9⌟
PDB 1MMM was passing test before but now failed with ⌜fill_missing_atoms⌟ error! Its run-log could be found in ⌜1MMM⌟
PDB 1MMS was passing test before but now failed with ⌜fill_missing_atoms⌟ error! Its run-log could be found in ⌜1MMS⌟
PDB 1MUW was passing test before but now failed with ⌜fill_missing_atoms⌟ error! Its run-log could be found in ⌜1MUW⌟
PDB 1MXD was passing test before but now failed with ⌜fill_missing_atoms⌟ error! Its run-log could be found in ⌜1MXD⌟
PDB 1MYR was passing test before but now failed with ⌜fill_missing_atoms⌟ error! Its run-log could be found in ⌜1MYR⌟
PDB 1N9F was passing test before but now failed with ⌜fill_missing_atoms⌟ error! Its run-log could be found in ⌜1N9F⌟
PDB 1N9H was passing test before but now failed with ⌜fill_missing_atoms⌟ error! Its run-log could be found in ⌜1N9H⌟
PDB 1N9I was passing test before but now failed with ⌜fill_missing_atoms⌟ error! Its run-log could be found in ⌜1N9I⌟
PDB 1N9X was passing test before but now failed with ⌜fill_missing_atoms⌟ error! Its run-log could be found in ⌜1N9X⌟
PDB 1NAE was passing test before but now failed with ⌜unknown⌟ error! Its run-log could be found in ⌜1NAE⌟
PDB 1NAZ was passing test before but now failed with ⌜fill_missing_atoms⌟ error! Its run-log could be found in ⌜1NAZ⌟
PDB 1NIR was passing test before but now failed with ⌜fill_missing_atoms⌟ error! Its run-log could be found in ⌜1NIR⌟
PDB 1NQ9 was passing test before but now failed with ⌜unrecognized_residue⌟ error! Its run-log could be found in ⌜1NQ9⌟
PDB 1NZ1 was passing test before but now failed with ⌜unknown⌟ error! Its run-log could be found in ⌜1NZ1⌟
PDB 1OD8 was passing test before but now failed with ⌜fill_missing_atoms⌟ error! Its run-log could be found in ⌜1OD8⌟
PDB 1OFL was passing test before but now failed with ⌜fill_missing_atoms⌟ error! Its run-log could be found in ⌜1OFL⌟
PDB 1OFM was passing test before but now failed with ⌜fill_missing_atoms⌟ error! Its run-log could be found in ⌜1OFM⌟
PDB 1OMW was passing test before but now failed with ⌜reroot_disconnected⌟ error! Its run-log could be found in ⌜1OMW⌟
PDB 1P1N was passing test before but now failed with ⌜unknown⌟ error! Its run-log could be found in ⌜1P1N⌟
PDB 1P60 was passing test before but now failed with ⌜unrecognized_residue⌟ error! Its run-log could be found in ⌜1P60⌟
PDB 1P61 was passing test before but now failed with ⌜unrecognized_residue⌟ error! Its run-log could be found in ⌜1P61⌟
PDB 1P7P was passing test before but now failed with ⌜bad_patch⌟ error! Its run-log could be found in ⌜1P7P⌟
PDB 1PAJ was passing test before but now failed with ⌜fill_missing_atoms⌟ error! Its run-log could be found in ⌜1PAJ⌟
PDB 1PAK was passing test before but now failed with ⌜fill_missing_atoms⌟ error! Its run-log could be found in ⌜1PAK⌟
PDB 1PCN was passing test before but now failed with ⌜fill_missing_atoms⌟ error! Its run-log could be found in ⌜1PCN⌟
PDB 1PCO was passing test before but now failed with ⌜fill_missing_atoms⌟ error! Its run-log could be found in ⌜1PCO⌟
PDB 1PX8 was passing test before but now failed with ⌜unknown⌟ error! Its run-log could be found in ⌜1PX8⌟
PDB 1PYY was passing test before but now failed with ⌜unrecognized_residue⌟ error! Its run-log could be found in ⌜1PYY⌟
PDB 1PZ2 was passing test before but now failed with ⌜unknown⌟ error! Its run-log could be found in ⌜1PZ2⌟
PDB 1QD3 was passing test before but now failed with ⌜fill_missing_atoms⌟ error! Its run-log could be found in ⌜1QD3⌟
PDB 1QH6 was passing test before but now failed with ⌜unknown⌟ error! Its run-log could be found in ⌜1QH6⌟
PDB 1QH7 was passing test before but now failed with ⌜unknown⌟ error! Its run-log could be found in ⌜1QH7⌟
PDB 1QK0 was passing test before but now failed with ⌜fill_missing_atoms⌟ error! Its run-log could be found in ⌜1QK0⌟
PDB 1QW8 was passing test before but now failed with ⌜unknown⌟ error! Its run-log could be found in ⌜1QW8⌟
PDB 1R0B was passing test before but now failed with ⌜multiple_disulfides⌟ error! Its run-log could be found in ⌜1R0B⌟
PDB 1R87 was passing test before but now failed with ⌜unknown⌟ error! Its run-log could be found in ⌜1R87⌟
PDB 1R9S was passing test before but now failed with ⌜bad_patch⌟ error! Its run-log could be found in ⌜1R9S⌟
PDB 1RK2 was passing test before but now failed with ⌜fill_missing_atoms⌟ error! Its run-log could be found in ⌜1RK2⌟
PDB 1RKD was passing test before but now failed with ⌜fill_missing_atoms⌟ error! Its run-log could be found in ⌜1RKD⌟
PDB 1RKS was passing test before but now failed with ⌜fill_missing_atoms⌟ error! Its run-log could be found in ⌜1RKS⌟
PDB 1RMY was passing test before but now failed with ⌜unrecognized_residue⌟ error! Its run-log could be found in ⌜1RMY⌟
PDB 1S4G was passing test before but now failed with ⌜fill_missing_atoms⌟ error! Its run-log could be found in ⌜1S4G⌟
PDB 1S5N was passing test before but now failed with ⌜fill_missing_atoms⌟ error! Its run-log could be found in ⌜1S5N⌟
PDB 1SR5 was passing test before but now failed with ⌜unrecognized_residue⌟ error! Its run-log could be found in ⌜1SR5⌟
PDB 1STO was passing test before but now failed with ⌜fill_missing_atoms⌟ error! Its run-log could be found in ⌜1STO⌟
PDB 1T0Q was passing test before but now failed with ⌜fill_missing_atoms⌟ error! Its run-log could be found in ⌜1T0Q⌟
PDB 1T0S was passing test before but now failed with ⌜fill_missing_atoms⌟ error! Its run-log could be found in ⌜1T0S⌟
PDB 1T3I was passing test before but now failed with ⌜unrecognized_residue⌟ error! Its run-log could be found in ⌜1T3I⌟
PDB 1TEQ was passing test before but now failed with ⌜fill_missing_atoms⌟ error! Its run-log could be found in ⌜1TEQ⌟
PDB 1TT1 was passing test before but now failed with ⌜unknown⌟ error! Its run-log could be found in ⌜1TT1⌟
PDB 1UQY was passing test before but now failed with ⌜unknown⌟ error! Its run-log could be found in ⌜1UQY⌟
PDB 1UQZ was passing test before but now failed with ⌜unknown⌟ error! Its run-log could be found in ⌜1UQZ⌟
PDB 1UR1 was passing test before but now failed with ⌜unknown⌟ error! Its run-log could be found in ⌜1UR1⌟
PDB 1UR2 was passing test before but now failed with ⌜unknown⌟ error! Its run-log could be found in ⌜1UR2⌟
PDB 1US2 was passing test before but now failed with ⌜unknown⌟ error! Its run-log could be found in ⌜1US2⌟
PDB 1UX7 was passing test before but now failed with ⌜unknown⌟ error! Its run-log could be found in ⌜1UX7⌟
PDB 1UXX was passing test before but now failed with ⌜unknown⌟ error! Its run-log could be found in ⌜1UXX⌟
PDB 1UY2 was passing test before but now failed with ⌜unknown⌟ error! Its run-log could be found in ⌜1UY2⌟
PDB 1UY3 was passing test before but now failed with ⌜unknown⌟ error! Its run-log could be found in ⌜1UY3⌟
PDB 1UY4 was passing test before but now failed with ⌜unknown⌟ error! Its run-log could be found in ⌜1UY4⌟
PDB 1UYZ was passing test before but now failed with ⌜unknown⌟ error! Its run-log could be found in ⌜1UYZ⌟
PDB 1UZY was passing test before but now failed with ⌜fill_missing_atoms⌟ error! Its run-log could be found in ⌜1UZY⌟

PDB 1V0K was passing test before but now failed with 「fill_missing_atoms」 error! Its run-log could be found in 「1V0K」
PDB 1V0L was passing test before but now failed with 「unknown」 error! Its run-log could be found in 「1V0L」
PDB 1V0M was passing test before but now failed with 「fill_missing_atoms」 error! Its run-log could be found in 「1V0M」
PDB 1V0N was passing test before but now failed with 「fill_missing_atoms」 error! Its run-log could be found in 「1V0N」
PDB 1V6U was passing test before but now failed with 「unknown」 error! Its run-log could be found in 「1V6U」
PDB 1V6V was passing test before but now failed with 「unknown」 error! Its run-log could be found in 「1V6V」
PDB 1V6W was passing test before but now failed with 「unknown」 error! Its run-log could be found in 「1V6W」
PDB 1V6X was passing test before but now failed with 「unknown」 error! Its run-log could be found in 「1V6X」
PDB 1VBR was passing test before but now failed with 「fill_missing_atoms」 error! Its run-log could be found in 「1VBR」
PDB 1VEW was passing test before but now failed with 「fill_missing_atoms」 error! Its run-log could be found in 「1VEW」
PDB 1W2H was passing test before but now failed with 「duplicate_atom_name」 error! Its run-log could be found in 「1W2H」
PDB 1W9B was passing test before but now failed with 「fill_missing_atoms」 error! Its run-log could be found in 「1W9B」
PDB 1W9D was passing test before but now failed with 「fill_missing_atoms」 error! Its run-log could be found in 「1W9D」
PDB 1W9T was passing test before but now failed with 「unknown」 error! Its run-log could be found in 「1W9T」
PDB 1WD4 was passing test before but now failed with 「fill_missing_atoms」 error! Its run-log could be found in 「1WD4」
PDB 1WU5 was passing test before but now failed with 「fill_missing_atoms」 error! Its run-log could be found in 「1WU5」
PDB 1WU6 was passing test before but now failed with 「unknown」 error! Its run-log could be found in 「1WU6」
PDB 1X38 was passing test before but now failed with 「fill_missing_atoms」 error! Its run-log could be found in 「1X38」
PDB 1X39 was passing test before but now failed with 「fill_missing_atoms」 error! Its run-log could be found in 「1X39」
PDB 1X9D was passing test before but now failed with 「unrecognized_residue」 error! Its run-log could be found in 「1X9D」
PDB 1XNK was passing test before but now failed with 「unrecognized_residue」 error! Its run-log could be found in 「1XNK」
PDB 1XU5 was passing test before but now failed with 「fill_missing_atoms」 error! Its run-log could be found in 「1XU5」
PDB 1XWQ was passing test before but now failed with 「unknown」 error! Its run-log could be found in 「1XWQ」
PDB 1XYA was passing test before but now failed with 「fill_missing_atoms」 error! Its run-log could be found in 「1XYA」
PDB 1XYL was passing test before but now failed with 「fill_missing_atoms」 error! Its run-log could be found in 「1XYL」
PDB 1XYM was passing test before but now failed with 「fill_missing_atoms」 error! Its run-log could be found in 「1XYM」
PDB 1YOE was passing test before but now failed with 「fill_missing_atoms」 error! Its run-log could be found in 「1YOE」
PDB 2A2Z was passing test before but now failed with 「unrecognized_residue」 error! Its run-log could be found in 「2A2Z」
PDB 2A30 was passing test before but now failed with 「unrecognized_residue」 error! Its run-log could be found in 「2A30」
PDB 2ANJ was passing test before but now failed with 「unknown」 error! Its run-log could be found in 「2ANJ」
PDB 2AT1 was passing test before but now failed with 「fill_missing_atoms」 error! Its run-log could be found in 「2AT1」
PDB 2B46 was passing test before but now failed with 「unknown」 error! Its run-log could be found in 「2B46」
PDB 2B4F was passing test before but now failed with 「unknown」 error! Its run-log could be found in 「2B4F」
PDB 2BMZ was passing test before but now failed with 「unknown」 error! Its run-log could be found in 「2BMZ」
PDB 2BNJ was passing test before but now failed with 「unknown」 error! Its run-log could be found in 「2BNJ」
PDB 2BTF was passing test before but now failed with 「bad_patch」 error! Its run-log could be found in 「2BTF」
PDB 2BUM was passing test before but now failed with 「fill_missing_atoms」 error! Its run-log could be found in 「2BUM」
PDB 2BUT was passing test before but now failed with 「fill_missing_atoms」 error! Its run-log could be found in 「2BUT」
PDB 2BUX was passing test before but now failed with 「fill_missing_atoms」 error! Its run-log could be found in 「2BUX」
PDB 2C40 was passing test before but now failed with 「unknown」 error! Its run-log could be found in 「2C40」
PDB 2C7F was passing test before but now failed with 「unknown」 error! Its run-log could be found in 「2C7F」
PDB 2C8N was passing test before but now failed with 「unknown」 error! Its run-log could be found in 「2C8N」
PDB 2CCH was passing test before but now failed with 「bad_patch」 error! Its run-log could be found in 「2CCH」
PDB 2CDC was passing test before but now failed with 「fill_missing_atoms」 error! Its run-log could be found in 「2CDC」
PDB 2CNC was passing test before but now failed with 「unknown」 error! Its run-log could be found in 「2CNC」
PDB 2D43 was passing test before but now failed with 「unknown」 error! Its run-log could be found in 「2D43」
PDB 2D44 was passing test before but now failed with 「unknown」 error! Its run-log could be found in 「2D44」
PDB 2E2Q was passing test before but now failed with 「fill_missing_atoms」 error! Its run-log could be found in 「2E2Q」
PDB 2F5V was passing test before but now failed with 「fill_missing_atoms」 error! Its run-log could be found in 「2F5V」
PDB 2FAF was passing test before but now failed with 「unrecognized_residue」 error! Its run-log could be found in 「2FAF」
PDB 2FAH was passing test before but now failed with 「unrecognized_residue」 error! Its run-log could be found in 「2FAH」
PDB 2GD4 was passing test before but now failed with 「unrecognized_residue」 error! Its run-log could be found in 「2GD4」
PDB 2GSM was passing test before but now failed with 「fill_missing_atoms」 error! Its run-log could be found in 「2GSM」
PDB 2HMP was passing test before but now failed with 「bad_patch」 error! Its run-log could be found in 「2HMP」
PDB 2HOX was passing test before but now failed with 「fill_missing_atoms」 error! Its run-log could be found in 「2HOX」
PDB 2HUO was passing test before but now failed with 「fill_missing_atoms」 error! Its run-log could be found in 「2HUO」
PDB 2IMH was passing test before but now failed with 「bad_patch」 error! Its run-log could be found in 「2IMH」
PDB 2IX8 was passing test before but now failed with 「bad_patch」 error! Its run-log could be found in 「2IX8」
PDB 2JIX was passing test before but now failed with 「missing_bond」 error! Its run-log could be found in 「2JIX」
PDB 2KQD was passing test before but now failed with 「unknown」 error! Its run-log could be found in 「2KQD」
PDB 2KQE was passing test before but now failed with 「unknown」 error! Its run-log could be found in 「2KQE」
PDB 2NC5 was passing test before but now failed with 「unknown」 error! Its run-log could be found in 「2NC5」
PDB 2NO1 was passing test before but now failed with 「unrecognized_residue」 error! Its run-log could be found in 「2NO1」
PDB 2NQY was passing test before but now failed with 「unknown」 error! Its run-log could be found in 「2NQY」
PDB 2NX3 was passing test before but now failed with 「unknown」 error! Its run-log could be found in 「2NX3」
PDB 2NXG was passing test before but now failed with 「unknown」 error! Its run-log could be found in 「2NXG」
PDB 2OXJ was passing test before but now failed with 「aa_difference」 error! Its run-log could be found in 「2OXJ」
PDB 2OXK was passing test before but now failed with 「aa_difference」 error! Its run-log could be found in 「2OXK」
PDB 2P10 was passing test before but now failed with 「bad_patch」 error! Its run-log could be found in 「2P10」
PDB 2P1S was passing test before but now failed with 「unrecognized_residue」 error! Its run-log could be found in 「2P1S」
PDB 2P37 was passing test before but now failed with 「unrecognized_residue」 error! Its run-log could be found in 「2P37」
PDB 2P39 was passing test before but now failed with 「unrecognized_residue」 error! Its run-log could be found in 「2P39」
PDB 2QJE was passing test before but now failed with 「unrecognized_residue」 error! Its run-log could be found in 「2QJE」
PDB 2QKH was passing test before but now failed with 「unrecognized_residue」 error! Its run-log could be found in 「2QKH」
PDB 2QOB was passing test before but now failed with 「missing_disulfide_partner」 error! Its run-log could be found in 「2QOB」
PDB 2QZ2 was passing test before but now failed with 「unknown」 error! Its run-log could be found in 「2QZ2」
PDB 2QZ3 was passing test before but now failed with 「unknown」 error! Its run-log could be found in 「2QZ3」
PDB 2QZY was passing test before but now failed with 「unrecognized_residue」 error! Its run-log could be found in 「2QZY」
PDB 2TMK was passing test before but now failed with 「duplicate_atom_name」 error! Its run-log could be found in 「2TMK」
PDB 2UUS was passing test before but now failed with 「unrecognized_residue」 error! Its run-log could be found in 「2UUS」

PDB 2UWN was passing test before but now failed with ⌈unrecognized_residue⌋ error! Its run-log could be found in ⌈2UWN⌋
PDB 2V1E was passing test before but now failed with ⌈fill_missing_atoms⌋ error! Its run-log could be found in ⌈2V1E⌋
PDB 2V1F was passing test before but now failed with ⌈fill_missing_atoms⌋ error! Its run-log could be found in ⌈2V1F⌋
PDB 2V1G was passing test before but now failed with ⌈fill_missing_atoms⌋ error! Its run-log could be found in ⌈2V1G⌋
PDB 2V4V was passing test before but now failed with ⌈fill_missing_atoms⌋ error! Its run-log could be found in ⌈2V4V⌋
PDB 2V5E was passing test before but now failed with ⌈unrecognized_residue⌋ error! Its run-log could be found in ⌈2V5E⌋
PDB 2V8E was passing test before but now failed with ⌈unrecognized_residue⌋ error! Its run-log could be found in ⌈2V8E⌋
PDB 2V92 was passing test before but now failed with ⌈nu_conformer⌋ error! Its run-log could be found in ⌈2V92⌋
PDB 2VGD was passing test before but now failed with ⌈unknown⌋ error! Its run-log could be found in ⌈2VGD⌋
PDB 2VLC was passing test before but now failed with ⌈unrecognized_residue⌋ error! Its run-log could be found in ⌈2VLC⌋
PDB 2VM0 was passing test before but now failed with ⌈fill_missing_atoms⌋ error! Its run-log could be found in ⌈2VM0⌋
PDB 2VP5 was passing test before but now failed with ⌈unrecognized_residue⌋ error! Its run-log could be found in ⌈2VP5⌋
PDB 2VRQ was passing test before but now failed with ⌈unknown⌋ error! Its run-log could be found in ⌈2VRQ⌋
PDB 2W5F was passing test before but now failed with ⌈unknown⌋ error! Its run-log could be found in ⌈2W5F⌋
PDB 2W5O was passing test before but now failed with ⌈unknown⌋ error! Its run-log could be found in ⌈2W5O⌋
PDB 2WW1 was passing test before but now failed with ⌈unrecognized_residue⌋ error! Its run-log could be found in ⌈2WW1⌋
PDB 2WW3 was passing test before but now failed with ⌈unrecognized_residue⌋ error! Its run-log could be found in ⌈2WW3⌋
PDB 2WWI was passing test before but now failed with ⌈duplicate_atom_name⌋ error! Its run-log could be found in ⌈2WWI⌋
PDB 2WXD was passing test before but now failed with ⌈fill_missing_atoms⌋ error! Its run-log could be found in ⌈2WXD⌋
PDB 2WYS was passing test before but now failed with ⌈unknown⌋ error! Its run-log could be found in ⌈2WYS⌋
PDB 2WZE was passing test before but now failed with ⌈unknown⌋ error! Its run-log could be found in ⌈2WZE⌋
PDB 2X2I was passing test before but now failed with ⌈fill_missing_atoms⌋ error! Its run-log could be found in ⌈2X2I⌋
PDB 2X87 was passing test before but now failed with ⌈fill_missing_atoms⌋ error! Its run-log could be found in ⌈2X87⌋
PDB 2X8S was passing test before but now failed with ⌈unknown⌋ error! Its run-log could be found in ⌈2X8S⌋
PDB 2XU9 was passing test before but now failed with ⌈fill_missing_atoms⌋ error! Its run-log could be found in ⌈2XU9⌋
PDB 2XXT was passing test before but now failed with ⌈unknown⌋ error! Its run-log could be found in ⌈2XXT⌋
PDB 2XXV was passing test before but now failed with ⌈unknown⌋ error! Its run-log could be found in ⌈2XXV⌋
PDB 2XXY was passing test before but now failed with ⌈unknown⌋ error! Its run-log could be found in ⌈2XXY⌋
PDB 2XY5 was passing test before but now failed with ⌈unrecognized_residue⌋ error! Its run-log could be found in ⌈2XY5⌋
PDB 2XYL was passing test before but now failed with ⌈unknown⌋ error! Its run-log could be found in ⌈2XYL⌋
PDB 2Y24 was passing test before but now failed with ⌈unknown⌋ error! Its run-log could be found in ⌈2Y24⌋
PDB 2Y64 was passing test before but now failed with ⌈unknown⌋ error! Its run-log could be found in ⌈2Y64⌋
PDB 2Y6K was passing test before but now failed with ⌈unknown⌋ error! Its run-log could be found in ⌈2Y6K⌋
PDB 2Y6L was passing test before but now failed with ⌈unknown⌋ error! Its run-log could be found in ⌈2Y6L⌋
PDB 2YAF was passing test before but now failed with ⌈fill_missing_atoms⌋ error! Its run-log could be found in ⌈2YAF⌋
PDB 2YAH was passing test before but now failed with ⌈fill_missing_atoms⌋ error! Its run-log could be found in ⌈2YAH⌋
PDB 2YAM was passing test before but now failed with ⌈fill_missing_atoms⌋ error! Its run-log could be found in ⌈2YAM⌋
PDB 2YAO was passing test before but now failed with ⌈fill_missing_atoms⌋ error! Its run-log could be found in ⌈2YAO⌋
PDB 2YAQ was passing test before but now failed with ⌈fill_missing_atoms⌋ error! Its run-log could be found in ⌈2YAQ⌋
PDB 2YAR was passing test before but now failed with ⌈fill_missing_atoms⌋ error! Its run-log could be found in ⌈2YAR⌋
PDB 2YD8 was passing test before but now failed with ⌈unrecognized_residue⌋ error! Its run-log could be found in ⌈2YD8⌋
PDB 2YDP was passing test before but now failed with ⌈fill_missing_atoms⌋ error! Its run-log could be found in ⌈2YDP⌋
PDB 2YDT was passing test before but now failed with ⌈fill_missing_atoms⌋ error! Its run-log could be found in ⌈2YDT⌋
PDB 2YGQ was passing test before but now failed with ⌈unrecognized_residue⌋ error! Its run-log could be found in ⌈2YGQ⌋
PDB 2Z2C was passing test before but now failed with ⌈bad_patch⌋ error! Its run-log could be found in ⌈2Z2C⌋
PDB 357D was passing test before but now failed with ⌈unknown⌋ error! Its run-log could be found in ⌈357D⌋
PDB 3A72 was passing test before but now failed with ⌈unknown⌋ error! Its run-log could be found in ⌈3A72⌋
PDB 3AKG was passing test before but now failed with ⌈unknown⌋ error! Its run-log could be found in ⌈3AKG⌋
PDB 3AKH was passing test before but now failed with ⌈unknown⌋ error! Its run-log could be found in ⌈3AKH⌋
PDB 3BVT was passing test before but now failed with ⌈unrecognized_residue⌋ error! Its run-log could be found in ⌈3BVT⌋
PDB 3BVU was passing test before but now failed with ⌈unrecognized_residue⌋ error! Its run-log could be found in ⌈3BVU⌋
PDB 3BVV was passing test before but now failed with ⌈unrecognized_residue⌋ error! Its run-log could be found in ⌈3BVV⌋
PDB 3BVW was passing test before but now failed with ⌈unrecognized_residue⌋ error! Its run-log could be found in ⌈3BVW⌋
PDB 3BVX was passing test before but now failed with ⌈unrecognized_residue⌋ error! Its run-log could be found in ⌈3BVX⌋
PDB 3BWH was passing test before but now failed with ⌈fill_missing_atoms⌋ error! Its run-log could be found in ⌈3BWH⌋
PDB 3BXD was passing test before but now failed with ⌈fill_missing_atoms⌋ error! Its run-log could be found in ⌈3BXD⌋
PDB 3C6Q was passing test before but now failed with ⌈unknown⌋ error! Its run-log could be found in ⌈3C6Q⌋
PDB 3C7F was passing test before but now failed with ⌈unknown⌋ error! Its run-log could be found in ⌈3C7F⌋
PDB 3C7G was passing test before but now failed with ⌈unknown⌋ error! Its run-log could be found in ⌈3C7G⌋
PDB 3C7H was passing test before but now failed with ⌈unknown⌋ error! Its run-log could be found in ⌈3C7H⌋
PDB 3CA0 was passing test before but now failed with ⌈fill_missing_atoms⌋ error! Its run-log could be found in ⌈3CA0⌋
PDB 3CIK was passing test before but now failed with ⌈reroot_disconnected⌋ error! Its run-log could be found in ⌈3CIK⌋
PDB 3CU1 was passing test before but now failed with ⌈unrecognized_residue⌋ error! Its run-log could be found in ⌈3CU1⌋
PDB 3CUI was passing test before but now failed with ⌈unrecognized_residue⌋ error! Its run-log could be found in ⌈3CUI⌋
PDB 3CUJ was passing test before but now failed with ⌈unrecognized_residue⌋ error! Its run-log could be found in ⌈3CUJ⌋
PDB 3CWH was passing test before but now failed with ⌈fill_missing_atoms⌋ error! Its run-log could be found in ⌈3CWH⌋
PDB 3D5Z was passing test before but now failed with ⌈unknown⌋ error! Its run-log could be found in ⌈3D5Z⌋
PDB 3D61 was passing test before but now failed with ⌈unknown⌋ error! Its run-log could be found in ⌈3D61⌋
PDB 3DTU was passing test before but now failed with ⌈fill_missing_atoms⌋ error! Its run-log could be found in ⌈3DTU⌋
PDB 3E5C was passing test before but now failed with ⌈bad_patch⌋ error! Its run-log could be found in ⌈3E5C⌋
PDB 3EN3 was passing test before but now failed with ⌈unknown⌋ error! Its run-log could be found in ⌈3EN3⌋
PDB 3ENZ was passing test before but now failed with ⌈fill_missing_atoms⌋ error! Its run-log could be found in ⌈3ENZ⌋
PDB 3EVJ was passing test before but now failed with ⌈unrecognized_residue⌋ error! Its run-log could be found in ⌈3EVJ⌋
PDB 3FP0 was passing test before but now failed with ⌈unrecognized_residue⌋ error! Its run-log could be found in ⌈3FP0⌋
PDB 3FYT was passing test before but now failed with ⌈fill_missing_atoms⌋ error! Its run-log could be found in ⌈3FYT⌋
PDB 3FYU was passing test before but now failed with ⌈fill_missing_atoms⌋ error! Its run-log could be found in ⌈3FYU⌋
PDB 3GO6 was passing test before but now failed with ⌈fill_missing_atoms⌋ error! Its run-log could be found in ⌈3GO6⌋
PDB 3GO7 was passing test before but now failed with ⌈fill_missing_atoms⌋ error! Its run-log could be found in ⌈3GO7⌋
PDB 3HKN was passing test before but now failed with ⌈unrecognized_residue⌋ error! Its run-log could be found in ⌈3HKN⌋
PDB 3I3Y was passing test before but now failed with ⌈unknown⌋ error! Its run-log could be found in ⌈3I3Y⌋
PDB 3IJ8 was passing test before but now failed with ⌈fill_missing_atoms⌋ error! Its run-log could be found in ⌈3IJ8⌋

PDB 3IJ9 was passing test before but now failed with 「fill_missing_atoms」 error! Its run-log could be found in 「3IJ9」
PDB 3IVA was passing test before but now failed with 「nu_conformer」 error! Its run-log could be found in 「3IVA」
PDB 3J4P was passing test before but now failed with 「unrecognized_residue」 error! Its run-log could be found in 「3J4P」
PDB 3KCG was passing test before but now failed with 「unrecognized_residue」 error! Its run-log could be found in 「3KCG」
PDB 3KFM was passing test before but now failed with 「unknown」 error! Its run-log could be found in 「3KFM」
PDB 3KL5 was passing test before but now failed with 「fill_missing_atoms」 error! Its run-log could be found in 「3KL5」
PDB 3KRW was passing test before but now failed with 「reroot_disconnected」 error! Its run-log could be found in 「3KRW」
PDB 3KRX was passing test before but now failed with 「reroot_disconnected」 error! Its run-log could be found in 「3KRX」
PDB 3KVR was passing test before but now failed with 「unknown」 error! Its run-log could be found in 「3KVR」
PDB 3KVV was passing test before but now failed with 「unknown」 error! Its run-log could be found in 「3KVV」
PDB 3KVY was passing test before but now failed with 「unknown」 error! Its run-log could be found in 「3KVY」
PDB 3L4J was passing test before but now failed with 「bad_patch」 error! Its run-log could be found in 「3L4J」
PDB 3L4K was passing test before but now failed with 「bad_patch」 error! Its run-log could be found in 「3L4K」
PDB 3LDJ was passing test before but now failed with 「unrecognized_residue」 error! Its run-log could be found in 「3LDJ」
PDB 3LHT was passing test before but now failed with 「alias_missing_atom」 error! Its run-log could be found in 「3LHT」
PDB 3LHU was passing test before but now failed with 「alias_missing_atom」 error! Its run-log could be found in 「3LHU」
PDB 3LHV was passing test before but now failed with 「alias_missing_atom」 error! Its run-log could be found in 「3LHV」
PDB 3LHW was passing test before but now failed with 「alias_missing_atom」 error! Its run-log could be found in 「3LHW」
PDB 3LHY was passing test before but now failed with 「alias_missing_atom」 error! Its run-log could be found in 「3LHY」
PDB 3LHZ was passing test before but now failed with 「alias_missing_atom」 error! Its run-log could be found in 「3LHZ」
PDB 3LI0 was passing test before but now failed with 「alias_missing_atom」 error! Its run-log could be found in 「3LI0」
PDB 3LI1 was passing test before but now failed with 「alias_missing_atom」 error! Its run-log could be found in 「3LI1」
PDB 3M9X was passing test before but now failed with 「unknown」 error! Its run-log could be found in 「3M9X」
PDB 3MA0 was passing test before but now failed with 「unknown」 error! Its run-log could be found in 「3MA0」
PDB 3MMD was passing test before but now failed with 「unknown」 error! Its run-log could be found in 「3MMD」
PDB 3MSD was passing test before but now failed with 「unknown」 error! Its run-log could be found in 「3MSD」
PDB 3MSG was passing test before but now failed with 「unknown」 error! Its run-log could be found in 「3MSG」
PDB 3MUA was passing test before but now failed with 「unknown」 error! Its run-log could be found in 「3MUA」
PDB 3MUI was passing test before but now failed with 「unknown」 error! Its run-log could be found in 「3MUI」
PDB 3N1X was passing test before but now failed with 「fill_missing_atoms」 error! Its run-log could be found in 「3N1X」
PDB 3NJ3 was passing test before but now failed with 「unknown」 error! Its run-log could be found in 「3NJ3」
PDB 3NPE was passing test before but now failed with 「fill_missing_atoms」 error! Its run-log could be found in 「3NPE」
PDB 3NW3 was passing test before but now failed with 「prepro_cyclic_pep」 error! Its run-log could be found in 「3NW3」
PDB 3OB0 was passing test before but now failed with 「unrecognized_residue」 error! Its run-log could be found in 「3OB0」
PDB 3OMA was passing test before but now failed with 「fill_missing_atoms」 error! Its run-log could be found in 「3OMA」
PDB 3OMI was passing test before but now failed with 「fill_missing_atoms」 error! Its run-log could be found in 「3OMI」
PDB 3OMN was passing test before but now failed with 「fill_missing_atoms」 error! Its run-log could be found in 「3OMN」
PDB 3P0I was passing test before but now failed with 「unknown_atom_name」 error! Its run-log could be found in 「3P0I」
PDB 3P0J was passing test before but now failed with 「unknown_atom_name」 error! Its run-log could be found in 「3P0J」
PDB 3PER was passing test before but now failed with 「fill_missing_atoms」 error! Its run-log could be found in 「3PER」
PDB 3PF7 was passing test before but now failed with 「fill_missing_atoms」 error! Its run-log could be found in 「3PF7」
PDB 3PMA was passing test before but now failed with 「unrecognized_residue」 error! Its run-log could be found in 「3PMA」
PDB 3PSC was passing test before but now failed with 「reroot_disconnected」 error! Its run-log could be found in 「3PSC」
PDB 3PVW was passing test before but now failed with 「reroot_disconnected」 error! Its run-log could be found in 「3PVW」
PDB 3Q1G was passing test before but now failed with 「fill_missing_atoms」 error! Its run-log could be found in 「3Q1G」
PDB 3Q7G was passing test before but now failed with 「unrecognized_residue」 error! Its run-log could be found in 「3Q7G」
PDB 3QBA was passing test before but now failed with 「unrecognized_residue」 error! Its run-log could be found in 「3QBA」
PDB 3QDX was passing test before but now failed with 「fill_missing_atoms」 error! Its run-log could be found in 「3QDX」
PDB 3QDY was passing test before but now failed with 「fill_missing_atoms」 error! Its run-log could be found in 「3QDY」
PDB 3QEF was passing test before but now failed with 「unknown」 error! Its run-log could be found in 「3QEF」
PDB 3QRC was passing test before but now failed with 「unrecognized_residue」 error! Its run-log could be found in 「3QRC」
PDB 3RCY was passing test before but now failed with 「unknown」 error! Its run-log could be found in 「3RCY」
PDB 3RDK was passing test before but now failed with 「fill_missing_atoms」 error! Its run-log could be found in 「3RDK」
PDB 3RN9 was passing test before but now failed with 「fill_missing_atoms」 error! Its run-log could be found in 「3RN9」
PDB 3RNA was passing test before but now failed with 「fill_missing_atoms」 error! Its run-log could be found in 「3RNA」
PDB 3RNB was passing test before but now failed with 「fill_missing_atoms」 error! Its run-log could be found in 「3RNB」
PDB 3RNC was passing test before but now failed with 「fill_missing_atoms」 error! Its run-log could be found in 「3RNC」
PDB 3RNE was passing test before but now failed with 「fill_missing_atoms」 error! Its run-log could be found in 「3RNE」
PDB 3RNG was passing test before but now failed with 「fill_missing_atoms」 error! Its run-log could be found in 「3RNG」
PDB 3S5Y was passing test before but now failed with 「fill_missing_atoms」 error! Its run-log could be found in 「3S5Y」
PDB 3S5Z was passing test before but now failed with 「fill_missing_atoms」 error! Its run-log could be found in 「3S5Z」
PDB 3T5G was passing test before but now failed with 「reroot_disconnected」 error! Its run-log could be found in 「3T5G」
PDB 3T5I was passing test before but now failed with 「reroot_disconnected」 error! Its run-log could be found in 「3T5I」
PDB 3T9H was passing test before but now failed with 「unknown」 error! Its run-log could be found in 「3T9H」
PDB 3TA2 was passing test before but now failed with 「nu_conformer」 error! Its run-log could be found in 「3TA2」
PDB 3TI3 was passing test before but now failed with 「fill_missing_atoms」 error! Its run-log could be found in 「3TI3」
PDB 3TI8 was passing test before but now failed with 「fill_missing_atoms」 error! Its run-log could be found in 「3TI8」
PDB 3TIA was passing test before but now failed with 「fill_missing_atoms」 error! Its run-log could be found in 「3TIA」
PDB 3U92 was passing test before but now failed with 「unknown」 error! Its run-log could be found in 「3U92」
PDB 3UER was passing test before but now failed with 「unrecognized_residue」 error! Its run-log could be found in 「3UER」
PDB 3UG4 was passing test before but now failed with 「unknown」 error! Its run-log could be found in 「3UG4」
PDB 3UG5 was passing test before but now failed with 「unknown」 error! Its run-log could be found in 「3UG5」
PDB 3UGW was passing test before but now failed with 「unrecognized_residue」 error! Its run-log could be found in 「3UGW」
PDB 3USU was passing test before but now failed with 「fill_missing_atoms」 error! Its run-log could be found in 「3USU」
PDB 3UZS was passing test before but now failed with 「reroot_disconnected」 error! Its run-log could be found in 「3UZS」
PDB 3V8V was passing test before but now failed with 「unrecognized_residue」 error! Its run-log could be found in 「3V8V」
PDB 3V97 was passing test before but now failed with 「unrecognized_residue」 error! Its run-log could be found in 「3V97」
PDB 3VPL was passing test before but now failed with 「unknown」 error! Its run-log could be found in 「3VPL」
PDB 3VSJ was passing test before but now failed with 「fill_missing_atoms」 error! Its run-log could be found in 「3VSJ」
PDB 3VSU was passing test before but now failed with 「unknown」 error! Its run-log could be found in 「3VSU」
PDB 3VSV was passing test before but now failed with 「fill_missing_atoms」 error! Its run-log could be found in 「3VSV」

PDB 3VV9 was passing test before but now failed with 「fill_missing_atoms」 error! Its run-log could be found in 「3VV9」
PDB 3VVA was passing test before but now failed with 「fill_missing_atoms」 error! Its run-log could be found in 「3VVA」
PDB 3VXC was passing test before but now failed with 「unknown」 error! Its run-log could be found in 「3VXC」
PDB 3VZM was passing test before but now failed with 「unknown」 error! Its run-log could be found in 「3VZM」
PDB 3VZN was passing test before but now failed with 「unknown」 error! Its run-log could be found in 「3VZN」
PDB 3VZO was passing test before but now failed with 「unknown」 error! Its run-log could be found in 「3VZO」
PDB 3W25 was passing test before but now failed with 「unknown」 error! Its run-log could be found in 「3W25」
PDB 3W26 was passing test before but now failed with 「unknown」 error! Its run-log could be found in 「3W26」
PDB 3W27 was passing test before but now failed with 「unknown」 error! Its run-log could be found in 「3W27」
PDB 3W28 was passing test before but now failed with 「unknown」 error! Its run-log could be found in 「3W28」
PDB 3W29 was passing test before but now failed with 「unknown」 error! Its run-log could be found in 「3W29」
PDB 3W54 was passing test before but now failed with 「fill_missing_atoms」 error! Its run-log could be found in 「3W54」
PDB 3WKX was passing test before but now failed with 「fill_missing_atoms」 error! Its run-log could be found in 「3WKX」
PDB 3WLH was passing test before but now failed with 「fill_missing_atoms」 error! Its run-log could be found in 「3WLH」
PDB 3WLJ was passing test before but now failed with 「fill_missing_atoms」 error! Its run-log could be found in 「3WLJ」
PDB 3WLK was passing test before but now failed with 「fill_missing_atoms」 error! Its run-log could be found in 「3WLK」
PDB 3WLL was passing test before but now failed with 「fill_missing_atoms」 error! Its run-log could be found in 「3WLL」
PDB 3WN0 was passing test before but now failed with 「fill_missing_atoms」 error! Its run-log could be found in 「3WN0」
PDB 3WN1 was passing test before but now failed with 「unknown」 error! Its run-log could be found in 「3WN1」
PDB 3WN2 was passing test before but now failed with 「unknown」 error! Its run-log could be found in 「3WN2」
PDB 3WP6 was passing test before but now failed with 「unknown」 error! Its run-log could be found in 「3WP6」
PDB 3WRG was passing test before but now failed with 「fill_missing_atoms」 error! Its run-log could be found in 「3WRG」
PDB 3WUE was passing test before but now failed with 「unknown」 error! Its run-log could be found in 「3WUE」
PDB 3WUG was passing test before but now failed with 「unknown」 error! Its run-log could be found in 「3WUG」
PDB 3ZKK was passing test before but now failed with 「unknown」 error! Its run-log could be found in 「3ZKK」
PDB 3ZKL was passing test before but now failed with 「unknown」 error! Its run-log could be found in 「3ZKL」
PDB 3ZSE was passing test before but now failed with 「unknown」 error! Its run-log could be found in 「3ZSE」
PDB 3ZXK was passing test before but now failed with 「unknown」 error! Its run-log could be found in 「3ZXK」
PDB 4A5G was passing test before but now failed with 「fill_missing_atoms」 error! Its run-log could be found in 「4A5G」
PDB 4A68 was passing test before but now failed with 「fill_missing_atoms」 error! Its run-log could be found in 「4A68」
PDB 4AC7 was passing test before but now failed with 「fill_missing_atoms」 error! Its run-log could be found in 「4AC7」
PDB 4AYP was passing test before but now failed with 「unrecognized_residue」 error! Its run-log could be found in 「4AYP」
PDB 4BDM was passing test before but now failed with 「unknown」 error! Its run-log could be found in 「4BDM」
PDB 4BDO was passing test before but now failed with 「unknown」 error! Its run-log could be found in 「4BDO」
PDB 4BDR was passing test before but now failed with 「unknown」 error! Its run-log could be found in 「4BDR」
PDB 4BQC was passing test before but now failed with 「unrecognized_residue」 error! Its run-log could be found in 「4BQC」
PDB 4BQF was passing test before but now failed with 「fill_missing_atoms」 error! Its run-log could be found in 「4BQF」
PDB 4C0N was passing test before but now failed with 「fill_missing_atoms」 error! Its run-log could be found in 「4C0N」
PDB 4C1P was passing test before but now failed with 「unknown」 error! Its run-log could be found in 「4C1P」
PDB 4C1T was passing test before but now failed with 「unknown」 error! Its run-log could be found in 「4C1T」
PDB 4C1U was passing test before but now failed with 「unknown」 error! Its run-log could be found in 「4C1U」
PDB 4CDR was passing test before but now failed with 「bad_patch」 error! Its run-log could be found in 「4CDR」
PDB 4CEU was passing test before but now failed with 「fill_missing_atoms」 error! Its run-log could be found in 「4CEU」
PDB 4D4Z was passing test before but now failed with 「fill_missing_atoms」 error! Its run-log could be found in 「4D4Z」
PDB 4D5I was passing test before but now failed with 「unknown」 error! Its run-log could be found in 「4D5I」
PDB 4D5J was passing test before but now failed with 「unknown」 error! Its run-log could be found in 「4D5J」
PDB 4D5O was passing test before but now failed with 「fill_missing_atoms」 error! Its run-log could be found in 「4D5O」
PDB 4D5P was passing test before but now failed with 「unknown」 error! Its run-log could be found in 「4D5P」
PDB 4D5Q was passing test before but now failed with 「unknown」 error! Its run-log could be found in 「4D5Q」
PDB 4D5V was passing test before but now failed with 「unknown」 error! Its run-log could be found in 「4D5V」
PDB 4DIG was passing test before but now failed with 「prepro_cyclic_pep」 error! Its run-log could be found in 「4DIG」
PDB 4E0W was passing test before but now failed with 「unknown」 error! Its run-log could be found in 「4E0W」
PDB 4E0X was passing test before but now failed with 「unknown」 error! Its run-log could be found in 「4E0X」
PDB 4E9V was passing test before but now failed with 「fill_missing_atoms」 error! Its run-log could be found in 「4E9V」
PDB 4EAQ was passing test before but now failed with 「duplicate_atom_name」 error! Its run-log could be found in 「4EAQ」
PDB 4EF3 was passing test before but now failed with 「fill_missing_atoms」 error! Its run-log could be found in 「4EF3」
PDB 4F22 was passing test before but now failed with 「unknown」 error! Its run-log could be found in 「4F22」
PDB 4F31 was passing test before but now failed with 「unknown」 error! Its run-log could be found in 「4F31」
PDB 4F39 was passing test before but now failed with 「unknown」 error! Its run-log could be found in 「4F39」
PDB 4F3G was passing test before but now failed with 「unknown」 error! Its run-log could be found in 「4F3G」
PDB 4G68 was passing test before but now failed with 「unknown」 error! Its run-log could be found in 「4G68」
PDB 4GBY was passing test before but now failed with 「fill_missing_atoms」 error! Its run-log could be found in 「4GBY」
PDB 4GFH was passing test before but now failed with 「bad_patch」 error! Its run-log could be found in 「4GFH」
PDB 4GMD was passing test before but now failed with 「duplicate_atom_name」 error! Its run-log could be found in 「4GMD」
PDB 4H9U was passing test before but now failed with 「fill_missing_atoms」 error! Its run-log could be found in 「4H9U」
PDB 4H9V was passing test before but now failed with 「fill_missing_atoms」 error! Its run-log could be found in 「4H9V」
PDB 4H9X was passing test before but now failed with 「fill_missing_atoms」 error! Its run-log could be found in 「4H9X」
PDB 4H9Y was passing test before but now failed with 「fill_missing_atoms」 error! Its run-log could be found in 「4H9Y」
PDB 4HA0 was passing test before but now failed with 「fill_missing_atoms」 error! Its run-log could be found in 「4HA0」
PDB 4HAK was passing test before but now failed with 「fill_missing_atoms」 error! Its run-log could be found in 「4HAK」
PDB 4HAL was passing test before but now failed with 「fill_missing_atoms」 error! Its run-log could be found in 「4HAL」
PDB 4HK8 was passing test before but now failed with 「unknown」 error! Its run-log could be found in 「4HK8」
PDB 4HK9 was passing test before but now failed with 「unknown」 error! Its run-log could be found in 「4HK9」
PDB 4HKW was passing test before but now failed with 「unknown」 error! Its run-log could be found in 「4HKW」
PDB 4HZW was passing test before but now failed with 「fill_missing_atoms」 error! Its run-log could be found in 「4HZW」
PDB 4IPN was passing test before but now failed with 「unrecognized_residue」 error! Its run-log could be found in 「4IPN」
PDB 4JCK was passing test before but now failed with 「unrecognized_residue」 error! Its run-log could be found in 「4JCK」
PDB 4L4P was passing test before but now failed with 「unknown」 error! Its run-log could be found in 「4L4P」
PDB 4LR9 was passing test before but now failed with 「fill_missing_atoms」 error! Its run-log could be found in 「4LR9」
PDB 4LRB was passing test before but now failed with 「fill_missing_atoms」 error! Its run-log could be found in 「4LRB」
PDB 4LRE was passing test before but now failed with 「fill_missing_atoms」 error! Its run-log could be found in 「4LRE」

PDB 4M4K was passing test before but now failed with ⌜unknown⌟ error! Its run-log could be found in ⌜4M4K⌟
PDB 4MAV was passing test before but now failed with ⌜unknown⌟ error! Its run-log could be found in ⌜4MAV⌟
PDB 4MWU was passing test before but now failed with ⌜fill_missing_atoms⌟ error! Its run-log could be found in ⌜4MWU⌟
PDB 4MWY was passing test before but now failed with ⌜fill_missing_atoms⌟ error! Its run-log could be found in ⌜4MWY⌟
PDB 4N2R was passing test before but now failed with ⌜fill_missing_atoms⌟ error! Its run-log could be found in ⌜4N2R⌟
PDB 4NER was passing test before but now failed with ⌜fill_missing_atoms⌟ error! Its run-log could be found in ⌜4NER⌟
PDB 4O8O was passing test before but now failed with ⌜unknown⌟ error! Its run-log could be found in ⌜4O8O⌟
PDB 4O8P was passing test before but now failed with ⌜unknown⌟ error! Its run-log could be found in ⌜4O8P⌟
PDB 4PMD was passing test before but now failed with ⌜unknown⌟ error! Its run-log could be found in ⌜4PMD⌟
PDB 4PMY was passing test before but now failed with ⌜fill_missing_atoms⌟ error! Its run-log could be found in ⌜4PMY⌟
PDB 4PMZ was passing test before but now failed with ⌜unknown⌟ error! Its run-log could be found in ⌜4PMZ⌟
PDB 4PN2 was passing test before but now failed with ⌜unknown⌟ error! Its run-log could be found in ⌜4PN2⌟
PDB 4PRW was passing test before but now failed with ⌜unknown⌟ error! Its run-log could be found in ⌜4PRW⌟
PDB 4PUD was passing test before but now failed with ⌜unknown⌟ error! Its run-log could be found in ⌜4PUD⌟
PDB 4PUE was passing test before but now failed with ⌜unknown⌟ error! Its run-log could be found in ⌜4PUE⌟
PDB 4PVI was passing test before but now failed with ⌜unknown⌟ error! Its run-log could be found in ⌜4PVI⌟
PDB 4Q27 was passing test before but now failed with ⌜unrecognized_residue⌟ error! Its run-log could be found in ⌜4Q27⌟
PDB 4QB6 was passing test before but now failed with ⌜fill_missing_atoms⌟ error! Its run-log could be found in ⌜4QB6⌟
PDB 4QEH was passing test before but now failed with ⌜unknown⌟ error! Its run-log could be found in ⌜4QEH⌟
PDB 4QN6 was passing test before but now failed with ⌜fill_missing_atoms⌟ error! Its run-log could be found in ⌜4QN6⌟
PDB 4QPW was passing test before but now failed with ⌜unknown⌟ error! Its run-log could be found in ⌜4QPW⌟
PDB 4R9W was passing test before but now failed with ⌜unrecognized_residue⌟ error! Its run-log could be found in ⌜4R9W⌟
PDB 4RHN was passing test before but now failed with ⌜unknown⌟ error! Its run-log could be found in ⌜4RHN⌟
PDB 4RK0 was passing test before but now failed with ⌜unknown⌟ error! Its run-log could be found in ⌜4RK0⌟
PDB 4RK1 was passing test before but now failed with ⌜fill_missing_atoms⌟ error! Its run-log could be found in ⌜4RK1⌟
PDB 4RYK was passing test before but now failed with ⌜fill_missing_atoms⌟ error! Its run-log could be found in ⌜4RYK⌟
PDB 4RZT was passing test before but now failed with ⌜unrecognized_residue⌟ error! Its run-log could be found in ⌜4RZT⌟
PDB 4U1O was passing test before but now failed with ⌜unknown⌟ error! Its run-log could be found in ⌜4U1O⌟
PDB 4U1W was passing test before but now failed with ⌜unknown⌟ error! Its run-log could be found in ⌜4U1W⌟
PDB 4U1X was passing test before but now failed with ⌜unknown⌟ error! Its run-log could be found in ⌜4U1X⌟
PDB 4U1Z was passing test before but now failed with ⌜unknown⌟ error! Its run-log could be found in ⌜4U1Z⌟
PDB 4U2Q was passing test before but now failed with ⌜unknown⌟ error! Its run-log could be found in ⌜4U2Q⌟
PDB 4U2Z was passing test before but now failed with ⌜unrecognized_residue⌟ error! Its run-log could be found in ⌜4U2Z⌟
PDB 4U5B was passing test before but now failed with ⌜unknown⌟ error! Its run-log could be found in ⌜4U5B⌟
PDB 4U5D was passing test before but now failed with ⌜unknown⌟ error! Its run-log could be found in ⌜4U5D⌟
PDB 4U5E was passing test before but now failed with ⌜unknown⌟ error! Its run-log could be found in ⌜4U5E⌟
PDB 4U5F was passing test before but now failed with ⌜unknown⌟ error! Its run-log could be found in ⌜4U5F⌟
PDB 4U5I was passing test before but now failed with ⌜unknown⌟ error! Its run-log could be found in ⌜4U5I⌟
PDB 4UY2 was passing test before but now failed with ⌜multiple_disulfides⌟ error! Its run-log could be found in ⌜4UY2⌟
PDB 4UZ6 was passing test before but now failed with ⌜unrecognized_residue⌟ error! Its run-log could be found in ⌜4UZ6⌟
PDB 4UZ9 was passing test before but now failed with ⌜unrecognized_residue⌟ error! Its run-log could be found in ⌜4UZ9⌟
PDB 4WEF was passing test before but now failed with ⌜fill_missing_atoms⌟ error! Its run-log could be found in ⌜4WEF⌟
PDB 4WEG was passing test before but now failed with ⌜fill_missing_atoms⌟ error! Its run-log could be found in ⌜4WEG⌟
PDB 4X7R was passing test before but now failed with ⌜unrecognized_residue⌟ error! Its run-log could be found in ⌜4X7R⌟
PDB 4XDA was passing test before but now failed with ⌜fill_missing_atoms⌟ error! Its run-log could be found in ⌜4XDA⌟
PDB 4XJA was passing test before but now failed with ⌜fill_missing_atoms⌟ error! Its run-log could be found in ⌜4XJA⌟
PDB 4XUQ was passing test before but now failed with ⌜unknown⌟ error! Its run-log could be found in ⌜4XUQ⌟
PDB 4XUR was passing test before but now failed with ⌜unknown⌟ error! Its run-log could be found in ⌜4XUR⌟
PDB 4XWO was passing test before but now failed with ⌜bad_patch⌟ error! Its run-log could be found in ⌜4XWO⌟
PDB 4Y27 was passing test before but now failed with ⌜unknown⌟ error! Its run-log could be found in ⌜4Y27⌟
PDB 4YGK was passing test before but now failed with ⌜fill_missing_atoms⌟ error! Its run-log could be found in ⌜4YGK⌟
PDB 4YGL was passing test before but now failed with ⌜fill_missing_atoms⌟ error! Its run-log could be found in ⌜4YGL⌟
PDB 4YWH was passing test before but now failed with ⌜unknown⌟ error! Its run-log could be found in ⌜4YWH⌟
PDB 4YZ5 was passing test before but now failed with ⌜fill_missing_atoms⌟ error! Its run-log could be found in ⌜4YZ5⌟
PDB 4Z8S was passing test before but now failed with ⌜fill_missing_atoms⌟ error! Its run-log could be found in ⌜4Z8S⌟
PDB 4ZE9 was passing test before but now failed with ⌜unrecognized_residue⌟ error! Its run-log could be found in ⌜4ZE9⌟
PDB 4ZEB was passing test before but now failed with ⌜unrecognized_residue⌟ error! Its run-log could be found in ⌜4ZEB⌟
PDB 4ZFU was passing test before but now failed with ⌜fill_missing_atoms⌟ error! Its run-log could be found in ⌜4ZFU⌟
PDB 4ZFW was passing test before but now failed with ⌜fill_missing_atoms⌟ error! Its run-log could be found in ⌜4ZFW⌟
PDB 4ZGR was passing test before but now failed with ⌜fill_missing_atoms⌟ error! Its run-log could be found in ⌜4ZGR⌟
PDB 4ZLB was passing test before but now failed with ⌜fill_missing_atoms⌟ error! Its run-log could be found in ⌜4ZLB⌟
PDB 4ZRV was passing test before but now failed with ⌜unrecognized_residue⌟ error! Its run-log could be found in ⌜4ZRV⌟
PDB 4ZTT was passing test before but now failed with ⌜fill_missing_atoms⌟ error! Its run-log could be found in ⌜4ZTT⌟
PDB 5A03 was passing test before but now failed with ⌜fill_missing_atoms⌟ error! Its run-log could be found in ⌜5A03⌟
PDB 5A6L was passing test before but now failed with ⌜unknown⌟ error! Its run-log could be found in ⌜5A6L⌟
PDB 5A6M was passing test before but now failed with ⌜unknown⌟ error! Its run-log could be found in ⌜5A6M⌟
PDB 5ACU was passing test before but now failed with ⌜fill_missing_atoms⌟ error! Its run-log could be found in ⌜5ACU⌟
PDB 5ACV was passing test before but now failed with ⌜fill_missing_atoms⌟ error! Its run-log could be found in ⌜5ACV⌟
PDB 5ACW was passing test before but now failed with ⌜fill_missing_atoms⌟ error! Its run-log could be found in ⌜5ACW⌟
PDB 5ACX was passing test before but now failed with ⌜fill_missing_atoms⌟ error! Its run-log could be found in ⌜5ACX⌟
PDB 5AE6 was passing test before but now failed with ⌜unknown⌟ error! Its run-log could be found in ⌜5AE6⌟
PDB 5AFA was passing test before but now failed with ⌜fill_missing_atoms⌟ error! Its run-log could be found in ⌜5AFA⌟
PDB 5AOG was passing test before but now failed with ⌜fill_missing_atoms⌟ error! Its run-log could be found in ⌜5AOG⌟
PDB 5BQC was passing test before but now failed with ⌜unrecognized_residue⌟ error! Its run-log could be found in ⌜5BQC⌟
PDB 5BU2 was passing test before but now failed with ⌜unknown⌟ error! Its run-log could be found in ⌜5BU2⌟
PDB 5C1M was passing test before but now failed with ⌜unrecognized_residue⌟ error! Its run-log could be found in ⌜5C1M⌟
PDB 5CJH was passing test before but now failed with ⌜fill_missing_atoms⌟ error! Its run-log could be found in ⌜5CJH⌟
PDB 5COY was passing test before but now failed with ⌜unrecognized_residue⌟ error! Its run-log could be found in ⌜5COY⌟
PDB 5D15 was passing test before but now failed with ⌜bad_patch⌟ error! Its run-log could be found in ⌜5D15⌟
PDB 5D4Y was passing test before but now failed with ⌜unknown⌟ error! Its run-log could be found in ⌜5D4Y⌟
PDB 5E8F was passing test before but now failed with ⌜reroot_disconnected⌟ error! Its run-log could be found in ⌜5E8F⌟

PDB 5F2U was passing test before but now failed with 「reroot_disconnected」 error! Its run-log could be found in 「5F2U」
PDB 5F9T was passing test before but now failed with 「fill_missing_atoms」 error! Its run-log could be found in 「5F9T」
PDB 5FBD was passing test before but now failed with 「unrecognized_residue」 error! Its run-log could be found in 「5FBD」
PDB 5FBG was passing test before but now failed with 「unrecognized_residue」 error! Its run-log could be found in 「5FBG」
PDB 5FQA was passing test before but now failed with 「fill_missing_atoms」 error! Its run-log could be found in 「5FQA」
PDB 5G62 was passing test before but now failed with 「unrecognized_residue」 error! Its run-log could be found in 「5G62」
PDB 5GLM was passing test before but now failed with 「unknown」 error! Its run-log could be found in 「5GLM」
PDB 5GLN was passing test before but now failed with 「unknown」 error! Its run-log could be found in 「5GLN」
PDB 5GLO was passing test before but now failed with 「fill_missing_atoms」 error! Its run-log could be found in 「5GLO」
PDB 5GLQ was passing test before but now failed with 「unknown」 error! Its run-log could be found in 「5GLQ」
PDB 5GLR was passing test before but now failed with 「unknown」 error! Its run-log could be found in 「5GLR」
PDB 5GN7 was passing test before but now failed with 「fill_missing_atoms」 error! Its run-log could be found in 「5GN7」
PDB 5GQD was passing test before but now failed with 「unknown」 error! Its run-log could be found in 「5GQD」
PDB 5GQE was passing test before but now failed with 「unknown」 error! Its run-log could be found in 「5GQE」
PDB 5HJO was passing test before but now failed with 「unrecognized_residue」 error! Its run-log could be found in 「5HJO」
PDB 5HO9 was passing test before but now failed with 「unknown」 error! Its run-log could be found in 「5HO9」
PDB 5HOF was passing test before but now failed with 「unknown」 error! Its run-log could be found in 「5HOF」
PDB 5HON was passing test before but now failed with 「unknown」 error! Its run-log could be found in 「5HON」
PDB 5HPO was passing test before but now failed with 「fill_missing_atoms」 error! Its run-log could be found in 「5HPO」
PDB 5HQJ was passing test before but now failed with 「fill_missing_atoms」 error! Its run-log could be found in 「5HQJ」
PDB 5IBQ was passing test before but now failed with 「fill_missing_atoms」 error! Its run-log could be found in 「5IBQ」
PDB 5IJC was passing test before but now failed with 「unrecognized_residue」 error! Its run-log could be found in 「5IJC」
PDB 5IKB was passing test before but now failed with 「unknown」 error! Its run-log could be found in 「5IKB」
PDB 5K6N was passing test before but now failed with 「fill_missing_atoms」 error! Its run-log could be found in 「5K6N」
PDB 5K73 was passing test before but now failed with 「fill_missing_atoms」 error! Its run-log could be found in 「5K73」
PDB 5K77 was passing test before but now failed with 「fill_missing_atoms」 error! Its run-log could be found in 「5K77」
PDB 5K8Z was passing test before but now failed with 「fill_missing_atoms」 error! Its run-log could be found in 「5K8Z」
PDB 5KK7 was passing test before but now failed with 「unrecognized_residue」 error! Its run-log could be found in 「5KK7」
PDB 5KXB was passing test before but now failed with 「fill_missing_atoms」 error! Its run-log could be found in 「5KXB」
PDB 5KXD was passing test before but now failed with 「fill_missing_atoms」 error! Its run-log could be found in 「5KXD」
PDB 5KXE was passing test before but now failed with 「fill_missing_atoms」 error! Its run-log could be found in 「5KXE」
PDB 5LA1 was passing test before but now failed with 「fill_missing_atoms」 error! Its run-log could be found in 「5LA1」
PDB 5LA2 was passing test before but now failed with 「unknown」 error! Its run-log could be found in 「5LA2」
PDB 5LAL was passing test before but now failed with 「fill_missing_atoms」 error! Its run-log could be found in 「5LAL」
PDB 5LQO was passing test before but now failed with 「no_orient_atoms」 error! Its run-log could be found in 「5LQO」
PDB 5LQT was passing test before but now failed with 「no_orient_atoms」 error! Its run-log could be found in 「5LQT」
PDB 5LR3 was passing test before but now failed with 「no_orient_atoms」 error! Its run-log could be found in 「5LR3」
PDB 5LR4 was passing test before but now failed with 「no_orient_atoms」 error! Its run-log could be found in 「5LR4」
PDB 5LR5 was passing test before but now failed with 「no_orient_atoms」 error! Its run-log could be found in 「5LR5」
PDB 5LSH was passing test before but now failed with 「unrecognized_residue」 error! Its run-log could be found in 「5LSH」
PDB 5M1Z was passing test before but now failed with 「unknown」 error! Its run-log could be found in 「5M1Z」
PDB 5M4G was passing test before but now failed with 「fill_missing_atoms」 error! Its run-log could be found in 「5M4G」
PDB 5M4L was passing test before but now failed with 「fill_missing_atoms」 error! Its run-log could be found in 「5M4L」
PDB 5M4Q was passing test before but now failed with 「fill_missing_atoms」 error! Its run-log could be found in 「5M4Q」
PDB 5M77 was passing test before but now failed with 「fill_missing_atoms」 error! Its run-log could be found in 「5M77」
PDB 5M93 was passing test before but now failed with 「unknown」 error! Its run-log could be found in 「5M93」
PDB 5MBY was passing test before but now failed with 「fill_missing_atoms」 error! Its run-log could be found in 「5MBY」
PDB 5MBZ was passing test before but now failed with 「fill_missing_atoms」 error! Its run-log could be found in 「5MBZ」
PDB 5MC0 was passing test before but now failed with 「fill_missing_atoms」 error! Its run-log could be found in 「5MC0」
PDB 5MC3 was passing test before but now failed with 「fill_missing_atoms」 error! Its run-log could be found in 「5MC3」
PDB 5MC4 was passing test before but now failed with 「fill_missing_atoms」 error! Its run-log could be found in 「5MC4」
PDB 5MC5 was passing test before but now failed with 「fill_missing_atoms」 error! Its run-log could be found in 「5MC5」
PDB 5MFQ was passing test before but now failed with 「unknown」 error! Its run-log could be found in 「5MFQ」
PDB 5MFV was passing test before but now failed with 「unknown」 error! Its run-log could be found in 「5MFV」
PDB 5MQS was passing test before but now failed with 「fill_missing_atoms」 error! Its run-log could be found in 「5MQS」
PDB 5MUI was passing test before but now failed with 「fill_missing_atoms」 error! Its run-log could be found in 「5MUI」
PDB 5MUJ was passing test before but now failed with 「fill_missing_atoms」 error! Its run-log could be found in 「5MUJ」
PDB 5MWB was passing test before but now failed with 「unknown」 error! Its run-log could be found in 「5MWB」
PDB 5NF7 was passing test before but now failed with 「unrecognized_residue」 error! Its run-log could be found in 「5NF7」
PDB 5NFA was passing test before but now failed with 「unrecognized_residue」 error! Its run-log could be found in 「5NFA」
PDB 5NH7 was passing test before but now failed with 「fill_missing_atoms」 error! Its run-log could be found in 「5NH7」
PDB 5NH8 was passing test before but now failed with 「fill_missing_atoms」 error! Its run-log could be found in 「5NH8」
PDB 5NH9 was passing test before but now failed with 「unknown」 error! Its run-log could be found in 「5NH9」
PDB 5NHD was passing test before but now failed with 「unknown」 error! Its run-log could be found in 「5NHD」
PDB 5NHE was passing test before but now failed with 「fill_missing_atoms」 error! Its run-log could be found in 「5NHE」
PDB 5NJW was passing test before but now failed with 「fill_missing_atoms」 error! Its run-log could be found in 「5NJW」
PDB 5NKU was passing test before but now failed with 「fill_missing_atoms」 error! Its run-log could be found in 「5NKU」
PDB 5NLN was passing test before but now failed with 「unknown」 error! Its run-log could be found in 「5NLN」
PDB 5NLO was passing test before but now failed with 「unknown」 error! Its run-log could be found in 「5NLO」
PDB 5NLP was passing test before but now failed with 「unknown」 error! Its run-log could be found in 「5NLP」
PDB 5NLQ was passing test before but now failed with 「unknown」 error! Its run-log could be found in 「5NLQ」
PDB 5NN8 was passing test before but now failed with 「fill_missing_atoms」 error! Its run-log could be found in 「5NN8」
PDB 5NZD was passing test before but now failed with 「no_orient_atoms」 error! Its run-log could be found in 「5NZD」
PDB 5O80 was passing test before but now failed with 「fill_missing_atoms」 error! Its run-log could be found in 「5O80」
PDB 5O82 was passing test before but now failed with 「unknown」 error! Its run-log could be found in 「5O82」
PDB 5OCA was passing test before but now failed with 「unrecognized_residue」 error! Its run-log could be found in 「5OCA」
PDB 5OCP was passing test before but now failed with 「unknown」 error! Its run-log could be found in 「5OCP」
PDB 5OCZ was passing test before but now failed with 「unrecognized_residue」 error! Its run-log could be found in 「5OCZ」
PDB 5ODF was passing test before but now failed with 「unrecognized_residue」 error! Its run-log could be found in 「5ODF」
PDB 5ODM was passing test before but now failed with 「unrecognized_residue」 error! Its run-log could be found in 「5ODM」
PDB 5OE1 was passing test before but now failed with 「unrecognized_residue」 error! Its run-log could be found in 「5OE1」

PDB 5OFK was passing test before but now failed with 「unknown」 error! Its run-log could be found in 「5OFK」
PDB 5OPJ was passing test before but now failed with 「fill_missing_atoms」 error! Its run-log could be found in 「5OPJ」
PDB 5TAR was passing test before but now failed with 「reroot_disconnected」 error! Its run-log could be found in 「5TAR」
PDB 5TB5 was passing test before but now failed with 「reroot_disconnected」 error! Its run-log could be found in 「5TB5」
PDB 5TIP was passing test before but now failed with 「fill_missing_atoms」 error! Its run-log could be found in 「5TIP」
PDB 5TIQ was passing test before but now failed with 「fill_missing_atoms」 error! Its run-log could be found in 「5TIQ」
PDB 5UCY was passing test before but now failed with 「bad_patch」 error! Its run-log could be found in 「5UCY」
PDB 5UF6 was passing test before but now failed with 「unrecognized_residue」 error! Its run-log could be found in 「5UF6」
PDB 5UHZ was passing test before but now failed with 「fill_missing_atoms」 error! Its run-log could be found in 「5UHZ」
PDB 5UKM was passing test before but now failed with 「reroot_disconnected」 error! Its run-log could be found in 「5UKM」
PDB 5UN3 was passing test before but now failed with 「unknown」 error! Its run-log could be found in 「5UN3」
PDB 5UQ6 was passing test before but now failed with 「fill_missing_atoms」 error! Its run-log could be found in 「5UQ6」
PDB 5UU8 was passing test before but now failed with 「unknown」 error! Its run-log could be found in 「5UU8」
PDB 5UU9 was passing test before but now failed with 「fill_missing_atoms」 error! Its run-log could be found in 「5UU9」
PDB 5UUA was passing test before but now failed with 「unknown」 error! Its run-log could be found in 「5UUA」
PDB 5UUB was passing test before but now failed with 「fill_missing_atoms」 error! Its run-log could be found in 「5UUB」
PDB 5W26 was passing test before but now failed with 「fill_missing_atoms」 error! Its run-log could be found in 「5W26」
PDB 5W2U was passing test before but now failed with 「fill_missing_atoms」 error! Its run-log could be found in 「5W2U」
PDB 5W2W was passing test before but now failed with 「fill_missing_atoms」 error! Its run-log could be found in 「5W2W」
PDB 5W2Y was passing test before but now failed with 「fill_missing_atoms」 error! Its run-log could be found in 「5W2Y」
PDB 5WD7 was passing test before but now failed with 「unrecognized_residue」 error! Its run-log could be found in 「5WD7」
PDB 5WV1 was passing test before but now failed with 「unknown」 error! Its run-log could be found in 「5WV1」
PDB 5X7Q was passing test before but now failed with 「fill_missing_atoms」 error! Its run-log could be found in 「5X7Q」
PDB 5X7R was passing test before but now failed with 「fill_missing_atoms」 error! Its run-log could be found in 「5X7R」
PDB 5X8B was passing test before but now failed with 「nu_conformer」 error! Its run-log could be found in 「5X8B」
PDB 5XOO was passing test before but now failed with 「fill_missing_atoms」 error! Its run-log could be found in 「5XOO」
PDB 5XP6 was passing test before but now failed with 「fill_missing_atoms」 error! Its run-log could be found in 「5XP6」
PDB 5XSJ was passing test before but now failed with 「fill_missing_atoms」 error! Its run-log could be found in 「5XSJ」
PDB 5XSS was passing test before but now failed with 「unknown」 error! Its run-log could be found in 「5XSS」
PDB 5Z5F was passing test before but now failed with 「unknown」 error! Its run-log could be found in 「5Z5F」
PDB 5Z5I was passing test before but now failed with 「unknown」 error! Its run-log could be found in 「5Z5I」
PDB 5ZDK was passing test before but now failed with 「bad_patch」 error! Its run-log could be found in 「5ZDK」
PDB 5ZDQ was passing test before but now failed with 「fill_missing_atoms」 error! Its run-log could be found in 「5ZDQ」
PDB 5ZDR was passing test before but now failed with 「fill_missing_atoms」 error! Its run-log could be found in 「5ZDR」
PDB 5ZF3 was passing test before but now failed with 「unknown」 error! Its run-log could be found in 「5ZF3」
PDB 5ZGE was passing test before but now failed with 「fill_missing_atoms」 error! Its run-log could be found in 「5ZGE」
PDB 5ZGF was passing test before but now failed with 「fill_missing_atoms」 error! Its run-log could be found in 「5ZGF」
PDB 5ZGI was passing test before but now failed with 「fill_missing_atoms」 error! Its run-log could be found in 「5ZGI」
PDB 5ZGQ was passing test before but now failed with 「fill_missing_atoms」 error! Its run-log could be found in 「5ZGQ」
PDB 5ZGT was passing test before but now failed with 「fill_missing_atoms」 error! Its run-log could be found in 「5ZGT」
PDB 5ZGU was passing test before but now failed with 「fill_missing_atoms」 error! Its run-log could be found in 「5ZGU」
PDB 5ZGV was passing test before but now failed with 「fill_missing_atoms」 error! Its run-log could be found in 「5ZGV」
PDB 5ZGW was passing test before but now failed with 「fill_missing_atoms」 error! Its run-log could be found in 「5ZGW」
PDB 5ZGX was passing test before but now failed with 「fill_missing_atoms」 error! Its run-log could be found in 「5ZGX」
PDB 5ZGY was passing test before but now failed with 「fill_missing_atoms」 error! Its run-log could be found in 「5ZGY」
PDB 5ZGZ was passing test before but now failed with 「fill_missing_atoms」 error! Its run-log could be found in 「5ZGZ」
PDB 5ZH1 was passing test before but now failed with 「fill_missing_atoms」 error! Its run-log could be found in 「5ZH1」
PDB 5ZII was passing test before but now failed with 「unknown」 error! Its run-log could be found in 「5ZII」
PDB 5ZKZ was passing test before but now failed with 「unknown」 error! Its run-log could be found in 「5ZKZ」
PDB 5ZQS was passing test before but now failed with 「unknown」 error! Its run-log could be found in 「5ZQS」
PDB 5ZQX was passing test before but now failed with 「unknown」 error! Its run-log could be found in 「5ZQX」
PDB 6A89 was passing test before but now failed with 「unknown」 error! Its run-log could be found in 「6A89」
PDB 6A8H was passing test before but now failed with 「unknown」 error! Its run-log could be found in 「6A8H」
PDB 6A8I was passing test before but now failed with 「unknown」 error! Its run-log could be found in 「6A8I」
PDB 6ALS was passing test before but now failed with 「unrecognized_residue」 error! Its run-log could be found in 「6ALS」
PDB 6ALT was passing test before but now failed with 「unrecognized_residue」 error! Its run-log could be found in 「6ALT」
PDB 6ALU was passing test before but now failed with 「unrecognized_residue」 error! Its run-log could be found in 「6ALU」
PDB 6AQ5 was passing test before but now failed with 「fill_missing_atoms」 error! Its run-log could be found in 「6AQ5」
PDB 6AQ6 was passing test before but now failed with 「fill_missing_atoms」 error! Its run-log could be found in 「6AQ6」
PDB 6AVL was passing test before but now failed with 「fill_missing_atoms」 error! Its run-log could be found in 「6AVL」
PDB 6B6O was passing test before but now failed with 「fill_missing_atoms」 error! Its run-log could be found in 「6B6O」
PDB 6B6P was passing test before but now failed with 「fill_missing_atoms」 error! Its run-log could be found in 「6B6P」
PDB 6B6Q was passing test before but now failed with 「fill_missing_atoms」 error! Its run-log could be found in 「6B6Q」
PDB 6CJV was passing test before but now failed with 「unrecognized_residue」 error! Its run-log could be found in 「6CJV」
PDB 6D5N was passing test before but now failed with 「unknown」 error! Its run-log could be found in 「6D5N」
PDB 6D5P was passing test before but now failed with 「unknown」 error! Its run-log could be found in 「6D5P」
PDB 6D5Q was passing test before but now failed with 「unknown」 error! Its run-log could be found in 「6D5Q」
PDB 6D5R was passing test before but now failed with 「unknown」 error! Its run-log could be found in 「6D5R」
PDB 6DT1 was passing test before but now failed with 「missing_bond」 error! Its run-log could be found in 「6DT1」
PDB 6EGW was passing test before but now failed with 「unknown」 error! Its run-log could be found in 「6EGW」
PDB 6EQ1 was passing test before but now failed with 「unrecognized_residue」 error! Its run-log could be found in 「6EQ1」
PDB 6EUF was passing test before but now failed with 「fill_missing_atoms」 error! Its run-log could be found in 「6EUF」
PDB 6F1G was passing test before but now failed with 「unknown」 error! Its run-log could be found in 「6F1G」
PDB 6F4V was passing test before but now failed with 「unrecognized_residue」 error! Its run-log could be found in 「6F4V」
PDB 6F5K was passing test before but now failed with 「fill_missing_atoms」 error! Its run-log could be found in 「6F5K」
PDB 6G09 was passing test before but now failed with 「unknown」 error! Its run-log could be found in 「6G09」
PDB 6G0B was passing test before but now failed with 「unknown」 error! Its run-log could be found in 「6G0B」
PDB 6G0N was passing test before but now failed with 「unknown」 error! Its run-log could be found in 「6G0N」
PDB 6G48 was passing test before but now failed with 「fill_missing_atoms」 error! Its run-log could be found in 「6G48」
PDB 6GCC was passing test before but now failed with 「unrecognized_residue」 error! Its run-log could be found in 「6GCC」
PDB 6HBM was passing test before but now failed with 「unknown」 error! Its run-log could be found in 「6HBM」

PDB 6I9Y was passing test before but now failed with 「fill_missing_atoms」 error! Its run-log could be found in 「6I9Y」
PDB 6IDN was passing test before but now failed with 「fill_missing_atoms」 error! Its run-log could be found in 「6IDN」
PDB 6IG0 was passing test before but now failed with 「bad_patch」 error! Its run-log could be found in 「6IG0」
PDB 6ILD was passing test before but now failed with 「unknown」 error! Its run-log could be found in 「6ILD」
PDB 6ILS was passing test before but now failed with 「fill_missing_atoms」 error! Its run-log could be found in 「6ILS」
PDB 6NKN was passing test before but now failed with 「fill_missing_atoms」 error! Its run-log could be found in 「6NKN」
PDB 7AT1 was passing test before but now failed with 「fill_missing_atoms」 error! Its run-log could be found in 「7AT1」
PDB 8AT1 was passing test before but now failed with 「fill_missing_atoms」 error! Its run-log could be found in 「8AT1」
PDB 8CGT was passing test before but now failed with 「unrecognized_residue」 error! Its run-log could be found in 「8CGT」
PDB 9CGT was passing test before but now failed with 「unrecognized_residue」 error! Its run-log could be found in 「9CGT」

NOTE: 26550 PDB's passed the tests but was not listed in reference results.

To update reference results please copy the files below into the main repository:
reference-results.full.new.json → 「main repository」 as tests/benchmark/tests/scientific/protein_data_bank_diagnostic/reference-results.full.json
blocklist.full.new.json → 「main repository」 as tests/benchmark/tests/scientific/protein_data_bank_diagnostic/blocklist.full.json

REVISION

revision:61592

test_id: 687995

status: passed

Scientific test: relax_cartesian

FAILURES

None

RESULTS

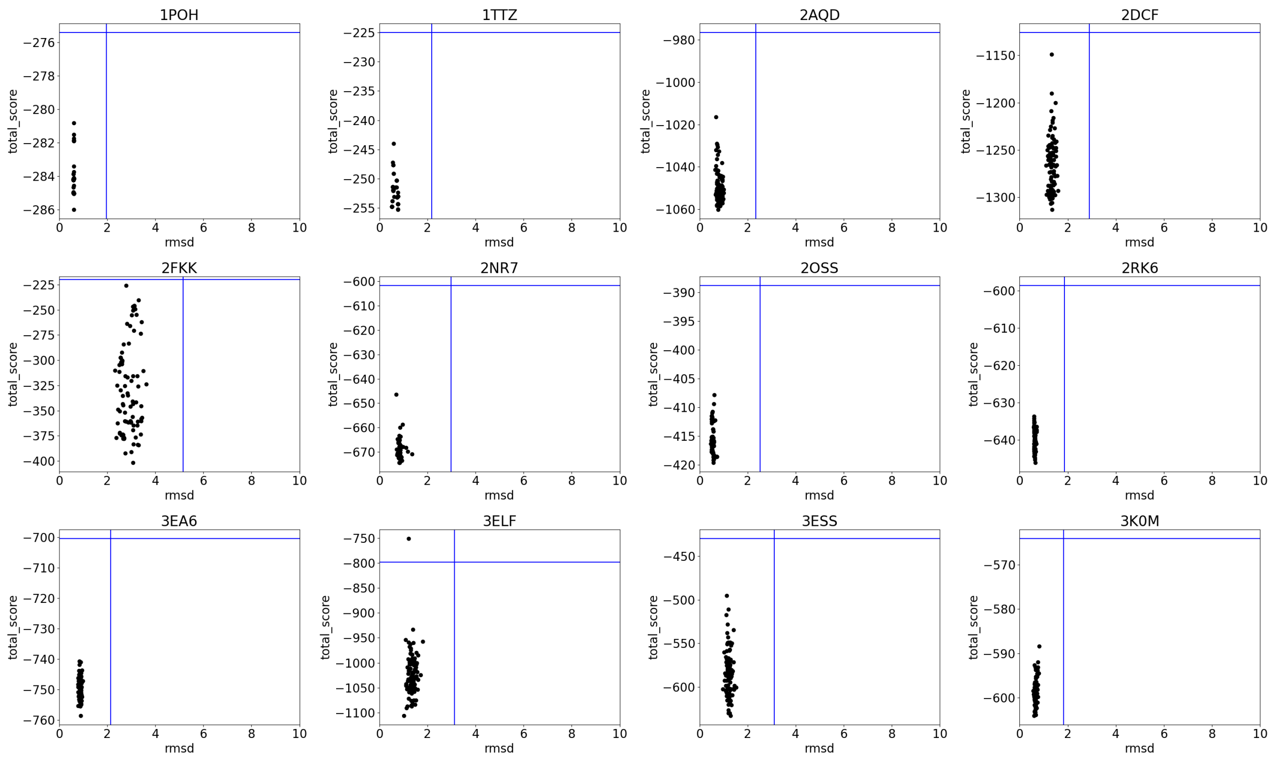

AUTHOR AND DATE

Julia Koehler Leman, julia.koehler.leman@gmail.com, Richard Bonneau, Oct 2018

PURPOSE OF THE TEST

This benchmark tests different versions of the relax protocol (fast_relax with 1 iteration (basic), 5 iterations (default) and cart_relax) to see what RMSD and score ranges are sampled with different protocols and how close the decoys stay to the native structure.

BENCHMARK DATASET

There are 12 proteins in the benchmark set that are taken from (Conway, P., Tyka, M. D., DiMaio, F., Konerding, D. E. & Baker, D. Relaxation of backbone bond geometry improves protein energy landscape modeling. Protein Sci. 23, 47â€“55 (2014).). These are single-chain proteins ranging from 75 to 384 residues. The input files are cleaned PDBs.

PROTOCOL

This is running RosettaScripts with the FastRelax mover, then superimposes the decoys to the native and computes RMSDs via SimpleMetrics. [It is running RosettaScripts because the fast_relax application wouldn't compute RMSDs, even with the native flag.] We use the ref2015 high-res scorefunction and superimpose on the backbone heavy atoms. 100 decoys are created for each protein in the set. Each relax protocol takes about 120 CPU hours for the entire set, which makes this 360 CPU hours for all three protocols (fast_relax with 1 iteration (basic), 5 iterations (default) and cart_relax) for the entire benchmark set.

PERFORMANCE METRICS

We use the total Rosetta score and RMSD to the native structure as we are interested in the sampling range and the score ranges in sampling. A passed test constitutes 90% of the decoys generated have a smaller than the defined score and RMSD cutoffs. The cutoffs were defined by looking at these ranges in a single run without outlier decoys. Specifically, for RMSD we use the maximum RMSD + stdev + 1A. The +1A is to account for the very narrow funnels with small stdevs. For score, we use the maximum score + stdev.

KEY RESULTS

Typically, we see deep funnels (vertical score-vs-RMSD plots) for most proteins, with sampling ranges around 1-2A RMSD and scores in the -200 to -1200 ranges. 5 iterations of fastrelax moves the decoys away from the native a little bit more than a single iteration and have slightly lower scores, both of which are expected. Cartrelax gets lower scores than even 5 iterations of fastrelax, sometimes significantly lower scores. The RMSD range for cartrelax is similar to fastrelax, except for the outliers, where cartrelax samples a more narrow range. Outliers are (1) 2DCF which has a chainbreak and a flexible N-terminus, sampling RMSDs up to 4A in fastrelax, up to 8A in fastrelax 5 iterations, but samples a normal narrow range in cartrelax <2A; (2) 2FKK has a chainbreak and a very long disordered region (~100 residues towards the C-terminus). Sampling ranges are up to 10A for fastrelax (1 or 5 iterations), but <4A in cartrelax.

DEFINITIONS AND COMMENTS

LIMITATIONS

The dataset is somewhat diverse and realistic in terms of protein size, alpha/beta content, and loop content. However, it is unclear how relax performs on multi-chain and symmetric proteins. Further, I am unclear whether this is the latest state-of-the-art Rosetta protocol to use.

REVISION

revision:61592

test_id: 687736

status: passed

Scientific test: relax_fast_5iter

FAILURES

None

RESULTS

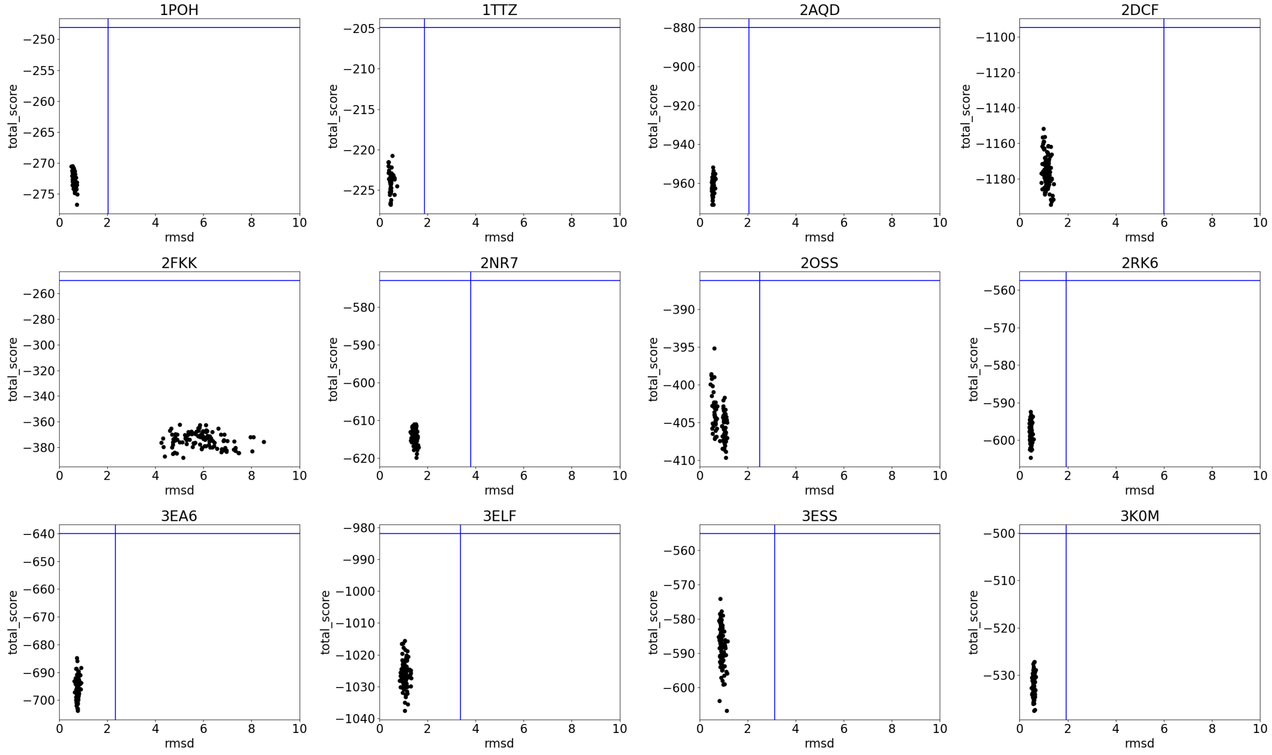

AUTHOR AND DATE

Julia Koehler Leman, julia.koehler.leman@gmail.com, Richard Bonneau, Oct 2018

PURPOSE OF THE TEST

This benchmark tests different versions of the relax protocol (fast_relax with 1 iteration (basic), 5 iterations (default) and cart_relax) to see what RMSD and score ranges are sampled with different protocols and how close the decoys stay to the native structure.

BENCHMARK DATASET

There are 12 proteins in the benchmark set that are taken from (Conway, P., Tyka, M. D., DiMaio, F., Konerding, D. E. & Baker, D. Relaxation of backbone bond geometry improves protein energy landscape modeling. Protein Sci. 23, 47â€“55 (2014).). These are single-chain proteins ranging from 75 to 384 residues. The input files are cleaned PDBs.

PROTOCOL

This is running RosettaScripts with the FastRelax mover, then superimposes the decoys to the native and computes RMSDs via SimpleMetrics. [It is running RosettaScripts because the fast_relax application wouldn't compute RMSDs, even with the native flag.] We use the ref2015 high-res scorefunction and superimpose on the backbone heavy atoms. 100 decoys are created for each protein in the set. Each relax protocol takes about 120 CPU hours for the entire set, which makes this 360 CPU hours for all three protocols (fast_relax with 1 iteration (basic), 5 iterations (default) and cart_relax) for the entire benchmark set.

PERFORMANCE METRICS

We use the total Rosetta score and RMSD to the native structure as we are interested in the sampling range and the score ranges in sampling. A passed test constitutes 90% of the decoys generated have a smaller than the defined score and RMSD cutoffs. The cutoffs were defined by looking at these ranges in a single run without outlier decoys. Specifically, for RMSD we use the maximum RMSD + stdev + 1A. The +1A is to account for the very narrow funnels with small stdevs. For score, we use the maximum score + stdev.

KEY RESULTS

Typically, we see deep funnels (vertical score-vs-RMSD plots) for most proteins, with sampling ranges around 1-2A RMSD and scores in the -200 to -1200 ranges. 5 iterations of fastrelax moves the decoys away from the native a little bit more than a single iteration and have slightly lower scores, both of which are expected. Cartrelax gets lower scores than even 5 iterations of fastrelax, sometimes significantly lower scores. The RMSD range for cartrelax is similar to fastrelax, except for the outliers, where cartrelax samples a more narrow range. Outliers are (1) 2DCF which has a chainbreak and a flexible N-terminus, sampling RMSDs up to 4A in fastrelax, up to 8A in fastrelax 5 iterations, but samples a normal narrow range in cartrelax <2A; (2) 2FKK has a chainbreak and a very long disordered region (~100 residues towards the C-terminus). Sampling ranges are up to 10A for fastrelax (1 or 5 iterations), but <4A in cartrelax.

DEFINITIONS AND COMMENTS

LIMITATIONS

The dataset is somewhat diverse and realistic in terms of protein size, alpha/beta content, and loop content. However, it is unclear how relax performs on multi-chain and symmetric proteins. Further, I am unclear whether this is the latest state-of-the-art Rosetta protocol to use.

REVISION

revision:61592

test_id: 687688

status: passed

Scientific test: relax_fast

FAILURES

None

RESULTS

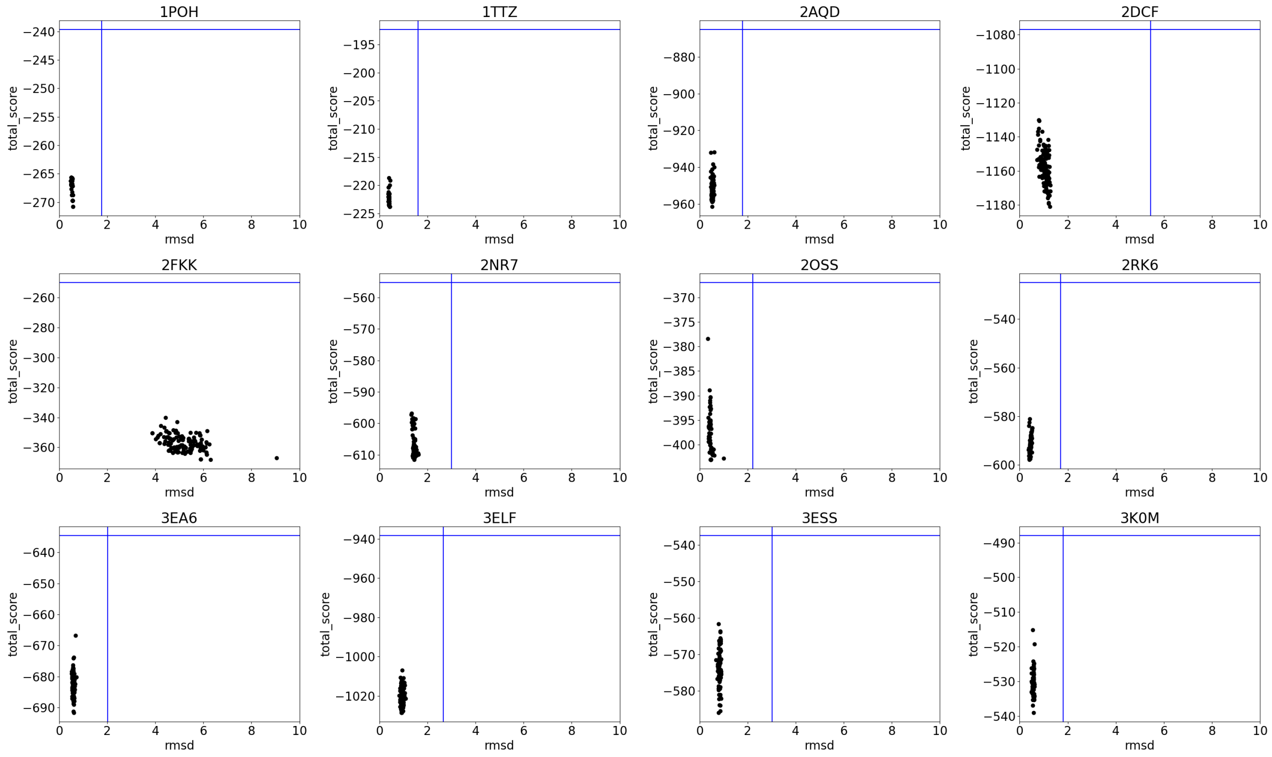

AUTHOR AND DATE

Julia Koehler Leman, julia.koehler.leman@gmail.com, Richard Bonneau, Oct 2018

PURPOSE OF THE TEST

This benchmark tests different versions of the relax protocol (fast_relax with 1 iteration (basic), 5 iterations (default) and cart_relax) to see what RMSD and score ranges are sampled with different protocols and how close the decoys stay to the native structure.

BENCHMARK DATASET

There are 12 proteins in the benchmark set that are taken from (Conway, P., Tyka, M. D., DiMaio, F., Konerding, D. E. & Baker, D. Relaxation of backbone bond geometry improves protein energy landscape modeling. Protein Sci. 23, 47â€“55 (2014).). These are single-chain proteins ranging from 75 to 384 residues. The input files are cleaned PDBs.

PROTOCOL

This is running RosettaScripts with the FastRelax mover, then superimposes the decoys to the native and computes RMSDs via SimpleMetrics. [It is running RosettaScripts because the fast_relax application wouldn't compute RMSDs, even with the native flag.] We use the ref2015 high-res scorefunction and superimpose on the backbone heavy atoms. 100 decoys are created for each protein in the set. Each relax protocol takes about 120 CPU hours for the entire set, which makes this 360 CPU hours for all three protocols (fast_relax with 1 iteration (basic), 5 iterations (default) and cart_relax) for the entire benchmark set.

PERFORMANCE METRICS

We use the total Rosetta score and RMSD to the native structure as we are interested in the sampling range and the score ranges in sampling. A passed test constitutes 90% of the decoys generated have a smaller than the defined score and RMSD cutoffs. The cutoffs were defined by looking at these ranges in a single run without outlier decoys. Specifically, for RMSD we use the maximum RMSD + stdev + 1A. The +1A is to account for the very narrow funnels with small stdevs. For score, we use the maximum score + stdev.

KEY RESULTS

Typically, we see deep funnels (vertical score-vs-RMSD plots) for most proteins, with sampling ranges around 1-2A RMSD and scores in the -200 to -1200 ranges. 5 iterations of fastrelax moves the decoys away from the native a little bit more than a single iteration and have slightly lower scores, both of which are expected. Cartrelax gets lower scores than even 5 iterations of fastrelax, sometimes significantly lower scores. The RMSD range for cartrelax is similar to fastrelax, except for the outliers, where cartrelax samples a more narrow range. Outliers are (1) 2DCF which has a chainbreak and a flexible N-terminus, sampling RMSDs up to 4A in fastrelax, up to 8A in fastrelax 5 iterations, but samples a normal narrow range in cartrelax <2A; (2) 2FKK has a chainbreak and a very long disordered region (~100 residues towards the C-terminus). Sampling ranges are up to 10A for fastrelax (1 or 5 iterations), but <4A in cartrelax.

DEFINITIONS AND COMMENTS

LIMITATIONS

The dataset is somewhat diverse and realistic in terms of protein size, alpha/beta content, and loop content. However, it is unclear how relax performs on multi-chain and symmetric proteins. Further, I am unclear whether this is the latest state-of-the-art Rosetta protocol to use.

REVISION

revision:61588

test_id: 686988

status: passed

Scientific test: rna_denovo_favorites

FAILURES

None

RESULTS

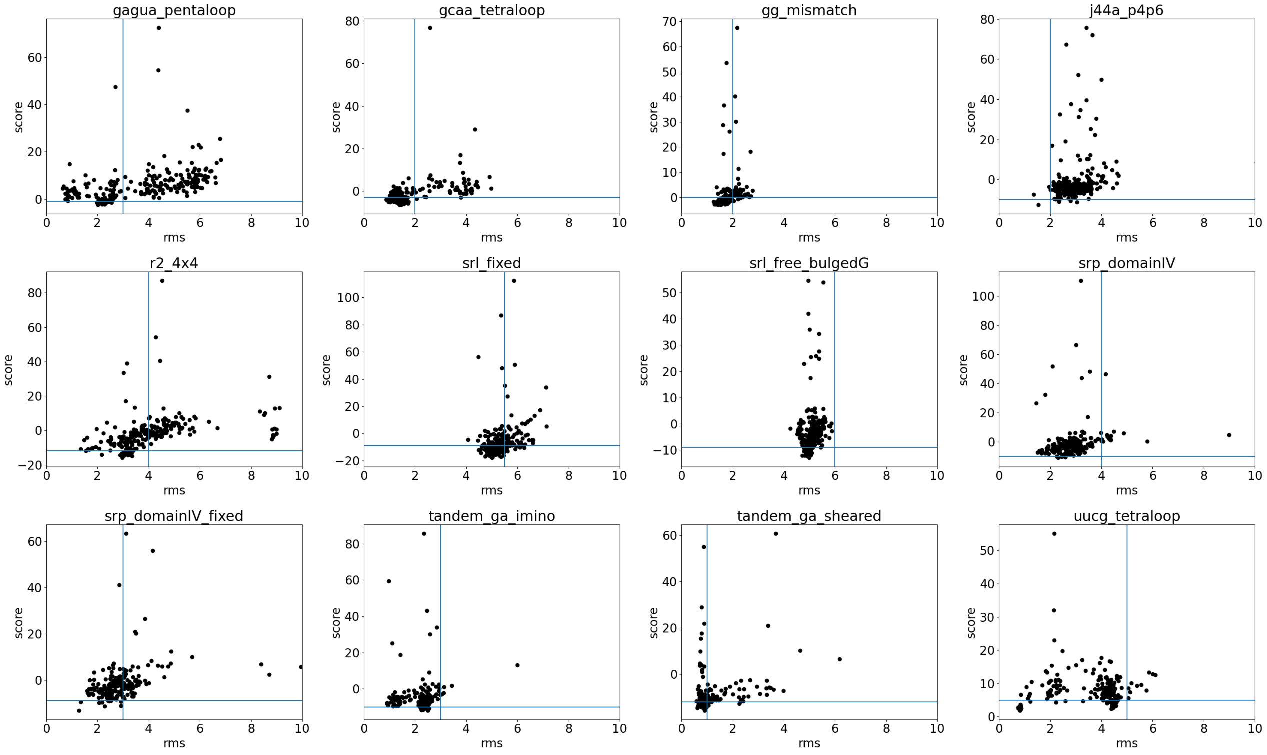

AUTHOR AND DATE

Andrew Watkins (andy.watkins2@gmail.com); Rhiju Das (October 2018)

PURPOSE OF THE TEST

This test assesses the stability of the FARFAR algorithm's performance. FARFAR stands for Fragment Assembly of RNA with Full-Atom Refinement; it is a mirror of the protein 'ab initio' application but it is also well equipped for homology modeling using fragments of input structure.

BENCHMARK DATASET

The benchmark set contains the 12 loop modeling problems from favorites.txt, a benchmark first established in Watkins et al., Science Advances 2018. The input files are ideal A-form RNA helices and fasta files; the FARFAR algorithm takes these input pieces of PDB structure and attempts to predict the remaining residues defined by the FASTA file; 'correctness' is defined as similarity to portions of crystal structures.

PROTOCOL

See Das et al., Nat. Methods 2010 for a description of the FARFAR protocol. The benchmark takes 6 hours on the test server (~120 CPU-hours).

PERFORMANCE METRICS

Benchmark performance is based on the minimum RMSD sampled and minimum energy sampled; each case has a separate threshold. Since sampling is limited for each test, cutoffs are determined conservatively through repeated runs. A superior measure, at least in theory, might be to compute pnear and compare it to some threshold value.

KEY RESULTS

Because of the high-energy outliers endemic to the fragment assembly method, real "funnel-like" energy surfaces won't be visible for this test. That said, the lowest energy overall should lie to the left of the RMSD threshold and below the scoring threshold for each test;

stochastic failures where a low-energy model or two appear slightly past the RMSD threshold should be unsurprising, but a failure to return sufficiently low-energy models at all should be viewed with suspicion.

DEFINITIONS AND COMMENTS

n/a

LIMITATIONS

The benchmark could be expanded to include favorites2.txt, challenges.txt, followups.txt, and more of the benchmarking challenges already developed for stepwise Monte Carlo. These files are located in the publicly available Github repo DasLab/rna_benchmark. Furthermore, the protocol could also target the RNA-Puzzles dataset (the equivalent of CASP), where we have pretty predictable performance characteristics as long as we can generate a few thousand models. More CPU power would permit better sampling and therefore more precise cutoffs.

REVISION

revision:61587

test_id: 686806

status: passed

Scientific test: RosettaCM

FAILURES

None

RESULTS

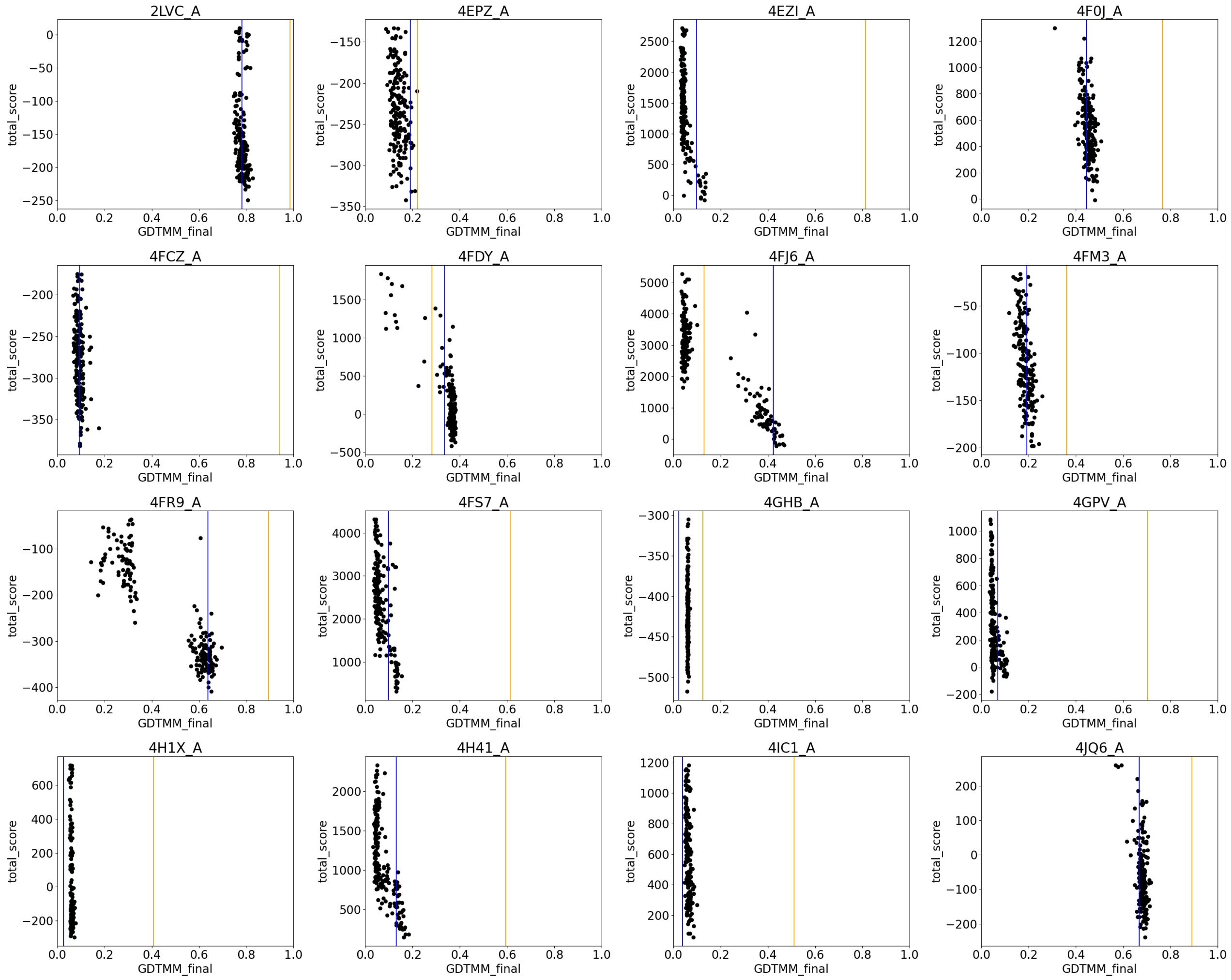

AUTHOR AND DATE

Adapted for current benchmark framework by Jason Fell (jsfell@ucdavis.edu; Siegel Lab @ UC Davis), April 2020

PURPOSE OF THE TEST

This benchmark is meant to test the current performance of generating homology models with the RosettaCM protocol.

(Song, Y.; DiMaio, F.; Wang, R.Y.R.; Kim, D.; Miles, C.; Brunette, T.; et al. High-resolution comparative modeling with RosettaCM. Structure. 2013. 21:1735-1742.)

BENCHMARK DATASET

The dataset currently consists of 16 of 68 targets from the CASP10 (http://predictioncenter.org/casp10/), and were used in the original

RosettaCM paper. These 16 targets had pdb codes listed in RosettaCM paper, as well as have a variety of different sizes, folds, and fold complexities. The entire set contains several multi-domain proteins, we did not take them here. Each target uses a set of template structures, which were listed in the original RosettaCM article (Structure 2013), that are used to generate the homology models.

The targets used (by pdb code): 4EPZ 4H41 4JQ6 4FDY 4GHB 4FMZ 4GOQ 4FM3 4FJ6 4FGM 4EZI 4FR9 4AK1 4HES 2LVC 4FCZ

PROTOCOL

The Rosetta wiki describes the RosettaCM protocol (https://www.rosettacommons.org/docs/latest/application_documentation/structure_prediction/RosettaCM)

To run the RosettaCM protocol the required input files are:

*target fasta sequence

*threaded models

*hybridize.xml

The hybridize.xml runs a hybridize mover that samples structural combinations of multiple input models. The mover runs in three stages:

-Stage 1 is a stocastic procedure to generate a global superposition of aligned poritons of target and templates, which uses a centroid scoring method (score3).

-Stage 2 improves model geometry and explore conformational changes through a Monte Carlo sampling with two-step moves, and is scored by a centroid energy function

with smooth reparameterizations (score4_smooth_cart)

-Stage 3 side chains are built and optimized by Rosetta Monte Carlo sampling and is scored with a Rosetta full-atom energy function (ref2015_cart)

After the hybridize mover is called, Rosetta then performs a relax mover on the model (FastRelax; https://www.rosettacommons.org/docs/latest/application_documentation/structure_prediction/relax),

which is scored with the Stage 3 full-atom energy function (talaris2013_cart).

Once all of these files are ready run the command:

Rosetta/main/source/bin/rosetta_scripts.linuxclangrelease \

-database Rosetta/main/database \

-in:file:fasta target.fasta \

-parser:protocol hybridize.xml \

-default_max_cycles 200 \

-dualspace

-in:file:native (x-stal structure pdb if running test)

As a current estimate, generating 200 models for each target requires ~ 24 hours. 16 targets x 24 = ~ 384 CPU hours.

PERFORMANCE METRICS

The metric to use is the global distance test (GDT; Zemla, A., Venclovas, C., Moult, J., and Fidelis, K. (1999). Processing and analysis of CASP3 protein structure predictions.

Proteins (Suppl 3), 22-29.) to compare models to their respective crystal stucture. This test is meant to measure how well Rosetta can generate acurate homology models and tests how well the top target GDT changes compared to the original measures.

GDT values in orange are the top GDT obtained from the original RosettaCM paper (Structure 2013) - see Table in the Supplement. These values were generated from the average of the lowest 10% of energy models from several thousands, using an older scorefunction (likely talaris13). Here, we define the cutoffs as the top GDT values from the initial run, minus 0.05. If no model is generated above this cutoff, the test fails. We don't use the stdev here as it is meaningless with GDT-MM as a quality measure because there are many models created with all kinds of GDTs. Only the top GDT is meaningful to define model quality.

KEY RESULTS

This test is meant to measure changes in the overall performance of RosettaCM. As noted earlier, GDT was used as a measure of model accuracy. The original paper also

utilized alignment constraints generated by Robetta and used Fragment picker to generate fragments (a very robost method), where as this test uses the basic RosettaCM

protocol to generate models. Therefore the GDT's generated from this test may not perform as well as the original paper. This test can measure can be used as a means to

compare how well the curent protocol performs in relation to the more robust method.

DEFINITIONS AND COMMENTS

Potentially running these tests are time consuming, so more hours/time may be required.

LIMITATIONS

Independent of this protocl there are additional methods that can improve RosettaCM (i.e. to include additional evolutionary and catalytic constraints) which are not

currently standard for this protocol. Therefor, RosettaCM could include these other constraints when applicable.

Lastly, multiple tests could be run using other scorefunctions.

REVISION

revision:61604

test_id: 690665

status: passed

Scientific test: sewing

FAILURES

None

RESULTS

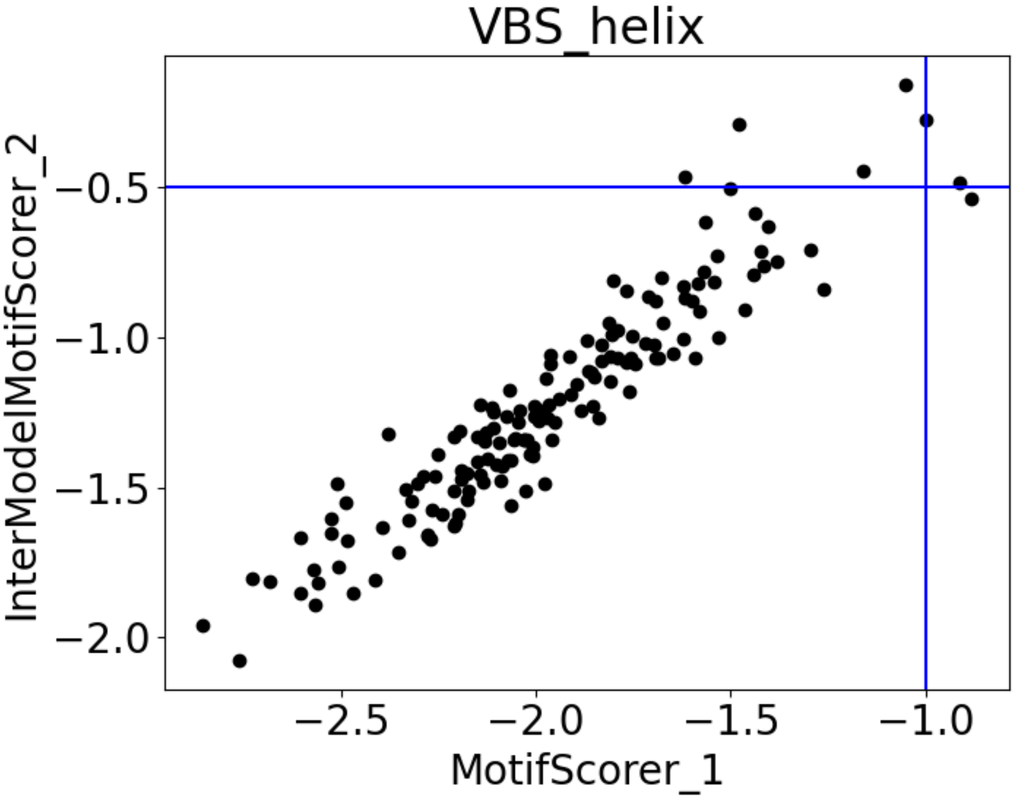

AUTHOR AND DATE

Frank Teets, teetsf@gmail.com, Brian Kuhlman, April 2019

PURPOSE OF THE TEST

This benchmark tests SEWING's capacity to explore restricted conformational spaces and still create long-range interactions; the restricted space magnifies the effect of poor sampling on score, making this the most sensitive test of SEWING's use cases. This tells us whether SEWING is sampling backbone additions with sufficient granularity to fit structure into confined spaces while still placing them in ways that approximate a globular protein.

BENCHMARK DATASET

There are two proteins in this set, vinculin (pdb 1T01, chain A) and the VBS1 helix of talin. (pdb 1T01, chain B) However, only one (VBS1) is actually used as the starting node for SEWING and consequently scored. The other serves as a SEWING "partner protein"; in normal use, this is something to which the starting node is intended to bind and therefore a region of space into which SEWING should not design structure, as well as serving as a target for encouraging the placement of backbone in regions likely to be conducive to expanding that interface via residue-level design. The requisite PartnerMotifScorer is not included in this test set, however; see PERFORMANCE METRICS below. The only other SEWING-specific input file is the segment file created as described in the paper from the TOP8000 dataset (Williams, C. J., Headd, J. J., Moriarty, N. W., Prisant, M. G., Videau, L. L., Deis, L. N., Verma, V. , Keedy, D. A., Hintze, B. J., Chen, V. B., Jain, S. , Lewis, S. M., Arendall, W. B., Snoeyink, J. , Adams, P. D., Lovell, S. C., Richardson, J. S. and Richardson, D. C. (2018), MolProbity: More and better reference data for improved all-atom structure validation. Protein Science, 27: 293-315. doi:10.1002/pro.3330)

PROTOCOL

The protocol is the AppendAssemblyMover protocol as described in (Protocols for Requirement-Driven Protein Design in the Rosetta Modeling Program (Guffy et al, 2018)) less the residue-level design step. It should run in 75 CPU hours.

PERFORMANCE METRICS

The performance metrics are the MotifScorer and InterModelMotifScorer. MotifScore measures whether the added structure is designable, and IMMS determines if it's globular. MotifScore represents the sum total of the highest possible full-atom attractive energy for any two small hydrophobic residues placed at every pair of residue positions within 6A of each other, normalized by the length of the design. This serves as a quickly evaluable metric of how well residue-level design can theoretically perform in designing a core from these residue positions. InterModelMotifScore restricts its evaluation to pairs of residues separated by at least one helix, meaning that it excludes all interactions between residues on the same helical hairpin. This is used internally to bias sampling toward globular proteins rather than flat sheets of helices, the latter maximizing the available volume for adding new structure. It also serves as a metric of globularity, and particularly of whether a given backbone has enough high-contact-order interactions for residue-level design to stabilize it in the designed configuration.

The cutoffs are defined as MotifScorer < -1.0/residue and InterModelMotifScorer < -0.5/residue, evaluated for the top 10% of outputs; the cutoffs are specific to this test case, and come from the paper. MotifScore is always numerically less than InterModelMotifScore for a given design, since InterModelMotifScore runs the same score function on a subset of the residue pairs, but if MotifScore passes the threshold and InterModelMotifScore does not, that indicates that SEWING has failed to sample with sufficient granularity to produce a globular protein; if MotifScore itself fails to reach < -1.0/residue, that generally indicates that SEWING has either added nothing at all to the starting node or that its additions have resulted in an unstructured assembly of helices distant from each other in space. This generally happens in a small fraction of decoys for every design run, but if it happens consistently (enough that >90% of the runs fail the test) it usually indicates that SEWING has failed to sample the restricted space around the starting node sufficiently.

The PartnerMotifScorer has been excluded from this test as it is less predictive than the other two, since it implicitly assumes that double-sided design is possible and will therefore position helices irrespective of the amino acids at the interface. Internally it serves to bias SEWING toward backbones suitable for interface design, but as a metric, good PartnerMotifScore is not predictive of good interface in the same way that (InterModel)MotifScore predicts a designable core so the score value is not informative. Similarly, since the output of SEWING precedes residue-level design, it includes the native residues from the helices it adds, and these will almost certainly clash with each other. It is therefore not appropriate to evaluate the full-atom score of the design as produced by SEWING alone, for which reason only internal SEWING scores are included in the benchmark.

KEY RESULTS

As this is a protocol for design, the baseline is relative to the original SEWING described in (Design of structurally distinct proteins using strategies inspired by evolution. (Jacobs et al.,2016)). It is expected that a small proportion of runs will fail to add anything to the starting helix, producing a population of data points close to 0 in both scores. Outside of that population, MotifScore and InterModelMotifScore should correlate as indicated by the cutoffs, indicating that SEWING is both placing helices designably close to each other (which is measured by MotifScore) and that it is also doing so for helices not adjacent in sequence space (as measured by InterModelMotifScore.)

DEFINITIONS AND COMMENTS

Run with 1000 minimum and maximum cycles, 9 minimum and maximum segments, a hash window width of 4, and a MotifScore:InterModelMotifScore weight ratio of 1:10, as described in the paper.

LIMITATIONS

This protocol is intentionally agnostic to idiosyncrasies in the correlation between (InterModel)MotifScore and final full-atom score; a future, multistage-compatible SEWING could be better benchmarked by actually relaxing those structures that passed the post-SEWING filters.

REVISION

revision:61587

test_id: 686809

status: passed

Scientific test: simple_cycpep_predict

FAILURES

None.

RESULTS SUMMARY

Total samples = 808633
Computed PNear = 0.945738
Computed PNear to lowest E = 0.985805
Lowest energy = -8.99493 kcal/mol
RMSD of lowest energy = 0.27398 Angstroms
Lowest RMSD = 0.192774 Angstroms
Highest RMSD = 2.86978 Angstroms
Energy gap (minE>1.5A - minE) = 6.19675 kcal/mol

More than 230,000 samples? YES
PNear value over 0.94? YES
PNear value to lowest E over 0.97? YES
Lowest energy under 0.3 A RMSD? YES
Sampling below 0.25 A RMSD? YES
Sampling beyond 1.5 A RMSD? YES
Sampling beyond 2.6 A RMSD? YES
6+ kcal/mol energy gap? YES
OVERALL PASS? YES

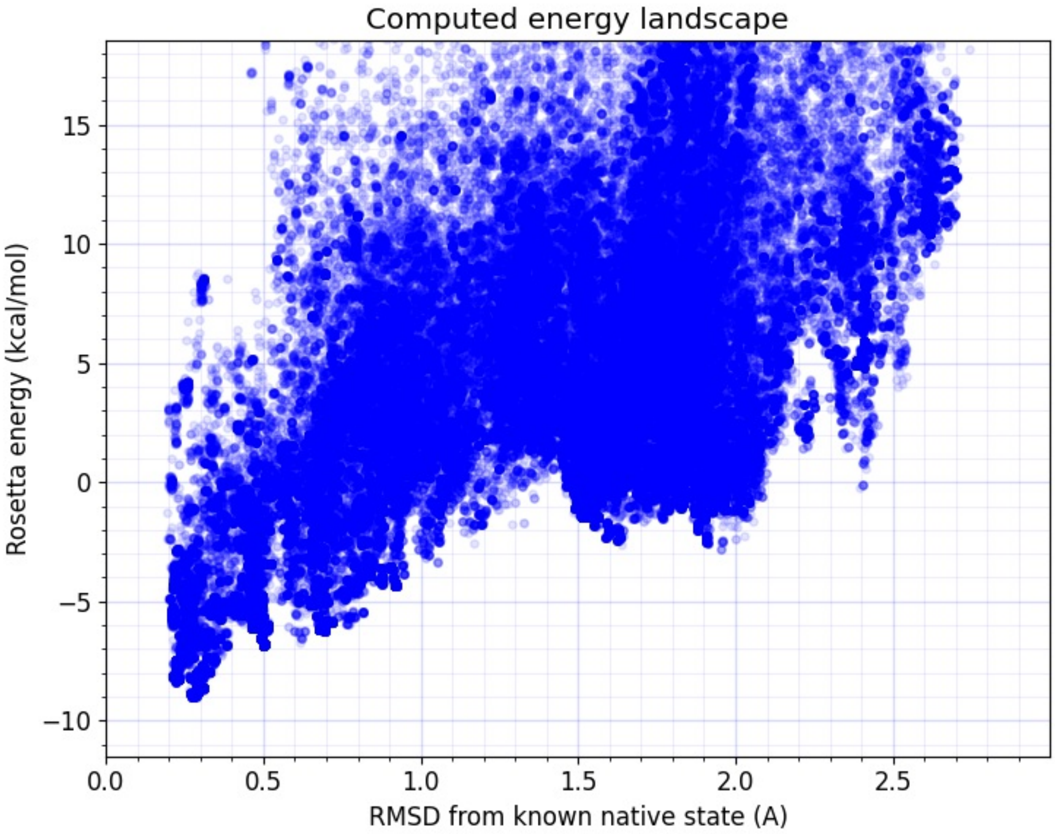

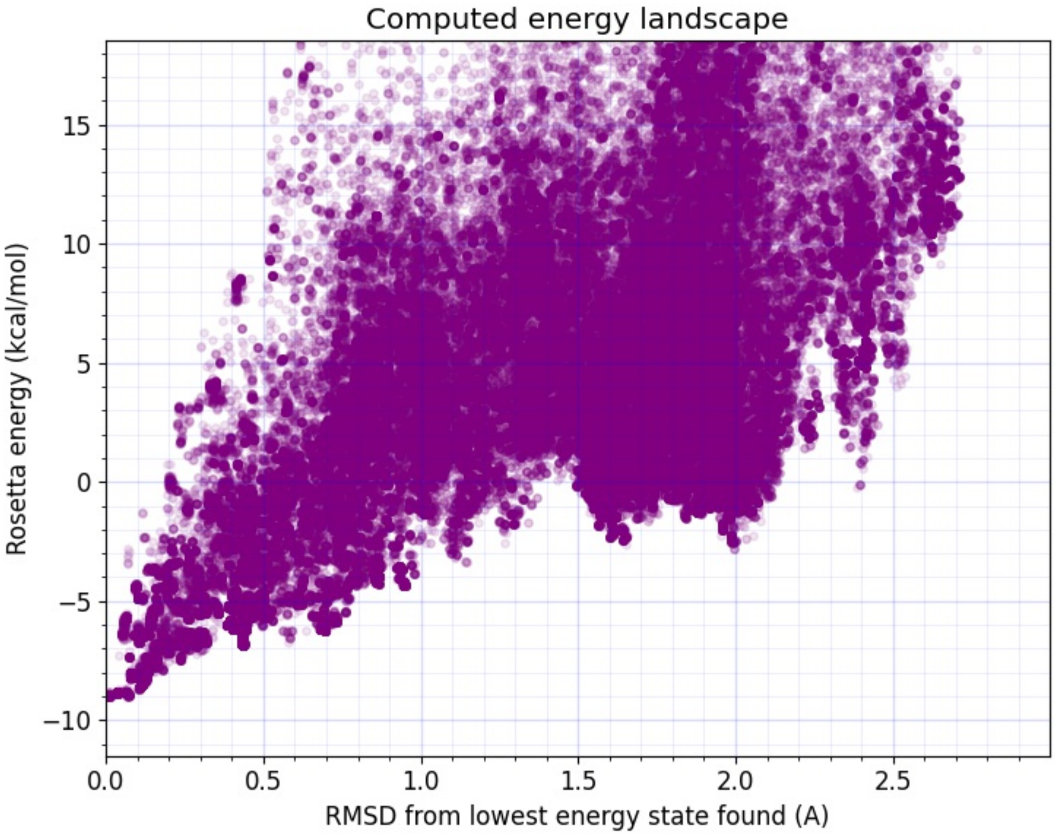

AUTHOR

Vikram K. Mulligan (vmulligan@flatironinstitute.org), Center for Computational Biology, Flatiron Institute, 1 March 2019

PURPOSE OF THE TEST

This test ensures that the simple_cycpep_predict application retains its scientific performance. This application is intended to predict the structures of peptide macrocycles built from arbitrary canonical or non-canonical building-blocks.

What does the benchmark test and why?

This test case is a peptide known to fold into the designed structure, for which multiple crystal structures have been solved. The application samples peptide conformations and attempts to predict the native state from sequence alone. The test is successful if:

- The application samples close to the native state.

- The lowest-energy sample is near native.

- The funnel quality metric PNear is greater than 0.9. (This metric ranges from 0.0, for a molecule that does not favour the designed state at all, to 1.0, for a molecule that spends all of its time in the designed state. It is calculated automatically from the sampled ensemble in MPI mode by the simple_cycpep_predict job distributor.)

BENCHMARK DATASET

How many proteins are in the set?

- One peptide, nicknamed "Moriarty".

What dataset are you using? Is it published? If yes, please add a citation.

- This peptide is currently unpublished.

What are the input files? How were the they created?

- The input is the peptide, designed with Rosetta, in PDB format, along with its sequence in ASCII text format.

PROTOCOL

State and briefly describe the protocol.

The simple_cycpep_predict application uses the generalized kinematic closure algorithm (GenKIC) to rapidly sample closed conformations of a heteropolymer macrocycle built from any combination of alpha-amino acids, peptoids, or other related building-blocks. Each closure attempt is relaxed using the FastRelax protocol. For small (~8 to ~10 residue) peptide macrocycles, the application can usually sample close to the native state with less expense than protein _ab initio_.

The simple_cycpep_predict application supports hierarchical MPI-based job distribution and data reduction, as well as multi-threaded parallel job execution within a node. In MPI mode, statistics about the full sampled ensemble, including the funnel quality metric PNear, are computed automatically during the data collection and reduction phase.

Is there a publication that describes the protocol?

The simple_cycpep_predict application is described in the following publications:

1. Bhardwaj G, Mulligan VK, Bahl CD, _et al._ (2016). Accurate de novo design of hyperstable constrained peptides. _Nature_ 538(7625):329-35.

2. Hosseinzadeh P, Bhardwaj G, Mulligan VK, _et al._ (2018). Comprehensive computational design of ordered peptide macrocycles. _Science_ 358(6369):1461-6.

How many CPU hours does this benchmark take approximately?

320 CPU-hours. The test runs on 4 nodes, 20 cores per node, for 4 wall hours. Note that job distribution ends after 2.5 hours, and results collection and analysis is expected to take less than an hour, so the actual cost is slightly lower.

In debug mode, this test takes 30 CPU-hours (1 node, 20 cores, for 1.5 wall hours). In this case, job distribution ends after 15 minutes, and results collection and analysis can take less than 75 minutes, so the actual cost is somewhat lower.

PERFORMANCE METRICS

What are the performance metrics used and why were they chosen?

All of the following must be true for the test to pass:

- More than 50,000 samples (1,500 in debug mode).

- Lowest-RMSD sample < 0.25 A from native.

- Highest-RMSD sample > 2.6 A from native.

- Lowest-energy sample < 0.3 A from native.

- Energy gap (gap between lowest-energy sample > 1.5 A and overall lowest) bigger than 6 kcal/mol

- PNear > 0.92.

How do you define a pass/fail for this test?

Failure of any of the above.

How were any cutoffs defined?

Arbitrarily, like so much else in Rosetta. These are based on the performance of Rosetta in predicting the crystal structure

of this peptide on 27 June 2019.

KEY RESULTS

What is the baseline to compare things to - experimental data or a previous Rosetta protocol?

Past iterations of this test.

Describe outliers in the dataset.

N/A.

DEFINITIONS AND COMMENTS

State anything you think is important for someone else to replicate your results.

N/A.

LIMITATIONS

What are the limitations of the benchmark? Consider dataset, quality measures, protocol etc.

We have very few known crystal structures of cyclic peptides.

How could the benchmark be improved?

More peptides. (We will add more in the future.)

What goals should be hit to make this a "good" benchmark?

REVISION

revision:61592

test_id: 687731

status: passed

Scientific test: stepwise_rna_favorites

FAILURES

None

RESULTS

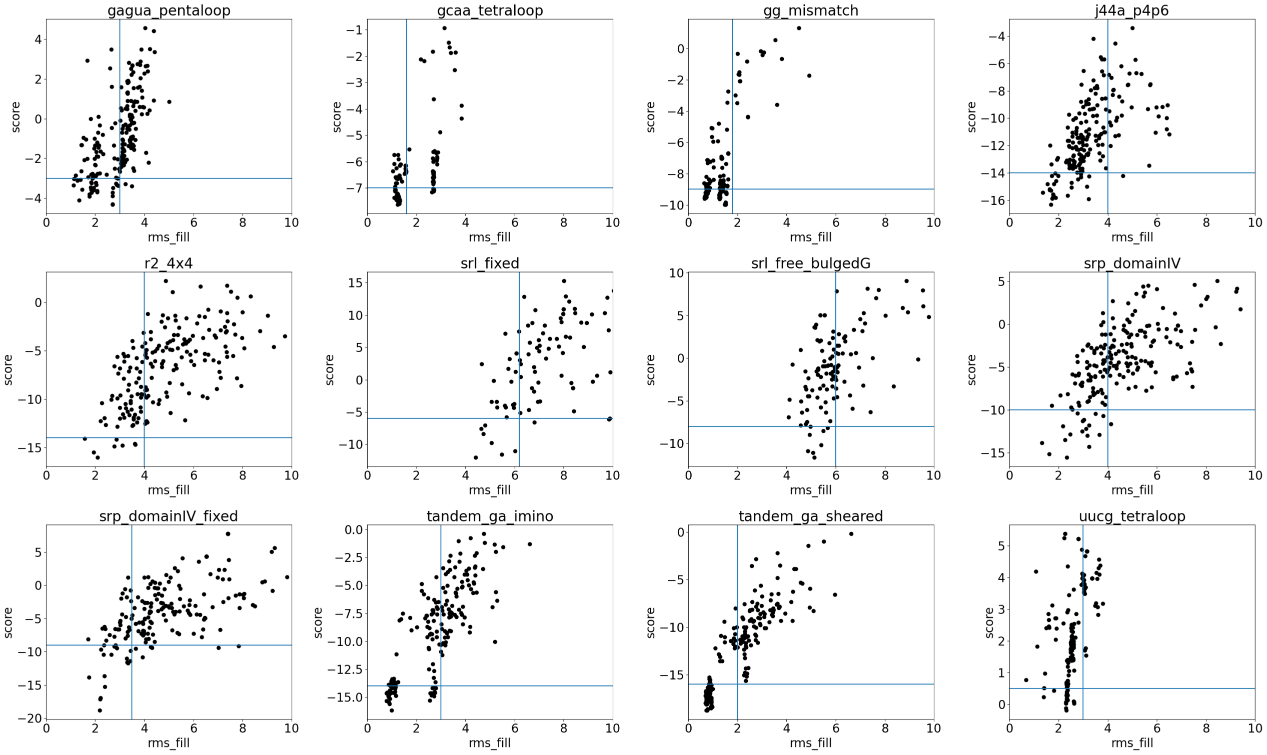

AUTHOR AND DATE

Andrew Watkins (andy.watkins2@gmail.com); Rhiju Das (October 2018)

PURPOSE OF THE TEST

This test judges the stability of the stepwise Monte Carlo algorithm's performance.

BENCHMARK DATASET

The benchmark set contains the 12 loop modeling problems from favorites.txt, a benchmark first established in Watkins et al., Science Advances 2018. The input files are ideal A-form RNA helices, fasta files, and portions of crystal structures.

PROTOCOL

See Watkins et al., Science Advances 2018 for a description of the SWM protocol. The benchmark takes 6 hours on the test server (~240 CPU-hours).

PERFORMANCE METRICS

Benchmark performance is based on the minimum RMSD sampled and minimum energy sampled; each case has a separate threshold. Since sampling is limited for each test, cutoffs are determined conservatively through repeated runs. Generally speaking, SWM should be able to achieve near-atomic accuracy in one of its top 5 lowest energy cluster centers if run for long enough on nearly every one of these cases; as is, we're just tracking some best-effort characteristics to see if the performance changes a *lot*.

KEY RESULTS

The majority of cases should display some strong funnel-like properties on the 'lower edge' of the score-versus-RMSD plot, with some outliers. That is, the lowest-energy structures for most models above the RMSD threshold should be much worse in energy than the lowest-energy structures for most models below the threshold. There are two expected "false minima": tandem_ga_imino should have a cluster of structures at about 2.9A RMSD; that cluster is sometimes slightly lower in energy than the near-native 0.9A RMSD cluster. Similarly, the gcaa_tetraloop should have a cluster just above the threshold that's only slightly worse in energy than the nativelike

cluster.

DEFINITIONS AND COMMENTS

n/a

LIMITATIONS

The benchmark could be expanded to include favorites2.txt, challenges.txt, followups.txt, and more of the benchmarking challenges already developed for stepwise Monte Carlo. More CPU power would permit better sampling and therefore more precise cutoffs.

REVISION

revision:61588

test_id: 686962

status: passed

Scientific test: Scorefunction comparison for FastDesign

RESULTS

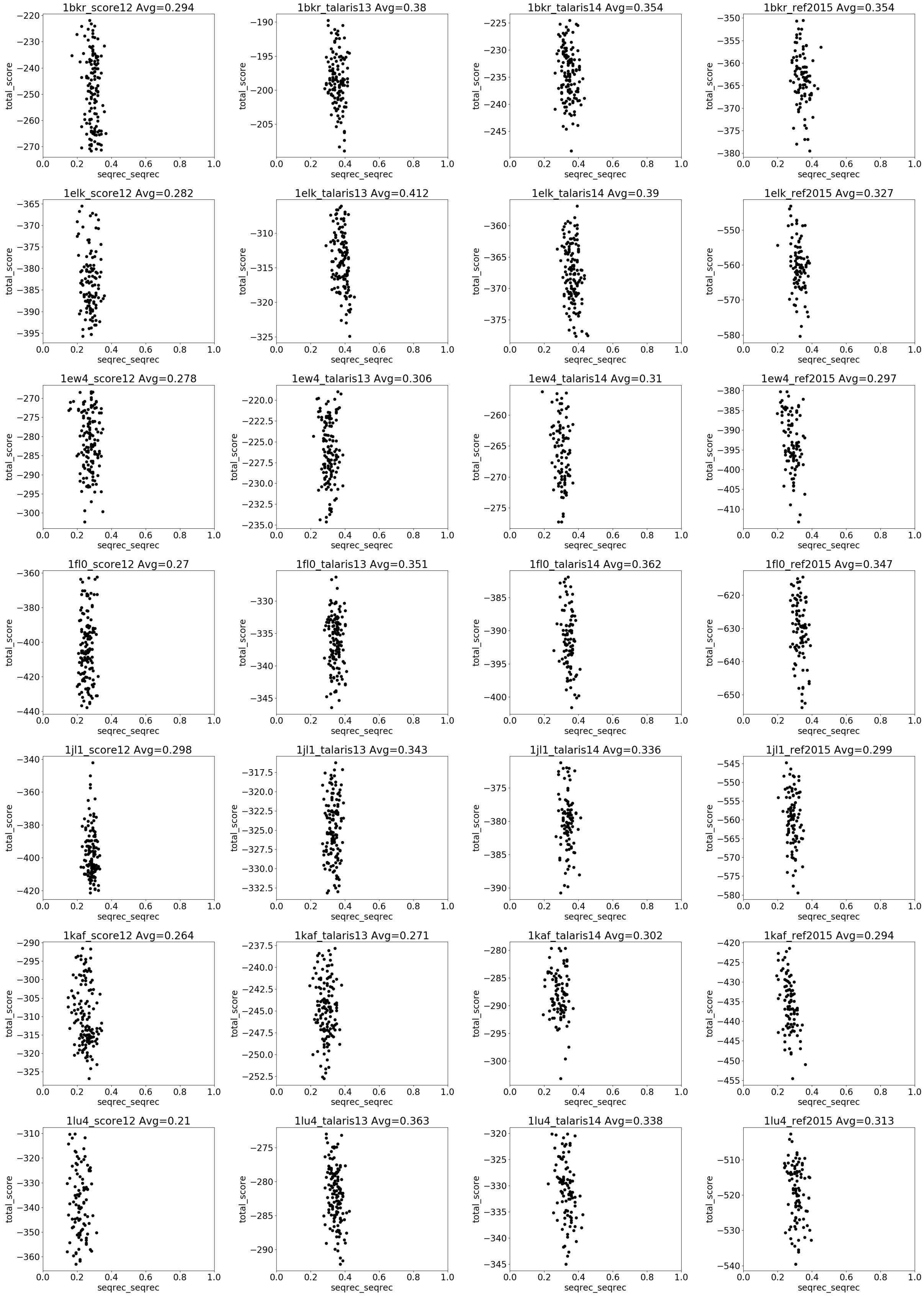

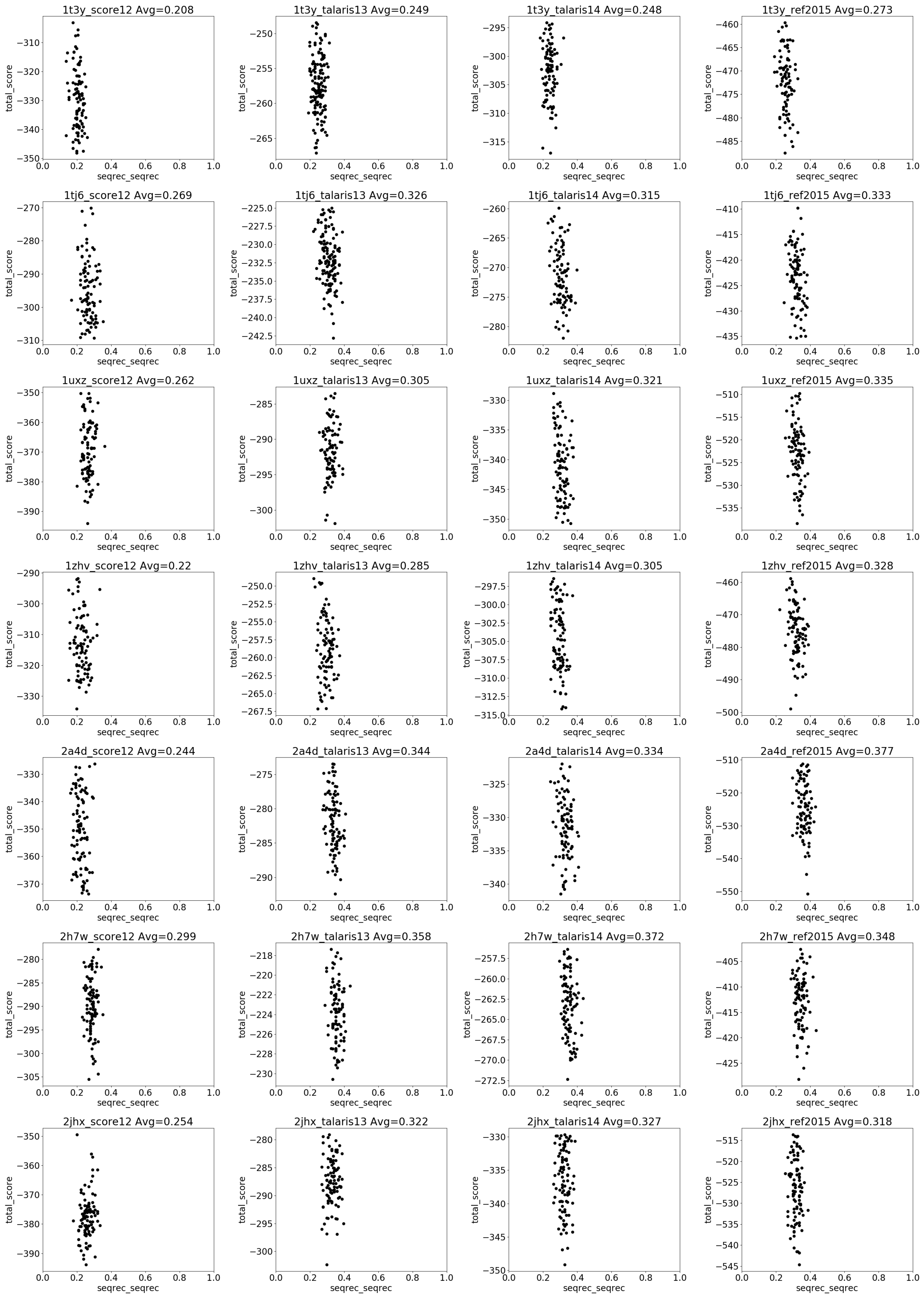
1t3y_score12 Avg=0.208
1t3y_talaris13 Avg=0.249
1t3y_talaris14 Avg=0.248
1t3y_ref2015 Avg=0.273
1tj6_score12 Avg=0.269
1tj6_talaris13 Avg=0.326
1tj6_talaris14 Avg=0.315
1tj6_ref2015 Avg=0.333
1uxz_score12 Avg=0.262
1uxz_talaris13 Avg=0.305
1uxz_talaris14 Avg=0.321
1uxz_ref2015 Avg=0.335
1zhv_score12 Avg=0.22
1zhv_talaris13 Avg=0.285
1zhv_talaris14 Avg=0.305
1zhv_ref2015 Avg=0.328
2a4d_score12 Avg=0.244
2a4d_talaris13 Avg=0.344
2a4d_talaris14 Avg=0.334
2a4d_ref2015 Avg=0.377
2h7w_score12 Avg=0.299
2h7w_talaris13 Avg=0.358
2h7w_talaris14 Avg=0.372
2h7w_ref2015 Avg=0.348
2jhx_score12 Avg=0.254
2jhx_talaris13 Avg=0.322
2jhx_talaris14 Avg=0.327
2jhx_ref2015 Avg=0.318
total_score
seqrec_seqrec

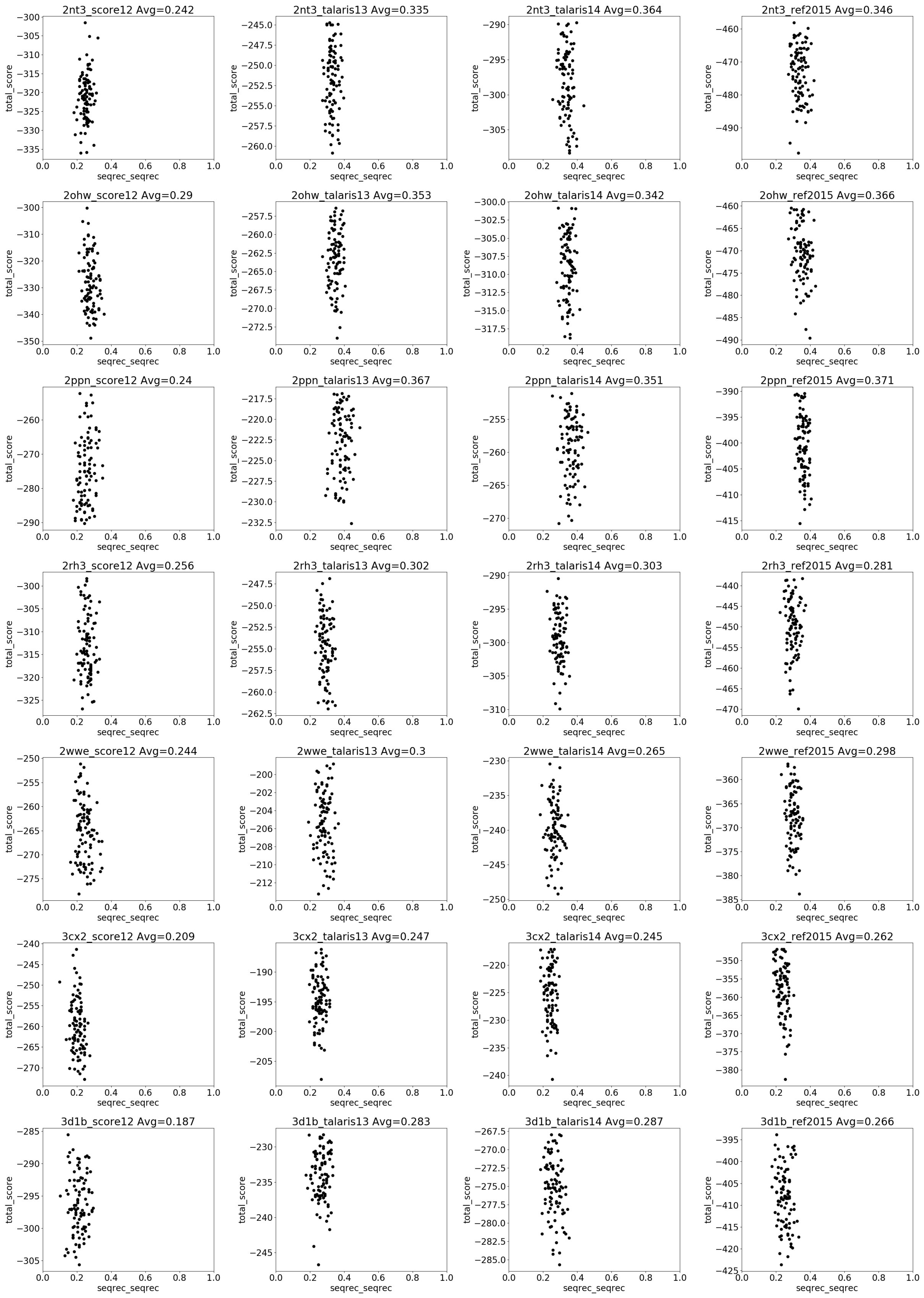
2nt3_score12 Avg=0.242
2nt3_talaris13 Avg=0.335
2nt3_talaris14 Avg=0.364
2nt3_ref2015 Avg=0.346
2ohw_score12 Avg=0.29
2ohw_talaris13 Avg=0.353
2ohw_talaris14 Avg=0.342
2ohw_ref2015 Avg=0.366
2ppn_score12 Avg=0.24
2ppn_talaris13 Avg=0.367
2ppn_talaris14 Avg=0.351
2ppn_ref2015 Avg=0.371
2rh3_score12 Avg=0.256
2rh3_talaris13 Avg=0.302
2rh3_talaris14 Avg=0.303
2rh3_ref2015 Avg=0.281
2wwe_score12 Avg=0.244
2wwe_talaris13 Avg=0.3
2wwe_talaris14 Avg=0.265
2wwe_ref2015 Avg=0.298
3cx2_score12 Avg=0.209
3cx2_talaris13 Avg=0.247
3cx2_talaris14 Avg=0.245
3cx2_ref2015 Avg=0.262
3d1b_score12 Avg=0.187
3d1b_talaris13 Avg=0.283
3d1b_talaris14 Avg=0.287
3d1b_ref2015 Avg=0.266
total_score
seqrec_seqrec

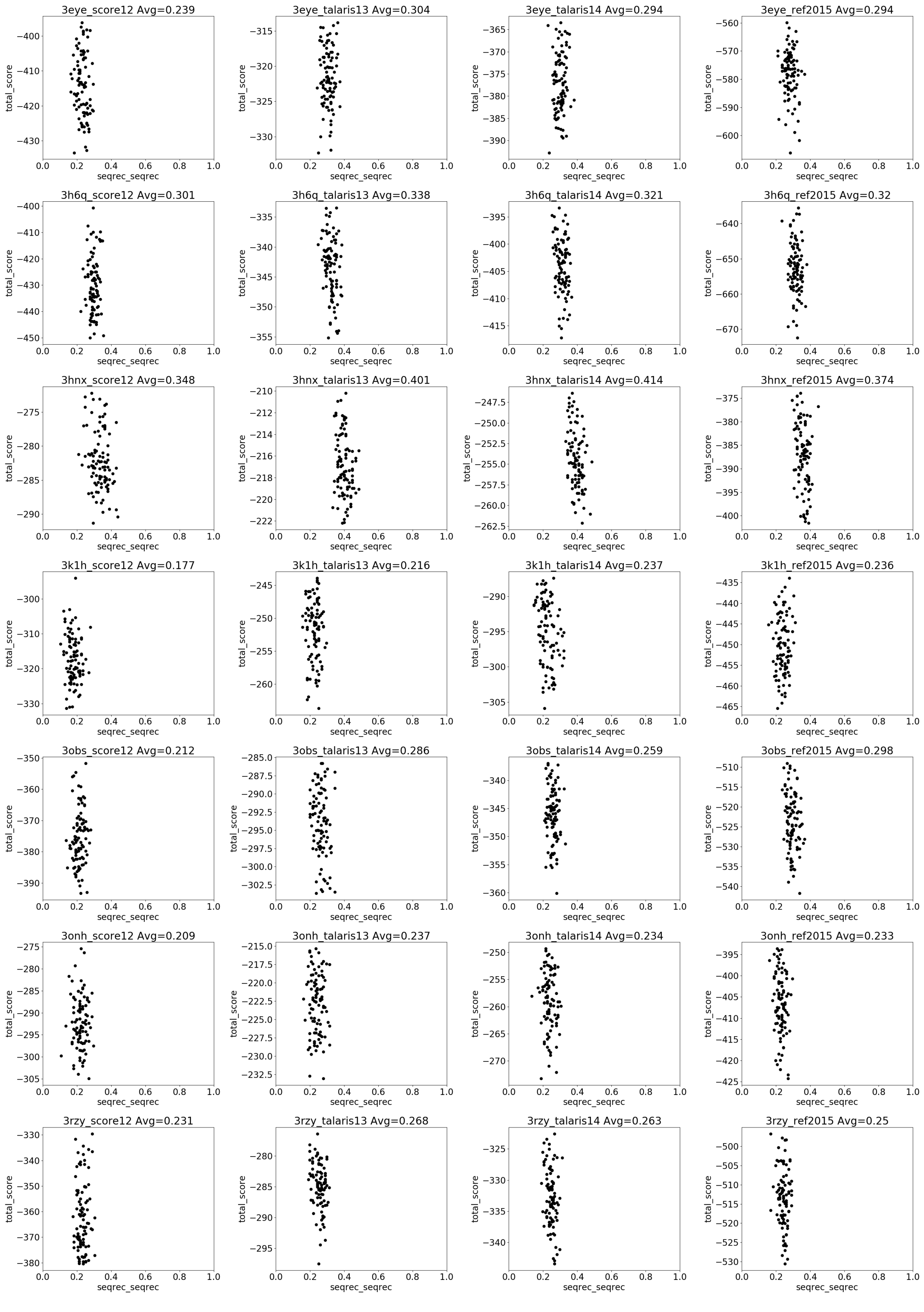
3eye_score12 Avg=0.239
3eye_talaris13 Avg=0.304
3eye_talaris14 Avg=0.294
3eye_ref2015 Avg=0.294
3h6q_score12 Avg=0.301
3h6q_talaris13 Avg=0.338
3h6q_talaris14 Avg=0.321
3h6q_ref2015 Avg=0.32
3hnx_score12 Avg=0.348
3hnx_talaris13 Avg=0.401
3hnx_talaris14 Avg=0.414
3hnx_ref2015 Avg=0.374
3k1h_score12 Avg=0.177
3k1h_talaris13 Avg=0.216
3k1h_talaris14 Avg=0.237
3k1h_ref2015 Avg=0.236
3obs_score12 Avg=0.212
3obs_talaris13 Avg=0.286
3obs_talaris14 Avg=0.259
3obs_ref2015 Avg=0.298
3onh_score12 Avg=0.209
3onh_talaris13 Avg=0.237
3onh_talaris14 Avg=0.234
3onh_ref2015 Avg=0.233
3rzy_score12 Avg=0.231
3rzy_talaris13 Avg=0.268
3rzy_talaris14 Avg=0.263
3rzy_ref2015 Avg=0.25
total_score
seqrec_seqrec

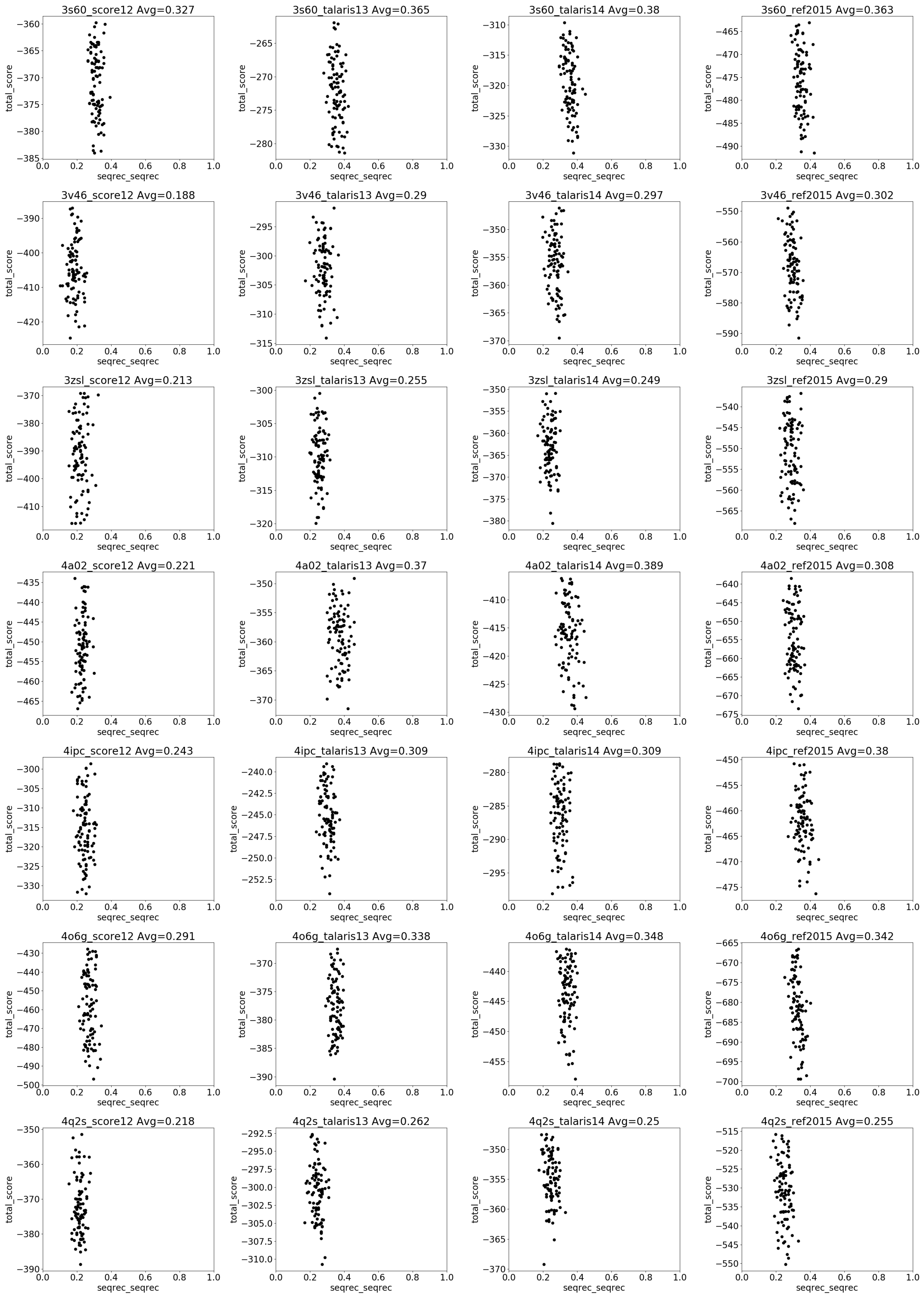
3s60_score12 Avg=0.327
3s60_talaris13 Avg=0.365
3s60_talaris14 Avg=0.38
3s60_ref2015 Avg=0.363
3v46_score12 Avg=0.188
3v46_talaris13 Avg=0.29
3v46_talaris14 Avg=0.297
3v46_ref2015 Avg=0.302
3zsl_score12 Avg=0.213
3zsl_talaris13 Avg=0.255
3zsl_talaris14 Avg=0.249
3zsl_ref2015 Avg=0.29
4a02_score12 Avg=0.221
4a02_talaris13 Avg=0.37
4a02_talaris14 Avg=0.389
4a02_ref2015 Avg=0.308
4ipc_score12 Avg=0.243
4ipc_talaris13 Avg=0.309
4ipc_talaris14 Avg=0.309
4ipc_ref2015 Avg=0.38
4o6g_score12 Avg=0.291
4o6g_talaris13 Avg=0.338
4o6g_talaris14 Avg=0.348
4o6g_ref2015 Avg=0.342
4q2s_score12 Avg=0.218
4q2s_talaris13 Avg=0.262
4q2s_talaris14 Avg=0.25
4q2s_ref2015 Avg=0.255
total_score
seqrec_seqrec

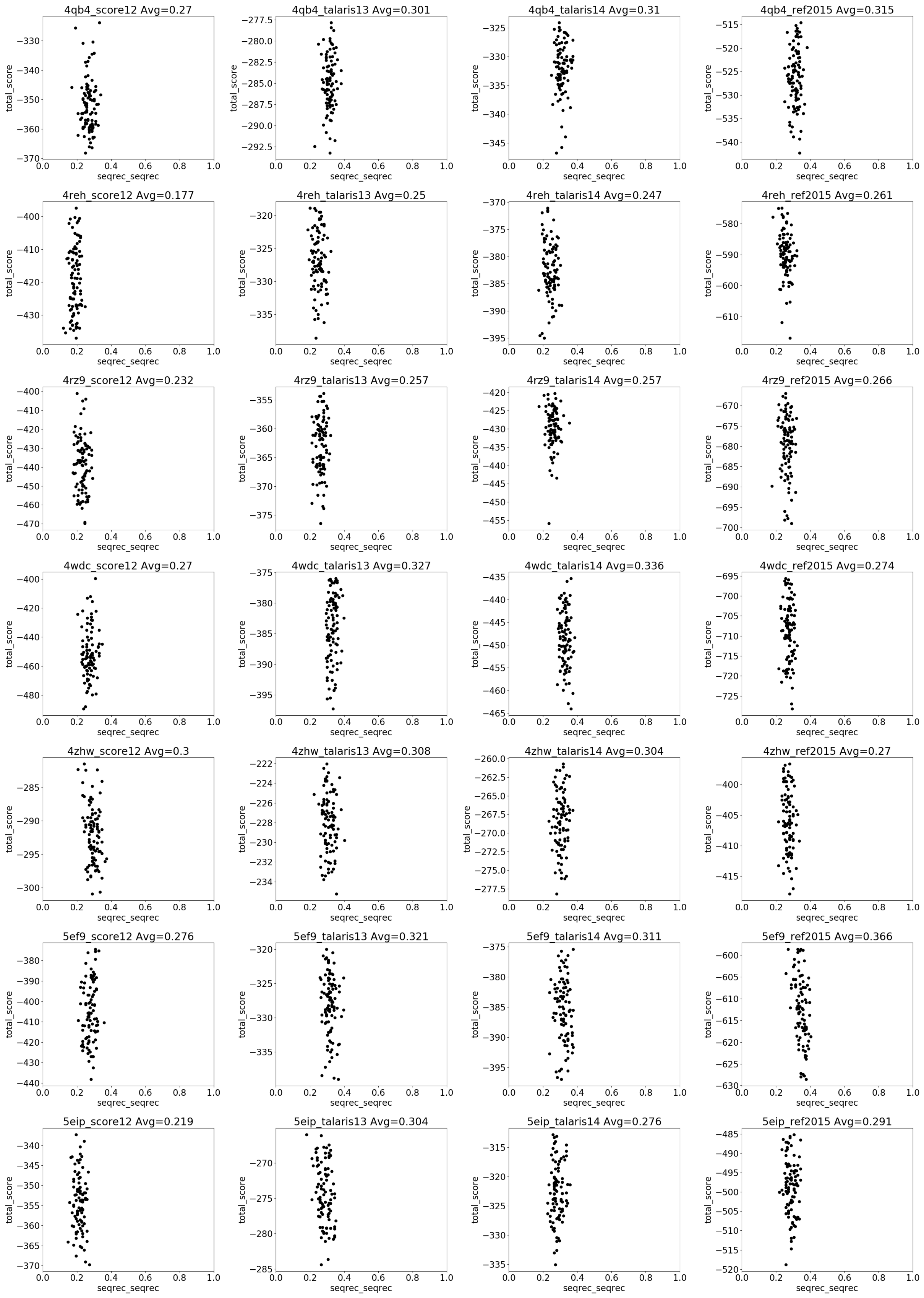
4qb4_score12 Avg=0.27
4qb4_talaris13 Avg=0.301
4qb4_talaris14 Avg=0.31
4qb4_ref2015 Avg=0.315
4reh_score12 Avg=0.177
4reh_talaris13 Avg=0.25
4reh_talaris14 Avg=0.247
4reh_ref2015 Avg=0.261
4rz9_score12 Avg=0.232
4rz9_talaris13 Avg=0.257
4rz9_talaris14 Avg=0.257
4rz9_ref2015 Avg=0.266
4wdc_score12 Avg=0.27
4wdc_talaris13 Avg=0.327
4wdc_talaris14 Avg=0.336
4wdc_ref2015 Avg=0.274
4zhw_score12 Avg=0.3
4zhw_talaris13 Avg=0.308
4zhw_talaris14 Avg=0.304
4zhw_ref2015 Avg=0.27
5ef9_score12 Avg=0.276
5ef9_talaris13 Avg=0.321
5ef9_talaris14 Avg=0.311
5ef9_ref2015 Avg=0.366
5eip_score12 Avg=0.219
5eip_talaris13 Avg=0.304
5eip_talaris14 Avg=0.276
5eip_ref2015 Avg=0.291
total_score
seqrec_seqrec

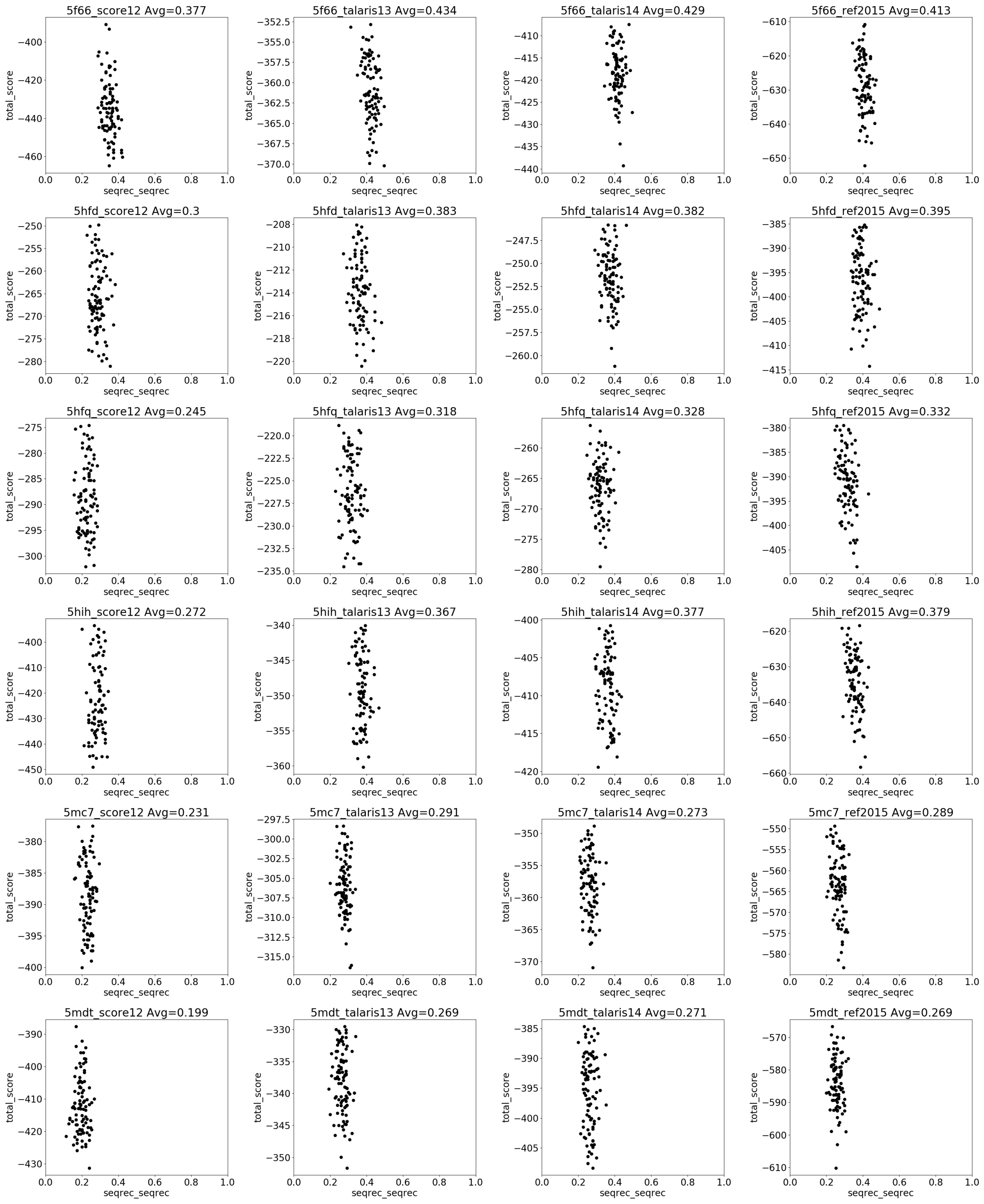

REVISION

revision: 61531

Scientific test: Scorefunction comparison for protein-protein docking

RESULTS

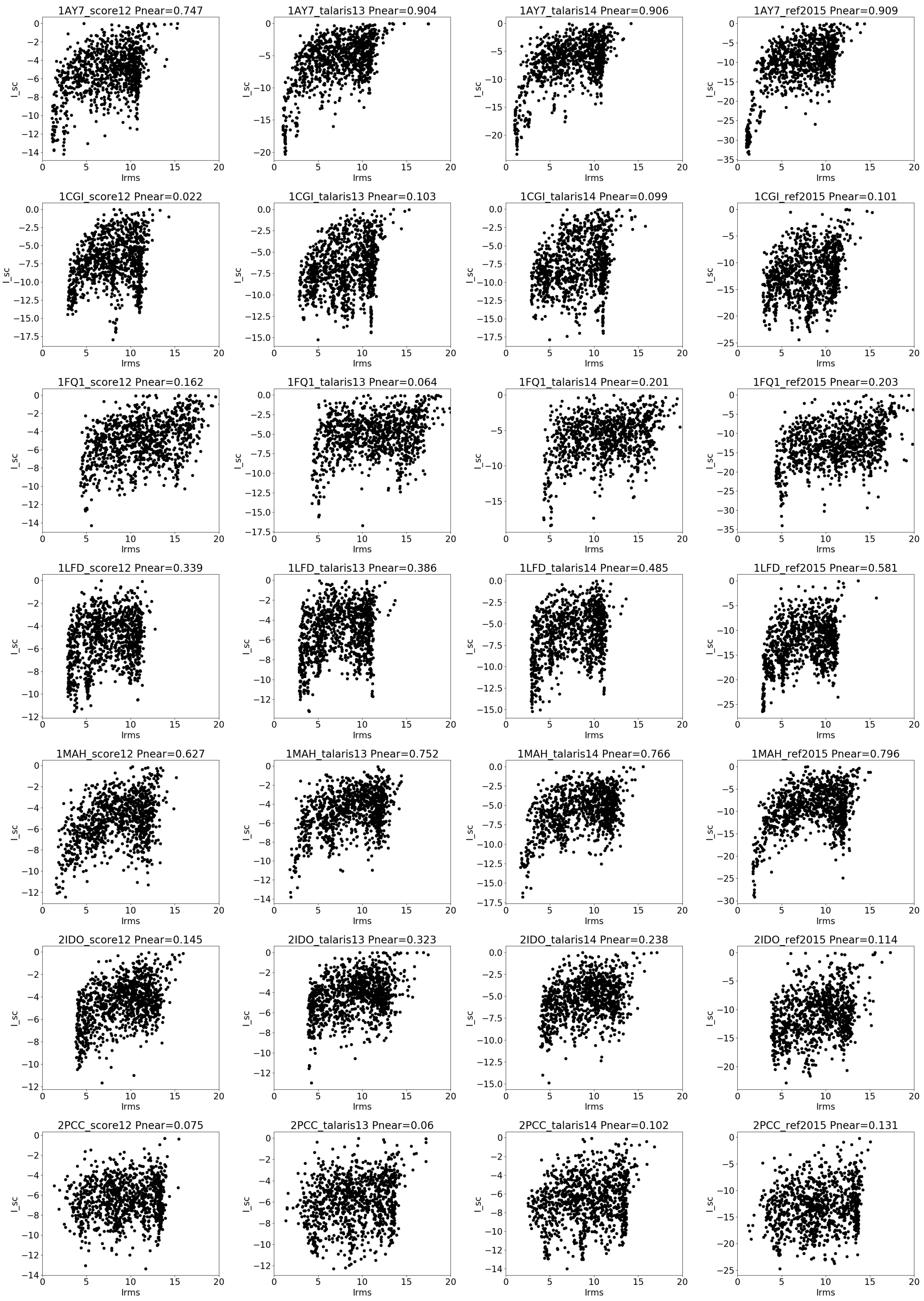

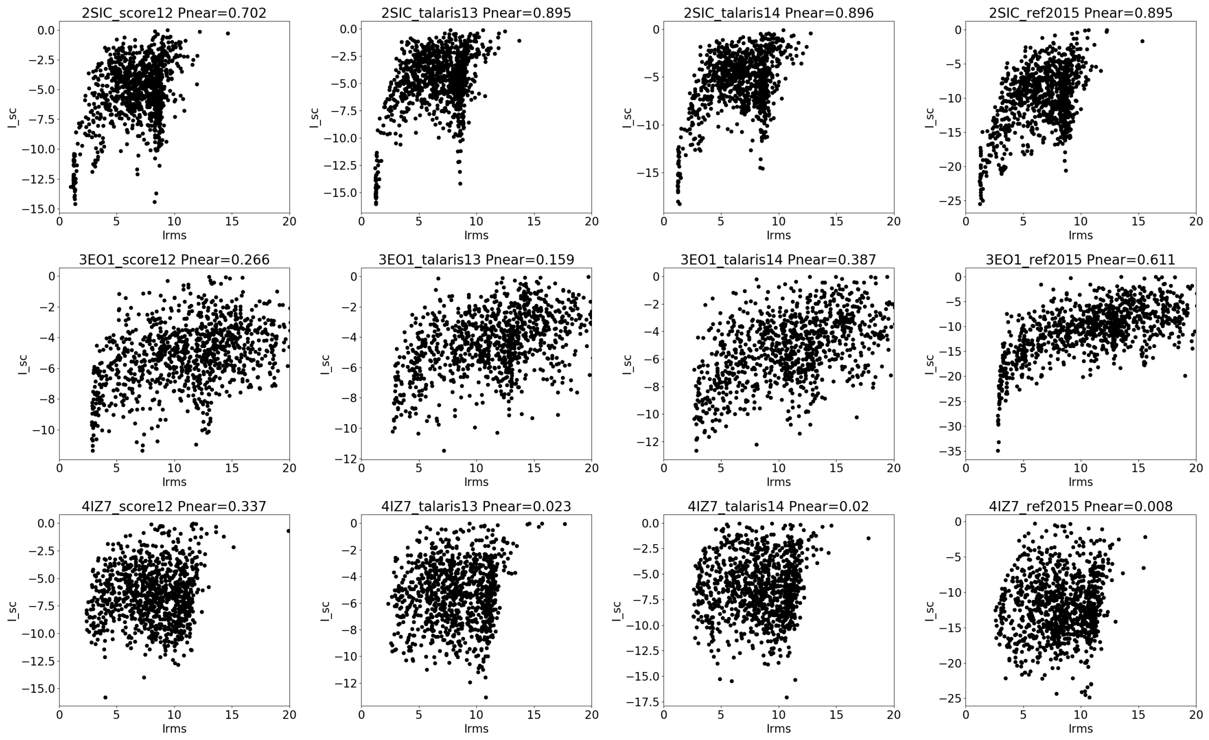

REVISION

revision: 61531

Scientific test: Scorefunction comparison for ligand_docking

RESULTS

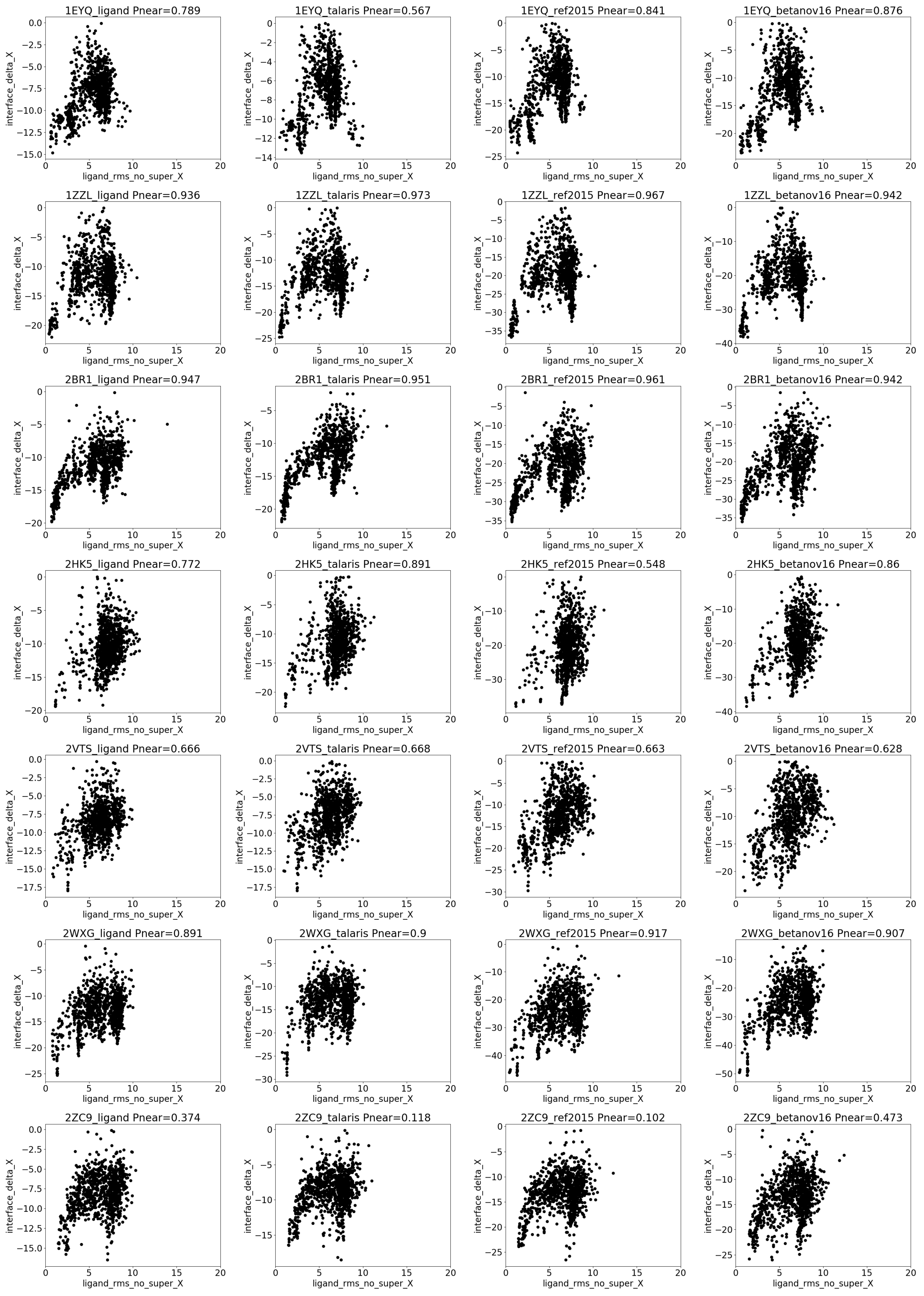

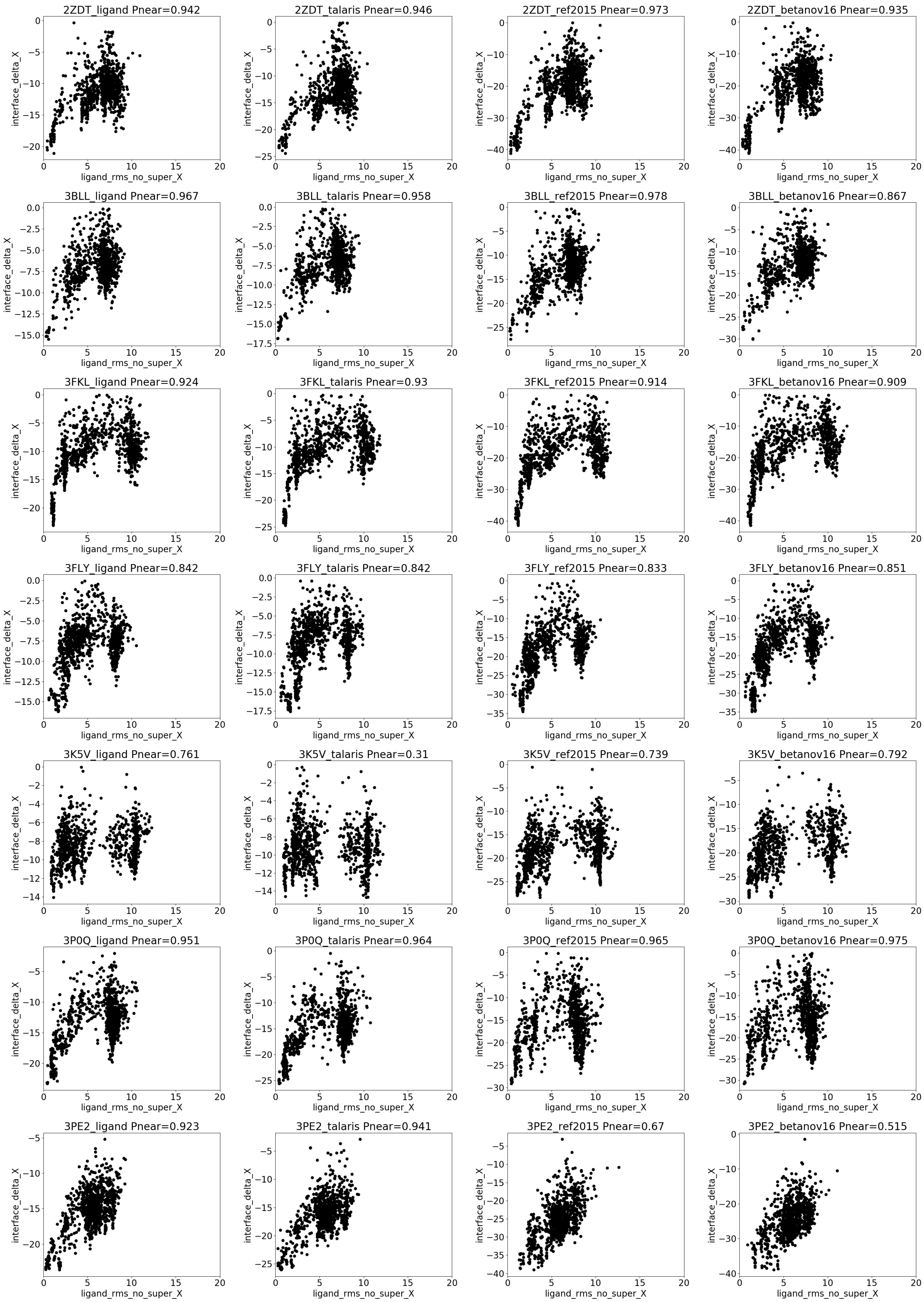

2ZDT_ligand Pnear=0.942
2ZDT_talaris Pnear=0.946
2ZDT_ref2015 Pnear=0.973
2ZDT_betanov16 Pnear=0.935
3BLL_ligand Pnear=0.967
3BLL_talaris Pnear=0.958
3BLL_ref2015 Pnear=0.978
3BLL_betanov16 Pnear=0.867
3FKL_ligand Pnear=0.924
3FKL_talaris Pnear=0.93
3FKL_ref2015 Pnear=0.914
3FKL_betanov16 Pnear=0.909
3FLY_ligand Pnear=0.842
3FLY_talaris Pnear=0.842
3FLY_ref2015 Pnear=0.833
3FLY_betanov16 Pnear=0.851
3K5V_ligand Pnear=0.761
3K5V_talaris Pnear=0.31
3K5V_ref2015 Pnear=0.739
3K5V_betanov16 Pnear=0.792
3P0Q_ligand Pnear=0.951
3P0Q_talaris Pnear=0.964
3P0Q_ref2015 Pnear=0.965
3P0Q_betanov16 Pnear=0.975
3PE2_ligand Pnear=0.923
3PE2_talaris Pnear=0.941
3PE2_ref2015 Pnear=0.67
3PE2_betanov16 Pnear=0.515
interface_delta_X
ligand_rms_no_super_X

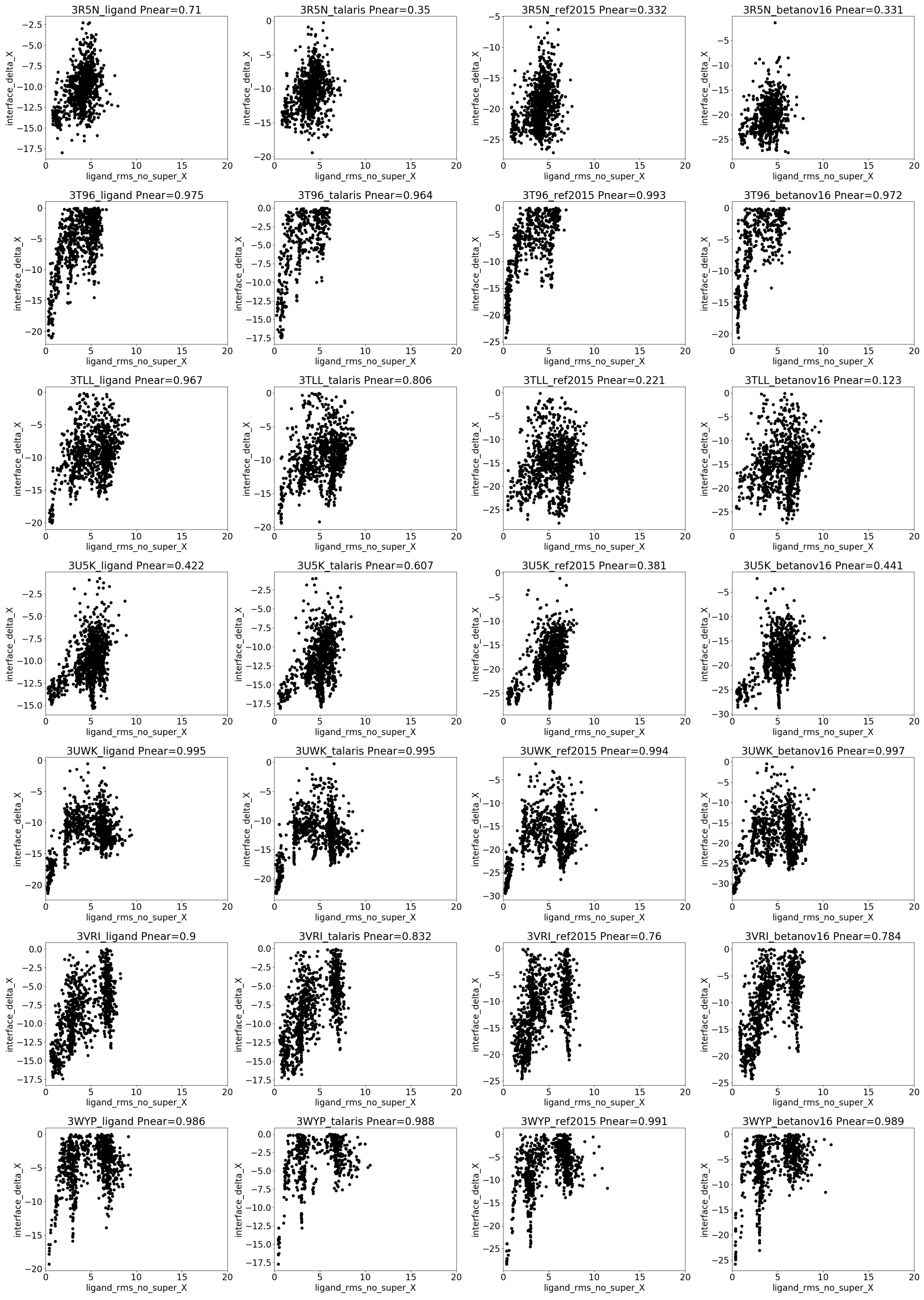

3R5N_ligand Pnear=0.71
3R5N_talaris Pnear=0.35
3R5N_ref2015 Pnear=0.332
3R5N_betanov16 Pnear=0.331
3T96_ligand Pnear=0.975
3T96_talaris Pnear=0.964
3T96_ref2015 Pnear=0.993
3T96_betanov16 Pnear=0.972
3TLL_ligand Pnear=0.967
3TLL_talaris Pnear=0.806
3TLL_ref2015 Pnear=0.221
3TLL_betanov16 Pnear=0.123
3U5K_ligand Pnear=0.422
3U5K_talaris Pnear=0.607
3U5K_ref2015 Pnear=0.381
3U5K_betanov16 Pnear=0.441
3UWK_ligand Pnear=0.995
3UWK_talaris Pnear=0.995
3UWK_ref2015 Pnear=0.994
3UWK_betanov16 Pnear=0.997
3VRI_ligand Pnear=0.9
3VRI_talaris Pnear=0.832
3VRI_ref2015 Pnear=0.76
3VRI_betanov16 Pnear=0.784
3WYP_ligand Pnear=0.986
3WYP_talaris Pnear=0.988
3WYP_ref2015 Pnear=0.991
3WYP_betanov16 Pnear=0.989
interface_delta_X
ligand_rms_no_super_X

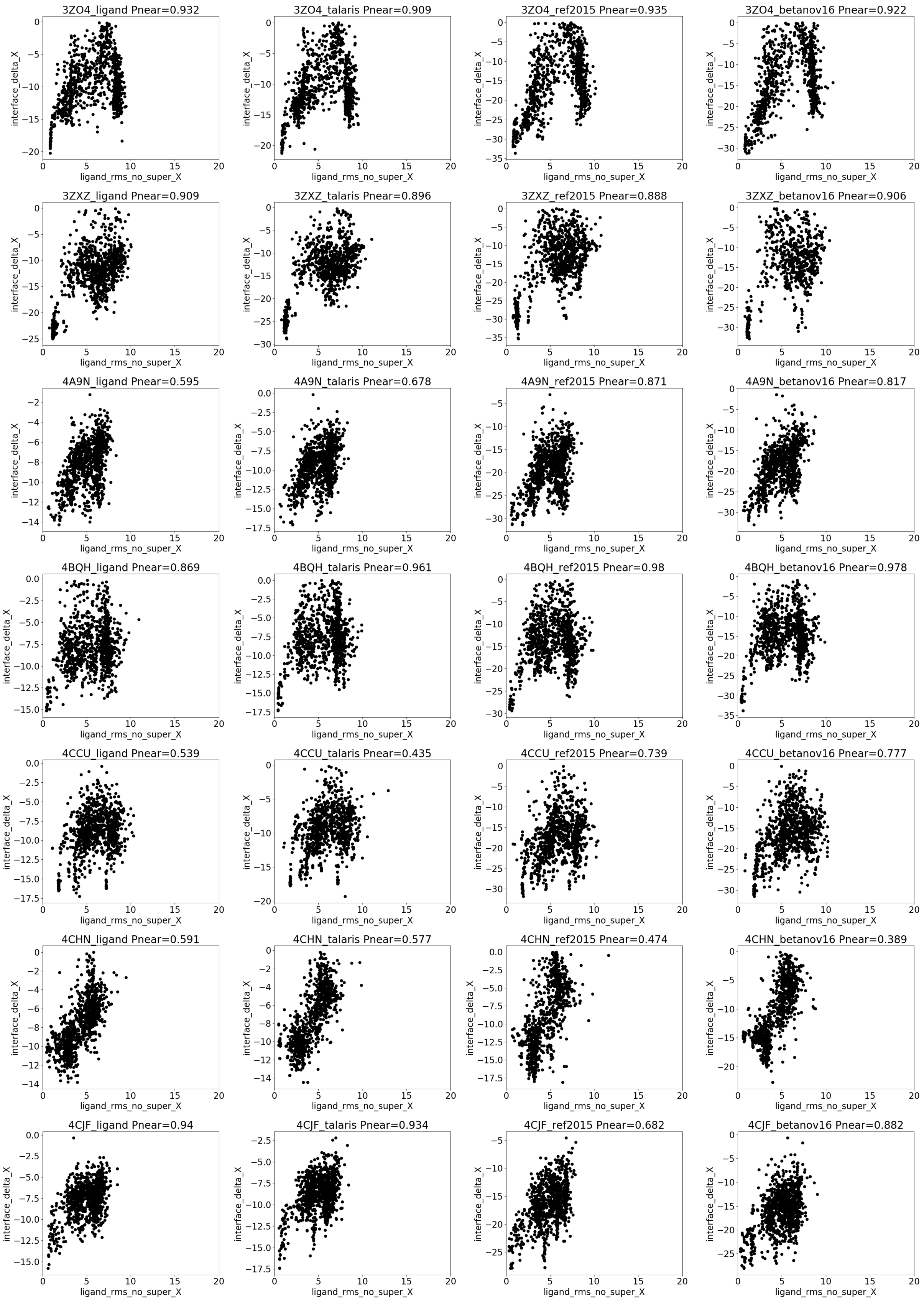

3ZO4_ligand Pnear=0.932
3ZO4_talaris Pnear=0.909
3ZO4_ref2015 Pnear=0.935
3ZO4_betanov16 Pnear=0.922
3ZXZ_ligand Pnear=0.909
3ZXZ_talaris Pnear=0.896
3ZXZ_ref2015 Pnear=0.888
3ZXZ_betanov16 Pnear=0.906
4A9N_ligand Pnear=0.595
4A9N_talaris Pnear=0.678
4A9N_ref2015 Pnear=0.871
4A9N_betanov16 Pnear=0.817
4BQH_ligand Pnear=0.869
4BQH_talaris Pnear=0.961
4BQH_ref2015 Pnear=0.98
4BQH_betanov16 Pnear=0.978
4CCU_ligand Pnear=0.539
4CCU_talaris Pnear=0.435
4CCU_ref2015 Pnear=0.739
4CCU_betanov16 Pnear=0.777
4CHN_ligand Pnear=0.591
4CHN_talaris Pnear=0.577
4CHN_ref2015 Pnear=0.474
4CHN_betanov16 Pnear=0.389
4CJF_ligand Pnear=0.94
4CJF_talaris Pnear=0.934
4CJF_ref2015 Pnear=0.682
4CJF_betanov16 Pnear=0.882
interface_delta_X
ligand_rms_no_super_X

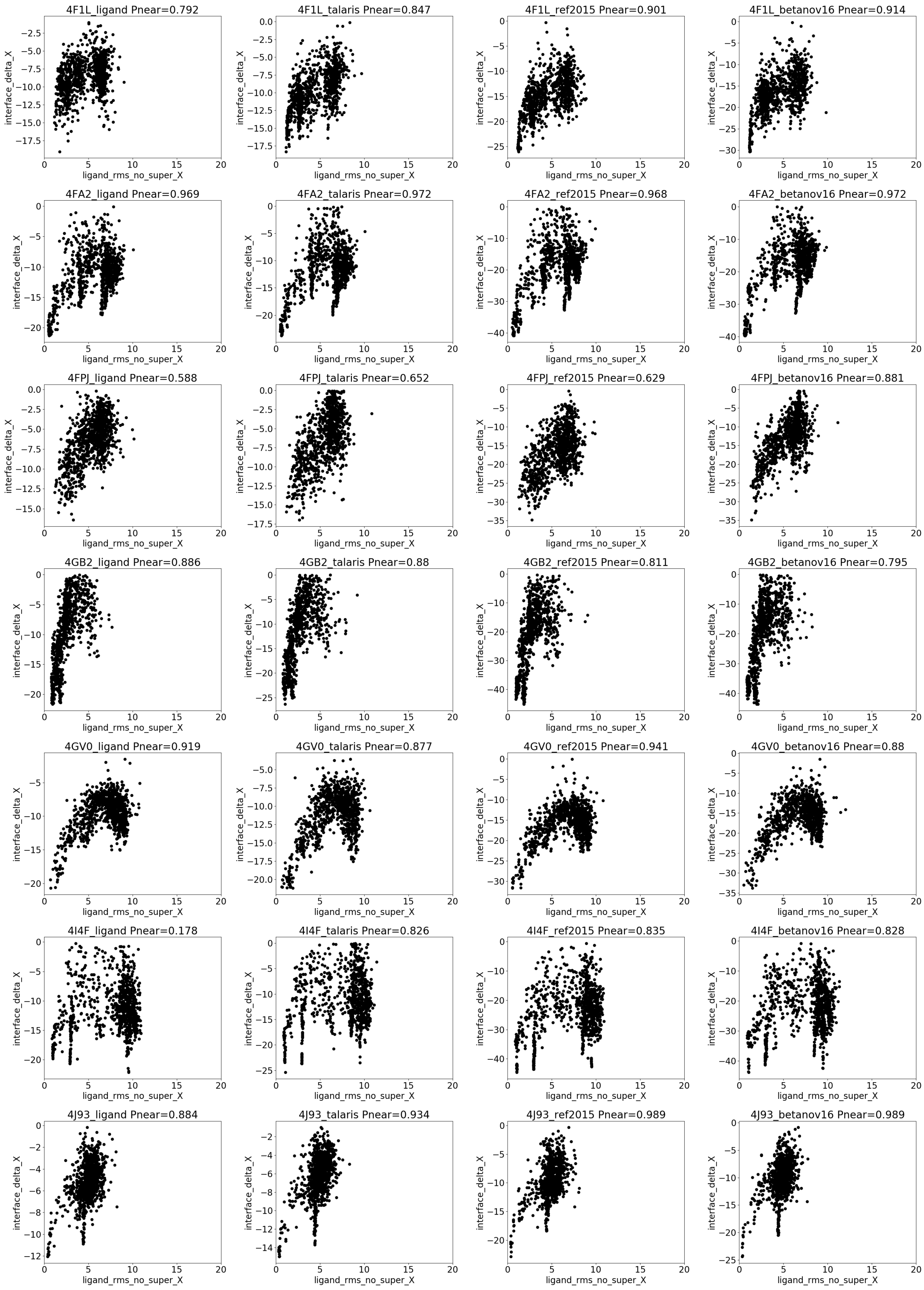
4F1L_ligand Pnear=0.792
4F1L_talaris Pnear=0.847
4F1L_ref2015 Pnear=0.901
4F1L_betanov16 Pnear=0.914
4FA2_ligand Pnear=0.969
4FA2_talaris Pnear=0.972
4FA2_ref2015 Pnear=0.968
4FA2_betanov16 Pnear=0.972
4FPJ_ligand Pnear=0.588
4FPJ_talaris Pnear=0.652
4FPJ_ref2015 Pnear=0.629
4FPJ_betanov16 Pnear=0.881
4GB2_ligand Pnear=0.886
4GB2_talaris Pnear=0.88
4GB2_ref2015 Pnear=0.811
4GB2_betanov16 Pnear=0.795
4GV0_ligand Pnear=0.919
4GV0_talaris Pnear=0.877
4GV0_ref2015 Pnear=0.941
4GV0_betanov16 Pnear=0.88
4I4F_ligand Pnear=0.178
4I4F_talaris Pnear=0.826
4I4F_ref2015 Pnear=0.835
4I4F_betanov16 Pnear=0.828
4J93_ligand Pnear=0.884
4J93_talaris Pnear=0.934
4J93_ref2015 Pnear=0.989
4J93_betanov16 Pnear=0.989
interface_delta_X
ligand_rms_no_super_X

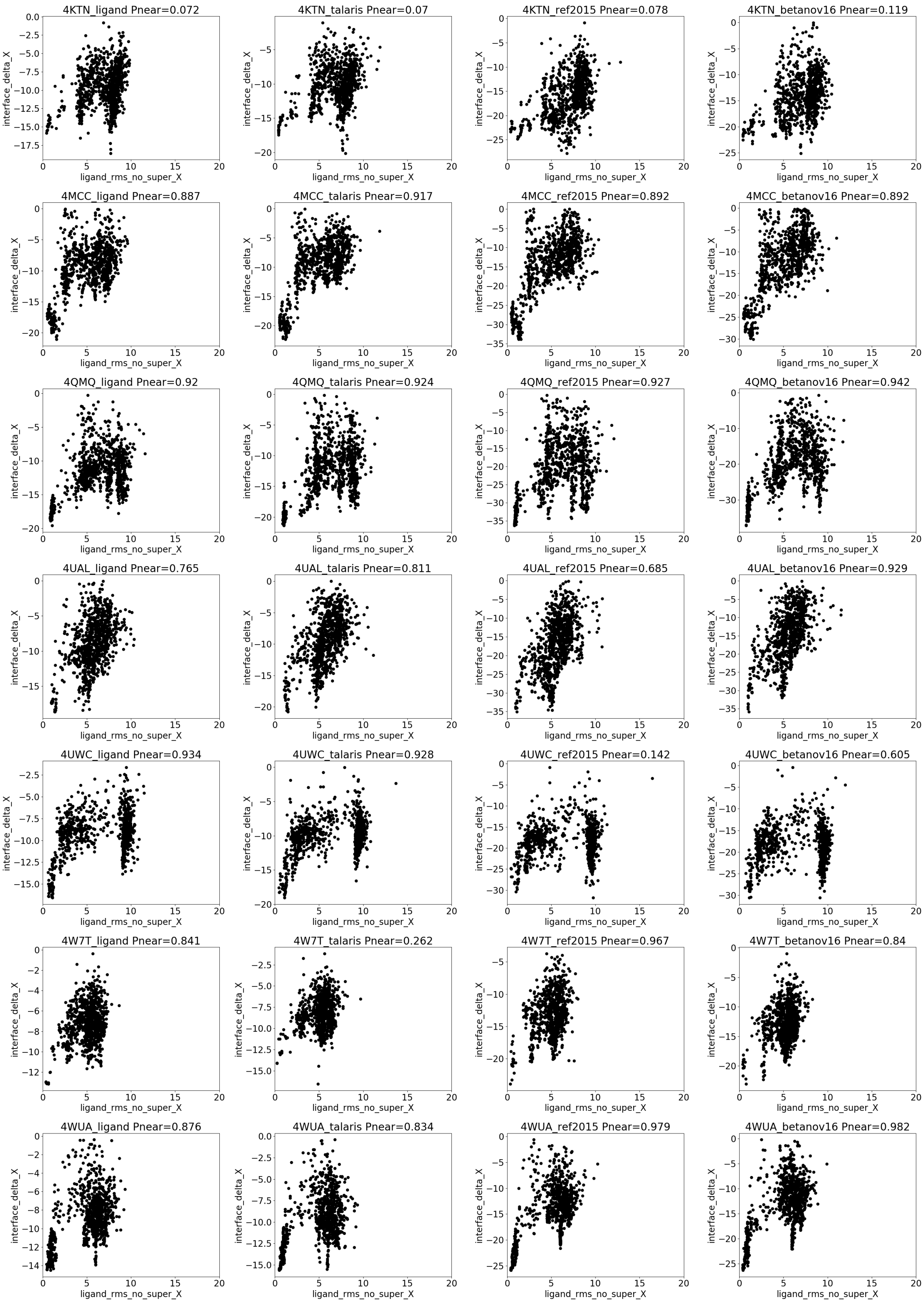
4KTN_ligand Pnear=0.072
4KTN_talaris Pnear=0.07
4KTN_ref2015 Pnear=0.078
4KTN_betanov16 Pnear=0.119
4MCC_ligand Pnear=0.887
4MCC_talaris Pnear=0.917
4MCC_ref2015 Pnear=0.892
4MCC_betanov16 Pnear=0.892
4QMQ_ligand Pnear=0.92
4QMQ_talaris Pnear=0.924
4QMQ_ref2015 Pnear=0.927
4QMQ_betanov16 Pnear=0.942
4UAL_ligand Pnear=0.765
4UAL_talaris Pnear=0.811
4UAL_ref2015 Pnear=0.685
4UAL_betanov16 Pnear=0.929
4UWC_ligand Pnear=0.934
4UWC_talaris Pnear=0.928
4UWC_ref2015 Pnear=0.142
4UWC_betanov16 Pnear=0.605
4W7T_ligand Pnear=0.841
4W7T_talaris Pnear=0.262
4W7T_ref2015 Pnear=0.967
4W7T_betanov16 Pnear=0.84
4WUA_ligand Pnear=0.876
4WUA_talaris Pnear=0.834
4WUA_ref2015 Pnear=0.979
4WUA_betanov16 Pnear=0.982
interface_delta_X
ligand_rms_no_super_X

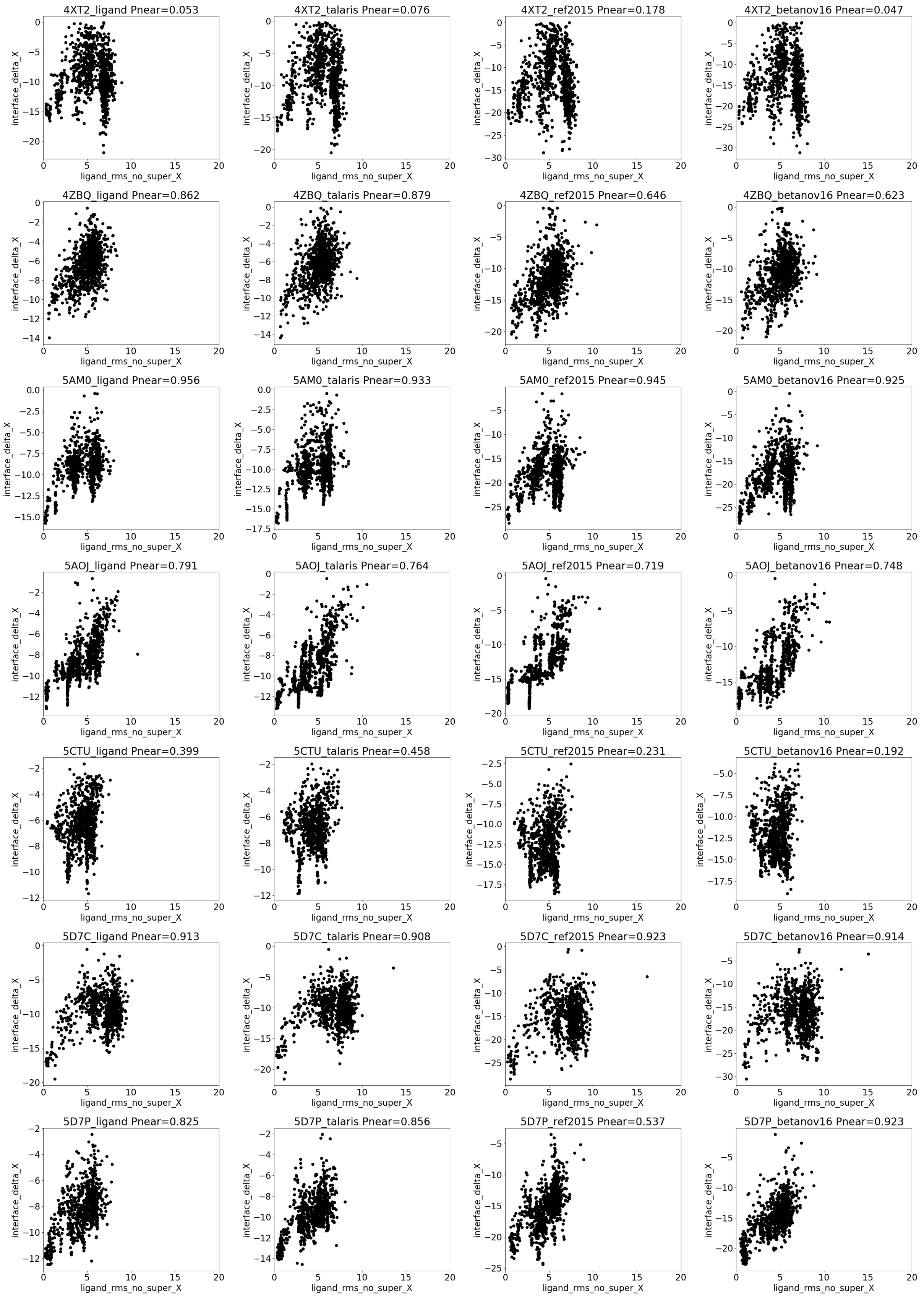
4XT2_ligand Pnear=0.053
4XT2_talaris Pnear=0.076
4XT2_ref2015 Pnear=0.178
4XT2_betanov16 Pnear=0.047
4ZBQ_ligand Pnear=0.862
4ZBQ_talaris Pnear=0.879
4ZBQ_ref2015 Pnear=0.646
4ZBQ_betanov16 Pnear=0.623
5AM0_ligand Pnear=0.956
5AM0_talaris Pnear=0.933
5AM0_ref2015 Pnear=0.945
5AM0_betanov16 Pnear=0.925
5AOJ_ligand Pnear=0.791
5AOJ_talaris Pnear=0.764
5AOJ_ref2015 Pnear=0.719
5AOJ_betanov16 Pnear=0.748
5CTU_ligand Pnear=0.399
5CTU_talaris Pnear=0.458
5CTU_ref2015 Pnear=0.231
5CTU_betanov16 Pnear=0.192
5D7C_ligand Pnear=0.913
5D7C_talaris Pnear=0.908
5D7C_ref2015 Pnear=0.923
5D7C_betanov16 Pnear=0.914
5D7P_ligand Pnear=0.825
5D7P_talaris Pnear=0.856
5D7P_ref2015 Pnear=0.537
5D7P_betanov16 Pnear=0.923
interface_delta_X
ligand_rms_no_super_X

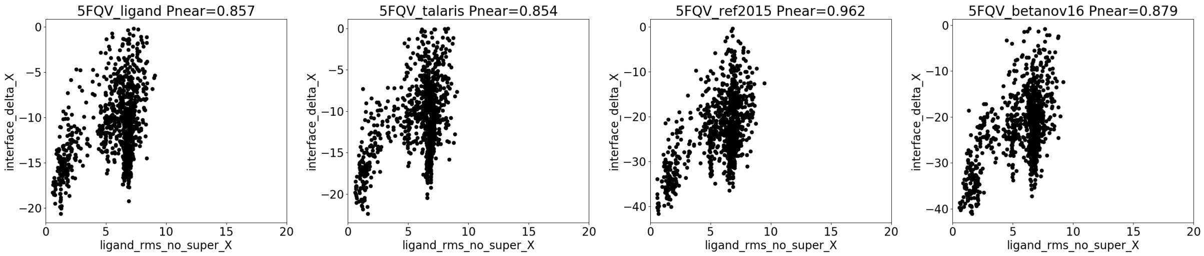

REVISION

revision: 61531

Scientific test: Scorefunction comparison for loop modeling with Cyclic Coordinate Descent (CCD)

RESULTS

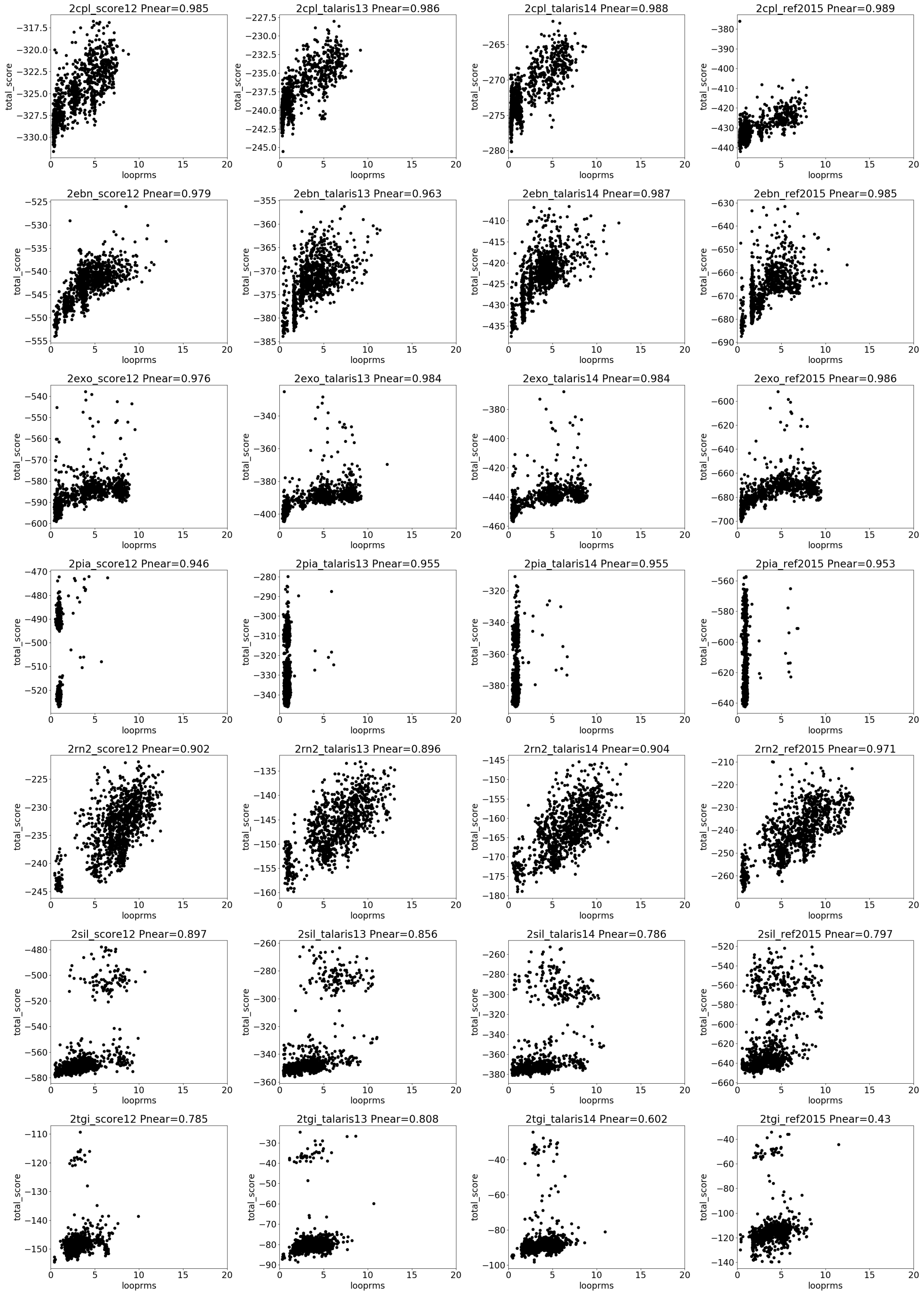

REVISION

revision: 61531

Scientific test: Scorefunction comparison for loop modeling with KIC

RESULTS

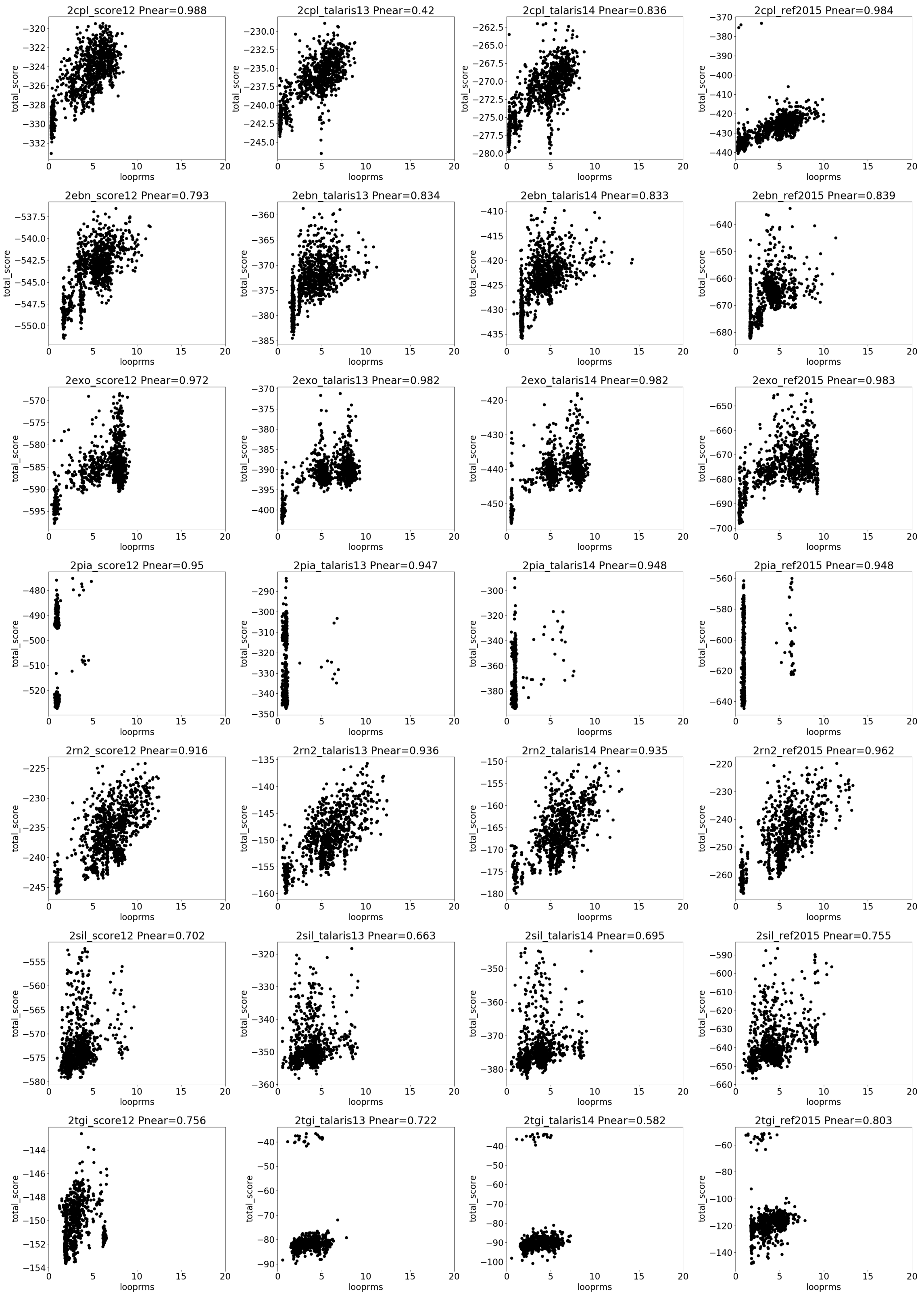

REVISION

revision: 61531

Scientific test: Scorefunction comparison for loop modeling with KIC with fragments

RESULTS

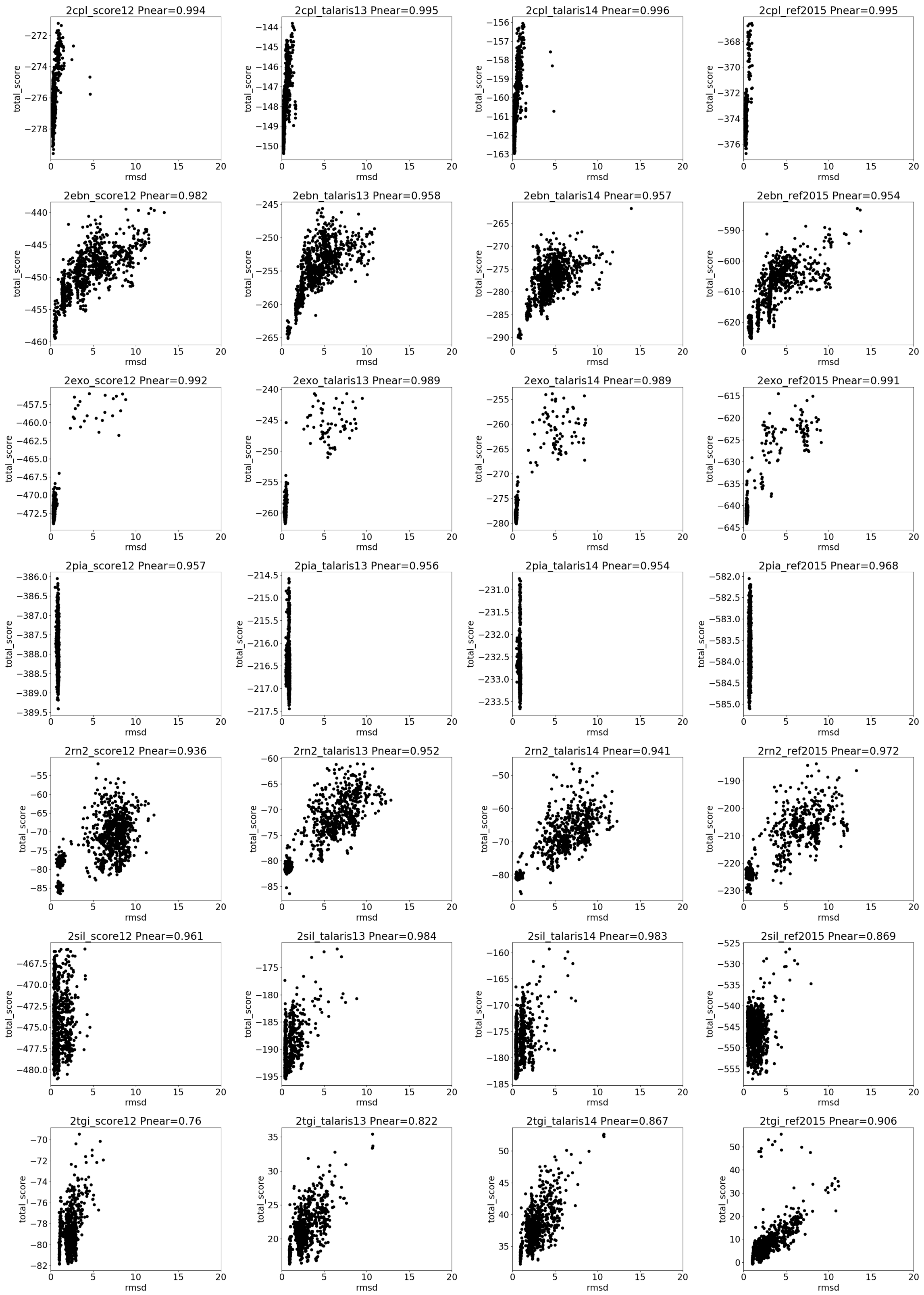

REVISION

revision: 61531

Scientific test: Scorefunction comparison for loop modeling with Next Generation KIC (NGK)

RESULTS

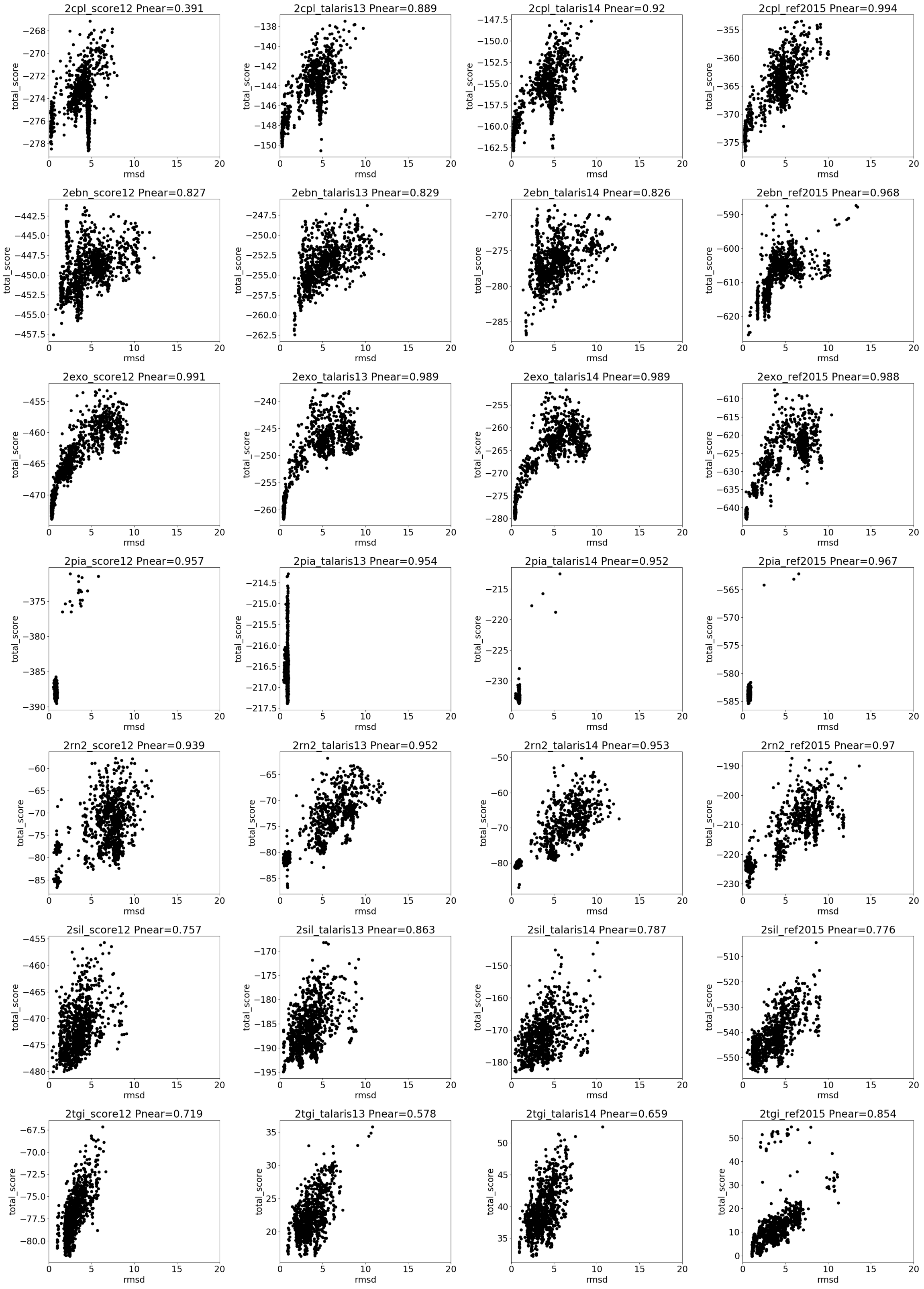

REVISION

revision: 61531

Scientific test: Scorefunction comparison for cartesian relax

RESULTS

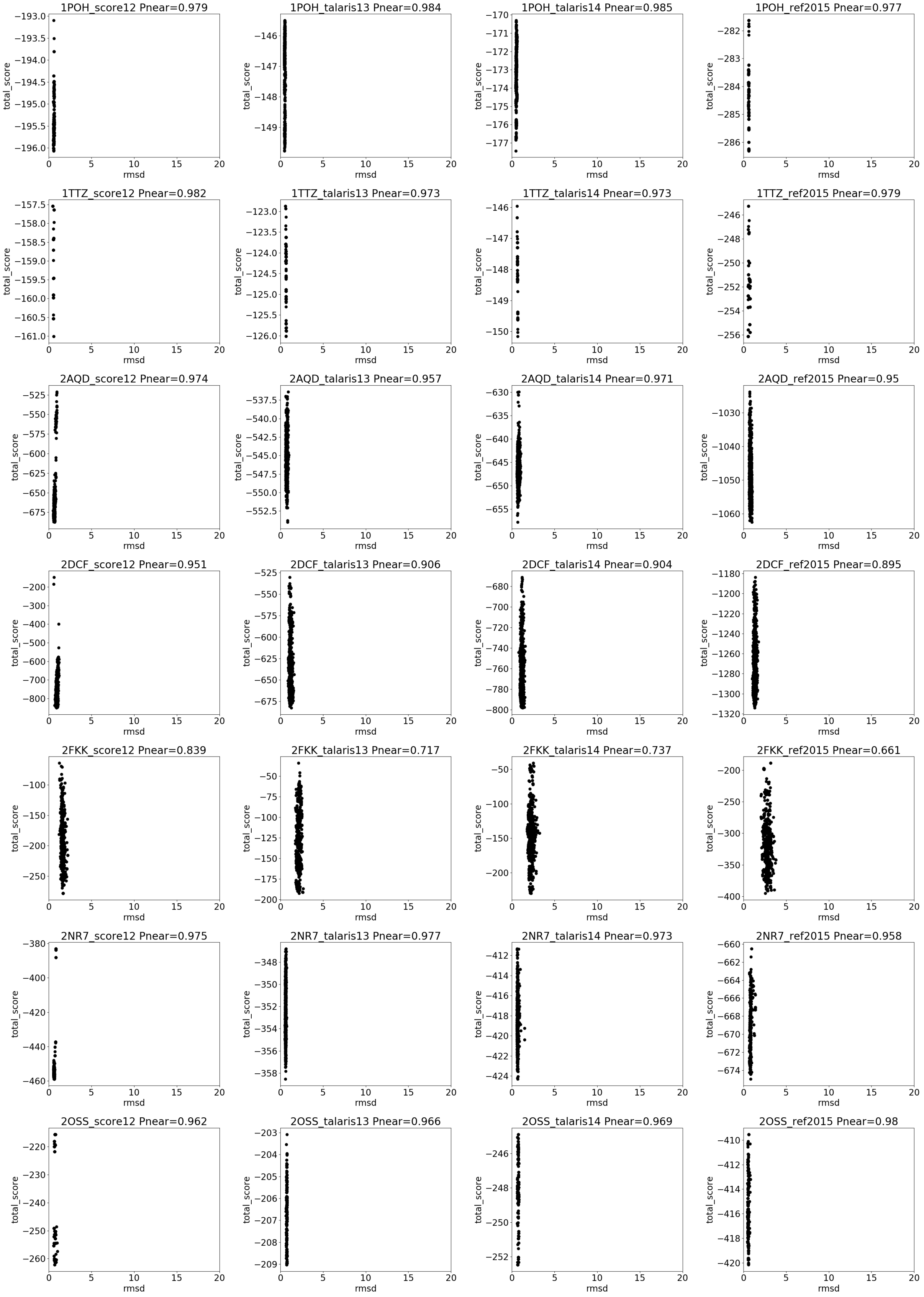

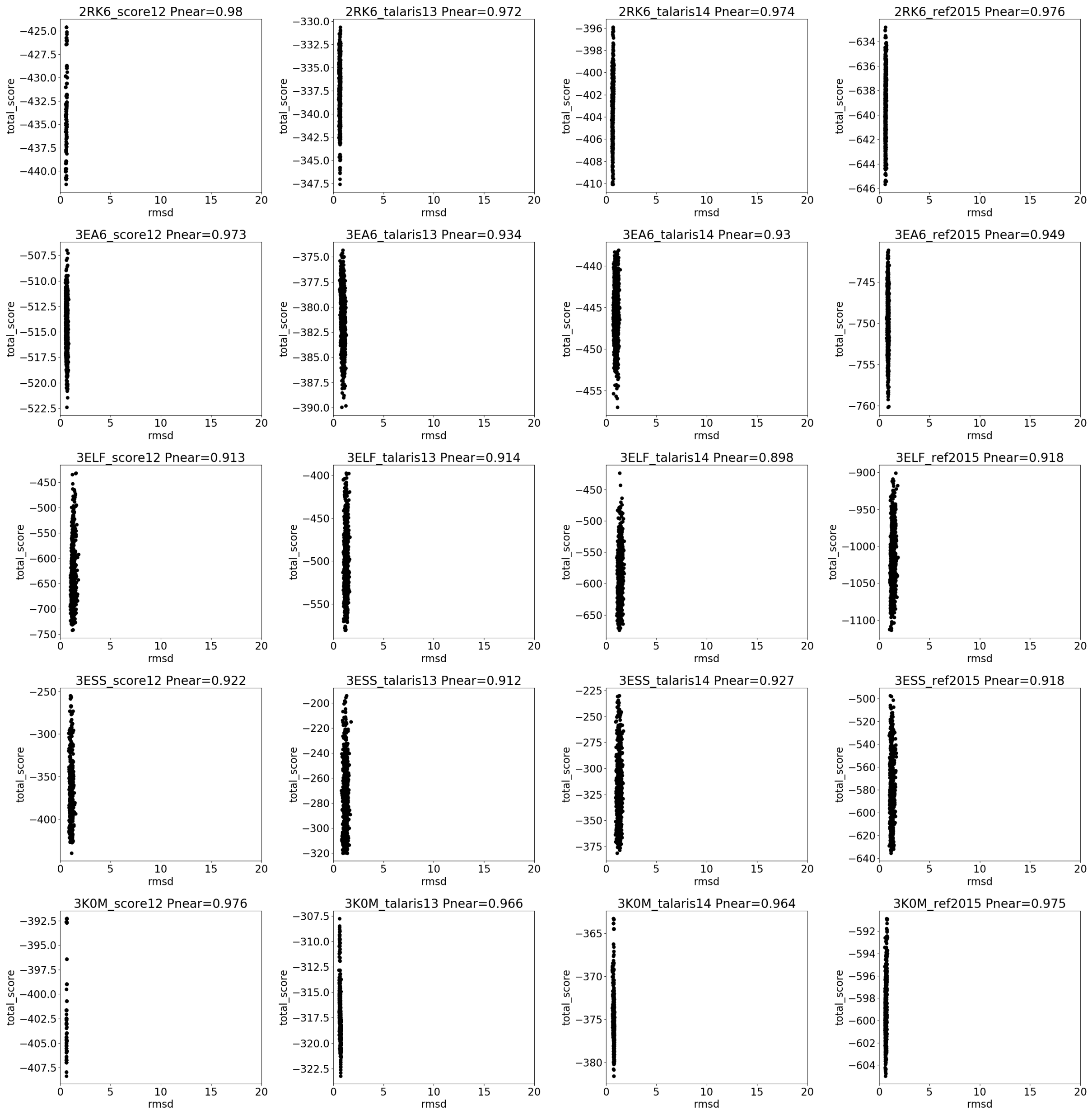

## REVISION

revision: 61531

Scientific test: Scorefunction comparison for FastRelax

RESULTS

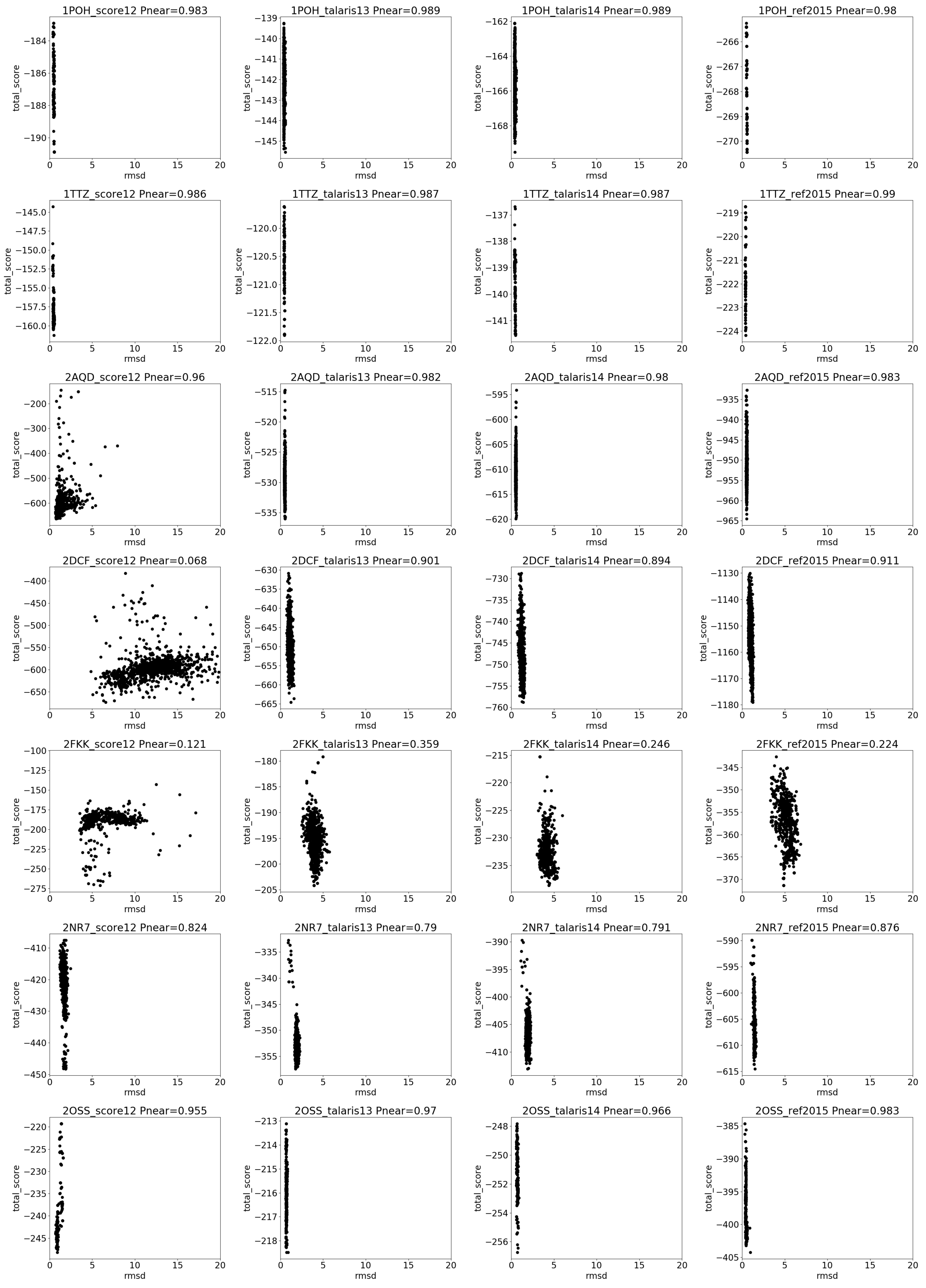

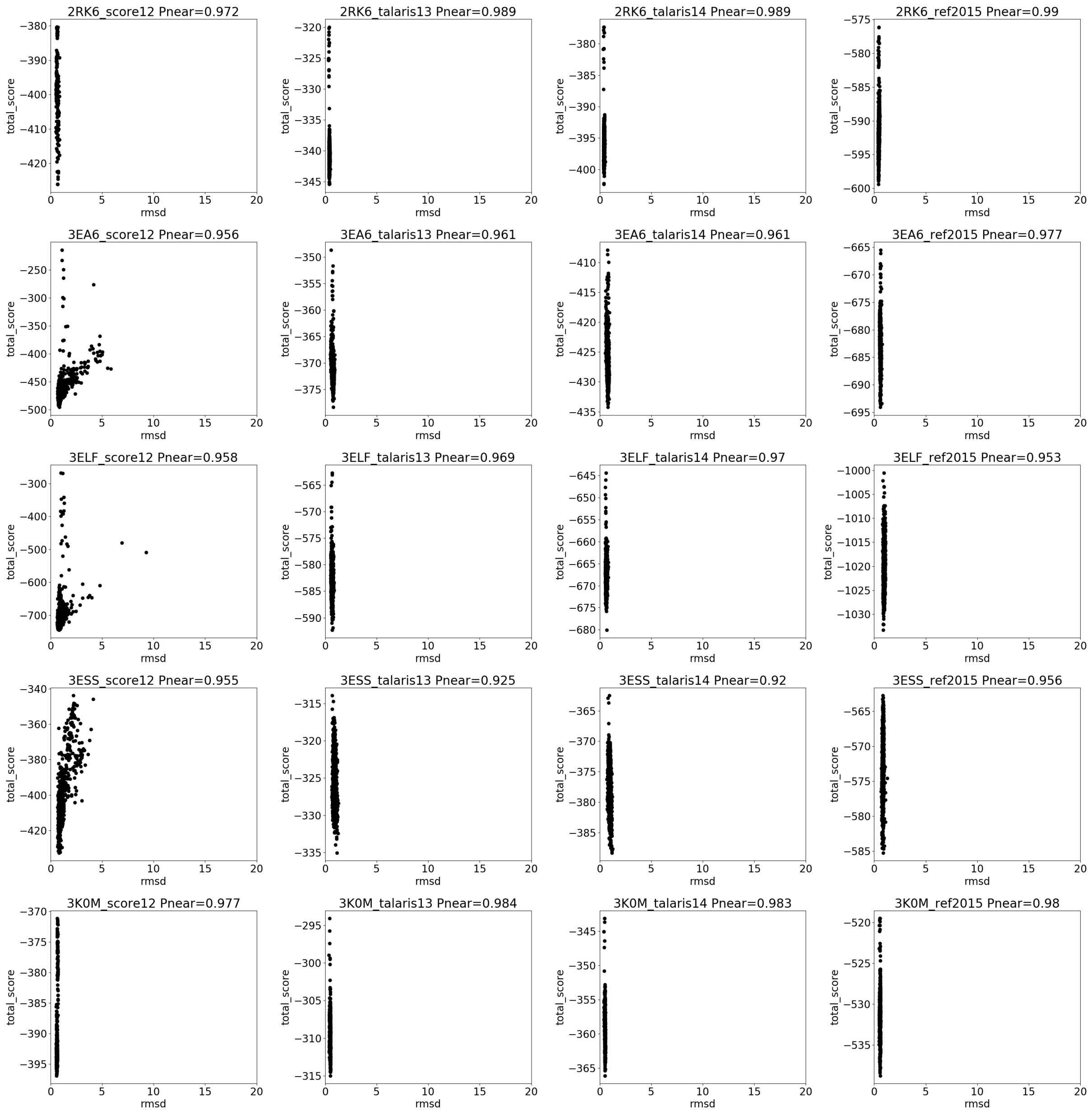

REVISION

revision: 61531

Scientific test: Scorefunction comparison for FastRelax with 5 iterations

RESULTS

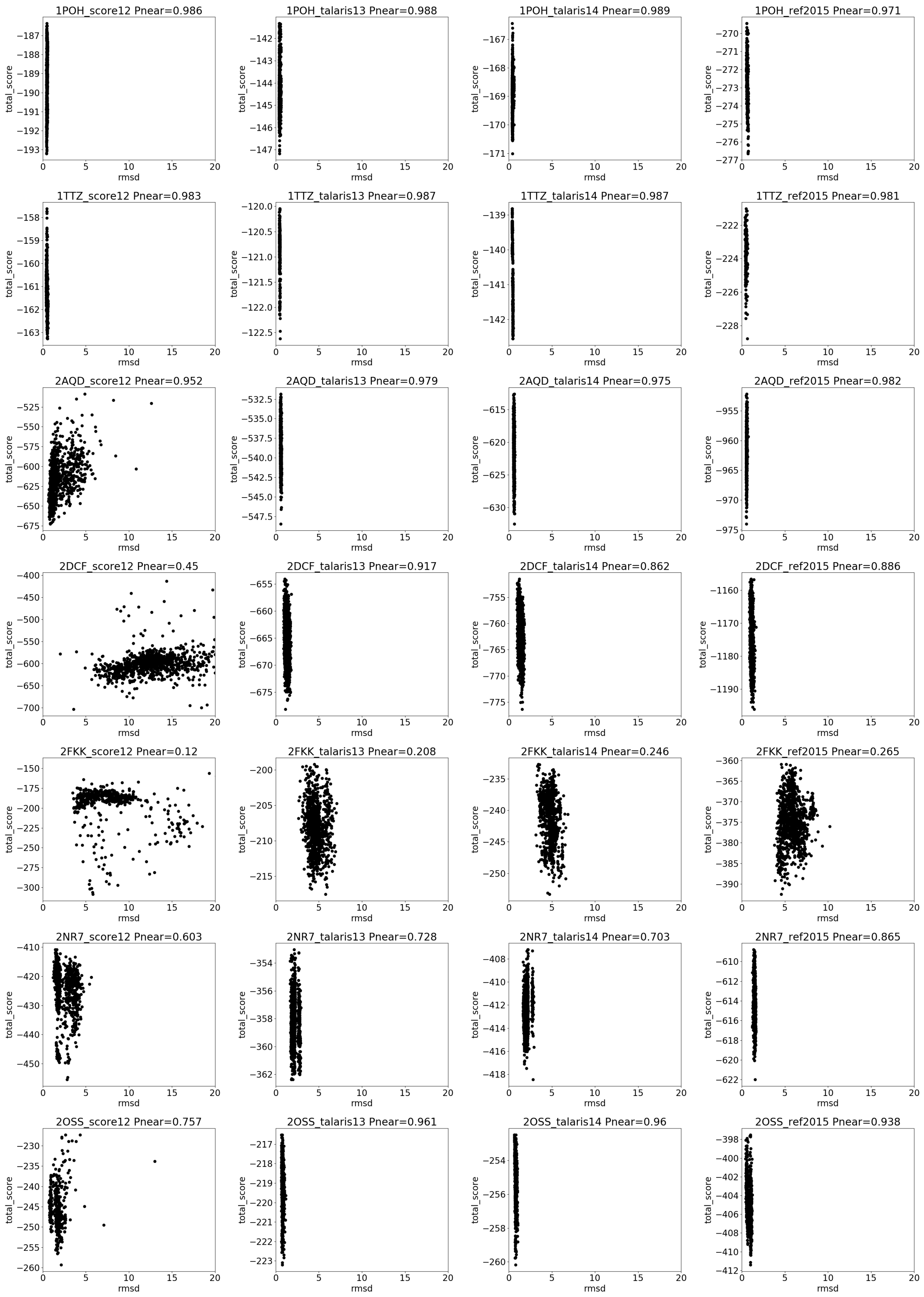

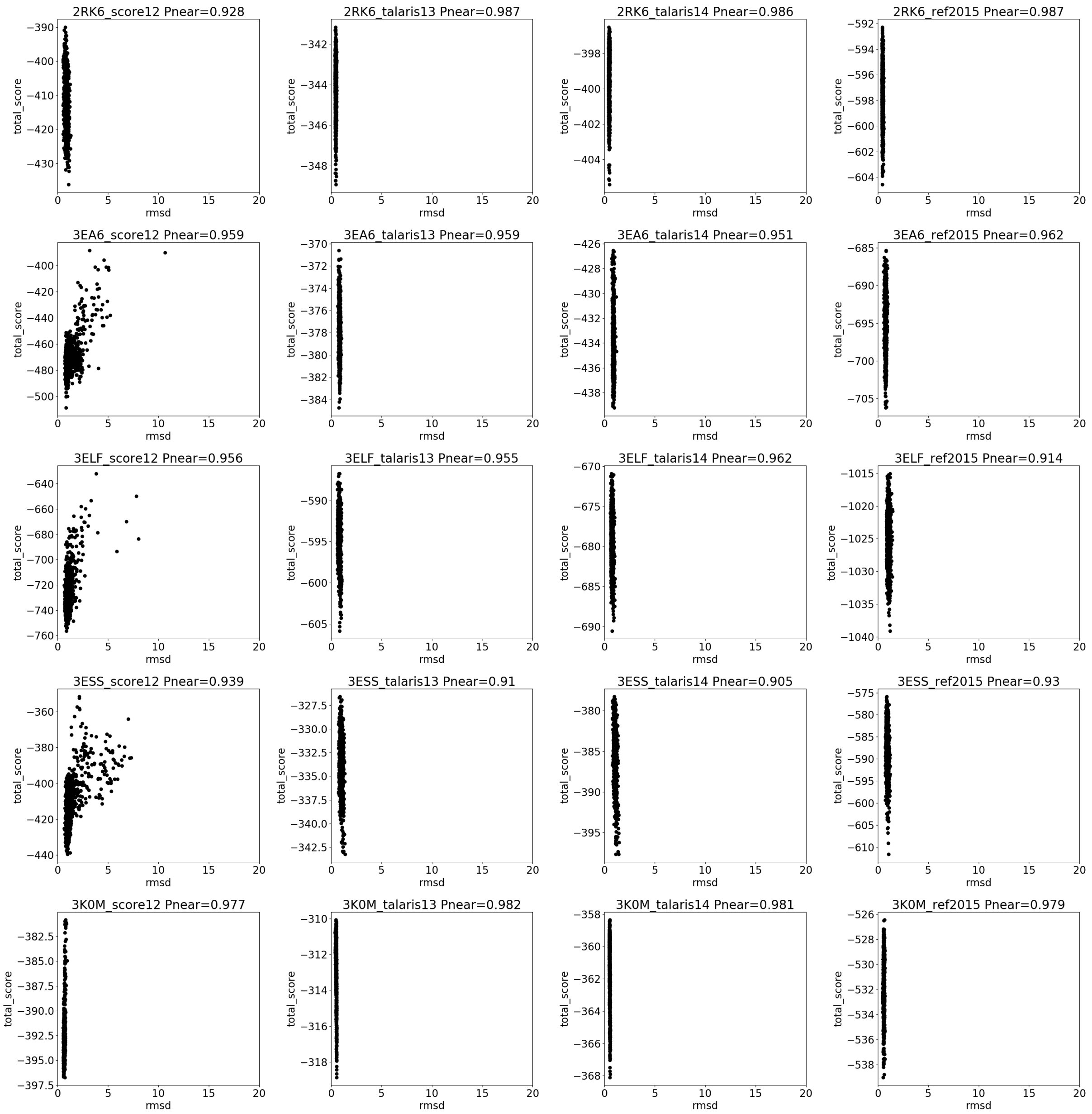

REVISION

revision: 61531

Scientific test: Scorefunction comparison for mp_ddg

RESULTS

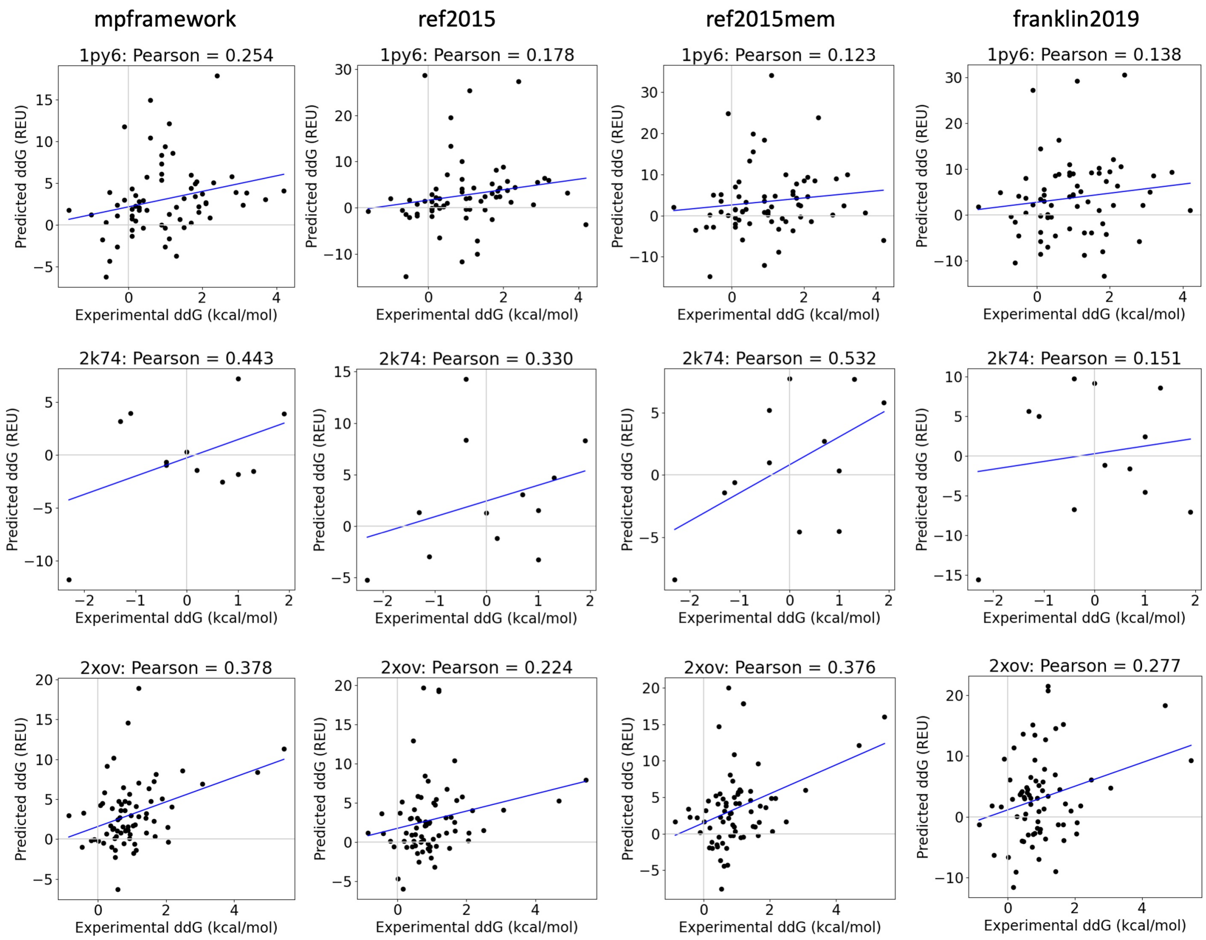

REVISION

revision: 61590

References for the Supplement:

1. Features • GitHub Actions · GitHub. at <https://github.com/features/actions>
2. GitHub. *https://github.com/*
3. Drone CI – Automate Software Testing and Delivery. at <https://www.drone.io/>
4. Travis CI - continuous integration. *https://travis-ci.org/*
5. Jenkins. *https://jenkins.io/*
6. Amazon Web Services (AWS) - Cloud Computing Services. at <https://aws.amazon.com/>
7. Cloud Application Platform | Heroku. at <https://www.heroku.com/>
8. Cloud Computing Services | Google Cloud. at <https://cloud.google.com/>
9. Getting Started - XSEDE. at <https://www.xsede.org/for-users/getting-started>
10. Fleishman, S. J., Leaver-Fay, A., Corn, J. E., Strauch, E.-M. M., Khare, S. D., Koga, N., Ashworth, J., Murphy, P., Richter, F., Lemmon, G., Meiler, J. & Baker, D. RosettaScripts: A scripting language interface to the Rosetta Macromolecular modeling suite. *PLoS One* **6,** 1–10 (2011).
11. Chaudhury, S., Lyskov, S. & Gray, J. J. PyRosetta: a script-based interface for implementing molecular modeling algorithms using Rosetta. *Bioinformatics* **26,** 689–691 (2010).
12. Gray, J. J., Chaudhury, S., Lyskov, S., and Labonte, J. W. The PyRosetta Interactive Platform for Protein Structure Prediction and Design: A Set of Educational Modules. (2014). at <http://www.amazon.com/PyRosetta-Interactive-Platform-Structure-Prediction/dp/1500968277>
13. Koehler Leman, J., Weitzner, B. D., Renfrew, P. D., Lewis, S. M., Moretti, R., Watkins, A. M., Mulligan, V. K., Lyskov, S., Adolf-Bryfogle, J., Labonte, J. W., Krys, J., Bystroff, C., Schief, W., Gront, D., Schueler-Furman, O., Baker, D., Bradley, P., Dunbrack, R., Kortemme, T., Leaver-Fay, A., Strauss, C. E. M., Meiler, J., Kuhlman, B., Gray, J. J. & Bonneau, R. Better together: Elements of successful scientific software development in a distributed collaborative community. *PLOS Comput. Biol.* **16,** e1007507 (2020).
14. RosettaCommons. Rosetta documentation - Scientific Benchmarks. at <http://new.rosettacommons.org/docs/latest/development_documentation/test/Scientific-Benchmarks>
