# Supplementary material for: Ensuring scientific reproducibility in bio-macromolecular modeling via extensive, automated benchmarks
Source: Nat Commun. 2021 Nov 29;12:6947. doi: 10.1038/s41467-021-27222-7 (PMC8630030; doi:10.1038/s41467-021-27222-7)
Supplement: Supplementary file 3 — Reporting Summary [file 41467_2021_27222_MOESM3_ESM.pdf]

nature research

Corresponding author(s): Julia Koehler Leman

Last updated by author(s): 04/18/2021

Reporting Summary

Nature Research wishes to improve the reproducibility of the work that we publish. This form provides structure for consistency and transparency in reporting. For further information on Nature Research policies, see our Editorial Policies and the Editorial Policy Checklist.

Statistics

For all statistical analyses, confirm that the following items are present in the figure legend, table legend, main text, or Methods section.

n/a	Confirmed	
☐	☒	The exact sample size (*n*) for each experimental group/condition, given as a discrete number and unit of measurement
☒	☐	A statement on whether measurements were taken from distinct samples or whether the same sample was measured repeatedly
☒	☐	The statistical test(s) used AND whether they are one- or two-sided<br>*Only common tests should be described solely by name; describe more complex techniques in the Methods section.*
☒	☐	A description of all covariates tested
☒	☐	A description of any assumptions or corrections, such as tests of normality and adjustment for multiple comparisons
☐	☒	A full description of the statistical parameters including central tendency (e.g. means) or other basic estimates (e.g. regression coefficient) AND variation (e.g. standard deviation) or associated estimates of uncertainty (e.g. confidence intervals)
☒	☐	For null hypothesis testing, the test statistic (e.g. *F*, *t*, *r*) with confidence intervals, effect sizes, degrees of freedom and *P* value noted<br>*Give P values as exact values whenever suitable.*
☒	☐	For Bayesian analysis, information on the choice of priors and Markov chain Monte Carlo settings
☒	☐	For hierarchical and complex designs, identification of the appropriate level for tests and full reporting of outcomes
☐	☒	Estimates of effect sizes (e.g. Cohen's *d*, Pearson's *r*), indicating how they were calculated

Our web collection on statistics for biologists contains articles on many of the points above.

Software and code

Policy information about availability of computer code

Data collection	Rosetta is licensed and distributed through https://www.rosettacommons.org. Licenses for academic, non-profit and government laboratories are free of charge; there is a license fee for industry users. A license is required to gain access to the Github repository. Specific version numbers are given in the supplement.
Data analysis	Exact details are given in the supplement, available for download at https://graylab.jhu.edu/download/rosetta-scientific-tests/ which includes exact analyses performed. All software versions for data analysis are listed in the Rosetta repository in /Rosetta/main/tests/benchmark/tests/command.py

For manuscripts utilizing custom algorithms or software that are central to the research but not yet described in published literature, software must be made available to editors and reviewers. We strongly encourage code deposition in a community repository (e.g. GitHub). See the Nature Research guidelines for submitting code & software for further information.

Data

Policy information about availability of data

All manuscripts must include a data availability statement. This statement should provide the following information, where applicable:

- Accession codes, unique identifiers, or web links for publicly available datasets
- A list of figures that have associated raw data
- A description of any restrictions on data availability

Datasets for all 42 scientific benchmarks are publicly available for download at https://graylab.jhu.edu/download/rosetta-scientific-tests/. Access is without restriction. Accession codes, if applicable are provided by following the link and navigating to the desired benchmark.

Field-specific reporting

Please select the one below that is the best fit for your research. If you are not sure, read the appropriate sections before making your selection.

- [x] Life sciences
- [ ] Behavioural & social sciences
- [ ] Ecological, evolutionary & environmental sciences

For a reference copy of the document with all sections, see nature.com/documents/nr-reporting-summary-flat.pdf

Life sciences study design

All studies must disclose on these points even when the disclosure is negative.

Sample size	Sample sizes vary for each benchmark and are provided at https://graylab.jhu.edu/download/rosetta-scientific-tests/
Data exclusions	Data exclusions vary for each benchmark and are provided at https://graylab.jhu.edu/download/rosetta-scientific-tests/
Replication	All data is being replicated on a continuous basis, with 2 replicates existing for all benchmarks at the time of paper acceptance.
Randomization	Randomizations vary for each benchmark and are provided at https://graylab.jhu.edu/download/rosetta-scientific-tests/
Blinding	Blinding vary for each benchmark and are provided at https://graylab.jhu.edu/download/rosetta-scientific-tests/

Reporting for specific materials, systems and methods

We require information from authors about some types of materials, experimental systems and methods used in many studies. Here, indicate whether each material, system or method listed is relevant to your study. If you are not sure if a list item applies to your research, read the appropriate section before selecting a response.

Materials & experimental systems

n/a	Involved in the study	
x		Antibodies
x		Eukaryotic cell lines
x		Palaeontology and archaeology
x		Animals and other organisms
x		Human research participants
x		Clinical data
x		Dual use research of concern

Methods

n/a	Involved in the study	
x		ChIP-seq
x		Flow cytometry
x		MRI-based neuroimaging
